# Supplementary material for: Dysregulated Wnt and NFAT signaling in a Parkinson’s disease LRRK2 G2019S knock-in model
Source: Sci Rep. 2024 May 29;14:12393. doi: 10.1038/s41598-024-63130-8 (PMC11137013; doi:10.1038/s41598-024-63130-8)

Supplementary material 1: Dysregulated Wnt and NFAT signaling in a Parkinson's disease LRRK2 G2019S knock-in model

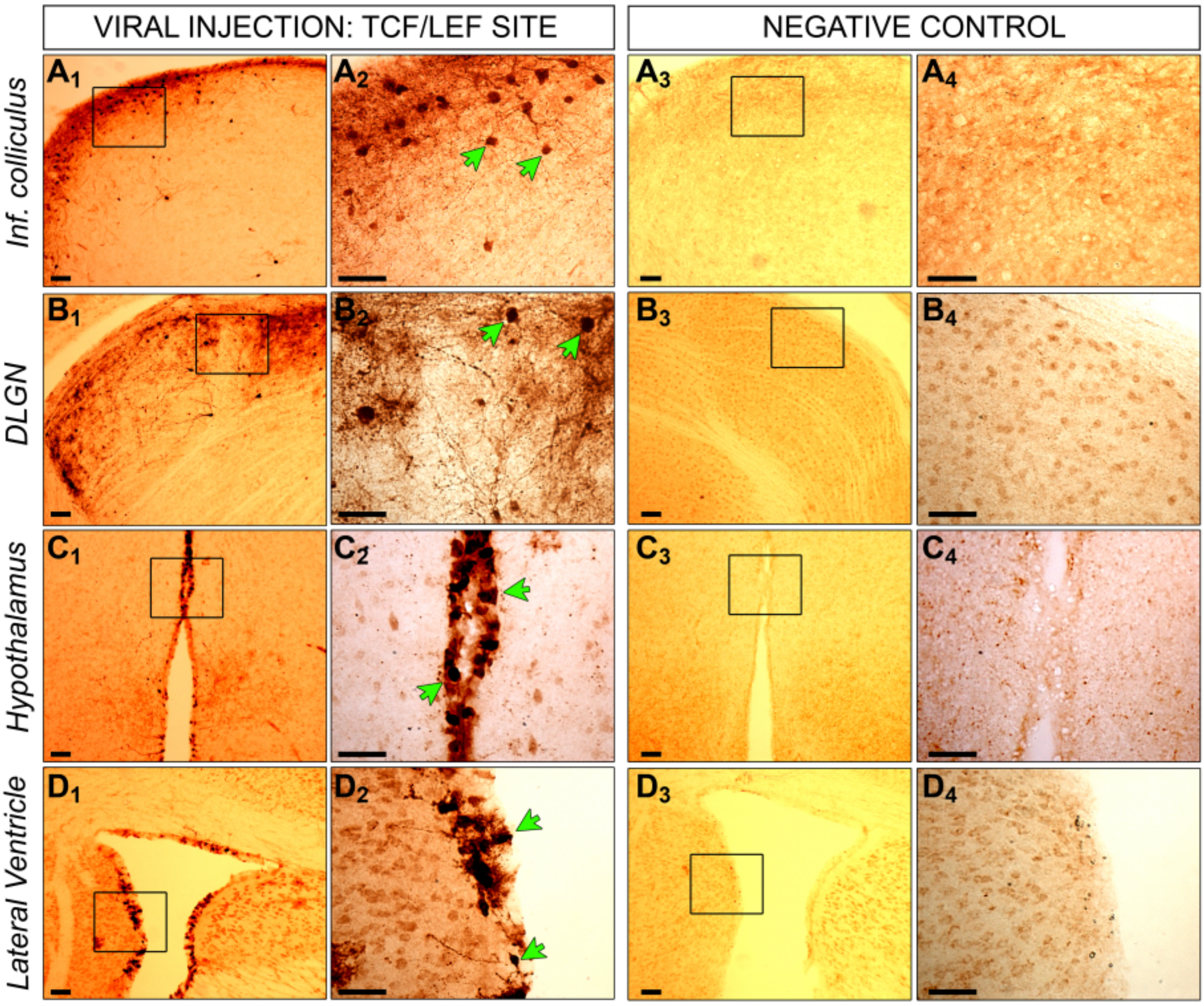

Figure S1. GFP staining of injected and non-injected mice with the TCF/LEF biosensor

Mice were injected intracranially at P0 with a lentiviral construct, containing a RE site for TCF/LEF and a second gene for GFP. Six months later, brains were collected from injected (**1** and **2**) and non-injected control mice (**3** and **4**). Brains were fixed in PFA, coronally cryosectioned into 40µm thick slices, which were stained via immunohistochemistry for GFP. Positive cells for GFP expression are visible in dark brown indicated by the green arrows. Positive cells were detectable in inferior colliculus (**A**), dorsal lateral geniculate nucleus (**B**), hypothalamus (**C**) and lateral ventricle (**D**). No positive cells were measurable in corresponding regions of non-injected control brains. Images in **2** and **4** represent the zoom of indicated squares in **1** and **3**. Scale bar for all images is 50µm.

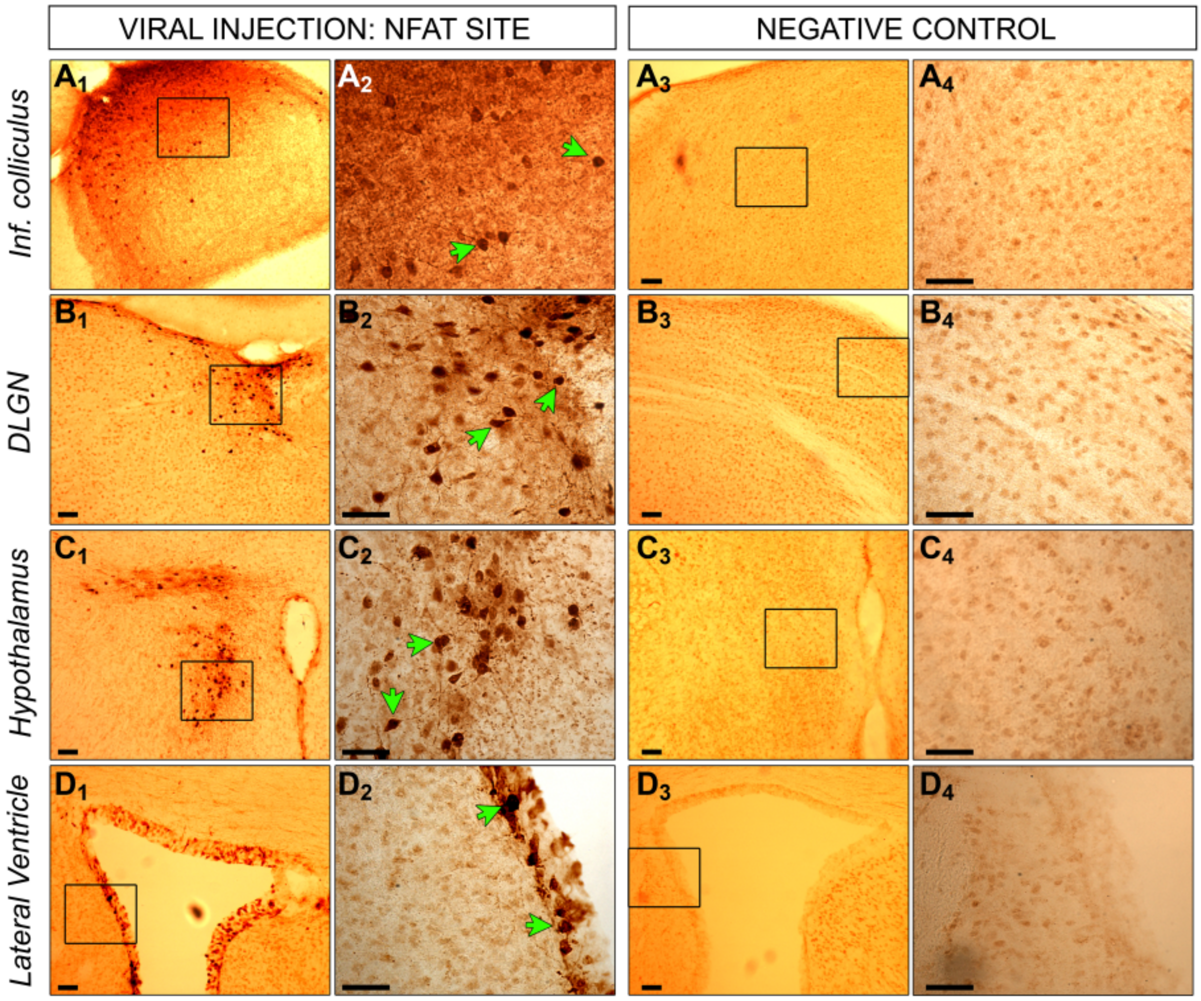

Figure S2. GFP staining of injected and non-injected mice with the NFATc1 biosensor

Mice were injected intracranially at P0 with a lentiviral construct, containing a RE site for NFATc1 and a second gene for GFP. Six months later, brains were collected from injected (**1** and **2**) and non-injected control mice (**3** and **4**). Brains were fixed in PFA, coronally cryosectioned into 40µm thick slices, which were stained via immunohistochemistry for GFP. Positive cells for GFP expression are visible in dark brown indicated by the green arrows. Positive cells were detectable in inferior colliculus (**A**), dorsal lateral geniculate nucleus (**B**), hypothalamus (**C**) and lateral ventricle (**D**). No positive cells were measurable in corresponding regions of non-injected control brains. Images in **2** and **4** represent the zoom of indicated squares in **1** and **3**. Scale bar for all images is 50µm.

	mRNA	Half Brain			Cortex			Striatum			Hippocampus		
	**LRRK2 KO**	Male	Female	Mixed	Male	Female	Mixed	Male	Female	Mixed	Male	Female	Mixed
**Wnt ligands**	***Wnt5a***			↓		↓	↓	↑		↑ P=0.06			
	Wnt7a												
**β-catenin destruction complex**	***Gsk3β***	↓	↓	↓↓				↑ P=0.09					
	β-catenin			↓				↑ P=0.05		↑ P=0.05			
	Axin2			↓				↑		↑			
**Transcriptional factors**	***Nfat***							↑ P=0.06		↑			
	Tcf1	↓ P=0.05			↑ P=0.05			↑					
**Downstream targets**	***Bdnf***				↓	↓ P=0.05	↓						

Supplementary Table 1.1: Summary table of significant mRNA expression changes in different brain regions in LRRK2 KO mouse tissue compared to WT mice.

↑ Indicates an upregulation compared to WT; ↓ indicates a downregulation compared to WT.

	mRNA	Half Brain			Cortex			Striatum			Hippocampus		
	**G2019S KI**	Male	Female	Mixed	Male	Female	Mixed	Male	Female	Mixed	Male	Female	Mixed
**Wnt ligands**	***Wnt5a***			↓ P=0.06				↑ P=0.08					
	Wnt7a			↑									
**β-catenin destruction complex**	***Gsk3β***		↑ p=0.08						↓				
	β-catenin							↑		↑ P=0.08			
	Axin2							↑↑		↑ P=0.08			
**Transcriptional factors**	***Nfat***												
	Tcf1							↑					
**Downstream targets**	***Bdnf***					↓	↓						

Supplementary Table 1.2: Summary table of significant mRNA expression changes in different brain regions in LRRK2 G2019S KI mouse tissue compared to WT mice.

↑ Indicates an upregulation compared to WT; ↓ indicates a downregulation compared to WT.

	Protein	Half Brain			Cortex			Striatum			Hippocampus		
	**LRRK2 KO**	Male	Female	Mixed	Male	Female	Mixed	Male	Female	Mixed	Male	Female	Mixed
**Genotype**	LRRK2												
**Wnt ligands**	Wnt3a	↓			↓		↓					↑ P=0.09	
	Wnt5a	↓		↓ P=0.07				↓		↓			
**Co-receptor**	pLRP6				↓ P=0.06		↓				↓		
	LRP6										↓↓		↓ P=0.07
	pLRP6/LRP6				↓		↓						
**β-catenin destruction complex**	pGsk3β			↓ P=0.09	↑ P=0.09	↓ P=0.06							
	Gsk3β				↓	↑							
	pGsk3β/Gsk3β				↑								
	Active-β-catenin						↑ P=0.08					↓	↓ P=0.06
	β-catenin					↑						↑↑	↑↑
	ABC/β-catenin				↑ P=0.08							↓	↓
**Transcriptional factors**	NFAT		↓		↓		↓ p=0.06	↓ P=0.07			↓		
	Tcf1		↑ P=0.07	↑ P=0.05							↓		↓↓
	Lef1	↓	↑ P=0.07		↓↓		↓↓						
**Downstream targets**	BDNF	↑		↑ P=0.05							↑ P=0.08		

Supplementary Table 2.1: Summary table of significant protein expression changes in different brain regions in LRRK2 KO mouse tissue compared to WT mice.

↑ Indicates an upregulation compared to WT; ↓ indicates a downregulation compared to WT.

	Protein	Half Brain			Cortex			Striatum			Hippocampus		
	**G2019S KI**	Male	Female	Mixed	Male	Female	Mixed	Male	Female	Mixed	Male	Female	Mixed
**Genotype**	LRRK2												↑ P=0.09
**Wnt ligands**	Wnt3a					↑ P=0.09	↑			↑ P=0.06			
	Wnt5a		↑	↑↑		↓↓				↓ P=0.08			
**Co-receptor**	pLRP6	↓	↓↓	↓↓↓		↓↓							
	LRP6	↑		↑ P=0.08	↓	↓↓	↓	↓ P=0.07	↓	↓↓	↓		
	pLRP6/LRP6	↓	↓	↓↓↓		↑↑	↑						
**β-catenin destruction complex**	pGsk3β	↓			↓ P=0.05	↓	↓↓↓						
	Gsk3β							↑ P=0.05		↑			
	pGsk3β/Gsk3β						↓ P=0.06	↓ P=0.06		↓			
	Active-β-catenin							↓ P=0.09		↓ P=0.05			
	β-catenin							↓		↓↓			↑
	ABC/β-catenin											↓ P=0.05	
**Transcriptional factors**	NFAT							↓	↓ p=0.09	↓↓			
	Tcf1		↑↑↑	↑					↑			↓	↓↓
	Lef1				↓	↑ P=0.07		↓↓		↓↓			
**Downstream targets**	BDNF	↓	↓	↓↓↓									

Supplementary Table 2.2: Summary table of significant mRNA expression changes in different brain regions in LRRK2 G2019S KI mouse tissue compared to WT mice.

↑ Indicates an upregulation compared to WT; ↓ indicates a downregulation compared to WT.

Western Blot images:

Note: *All membranes were cut prior to primary antibody incubation to minimize the use of reagents, ensuring sustainability. Depending on the size of the protein gels and corresponding membranes, some of the membrane edges are not fully visible as they were outside the imaging field. However, membrane images below are shown as originally imaged using a Syngene GeneGnome Imaging system.*

Western Blots corresponding to the main figure Fig 2 H-M:

Western Blots Analyzed in Fig 2 H-M.

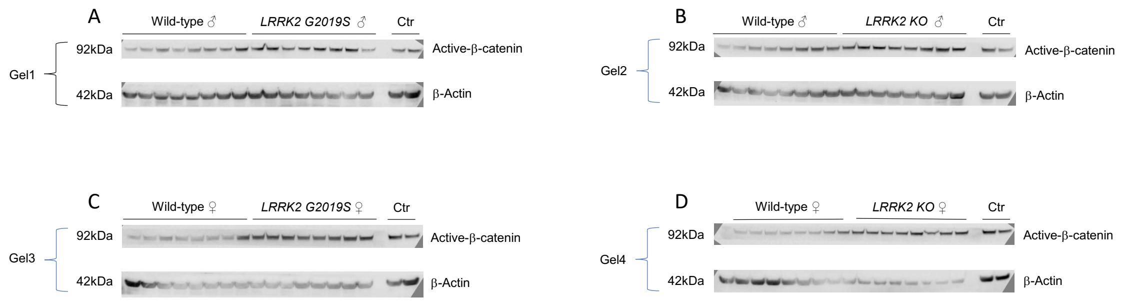

Supplement: Supplementary file 1 — Supplementary Information 1. [file 41598_2024_63130_MOESM1_ESM.pdf]
