# Supplementary material for: Dysregulated Wnt and NFAT signaling in a Parkinson’s disease LRRK2 G2019S knock-in model
Source: Sci Rep. 2024 May 29;14:12393. doi: 10.1038/s41598-024-63130-8 (PMC11137013; doi:10.1038/s41598-024-63130-8)

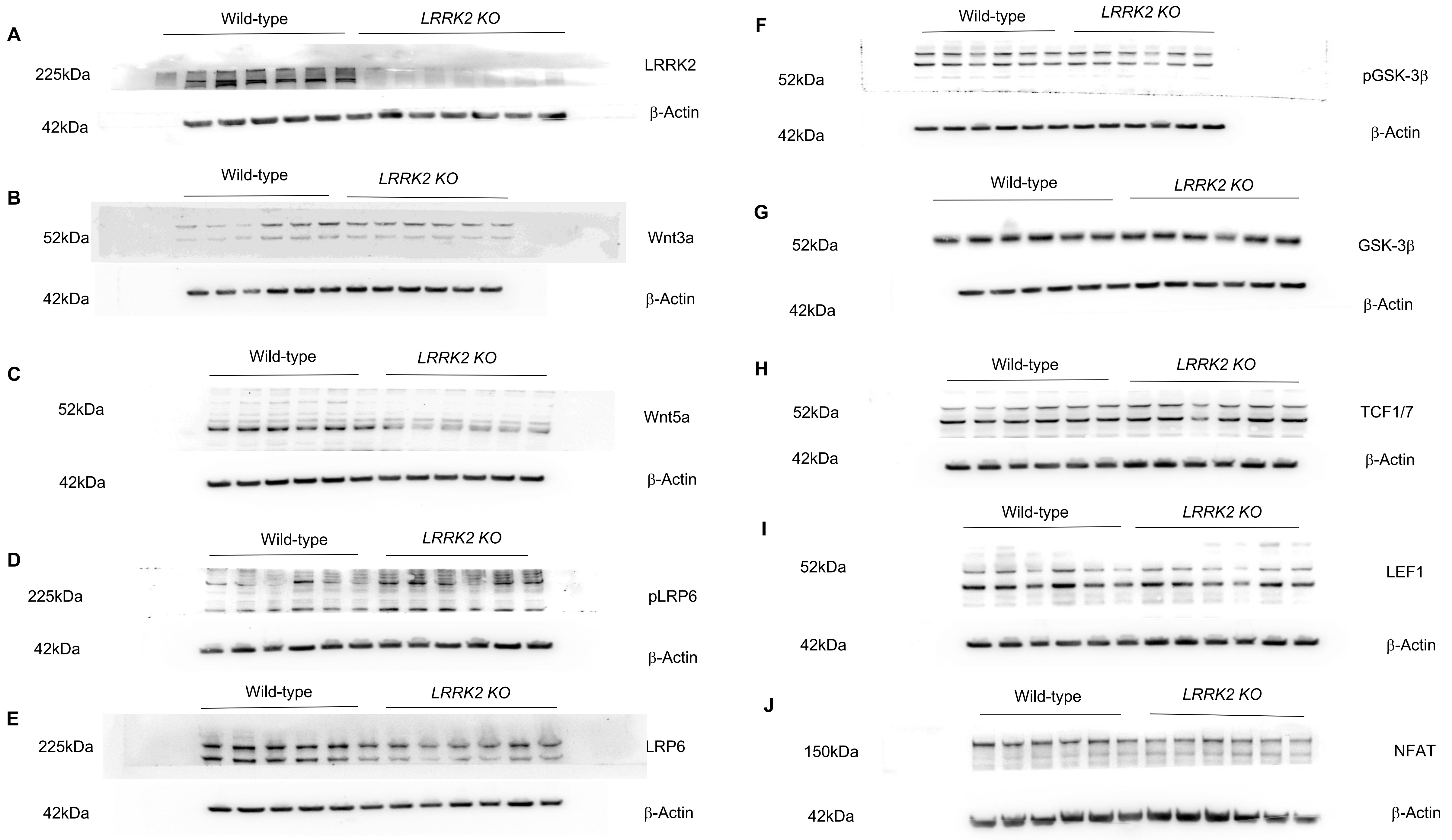
A
Wild-type
LRRK2 KO
225kDa
LRRK2
42kDa
β-Actin
B
Wild-type
LRRK2 KO
52kDa
Wnt3a
42kDa
β-Actin
C
Wild-type
LRRK2 KO
52kDa
Wnt5a
42kDa
β-Actin
D
Wild-type
LRRK2 KO
225kDa
pLRP6
42kDa
β-Actin
E
Wild-type
LRRK2 KO
225kDa
LRP6
42kDa
β-Actin
F
Wild-type
LRRK2 KO
52kDa
pGSK-3β
42kDa
β-Actin
G
Wild-type
LRRK2 KO
52kDa
GSK-3β
42kDa
β-Actin
H
Wild-type
LRRK2 KO
52kDa
TCF1/7
42kDa
β-Actin
I
Wild-type
LRRK2 KO
52kDa
LEF1
42kDa
β-Actin
J
Wild-type
LRRK2 KO
150kDa
NFAT
42kDa
β-Actin

K

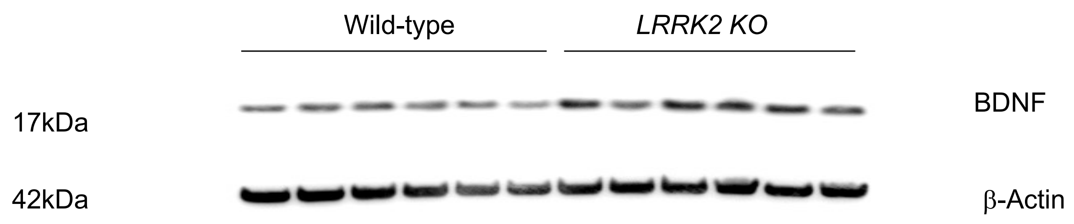

A

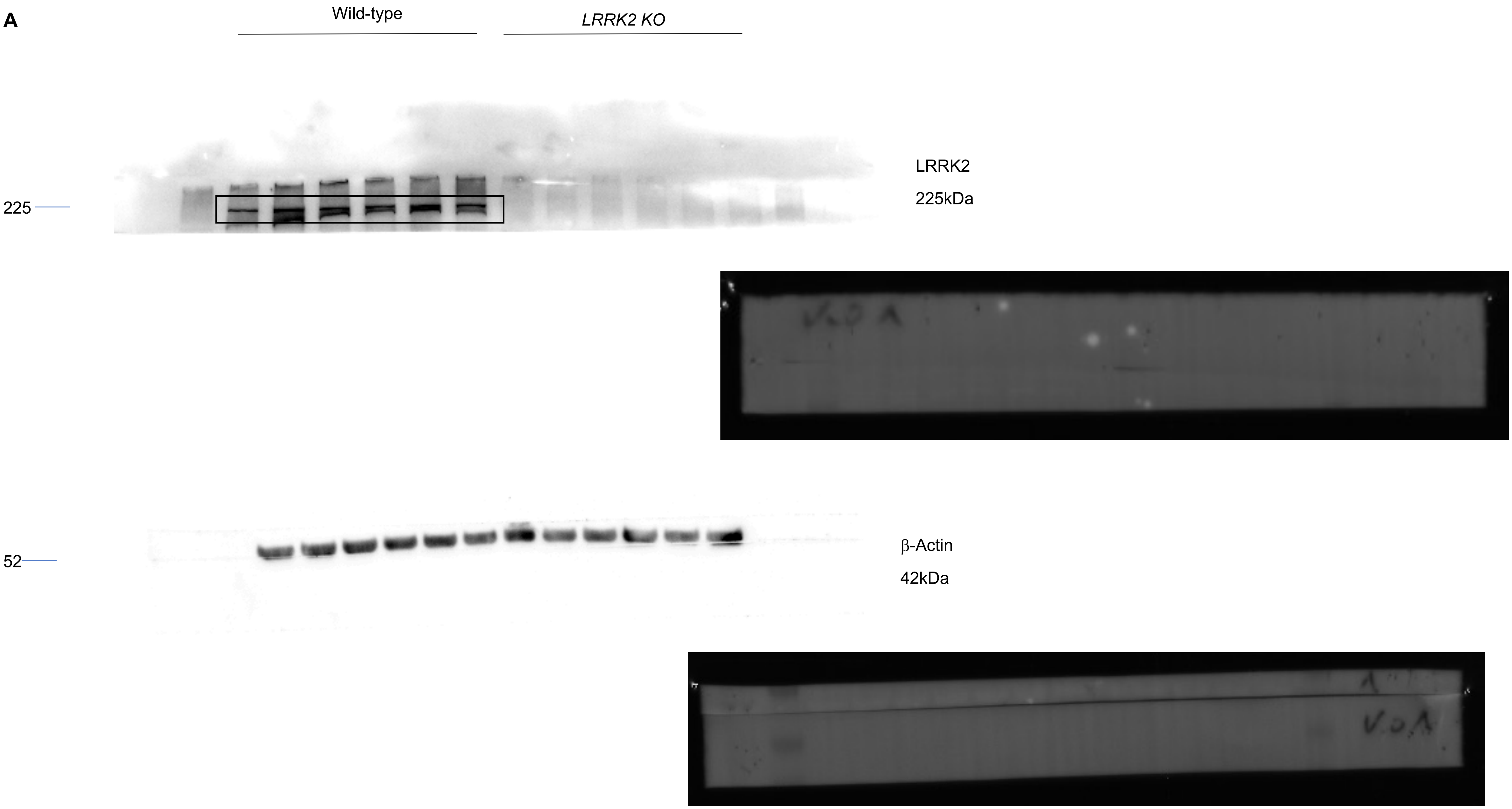

B

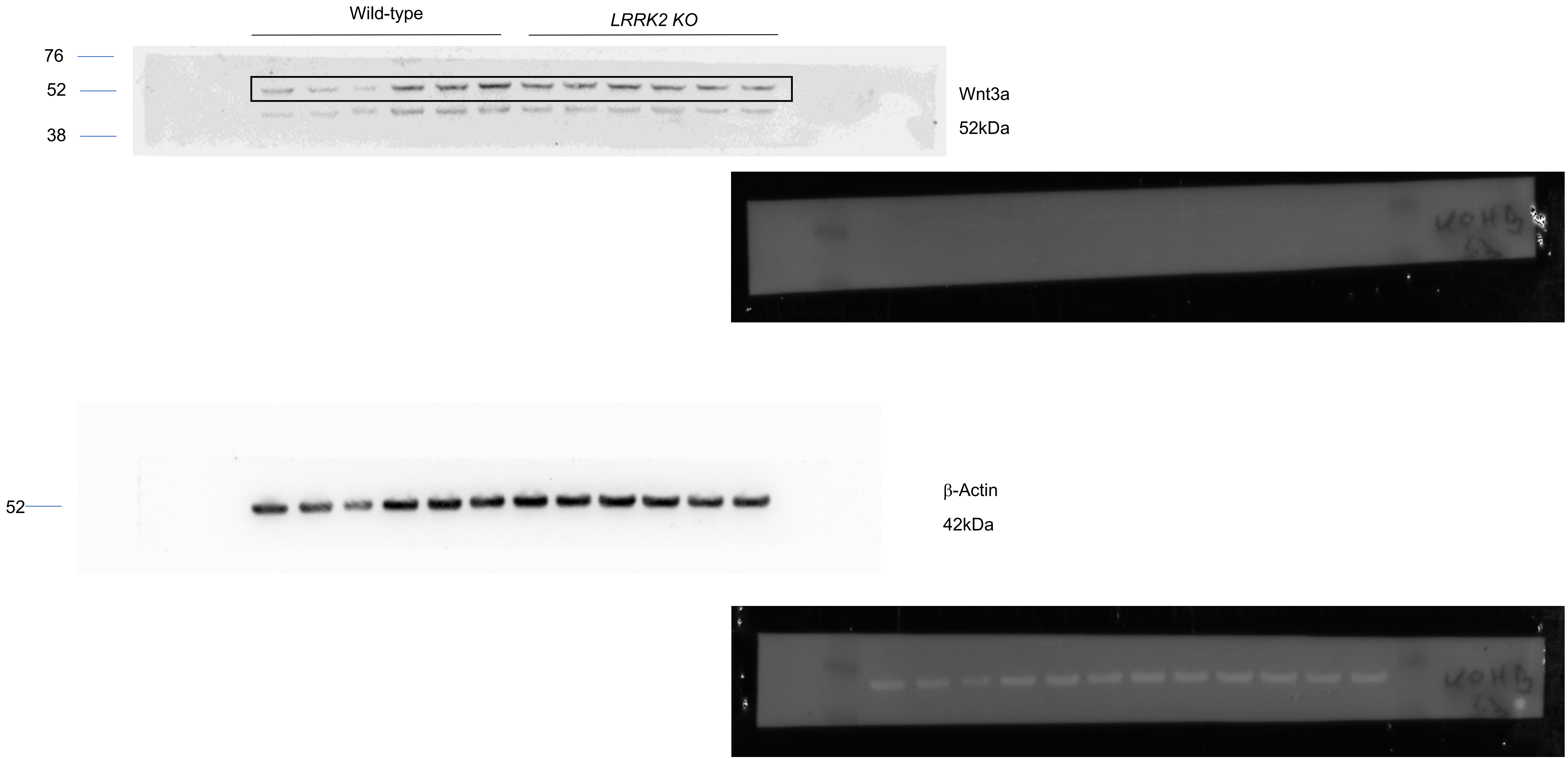

C

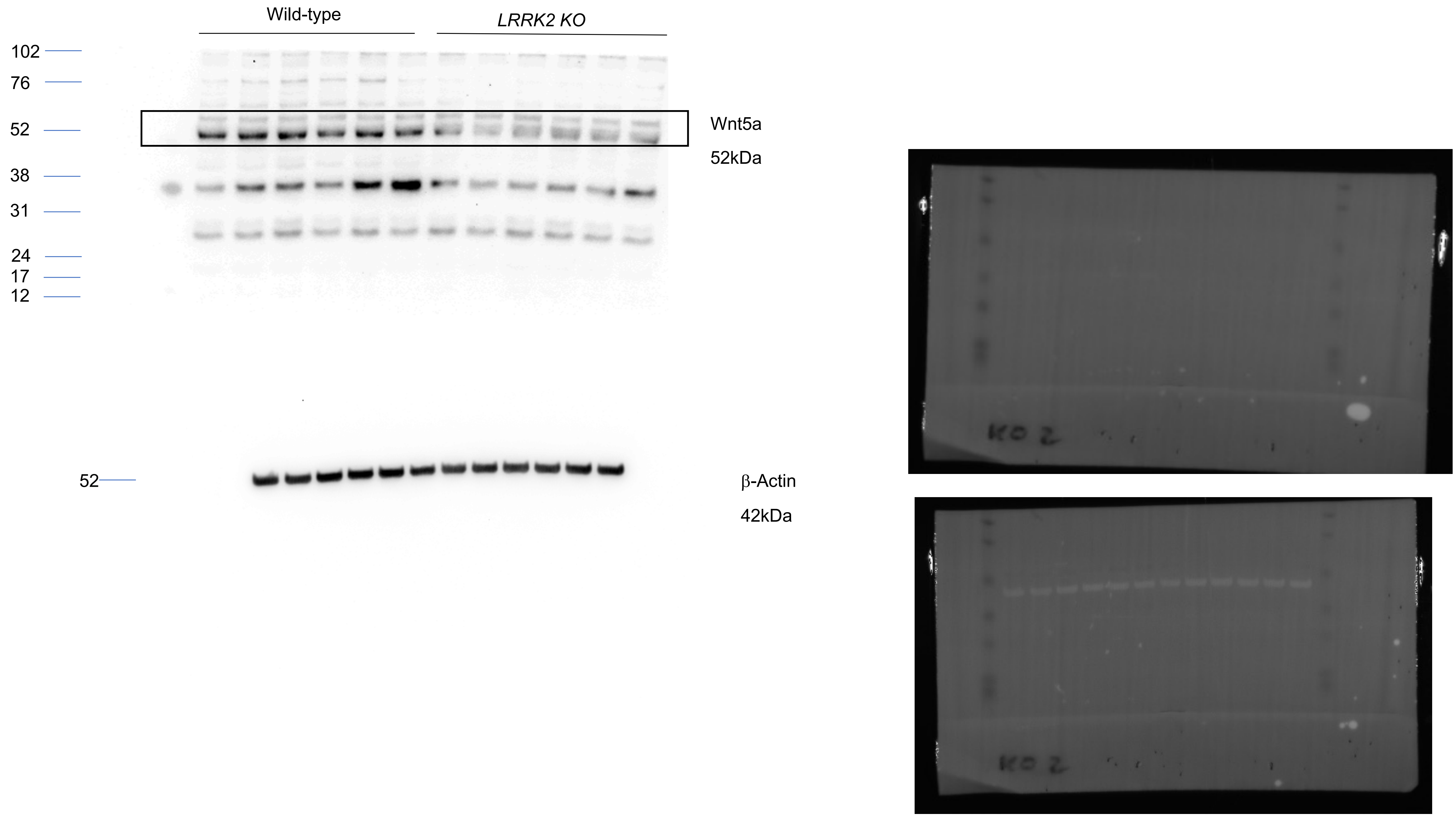
Wild-type
LRRK2 KO
102
76
52
38
31
24
17
12
Wnt5a
52kDa
52
β-Actin
42kDa

D

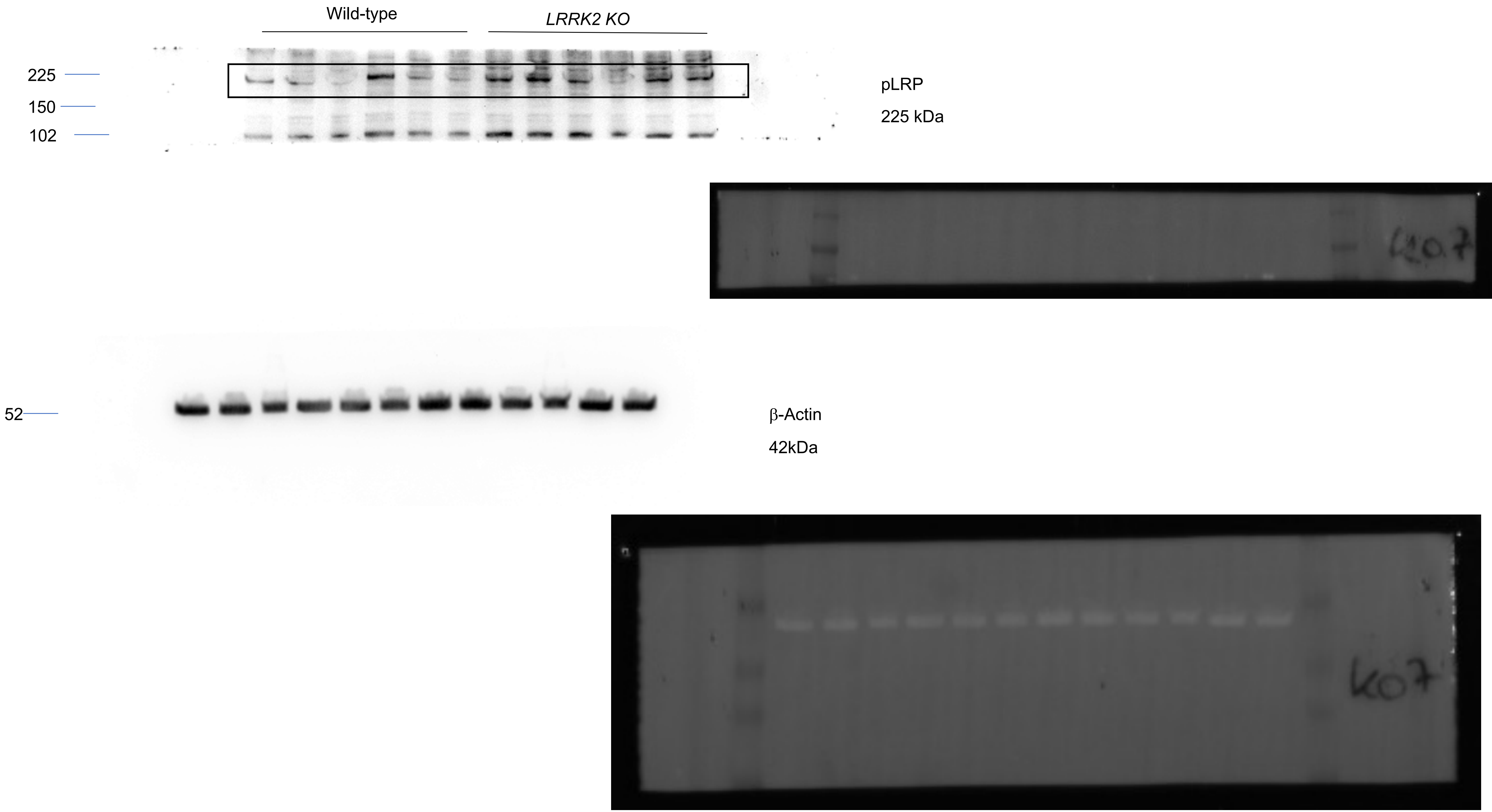

E

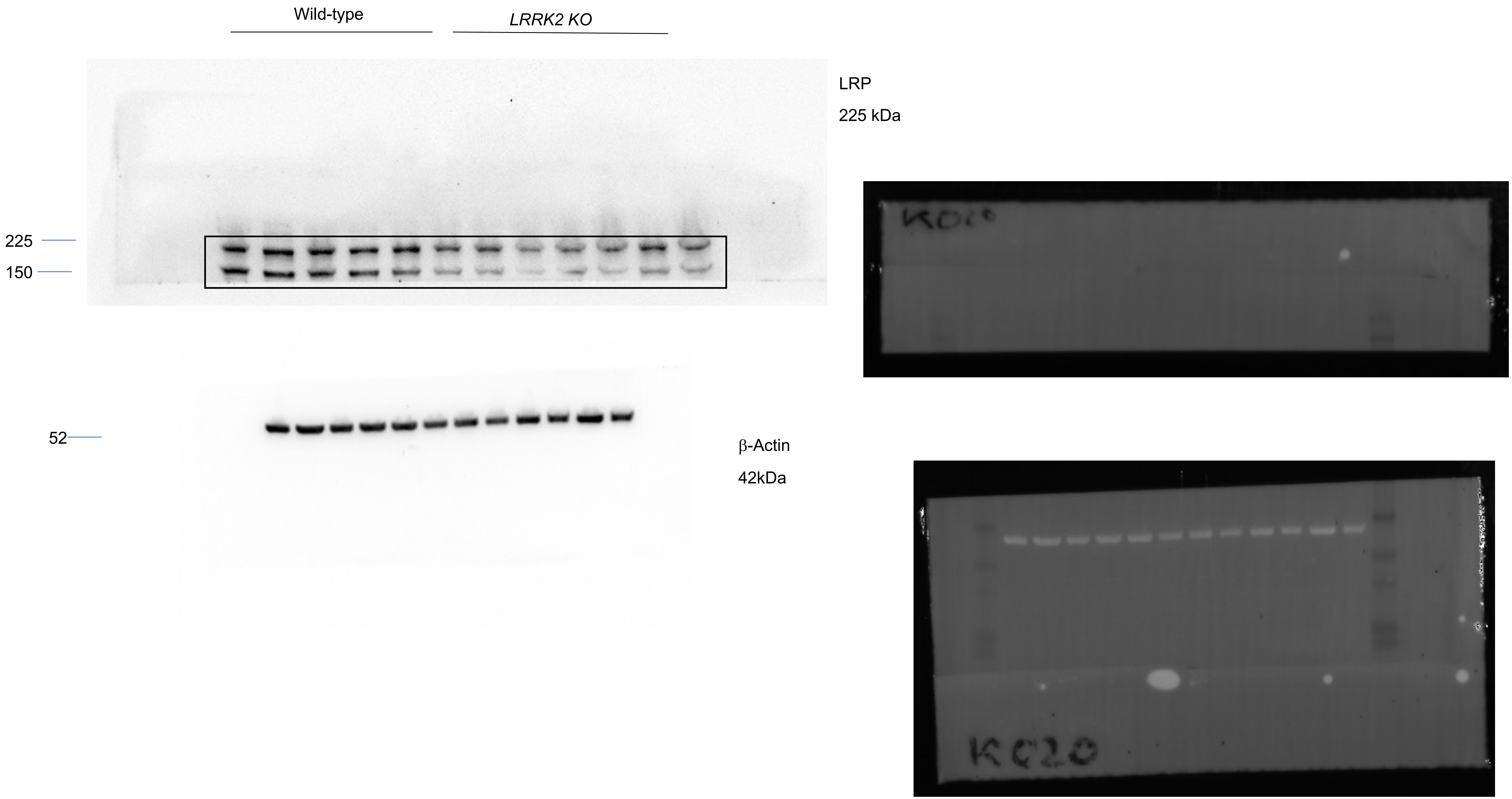

Wild-type
LRRK2 KO
LRP
225 kDa
225
150
52
β-Actin
42kDa
KO20
KO20

F

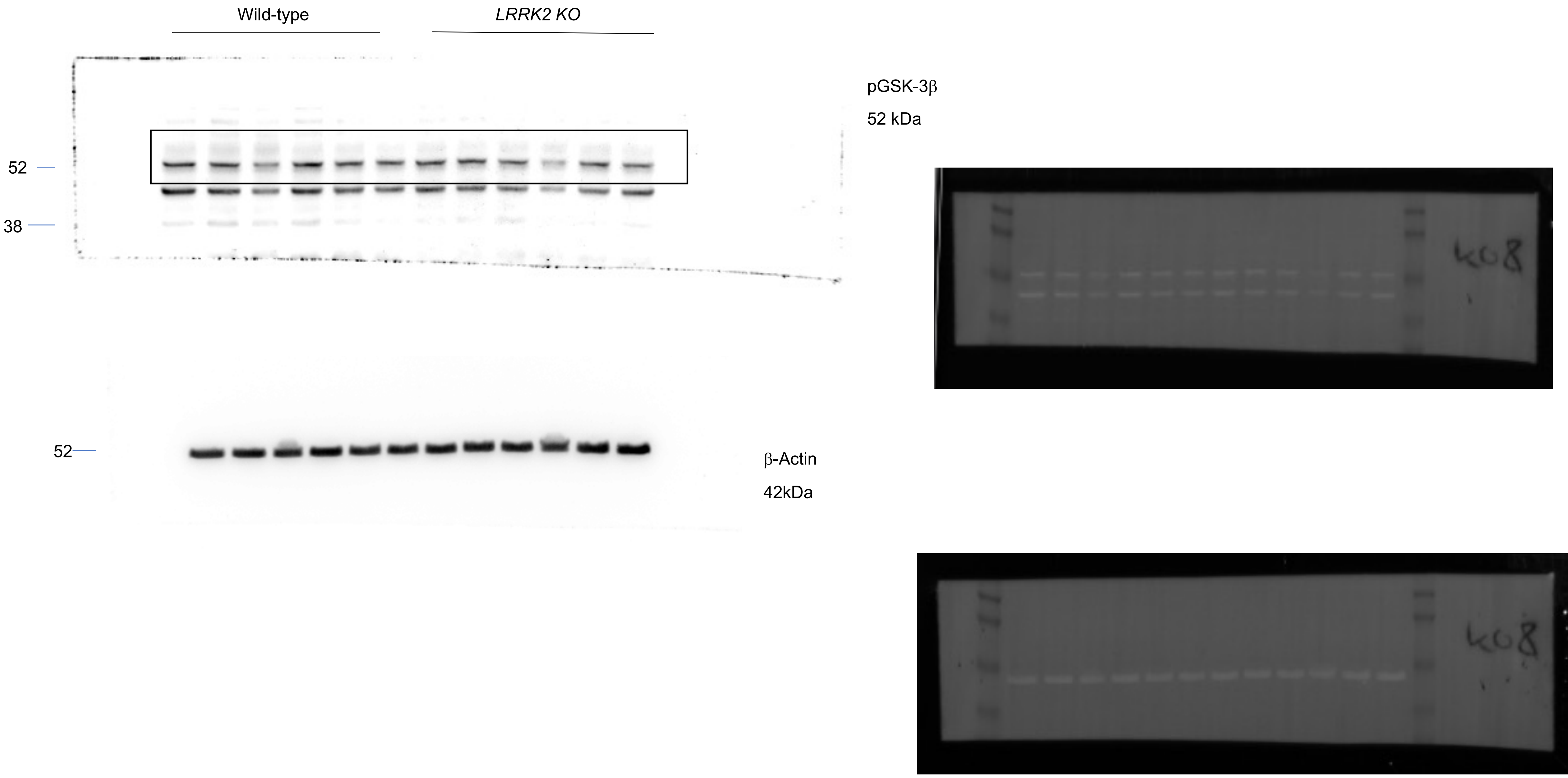

G

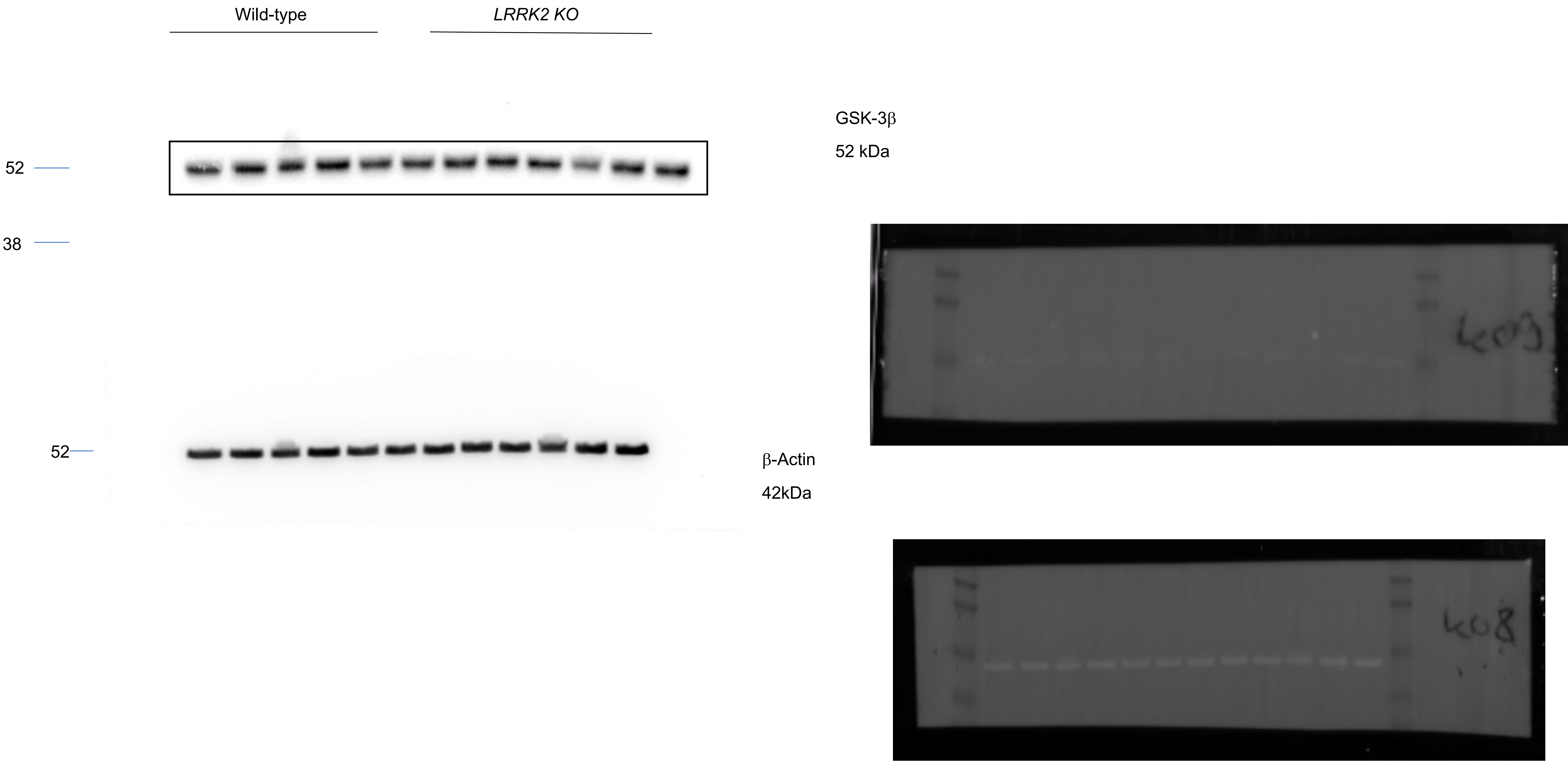

H

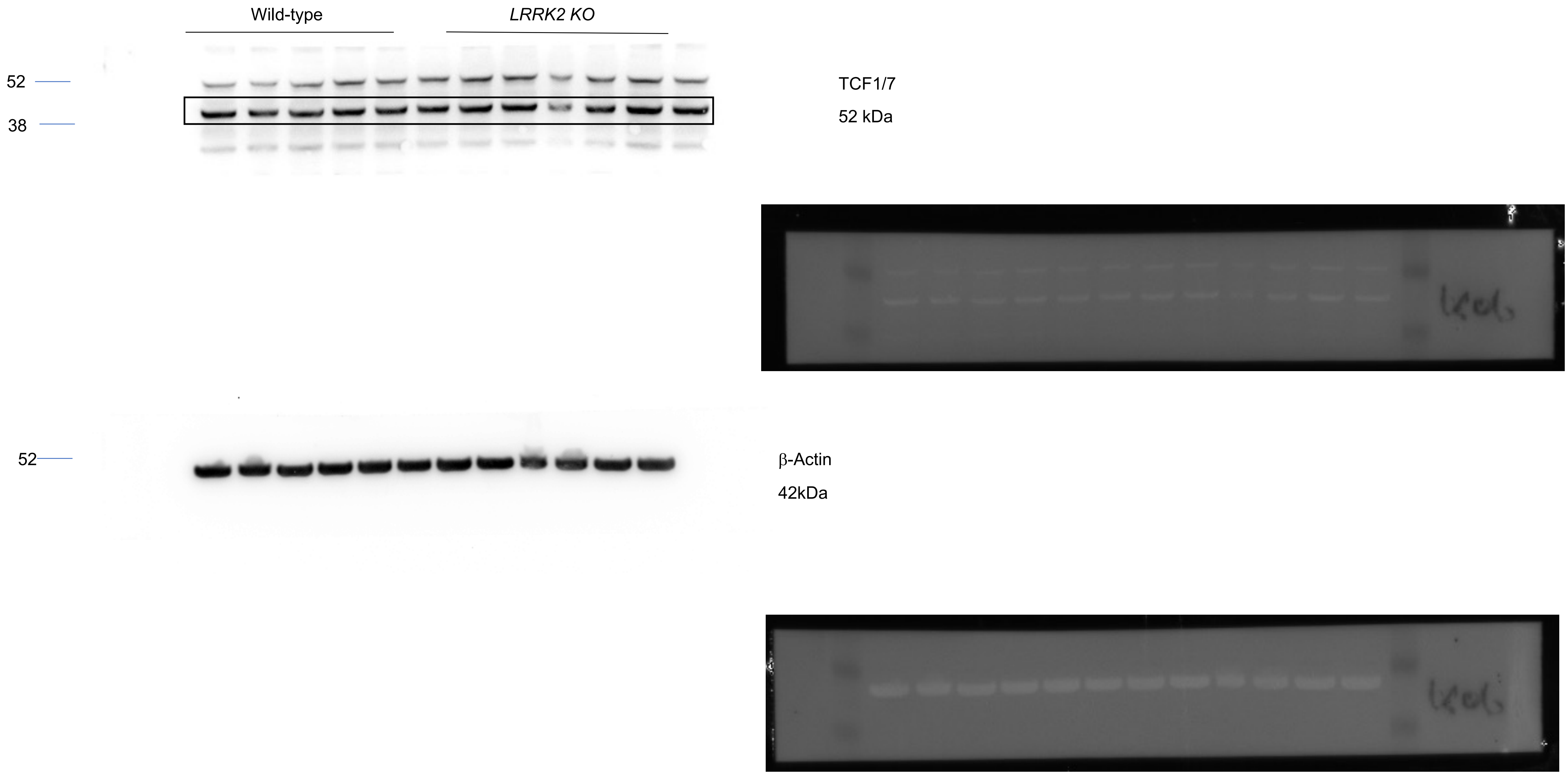

I

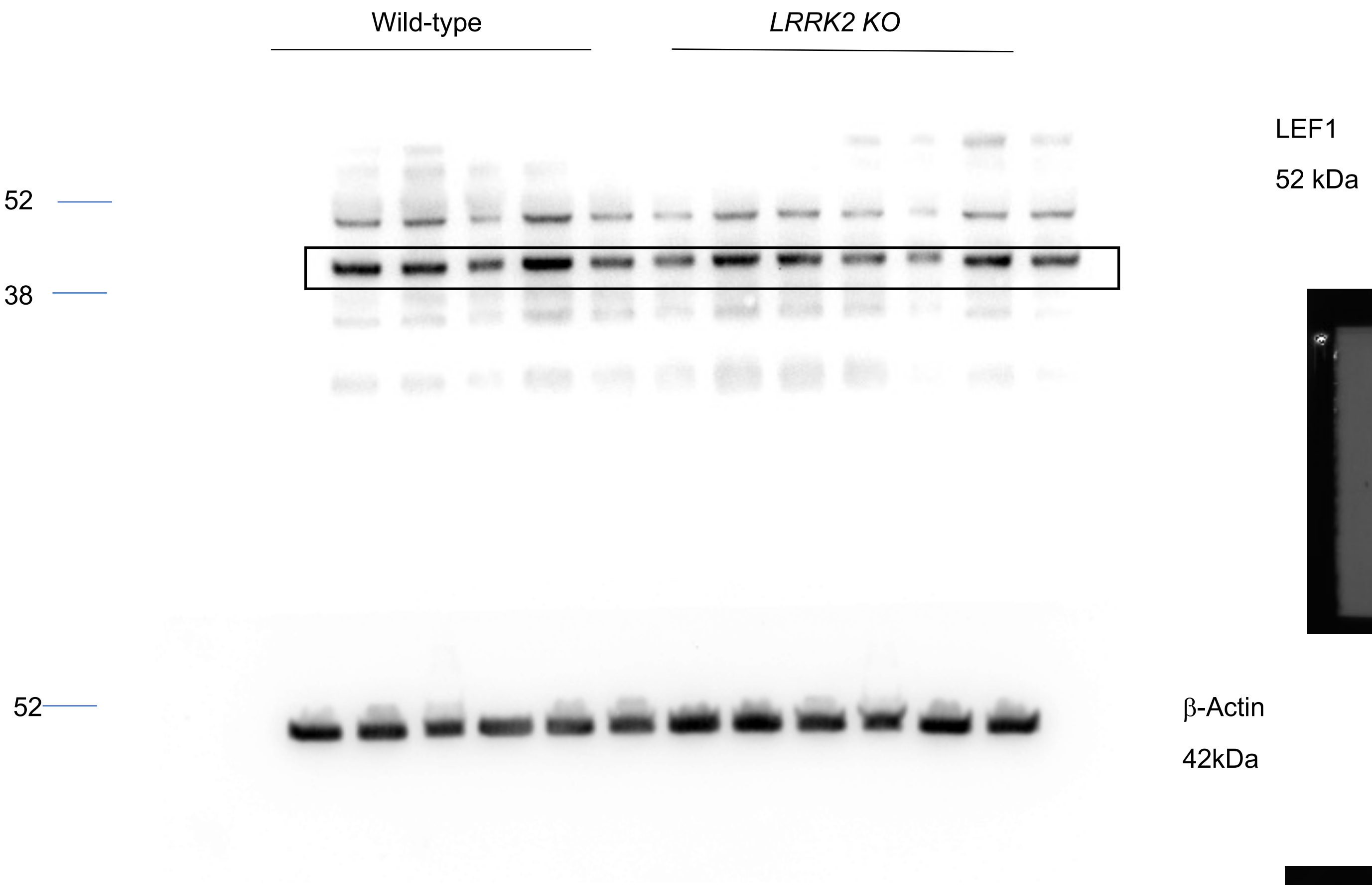

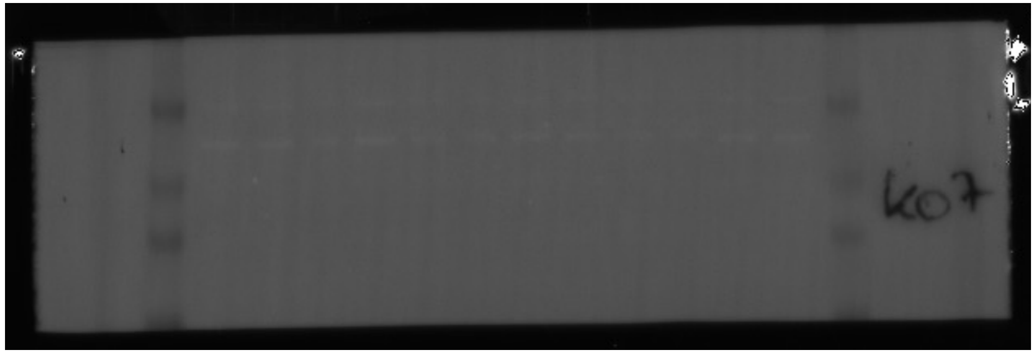

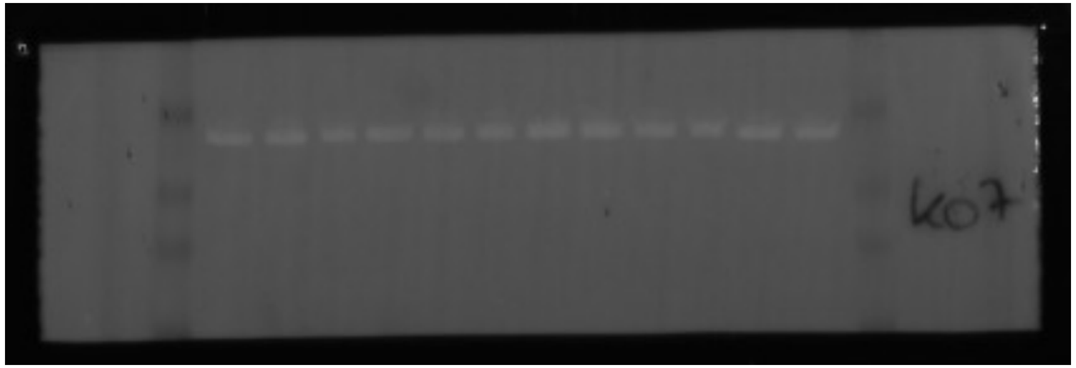

J

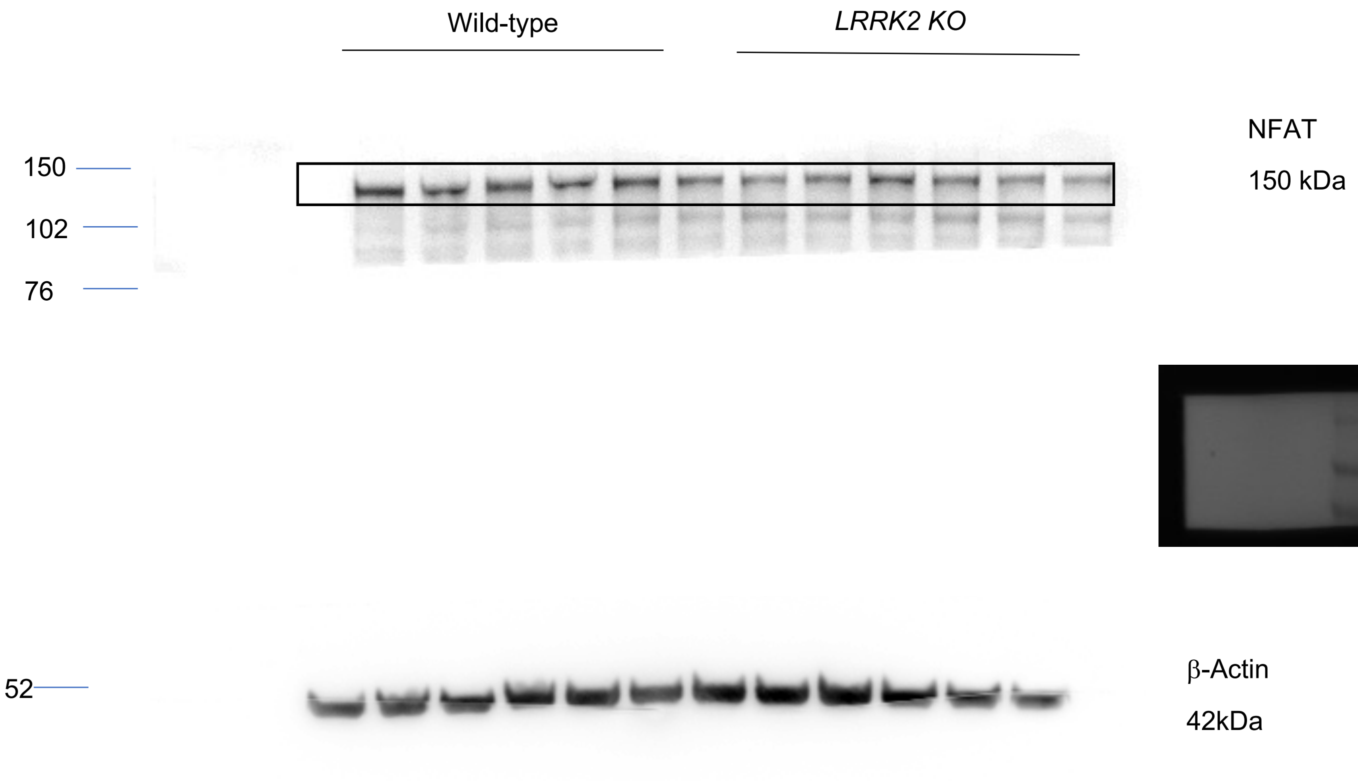

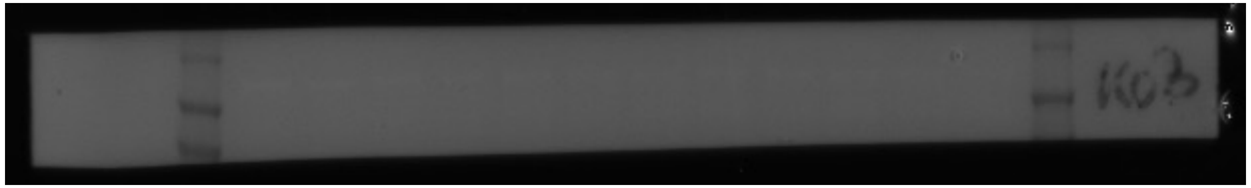

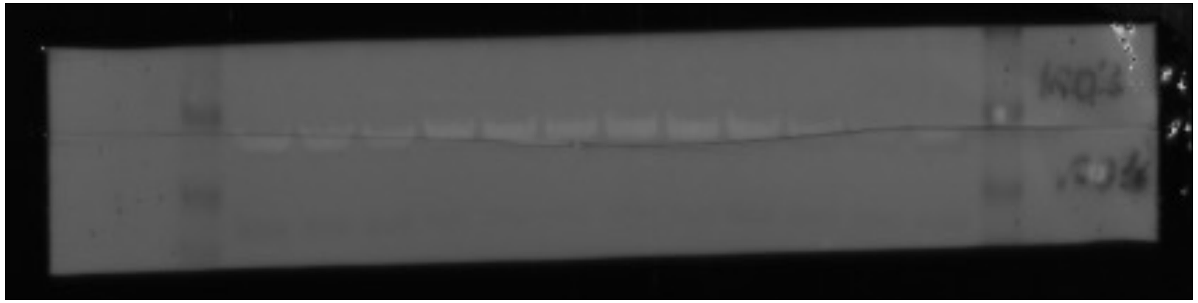

K

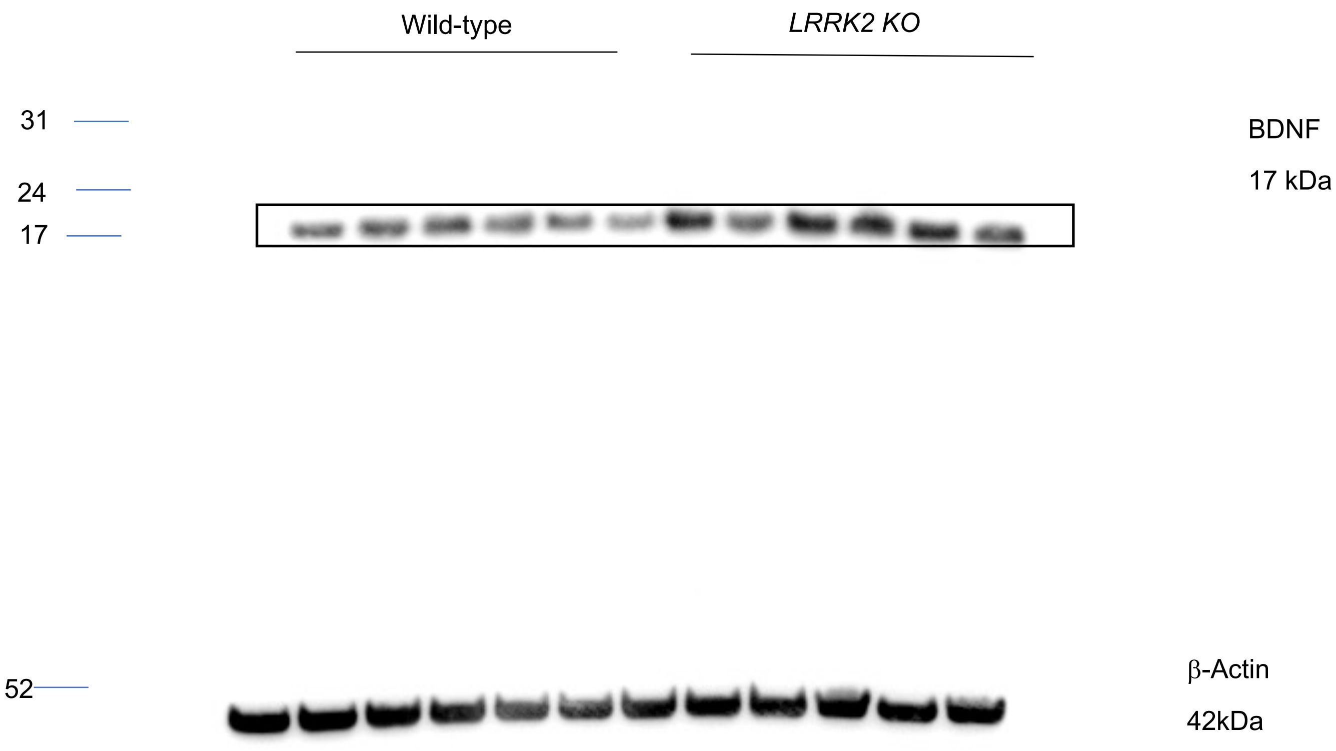

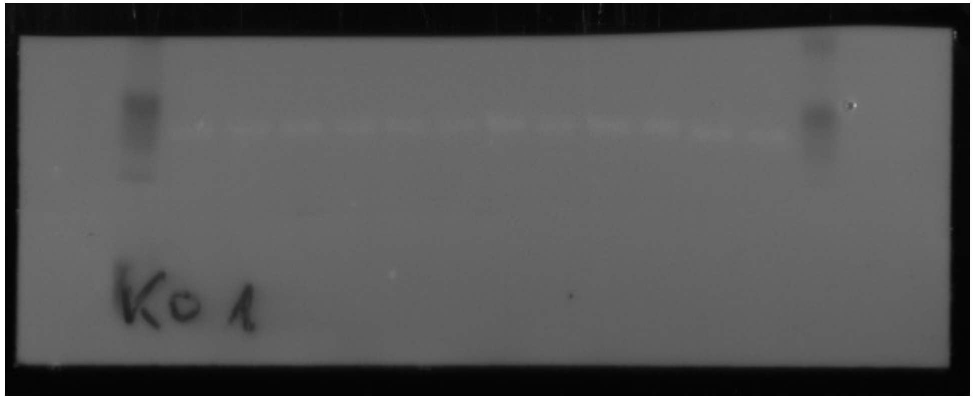

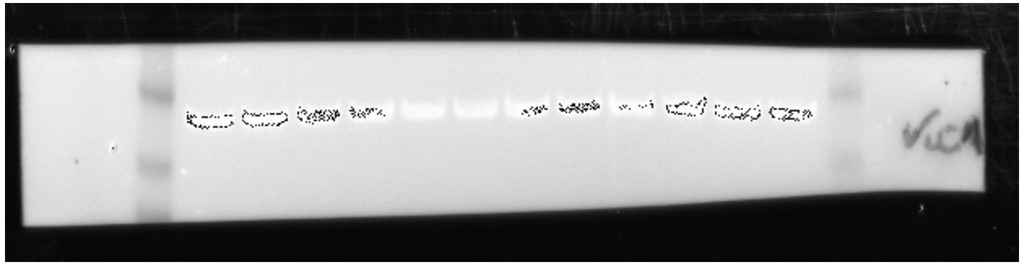

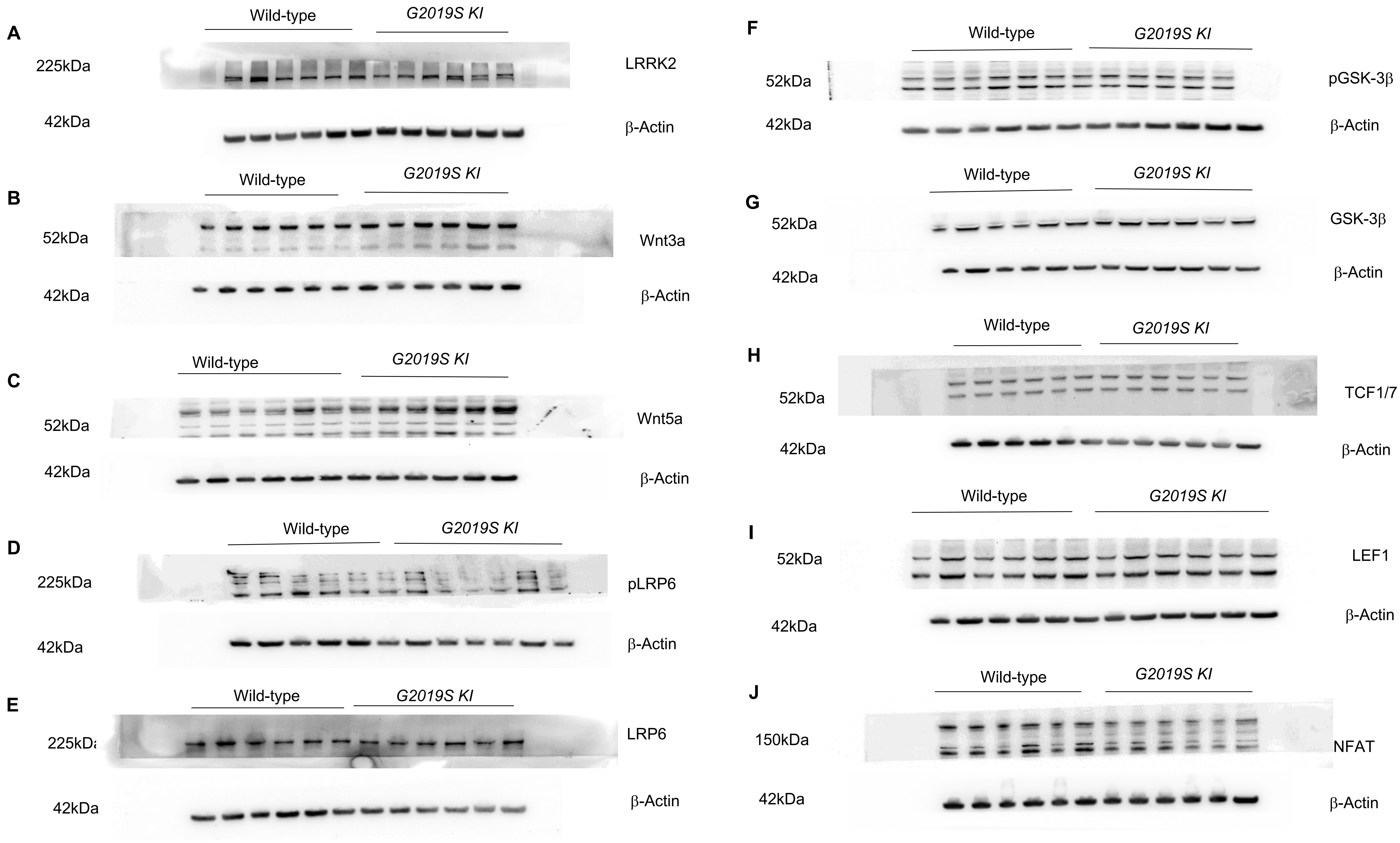

A
Wild-type
G2019S KI
225kDa
LRRK2
42kDa
β-Actin
B
Wild-type
G2019S KI
52kDa
Wnt3a
42kDa
β-Actin
C
Wild-type
G2019S KI
52kDa
Wnt5a
42kDa
β-Actin
D
Wild-type
G2019S KI
225kDa
pLRP6
42kDa
β-Actin
E
Wild-type
G2019S KI
225kDa
LRP6
42kDa
β-Actin
F
Wild-type
G2019S KI
52kDa
pGSK-3β
42kDa
β-Actin
G
Wild-type
G2019S KI
52kDa
GSK-3β
42kDa
β-Actin
H
Wild-type
G2019S KI
52kDa
TCF1/7
42kDa
β-Actin
I
Wild-type
G2019S KI
52kDa
LEF1
42kDa
β-Actin
J
Wild-type
G2019S KI
150kDa
NFAT
42kDa
β-Actin

K

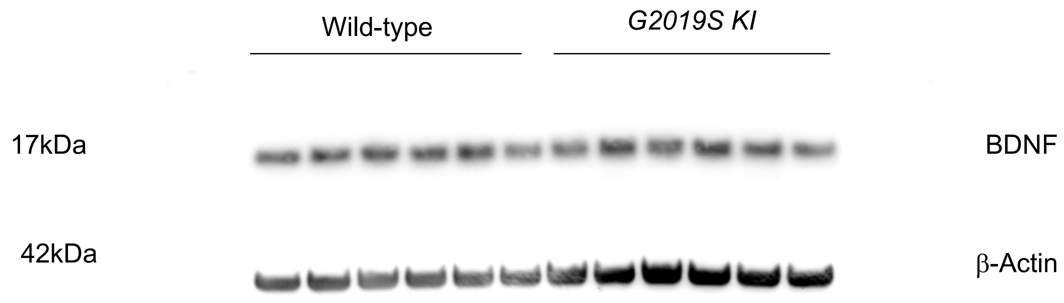

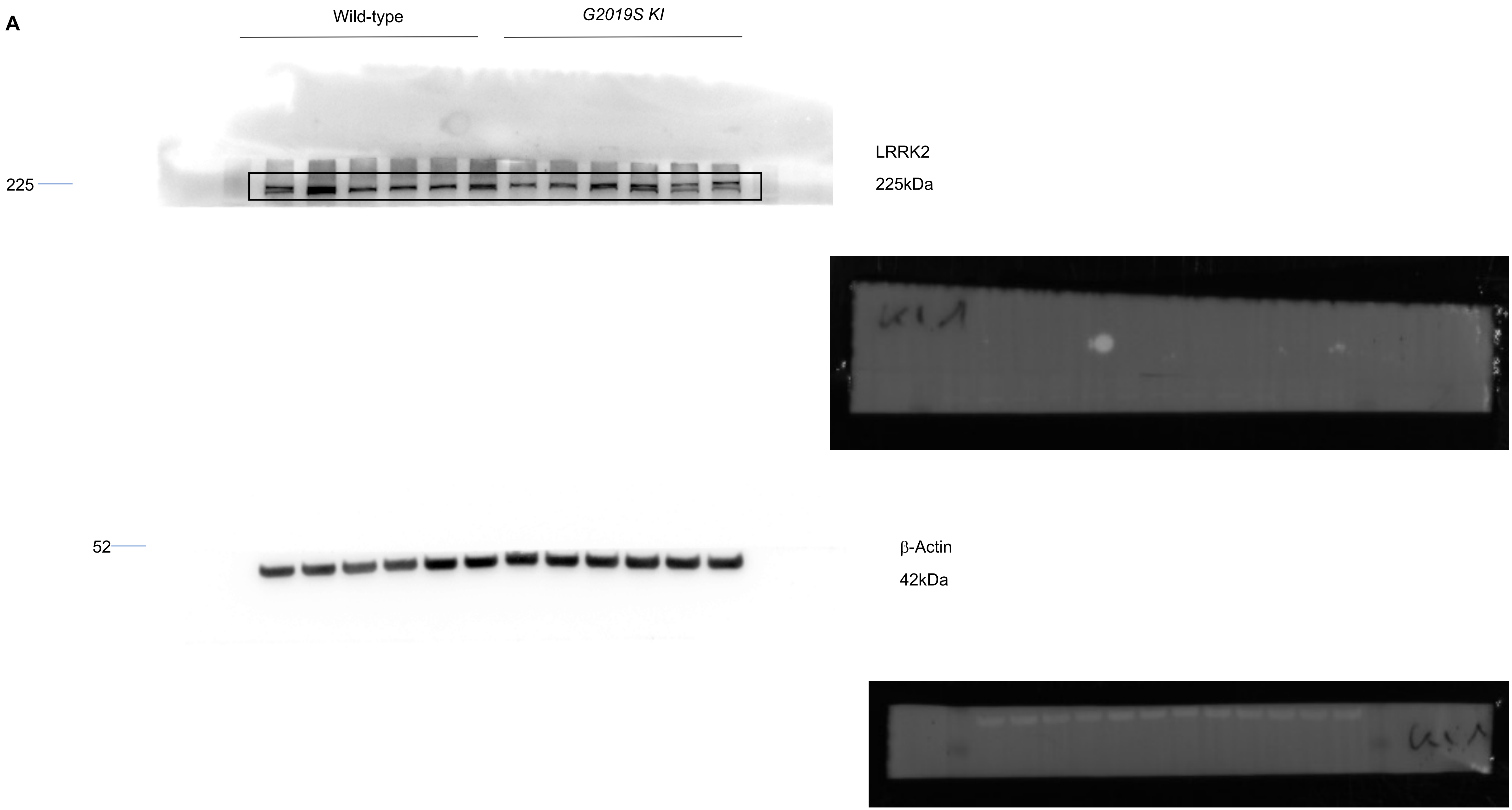
A
Wild-type
G2019S KI
225
LRRK2
225kDa
52
β-Actin
42kDa

B

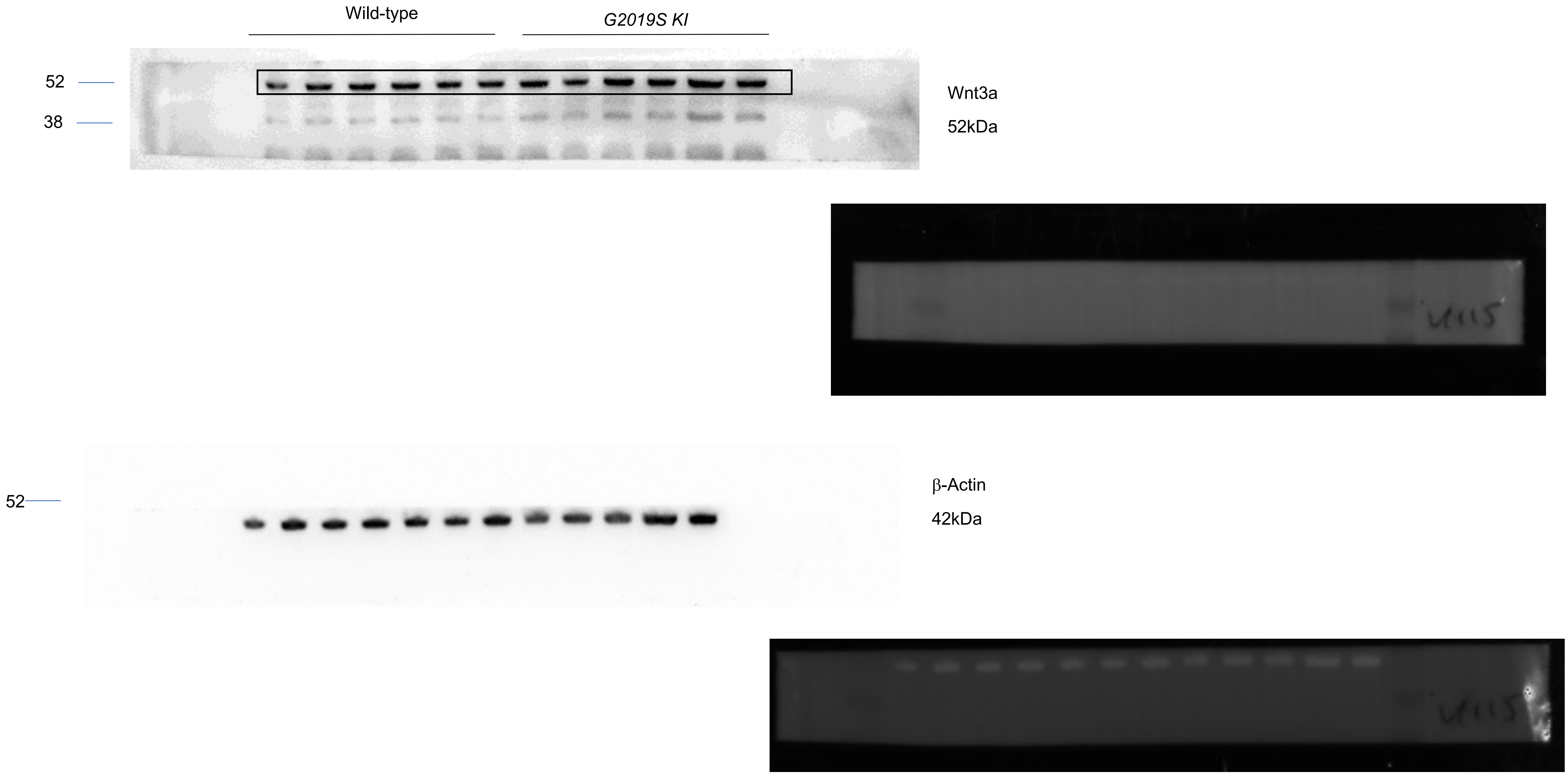

C

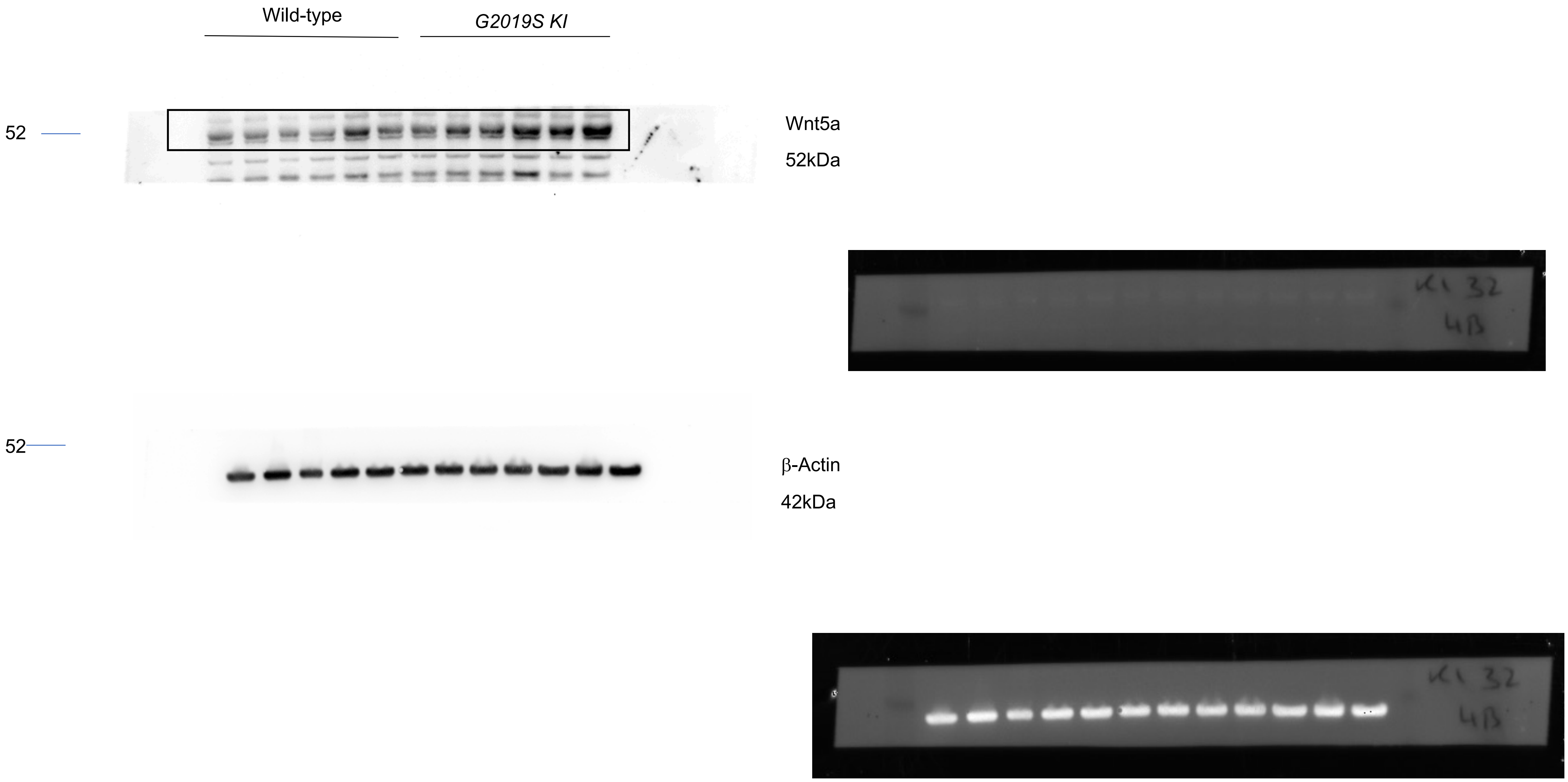

D

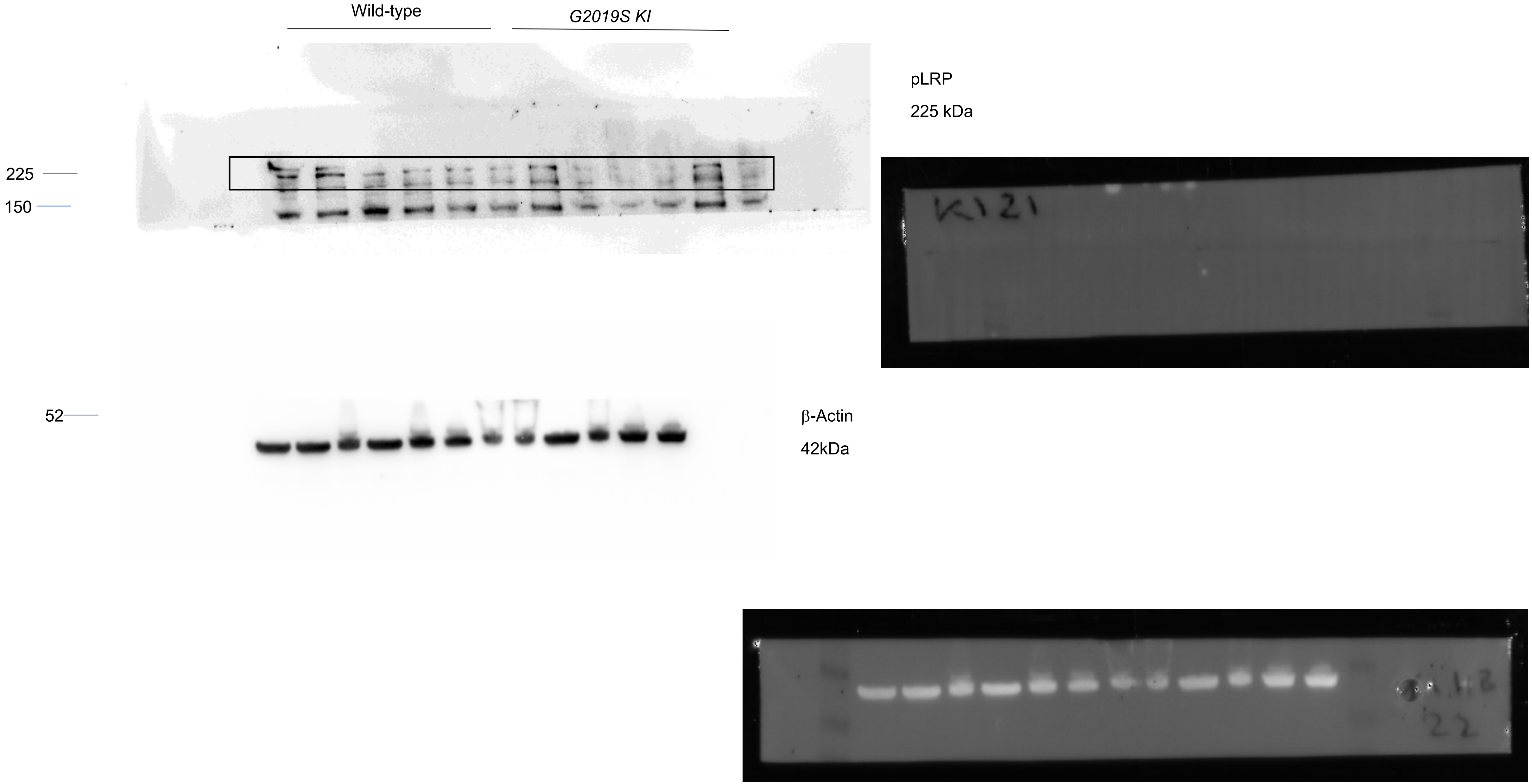

E

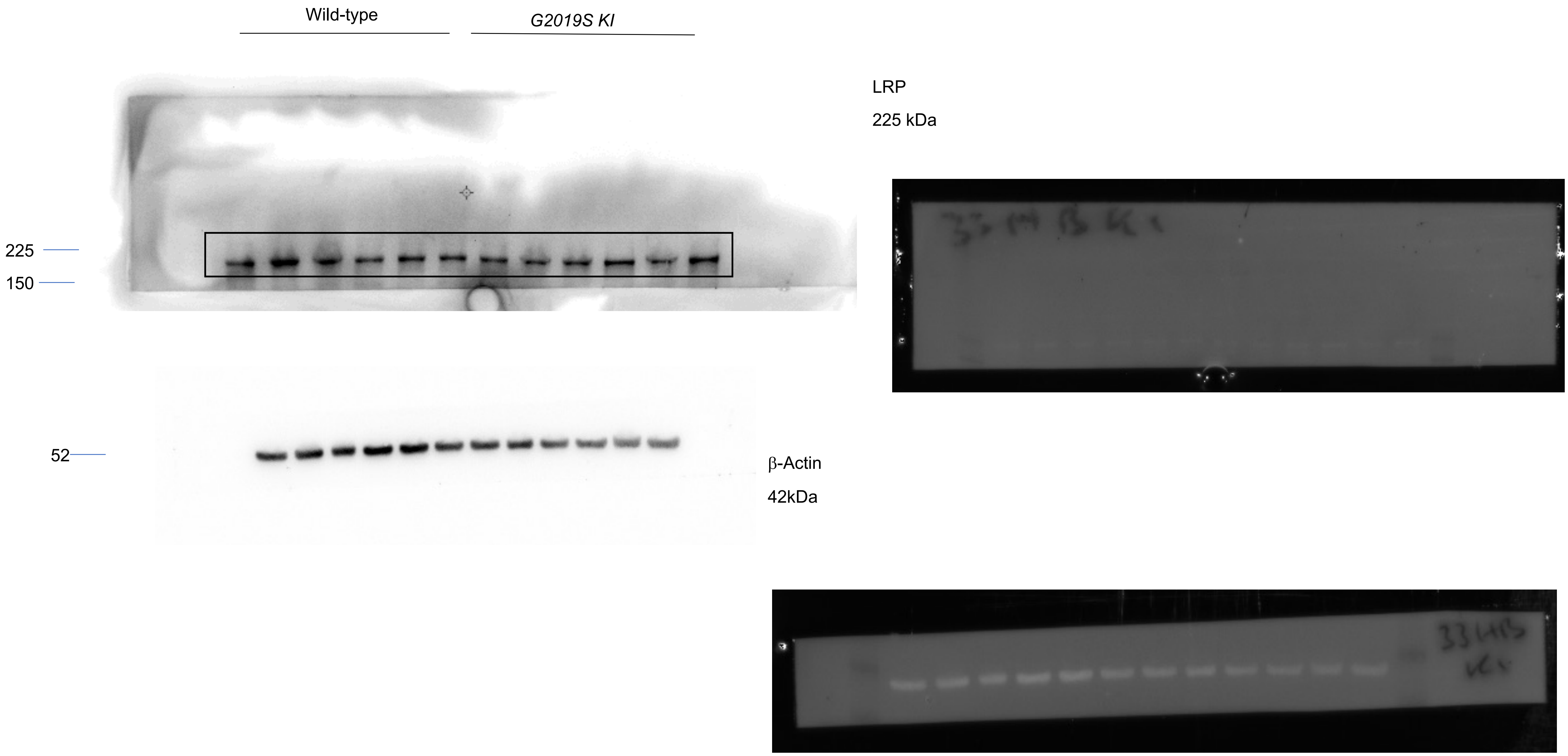

F

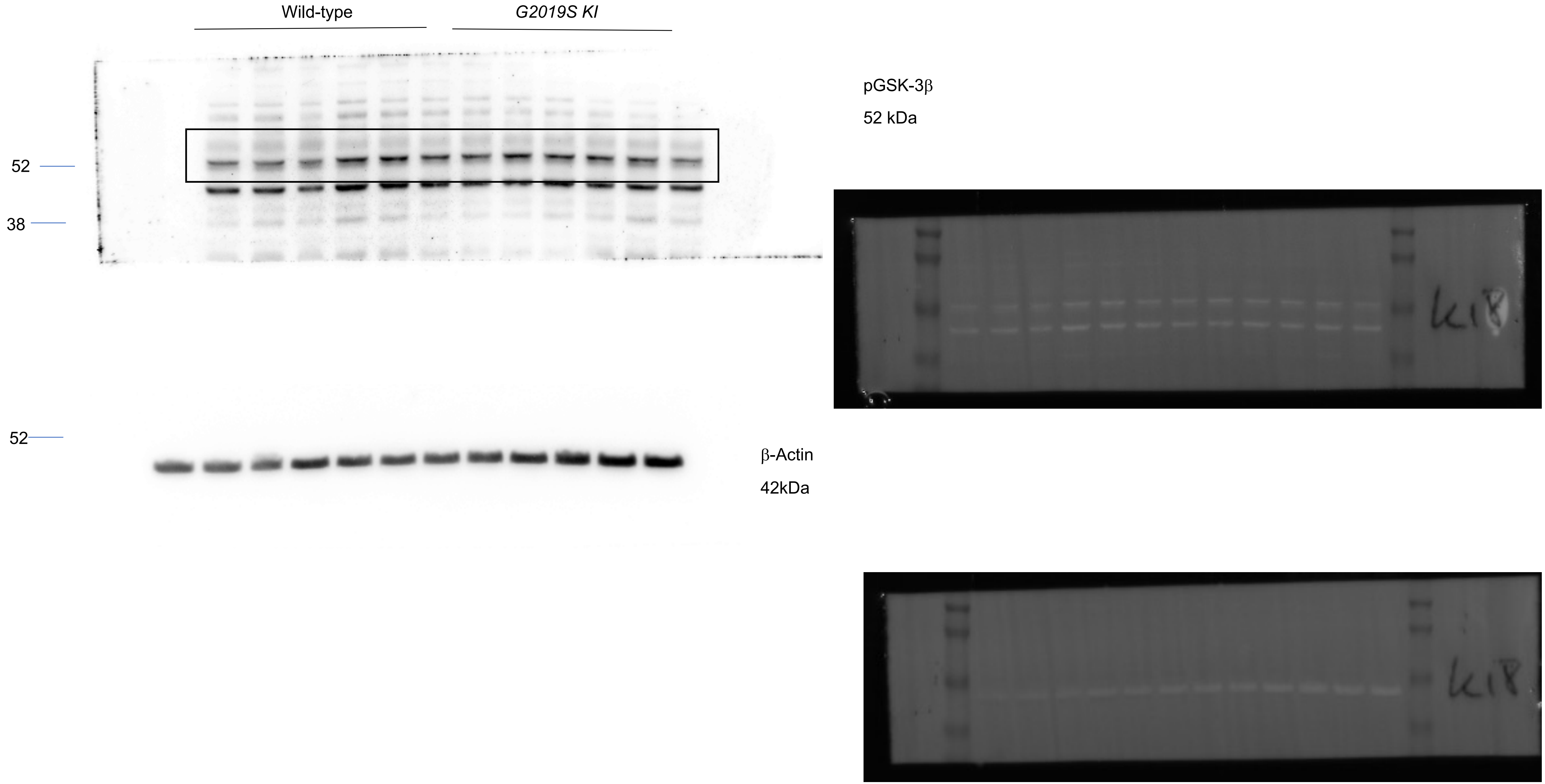

G

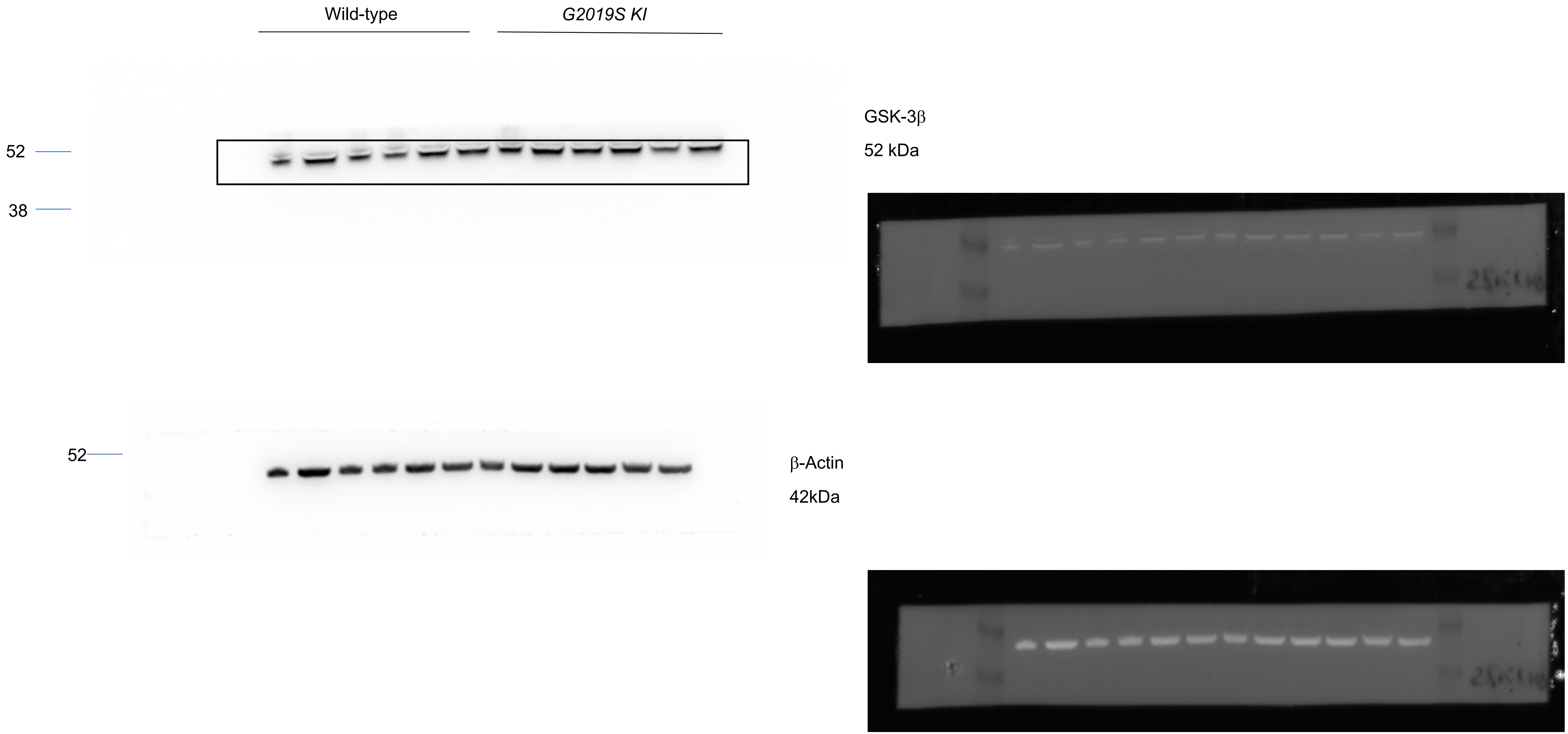

H

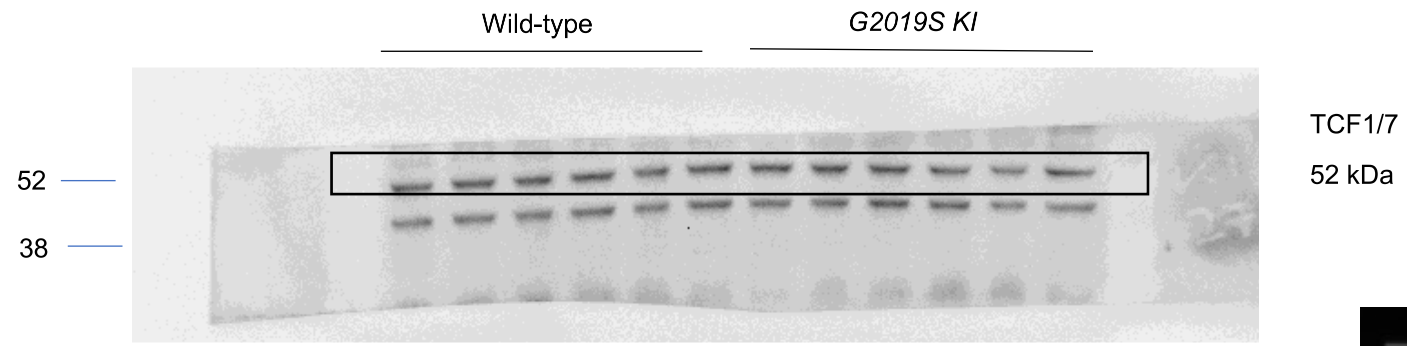

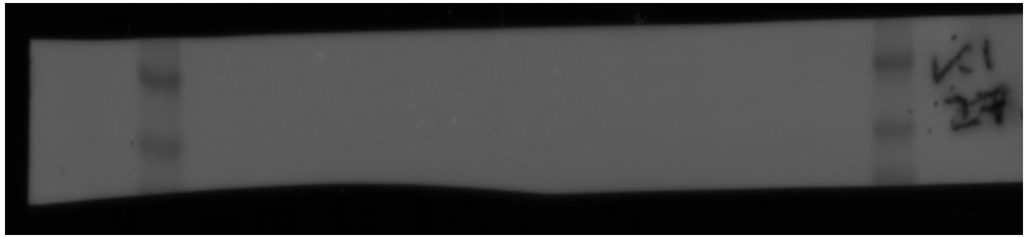

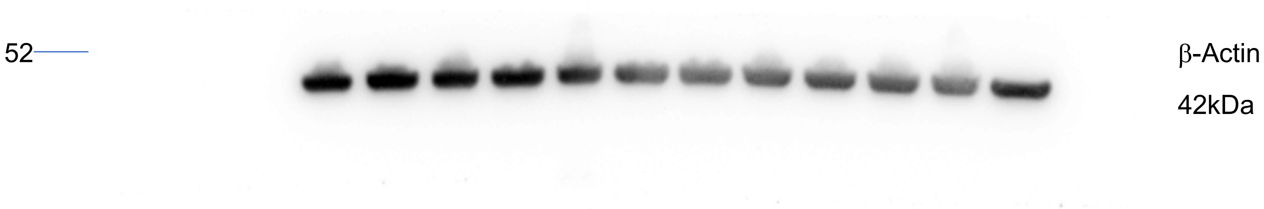

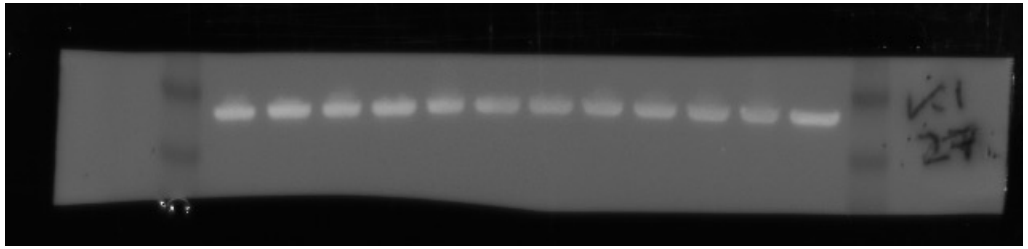

I

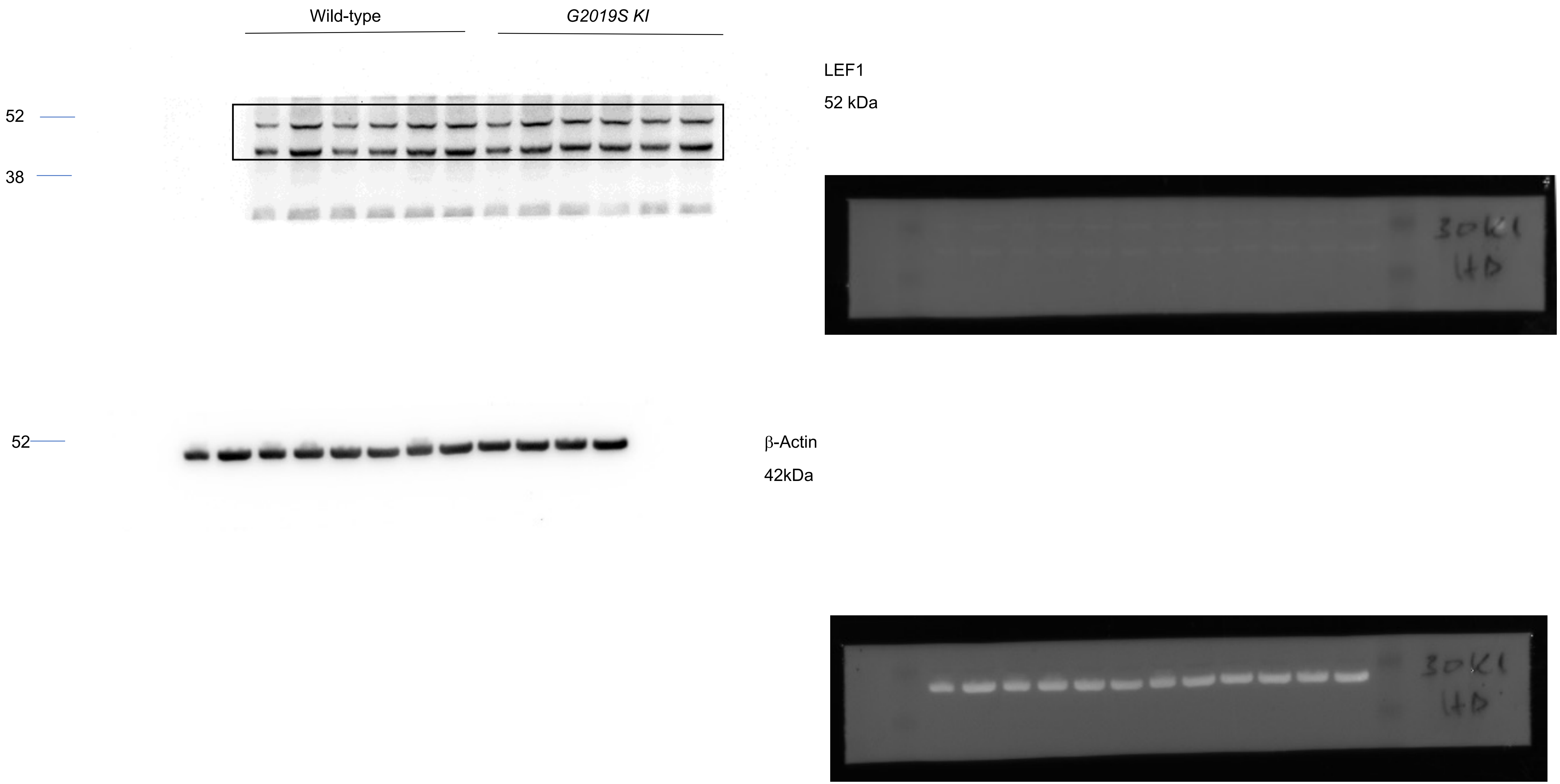

J

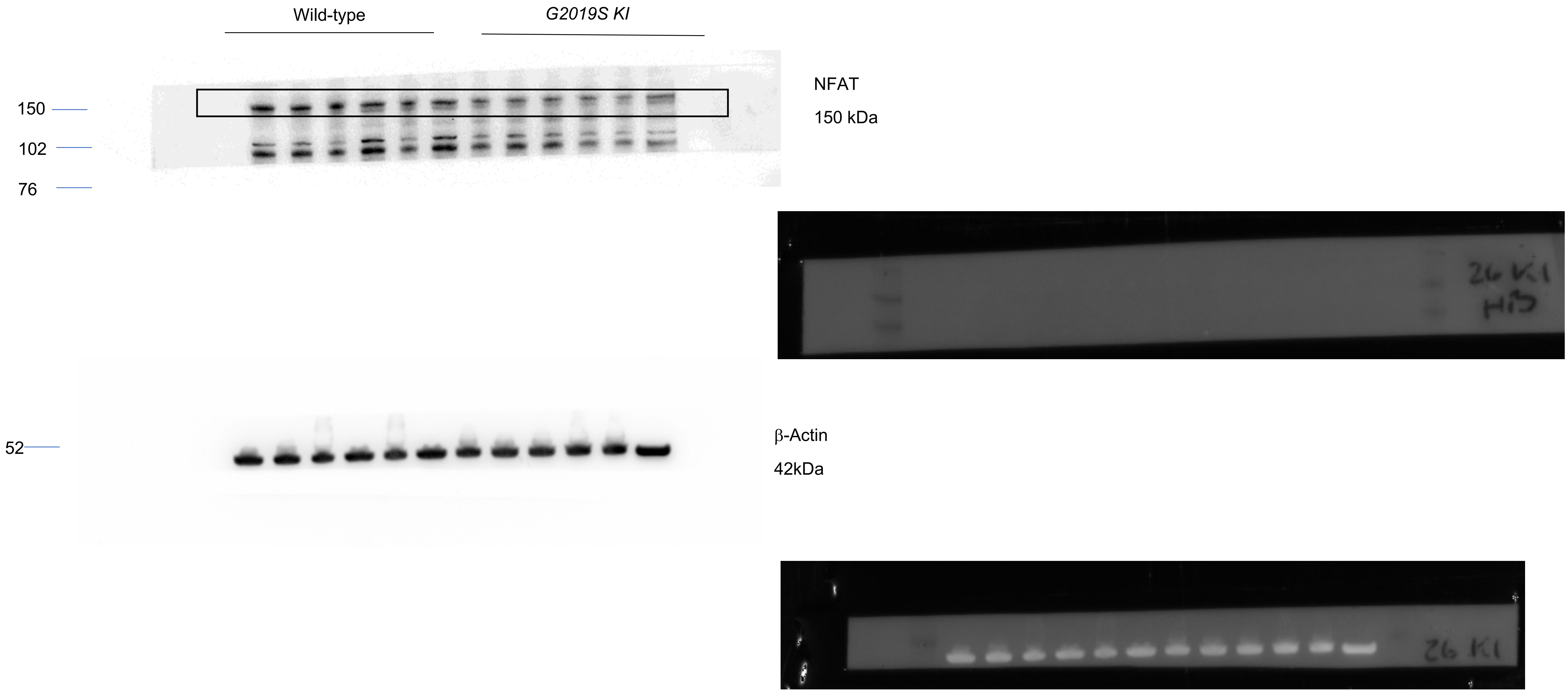

K

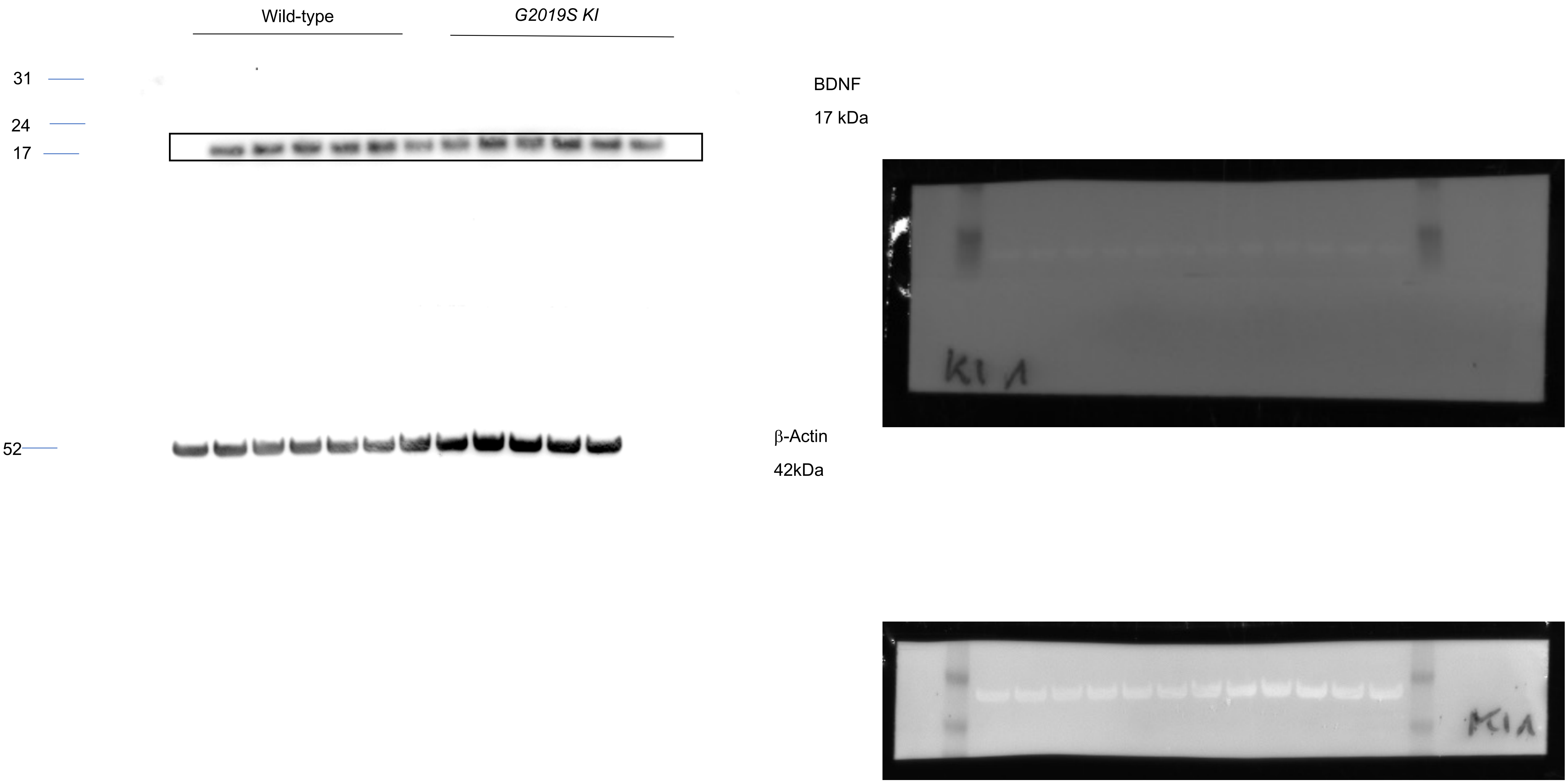

Supplement: Supplementary file 2 — Supplementary Figure 1. [file 41598_2024_63130_MOESM2_ESM.pdf]
