# Supplementary material for: Dysregulated Wnt and NFAT signaling in a Parkinson’s disease LRRK2 G2019S knock-in model
Source: Sci Rep. 2024 May 29;14:12393. doi: 10.1038/s41598-024-63130-8 (PMC11137013; doi:10.1038/s41598-024-63130-8)

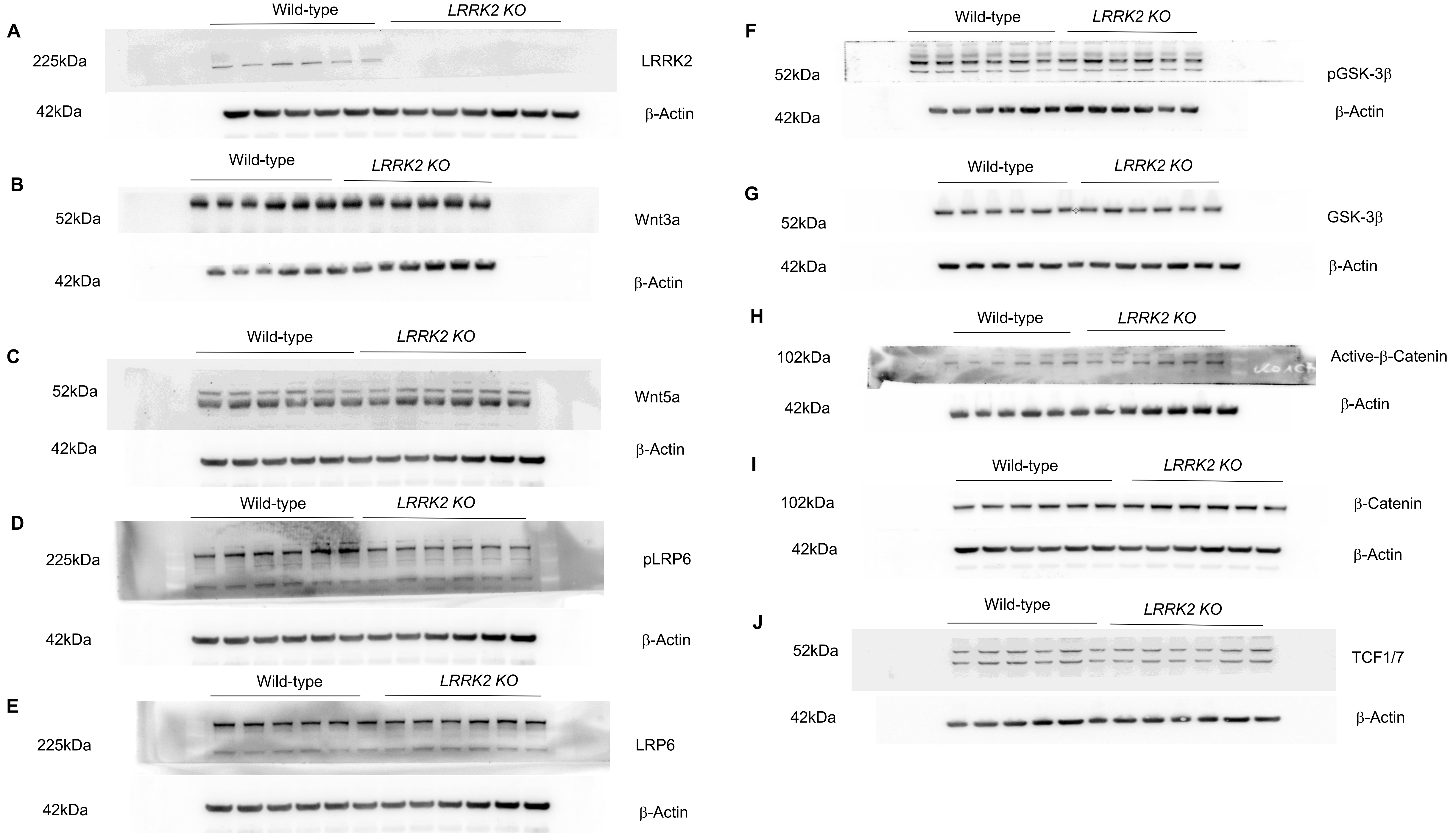
A
Wild-type
LRRK2 KO
225kDa
LRRK2
42kDa
β-Actin
B
52kDa
Wnt3a
C
Wnt5a
D
pLRP6
E
LRP6
F
pGSK-3β
G
GSK-3β
H
102kDa
Active-β-Catenin
I
β-Catenin
J
TCF1/7

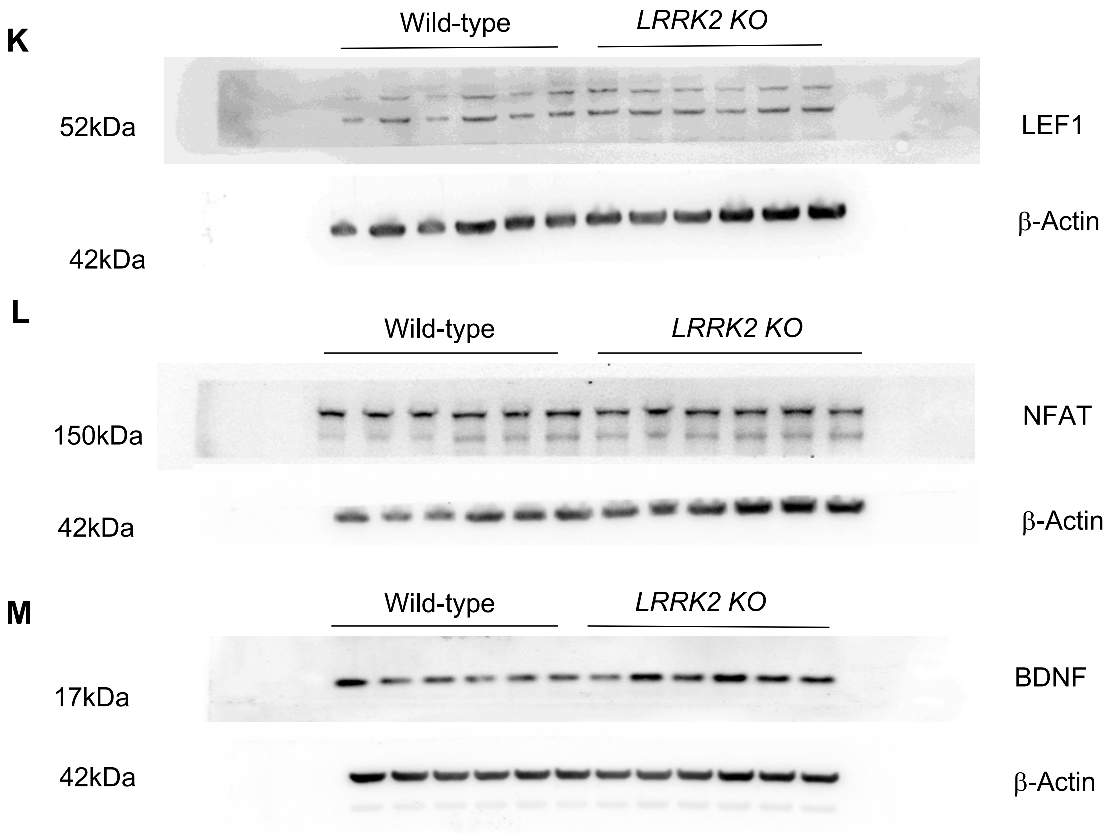
K
Wild-type
LRRK2 KO
52kDa
LEF1
42kDa
β-Actin
L
Wild-type
LRRK2 KO
150kDa
NFAT
42kDa
β-Actin
M
Wild-type
LRRK2 KO
17kDa
BDNF
42kDa
β-Actin

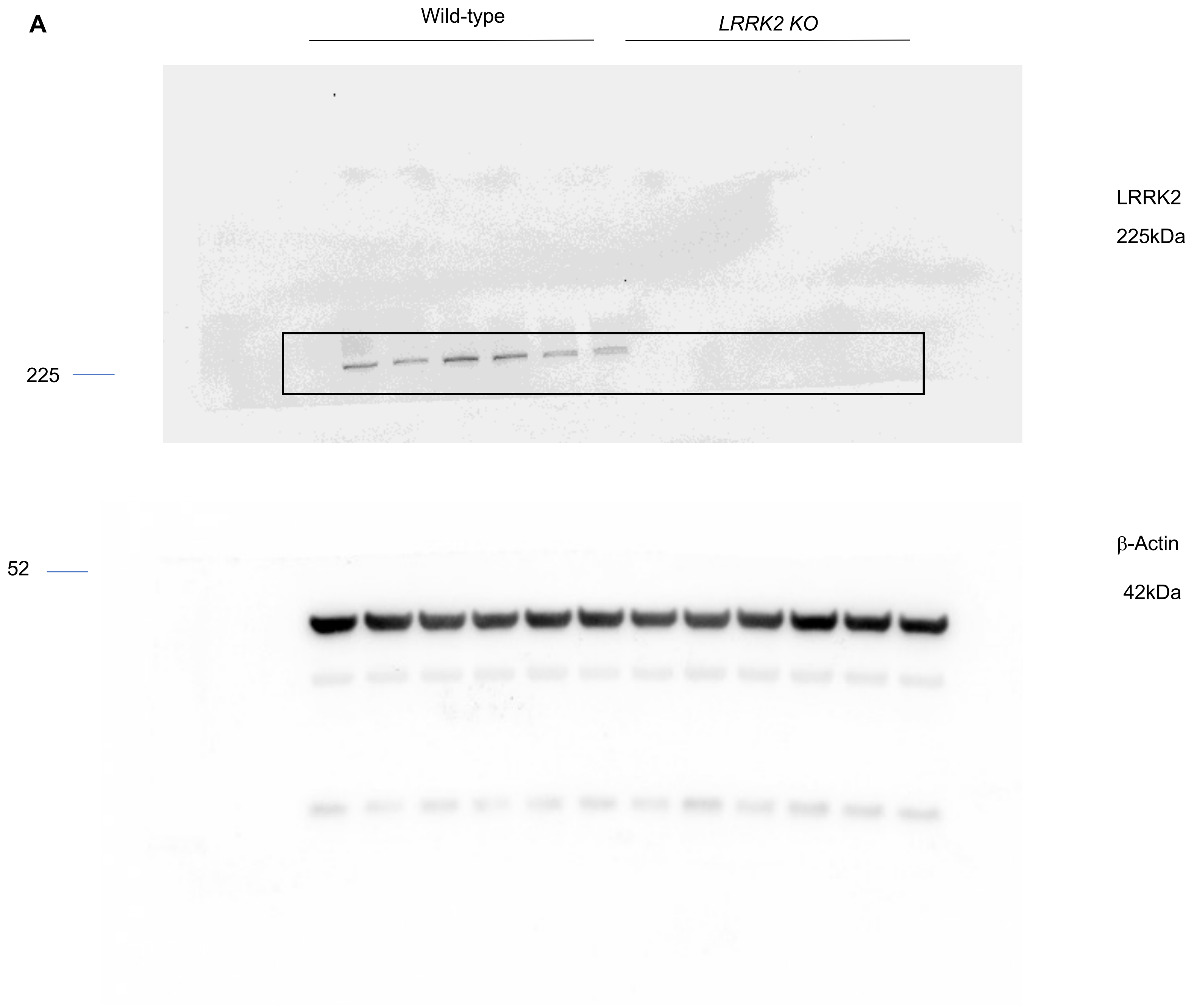
A
Wild-type
LRRK2 KO
LRRK2
225kDa
225
β-Actin
42kDa
52

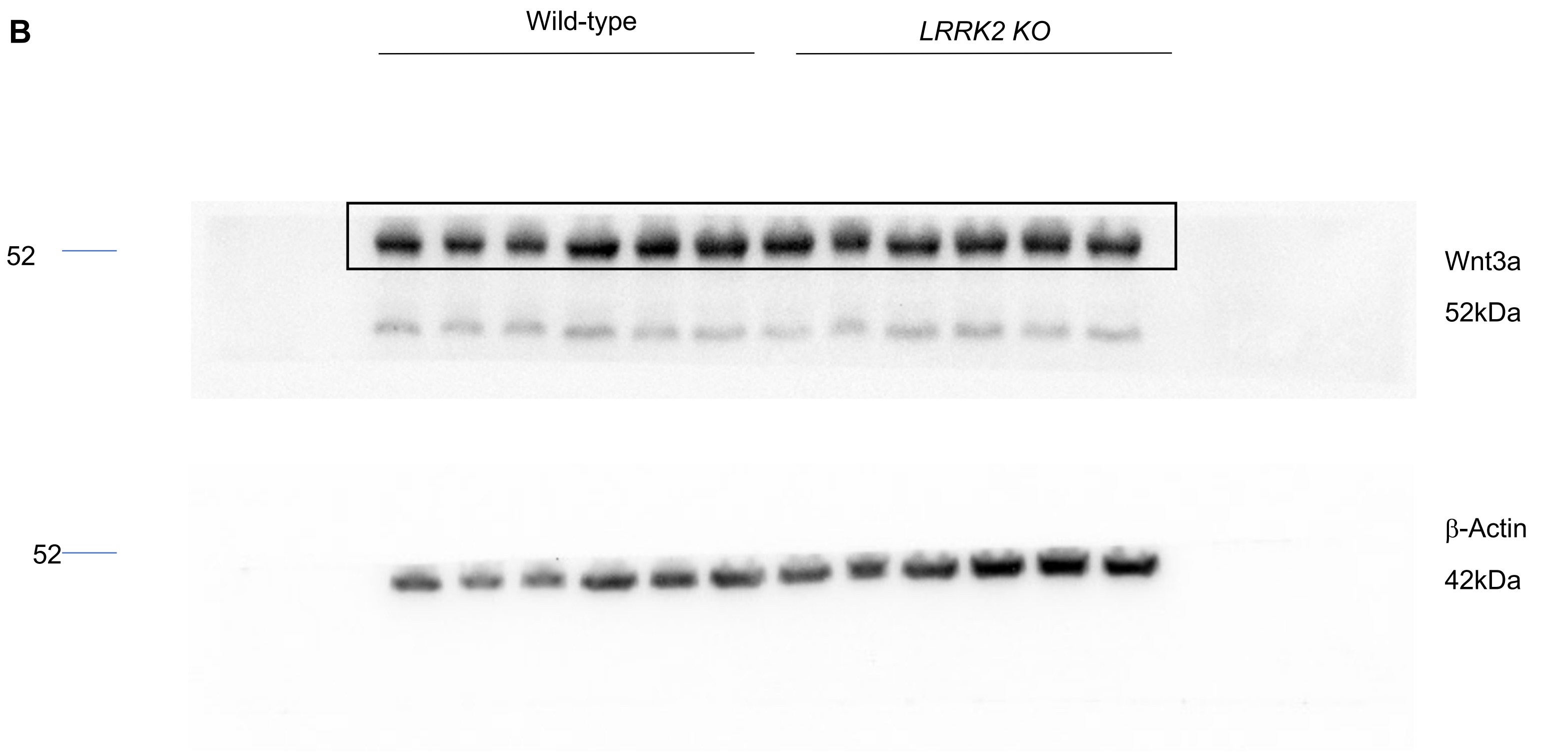
B
Wild-type
LRRK2 KO
52
Wnt3a
52kDa
52
β-Actin
42kDa

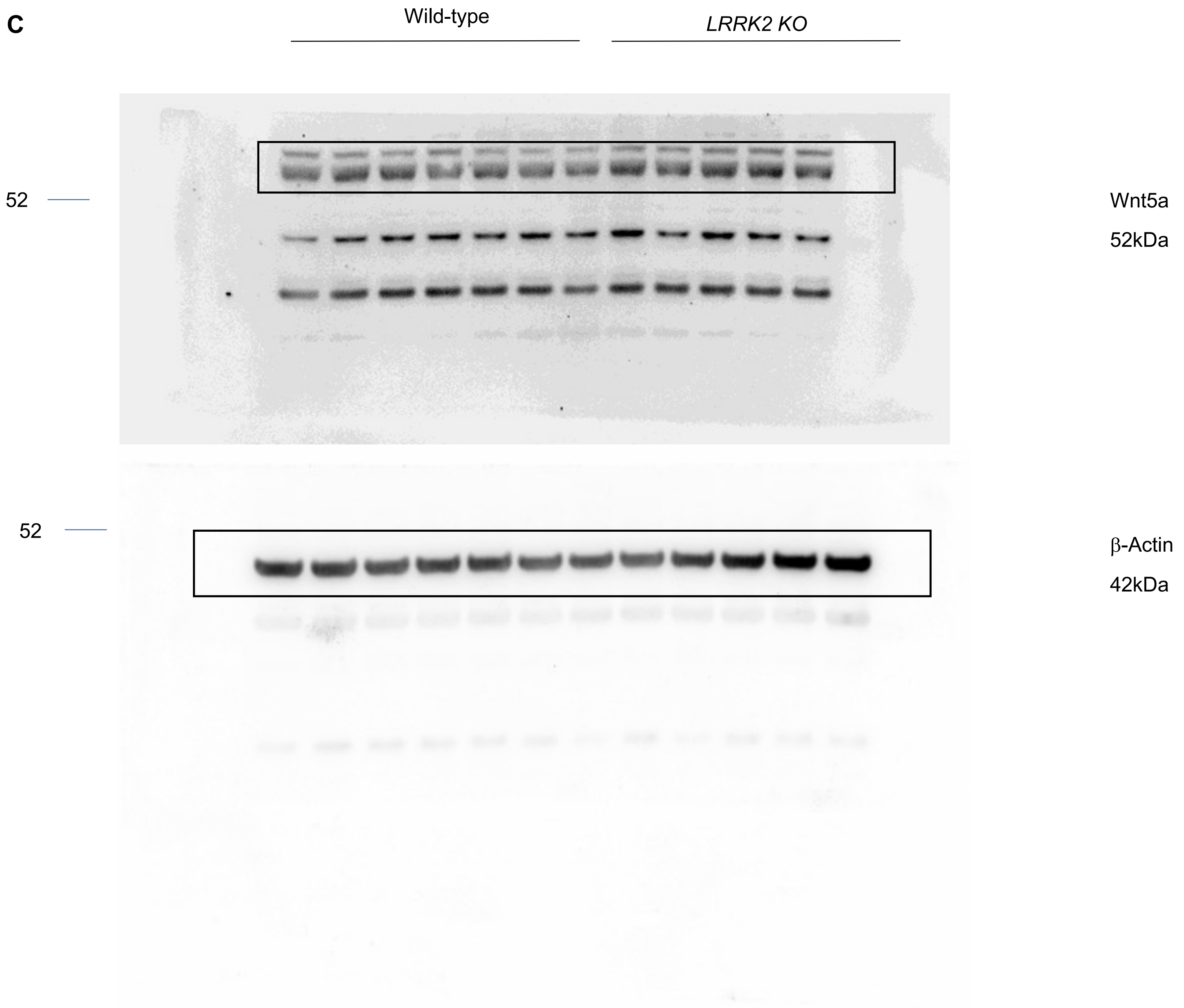
C
Wild-type
LRRK2 KO
52
Wnt5a
52kDa
52
β-Actin
42kDa

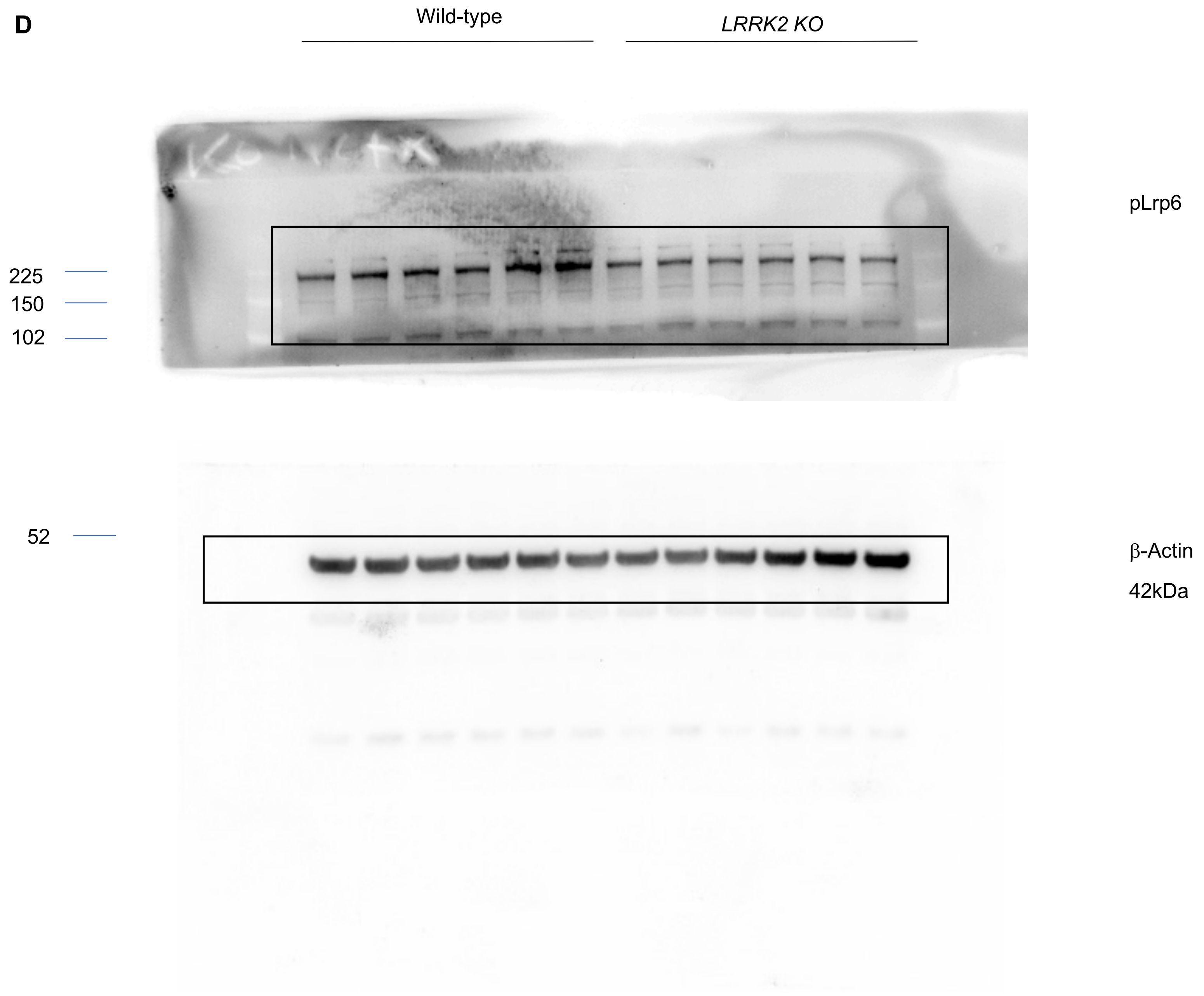
D
Wild-type
LRRK2 KO
pLrp6
225
150
102
52
β-Actin
42kDa

E

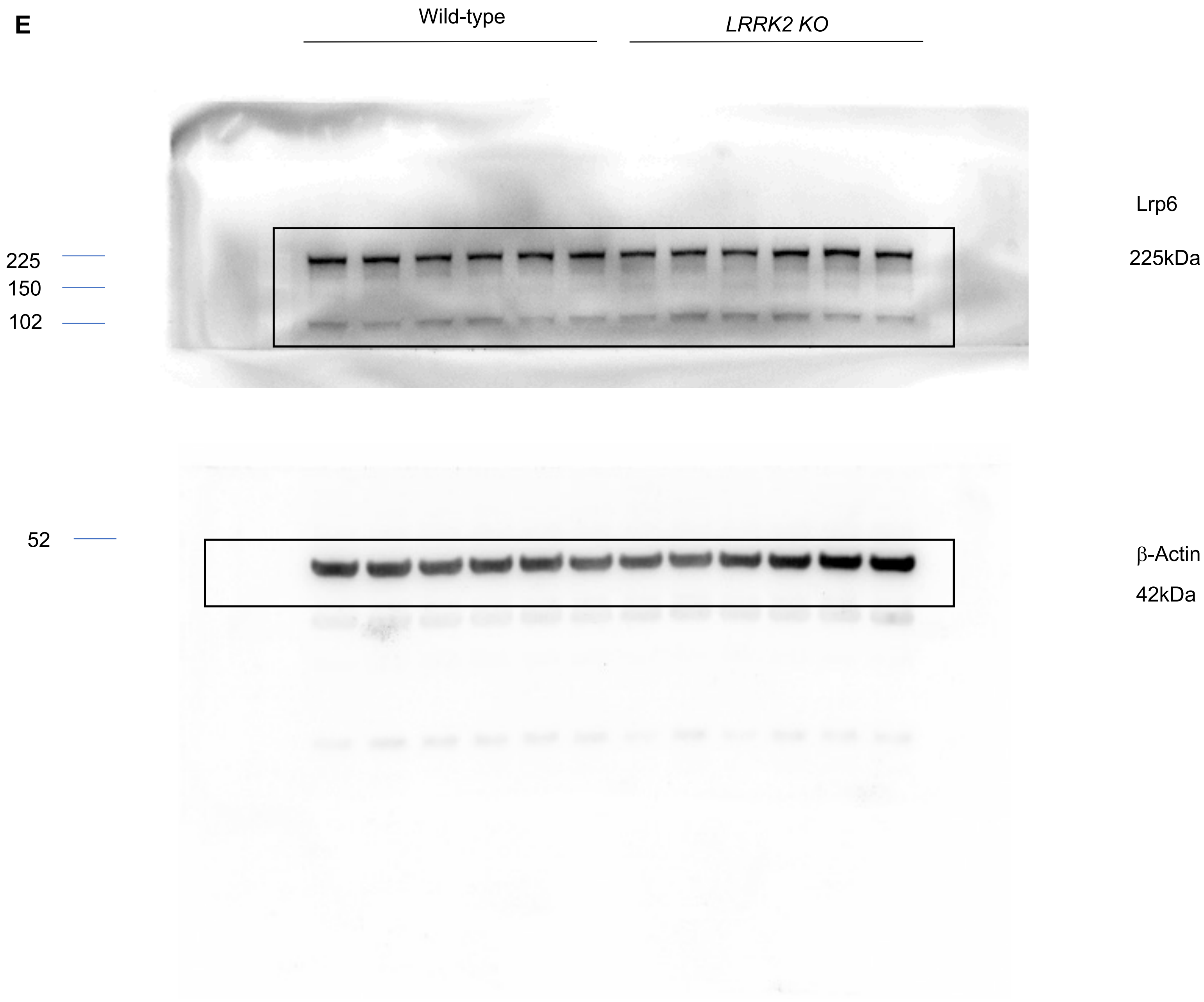

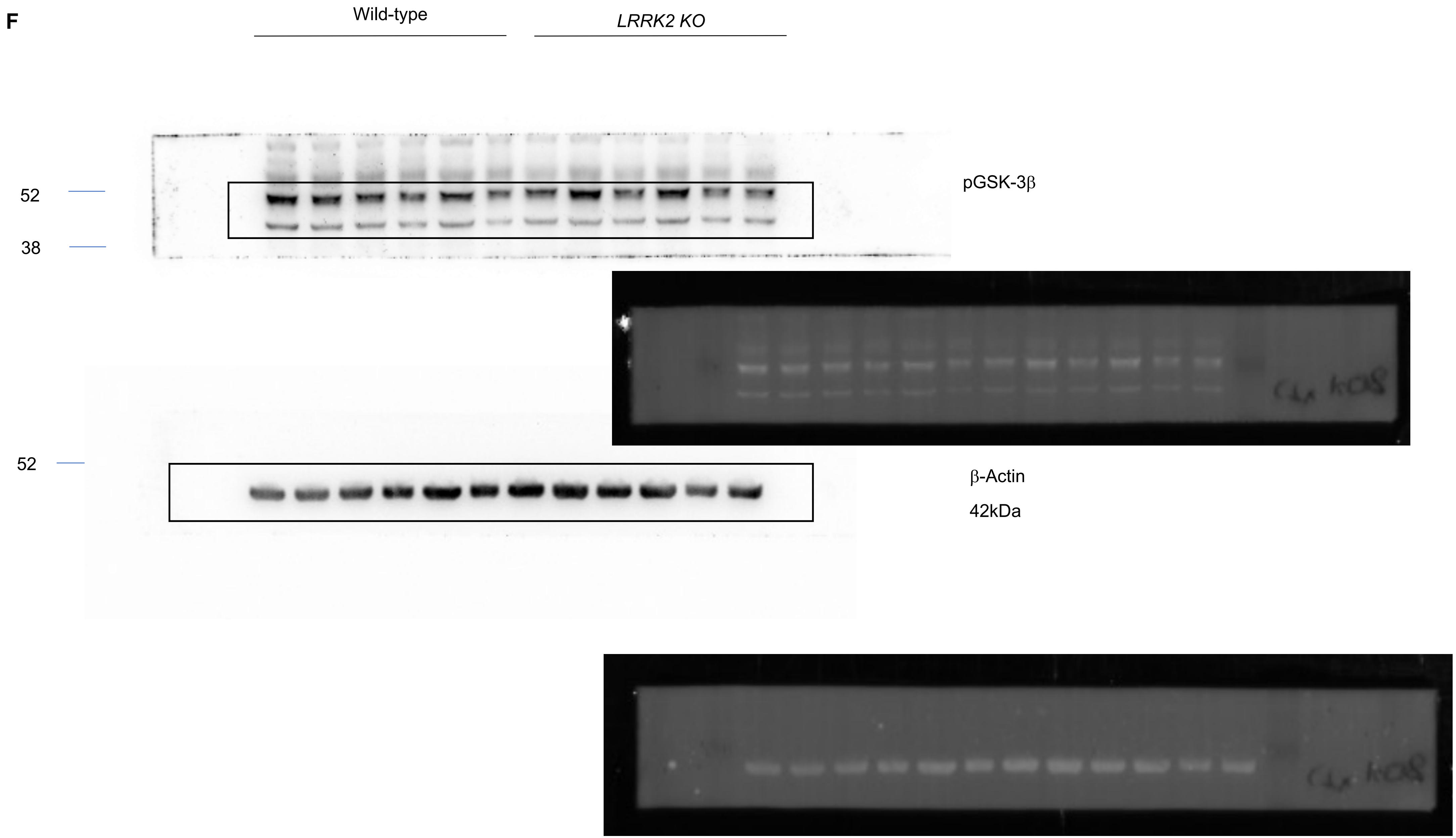
F
Wild-type
LRRK2 KO
52
38
pGSK-3β
52
β-Actin
42kDa

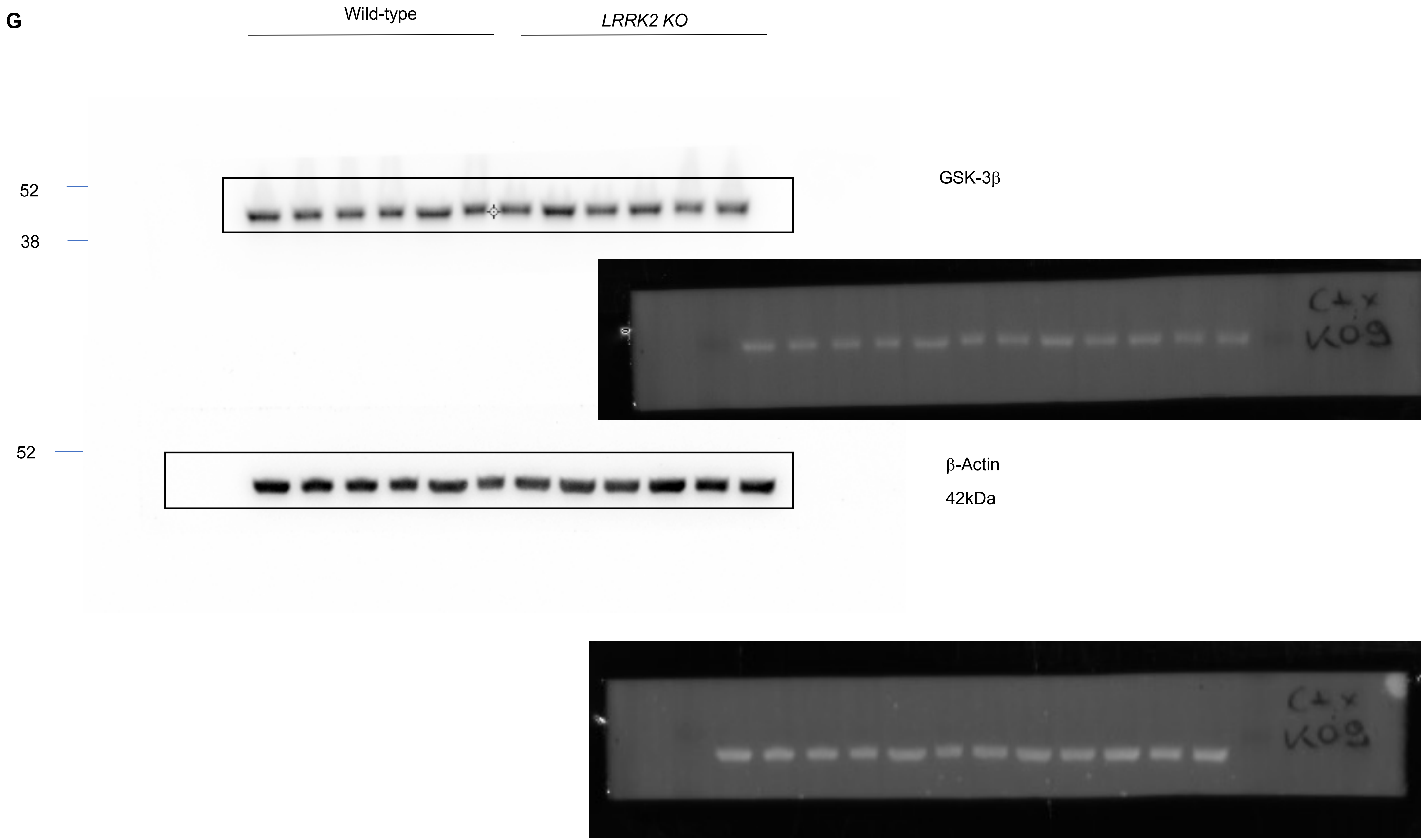
G
Wild-type
LRRK2 KO
52
38
GSK-3β
52
β-Actin
42kDa

H

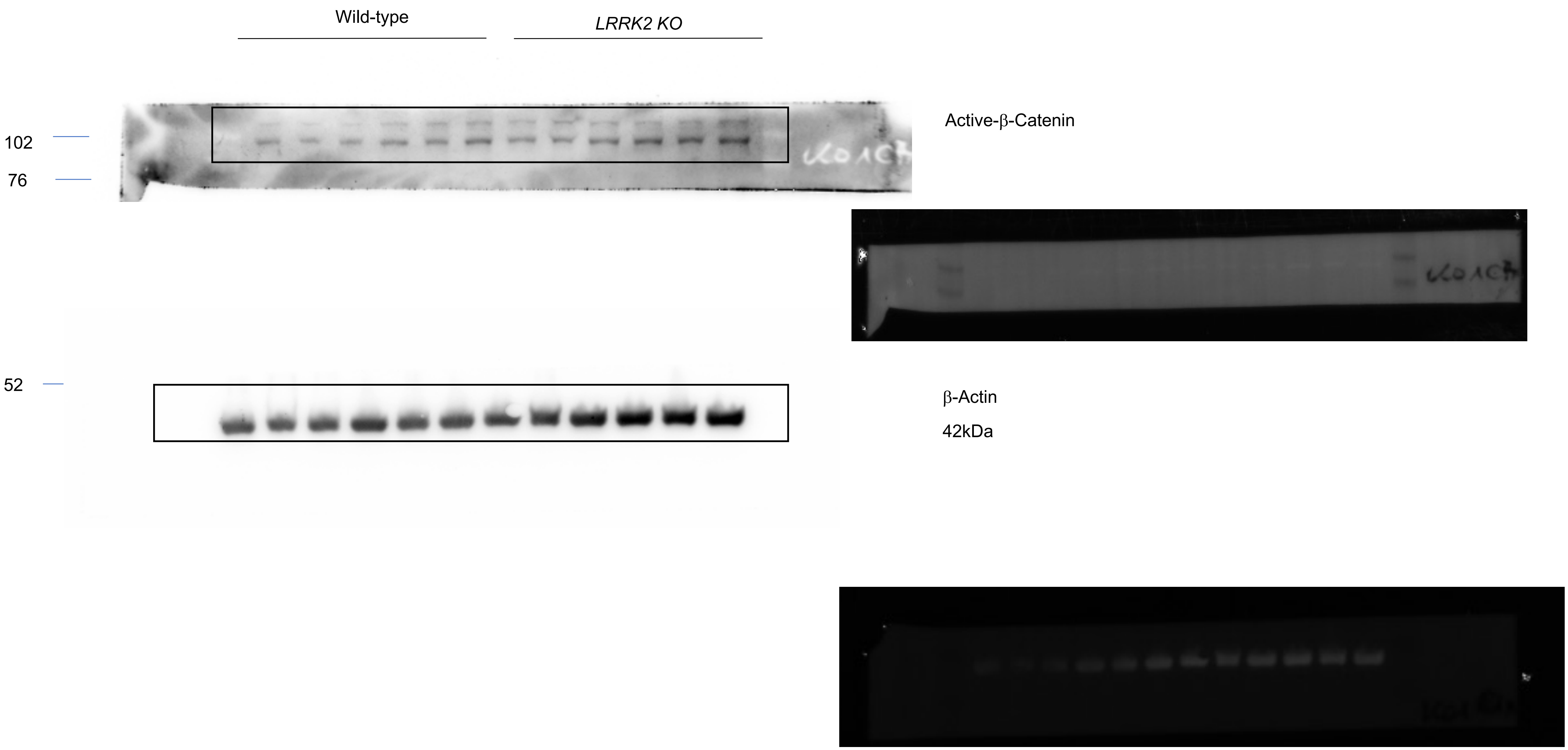

I

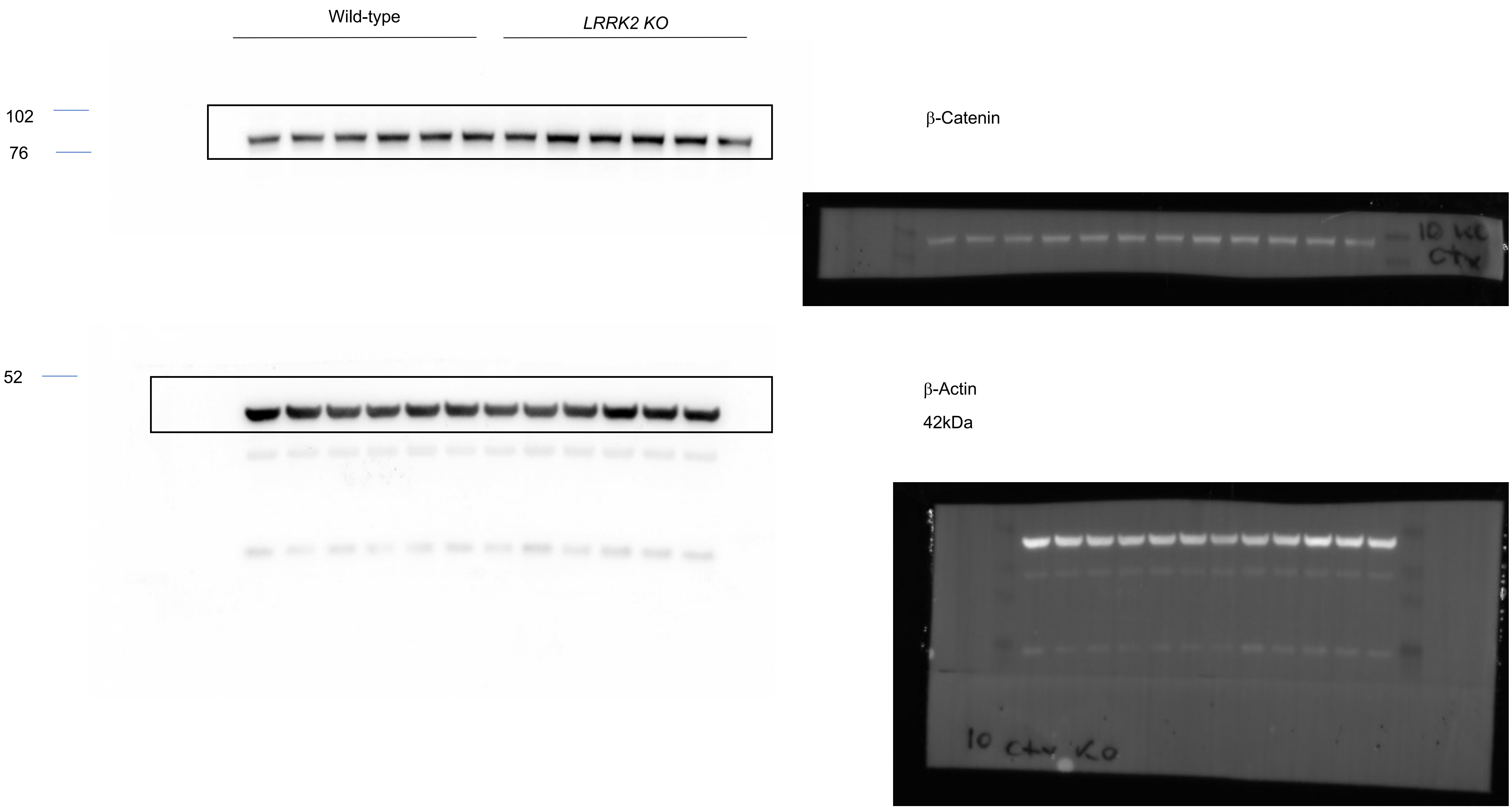

J

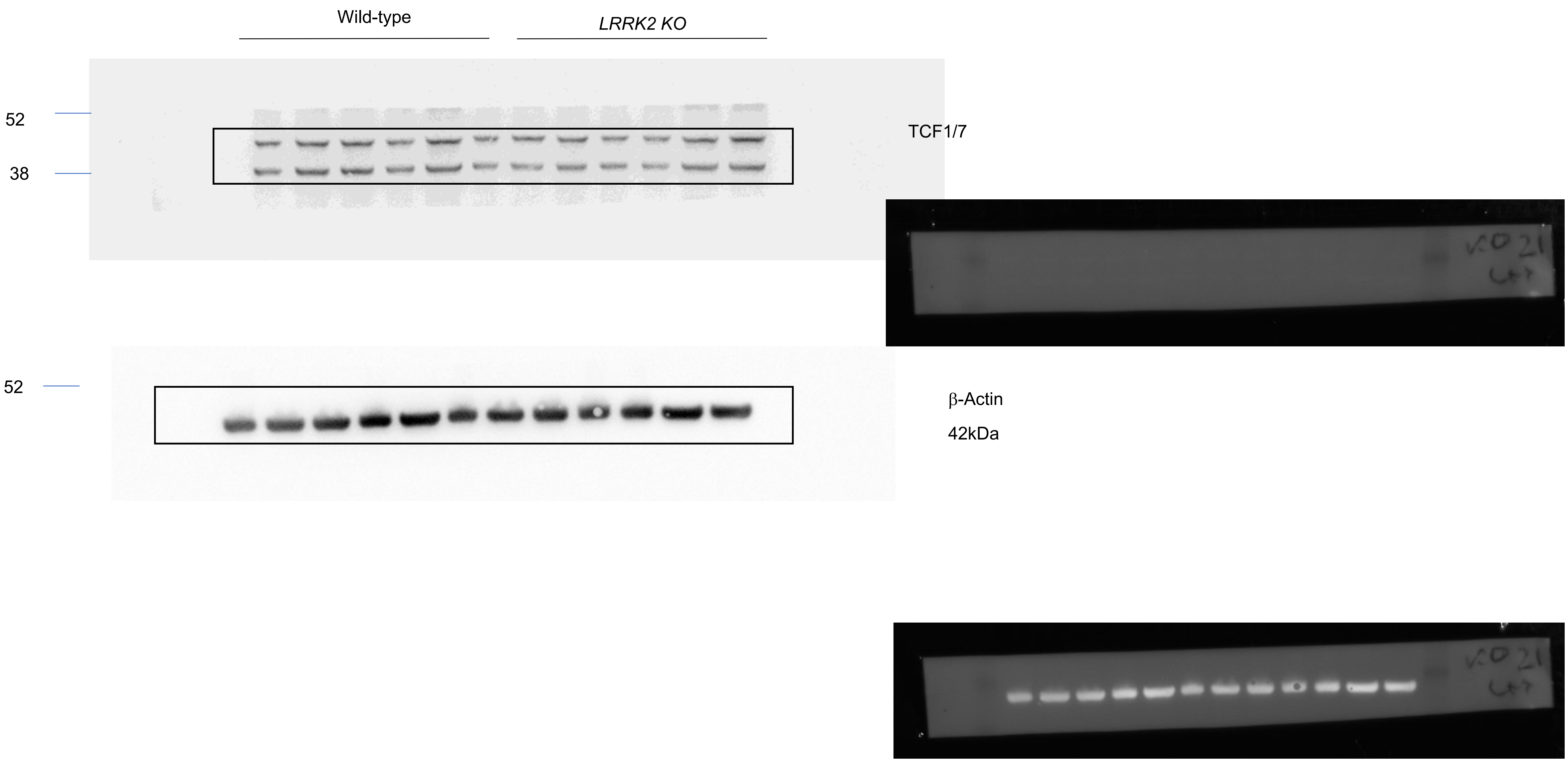

K

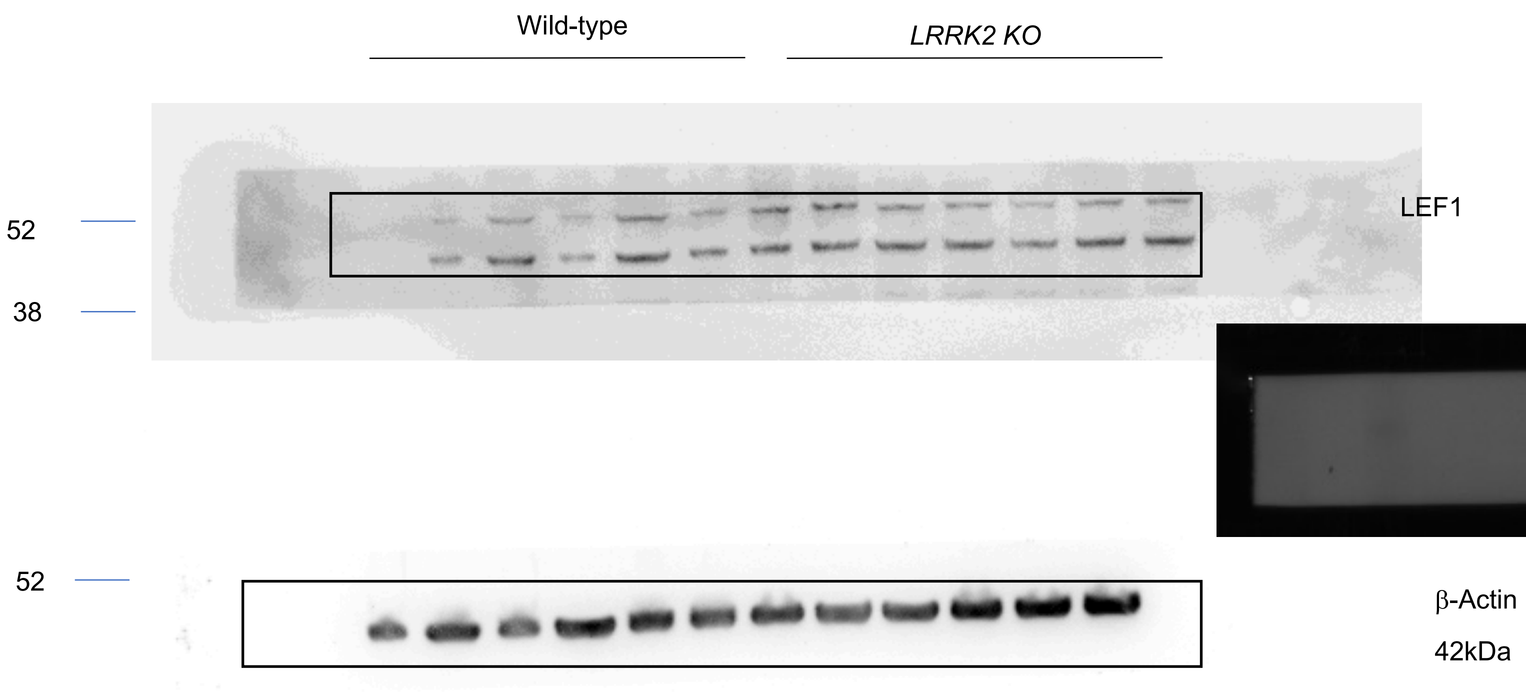

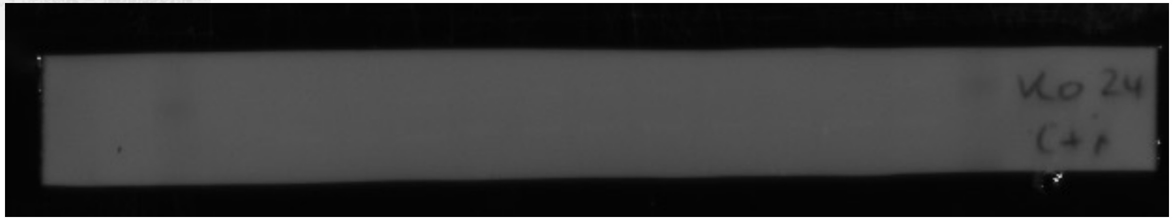

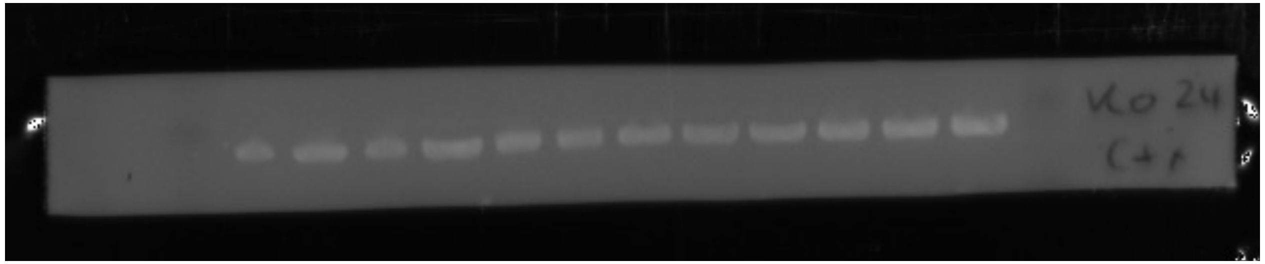

L

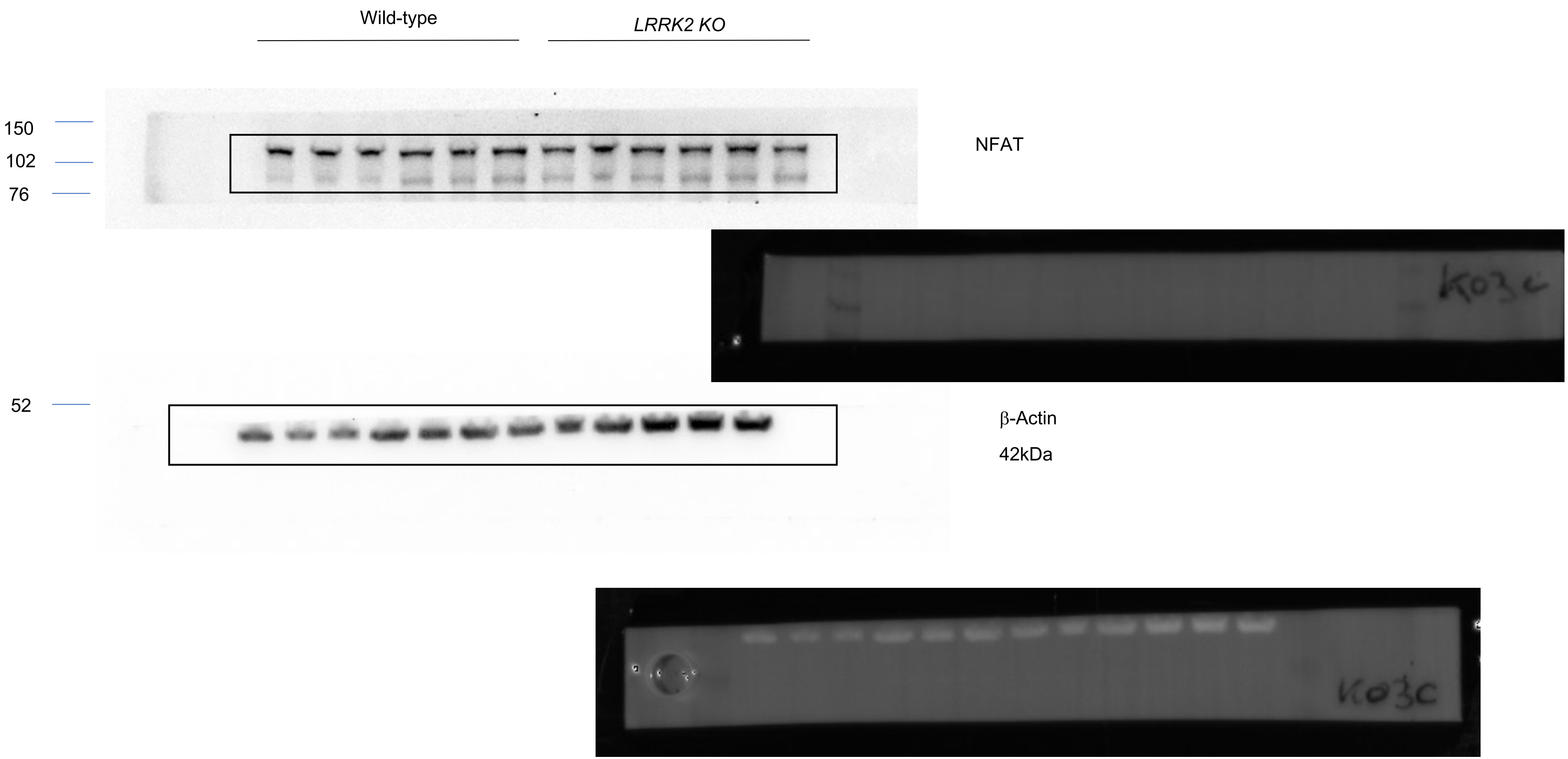

M

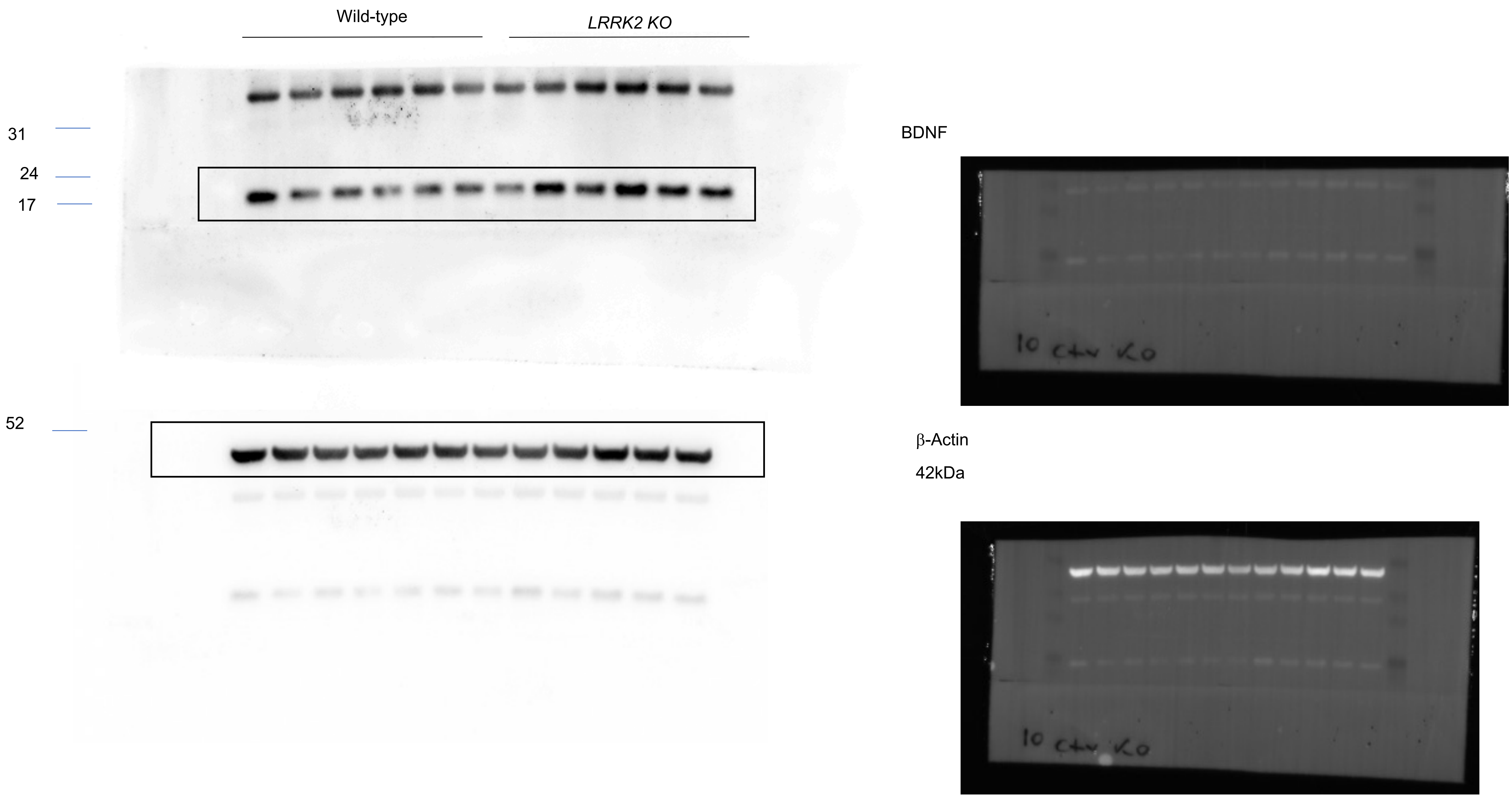
Wild-type
LRRK2 KO
31
24
17
52
BDNF
β-Actin
42kDa
10 ctr KO
10 ctr KO

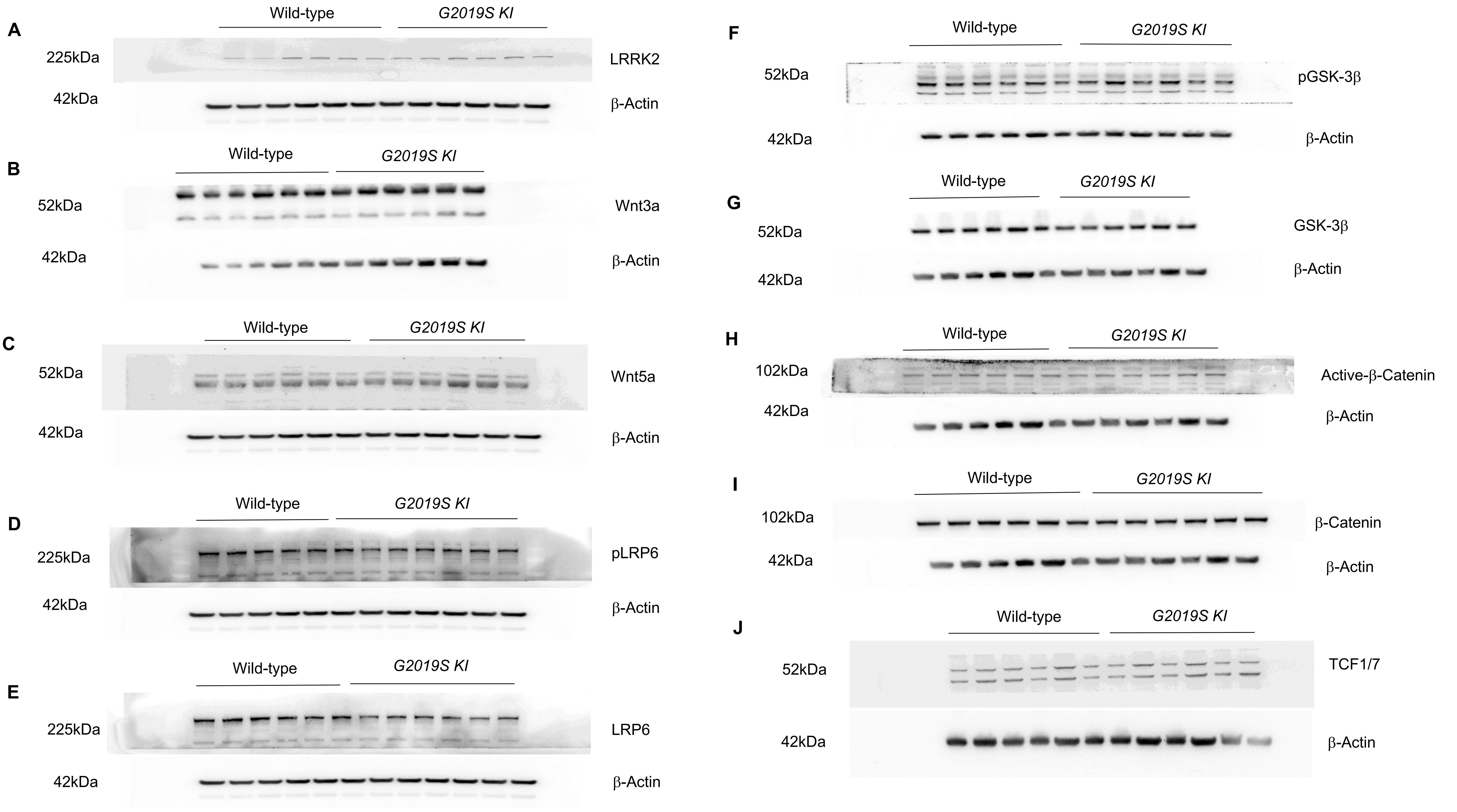

A
Wild-type
G2019S KI
225kDa
LRRK2
42kDa
β-Actin
B
Wild-type
G2019S KI
52kDa
Wnt3a
42kDa
β-Actin
C
Wild-type
G2019S KI
52kDa
Wnt5a
42kDa
β-Actin
D
Wild-type
G2019S KI
225kDa
pLRP6
42kDa
β-Actin
E
Wild-type
G2019S KI
225kDa
LRP6
42kDa
β-Actin
F
Wild-type
G2019S KI
52kDa
pGSK-3β
42kDa
β-Actin
G
Wild-type
G2019S KI
52kDa
GSK-3β
42kDa
β-Actin
H
Wild-type
G2019S KI
102kDa
Active-β-Catenin
42kDa
β-Actin
I
Wild-type
G2019S KI
102kDa
β-Catenin
42kDa
β-Actin
J
Wild-type
G2019S KI
52kDa
TCF1/7
42kDa
β-Actin

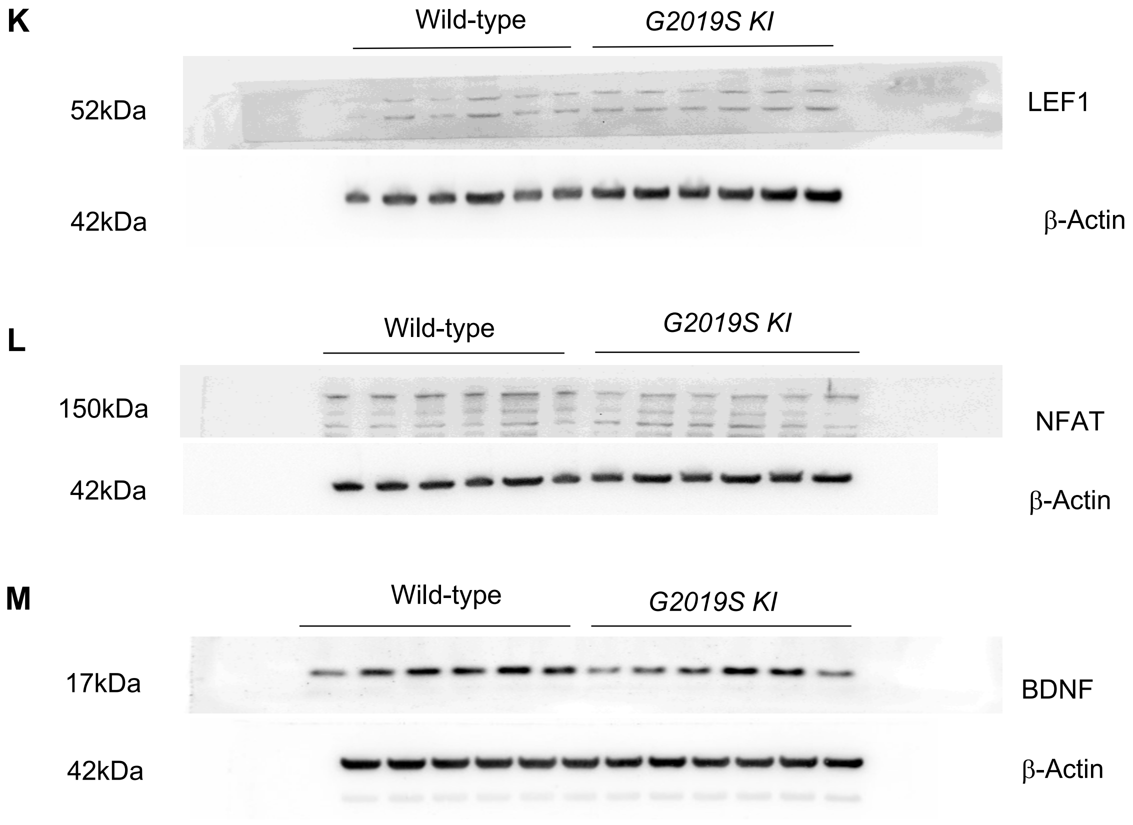
K
Wild-type
G2019S KI
52kDa
LEF1
42kDa
β-Actin
L
Wild-type
G2019S KI
150kDa
NFAT
42kDa
β-Actin
M
Wild-type
G2019S KI
17kDa
BDNF
42kDa
β-Actin

A

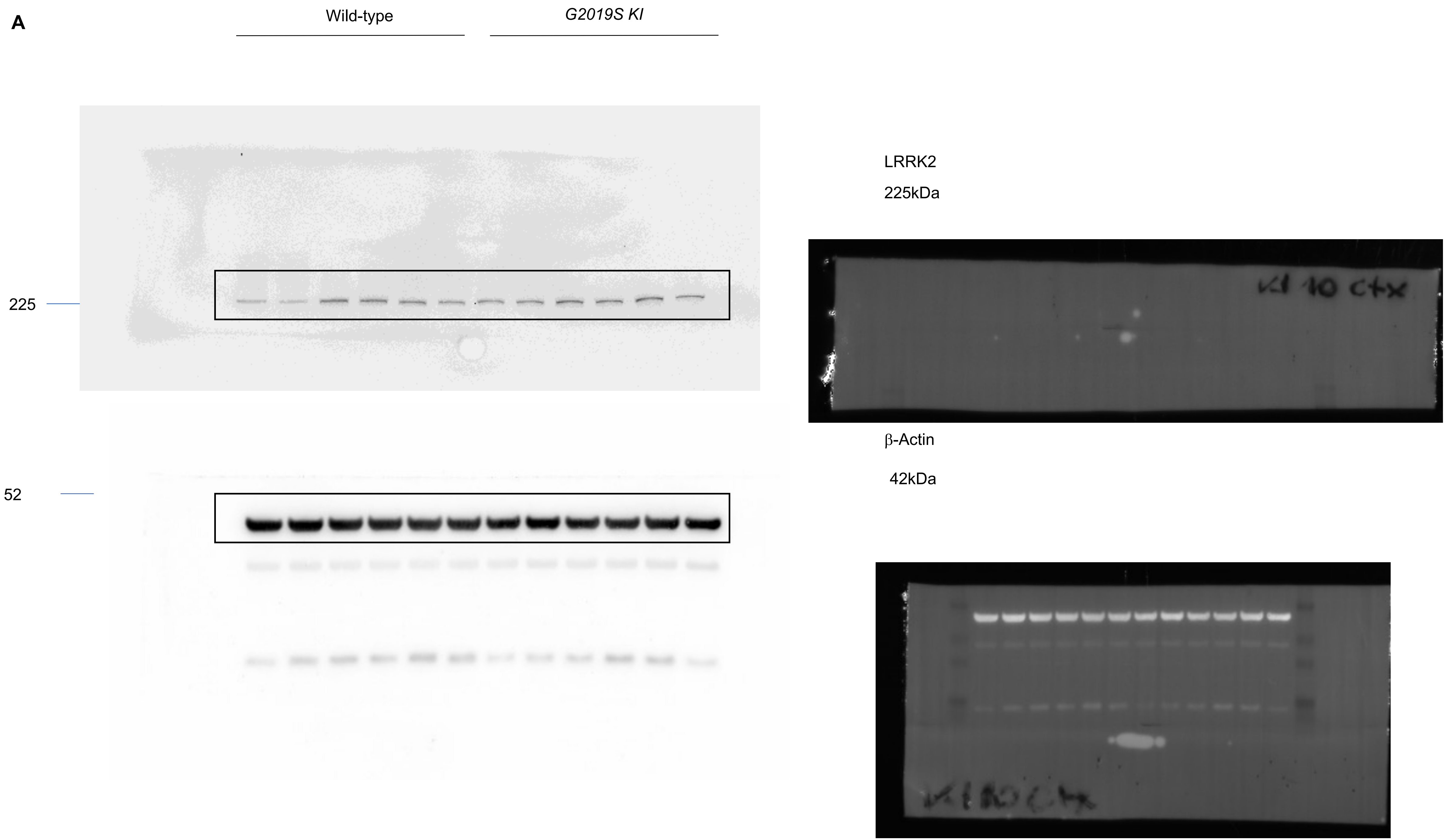

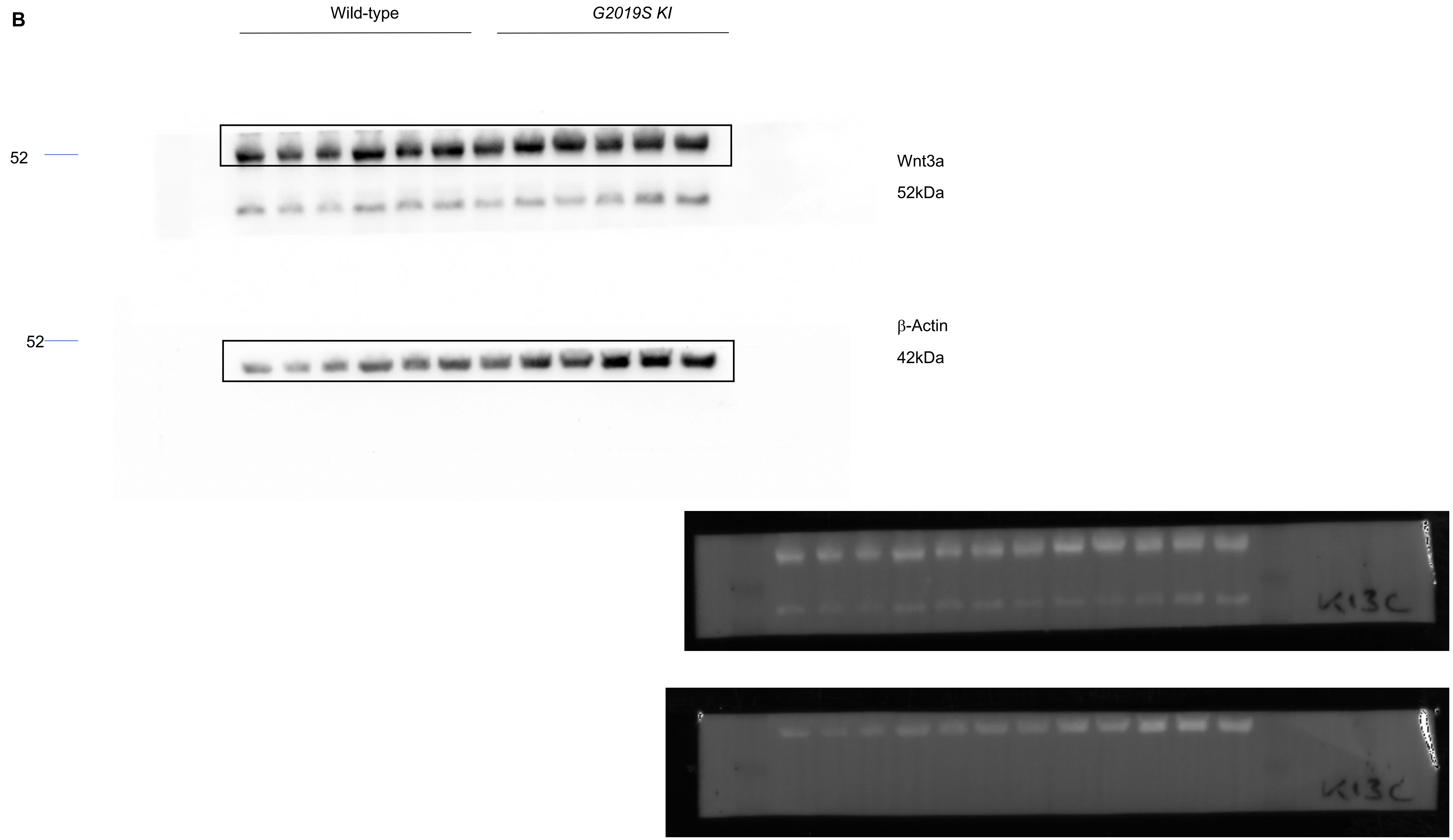
B
Wild-type
G2019S KI
52
Wnt3a
52kDa
52
β-Actin
42kDa
K13C
K13C

C

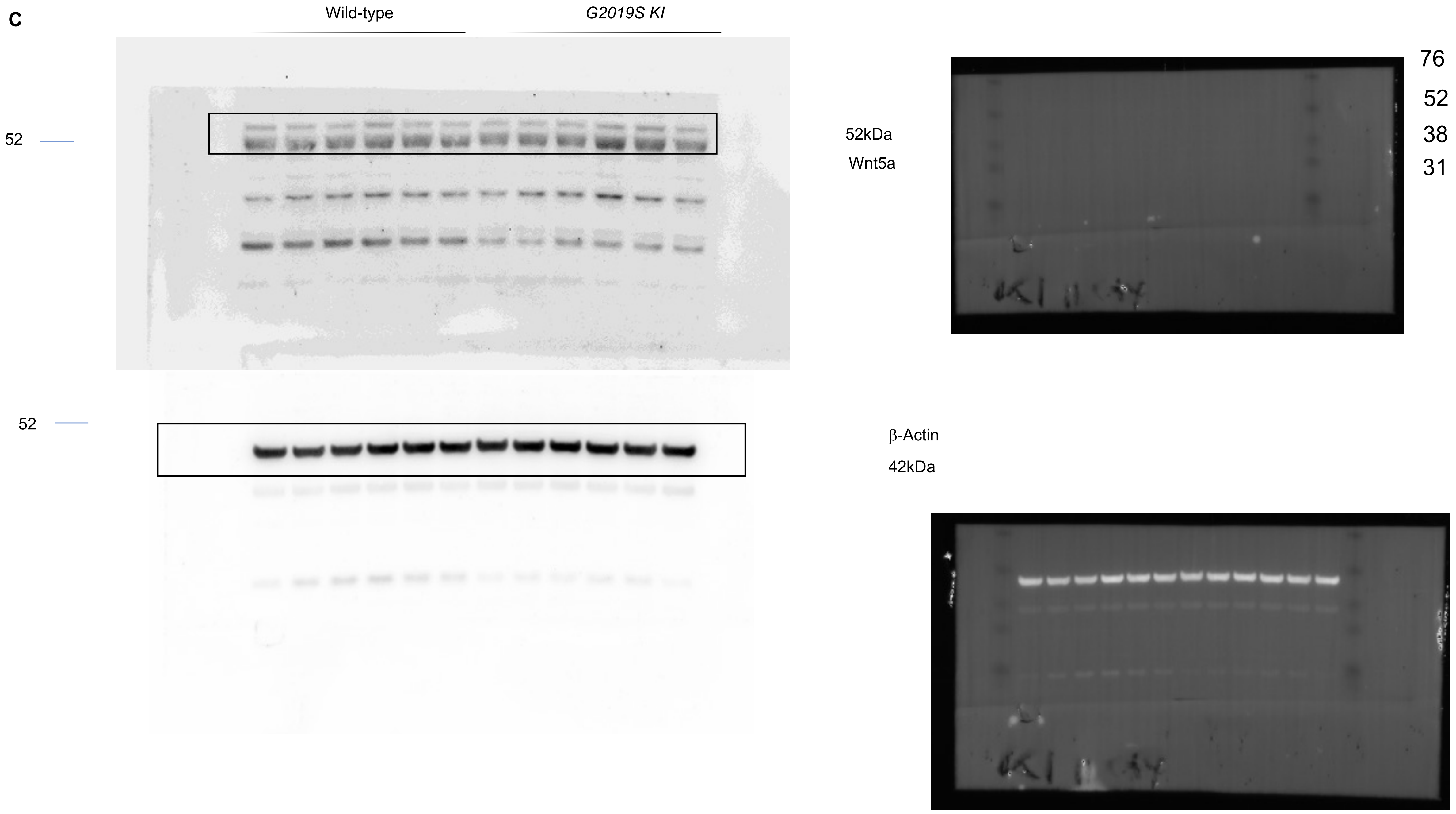

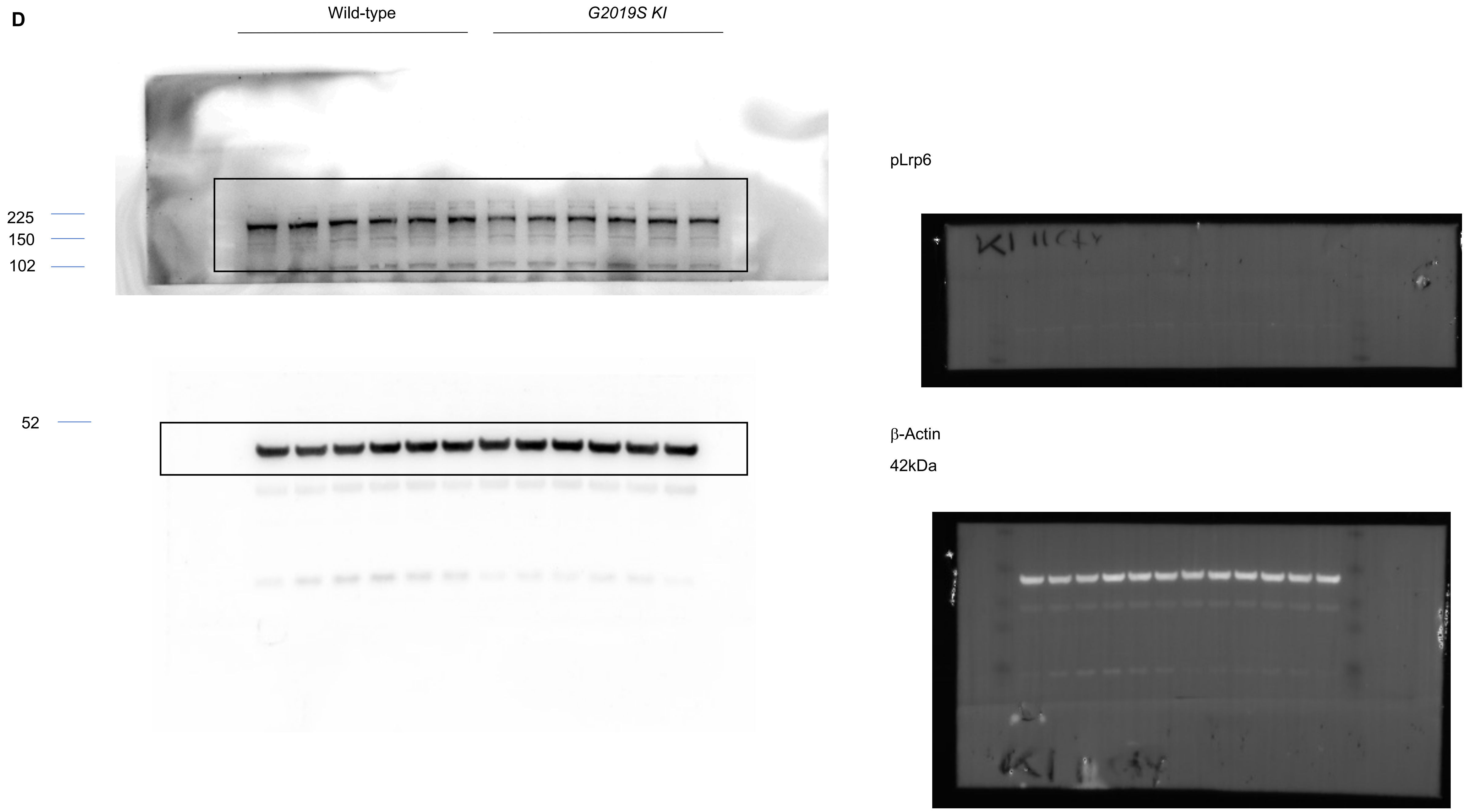
D
Wild-type
G2019S KI
225
150
102
52
pLrp6
β-Actin
42kDa

E

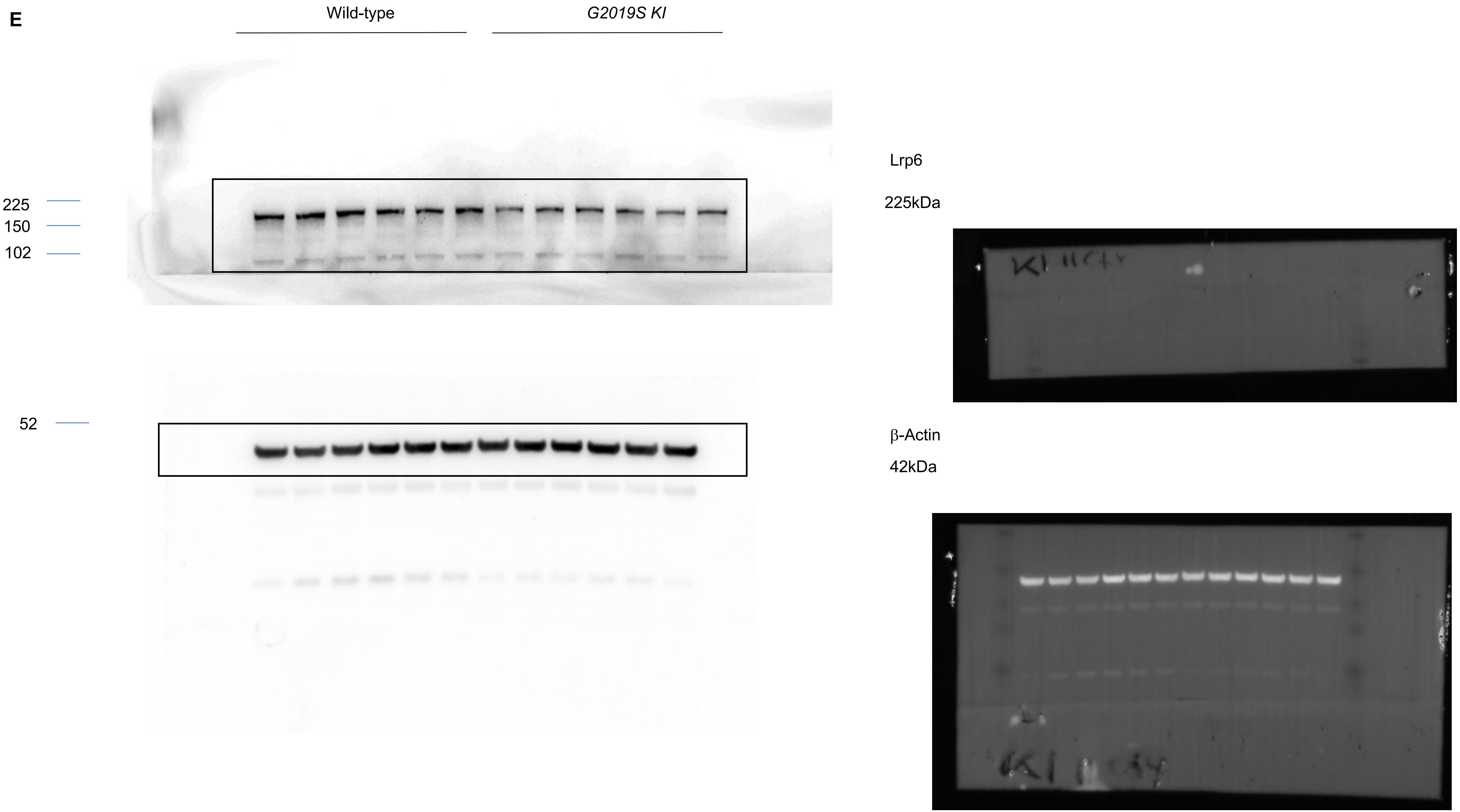

F

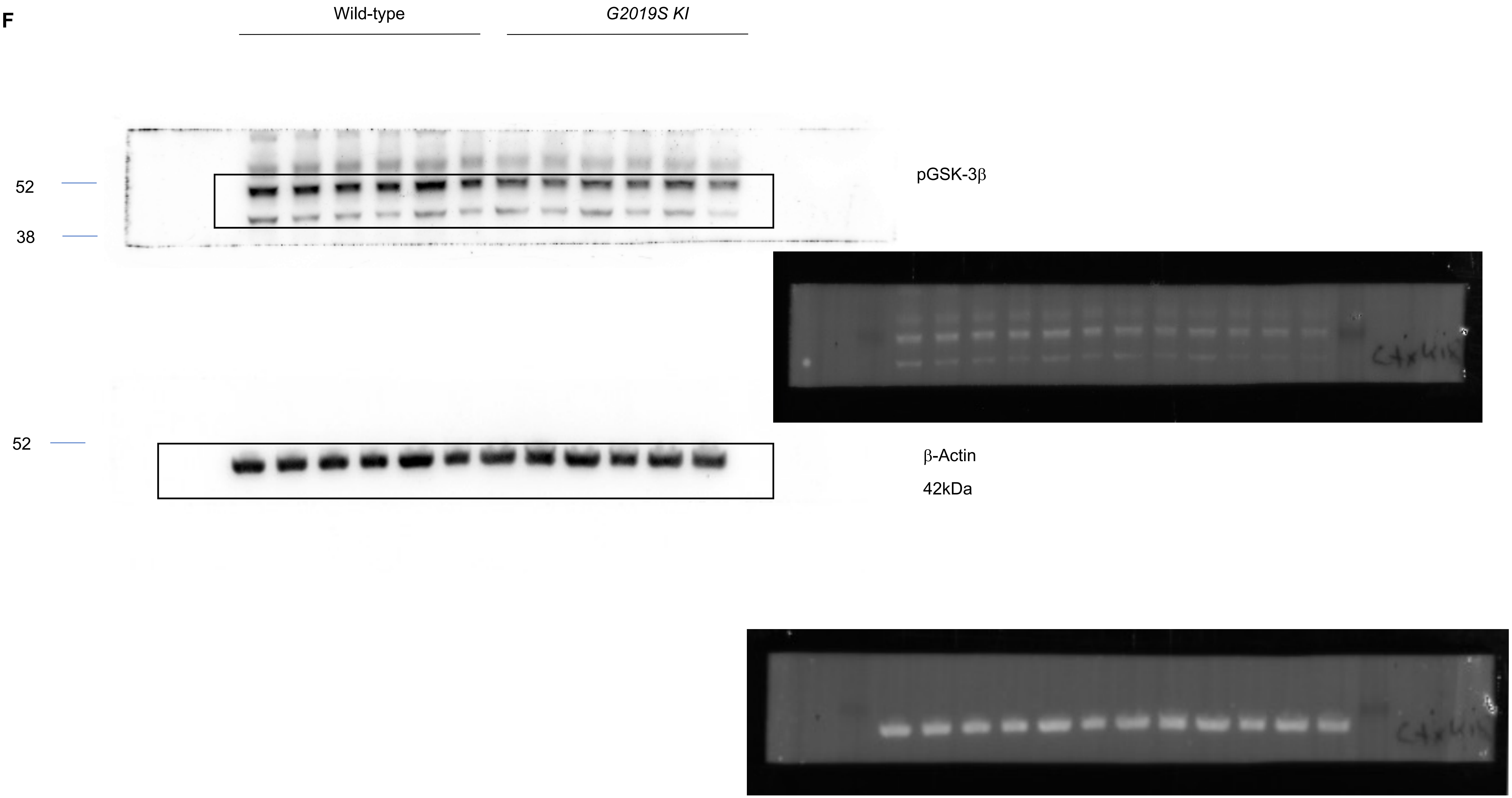

G

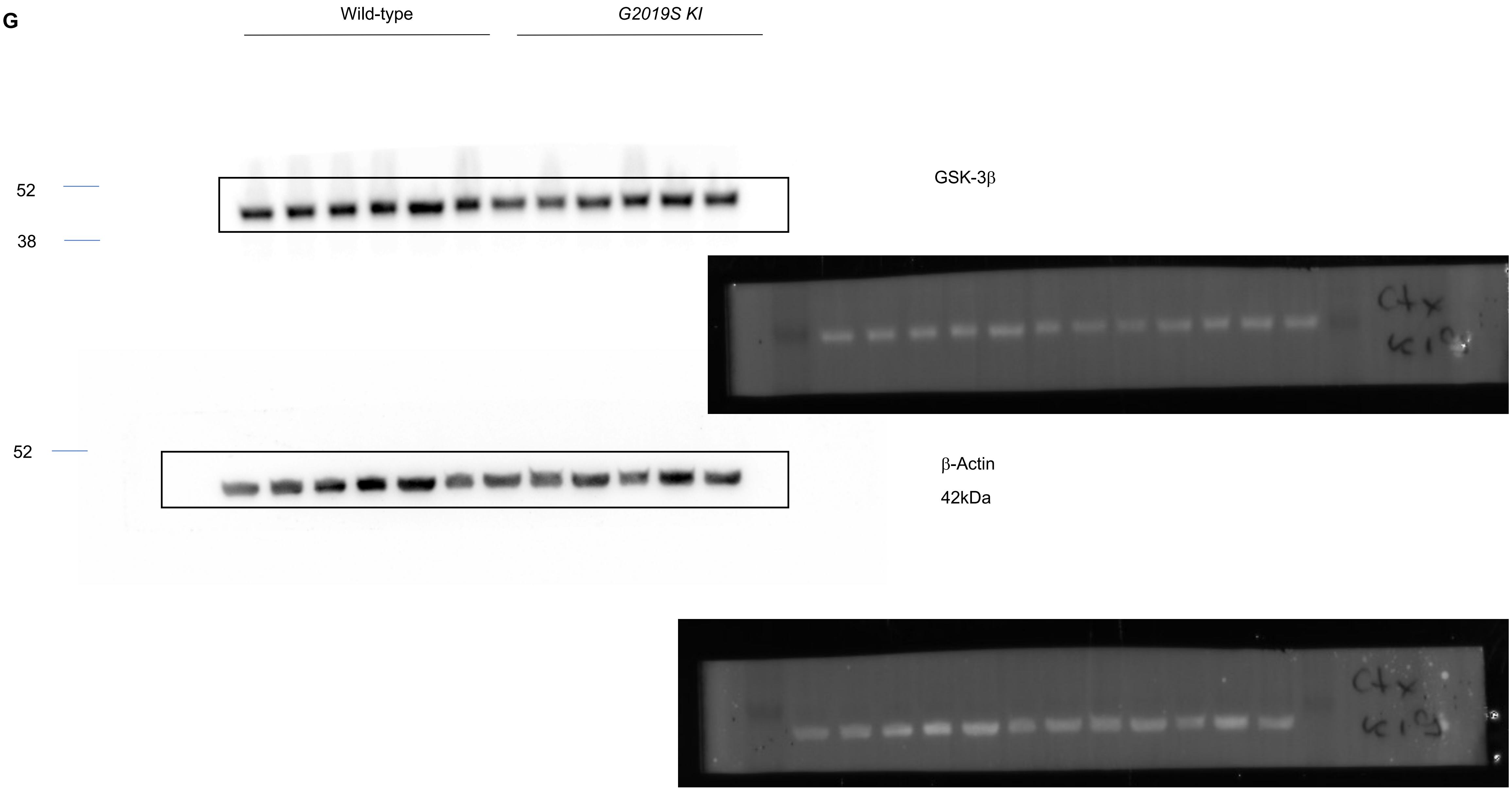

H

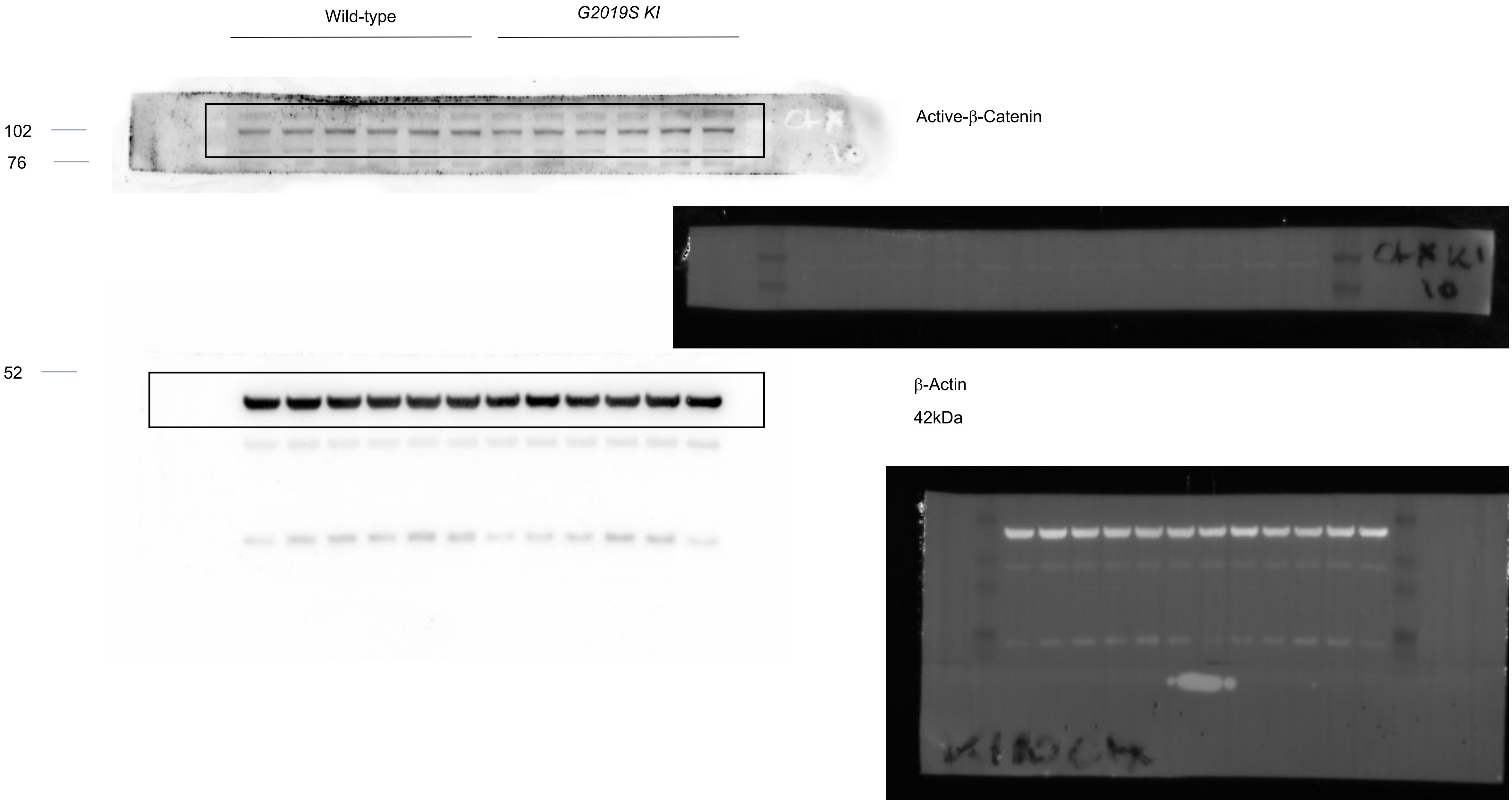

I

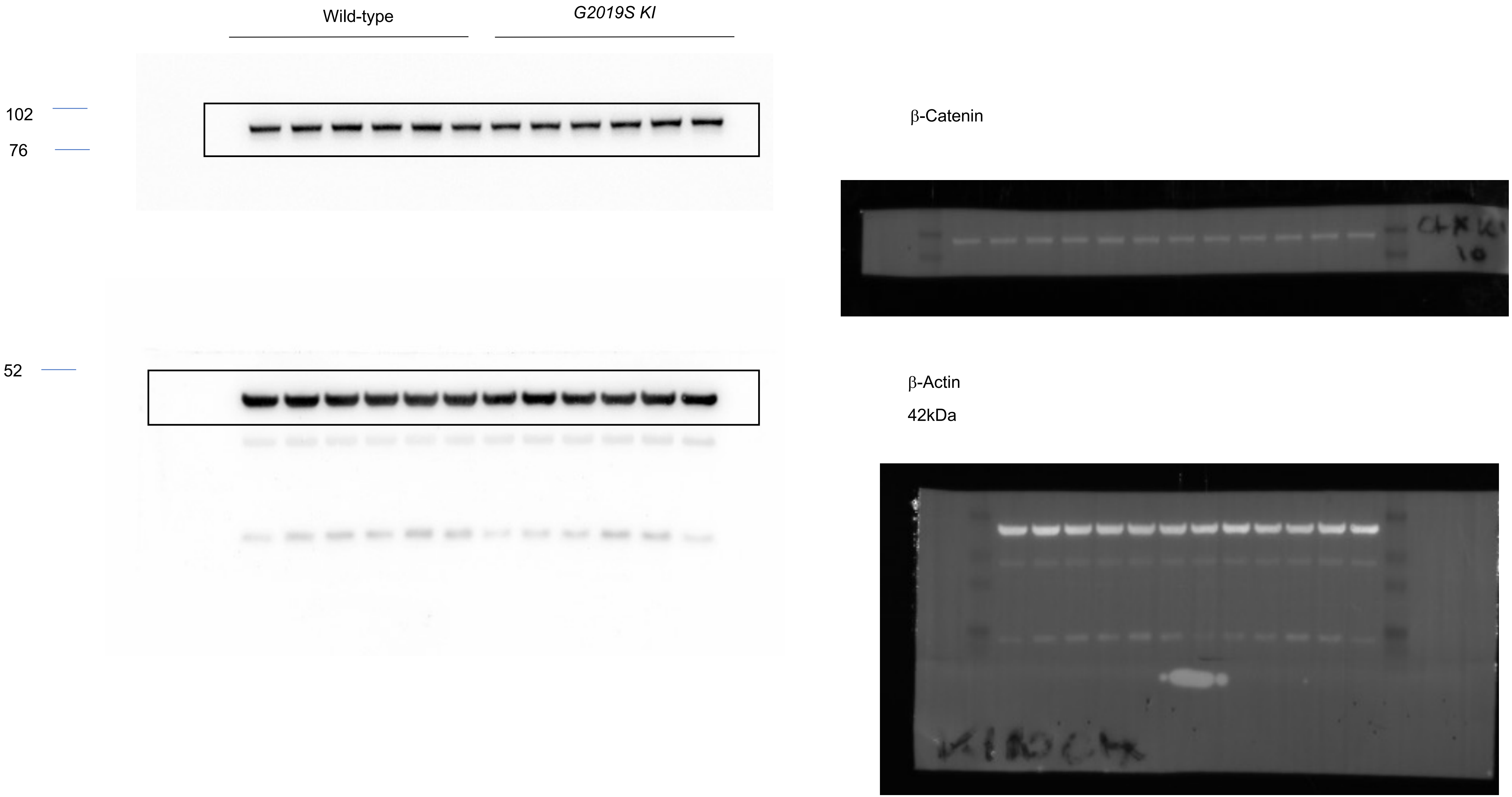

J

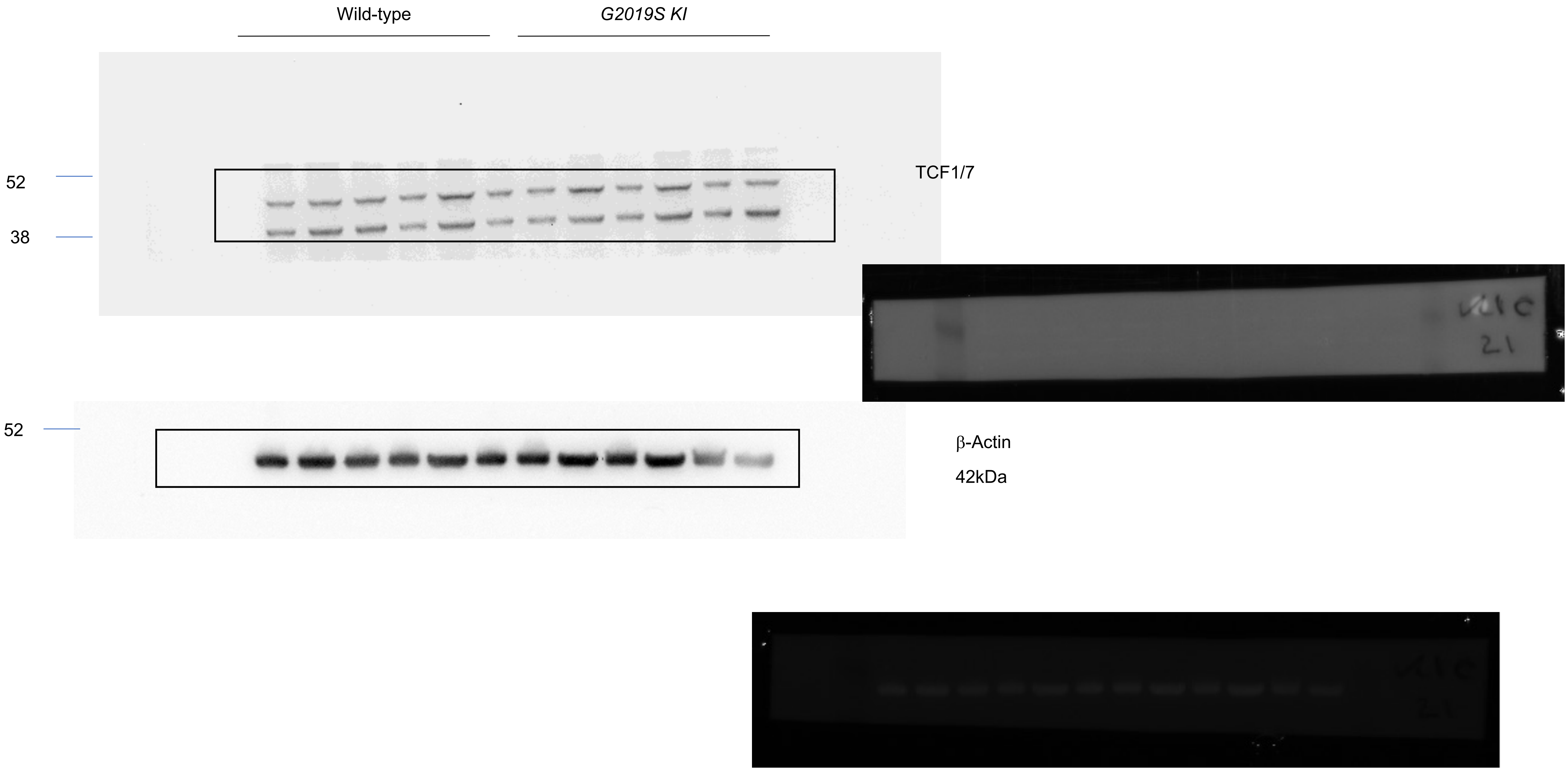

K

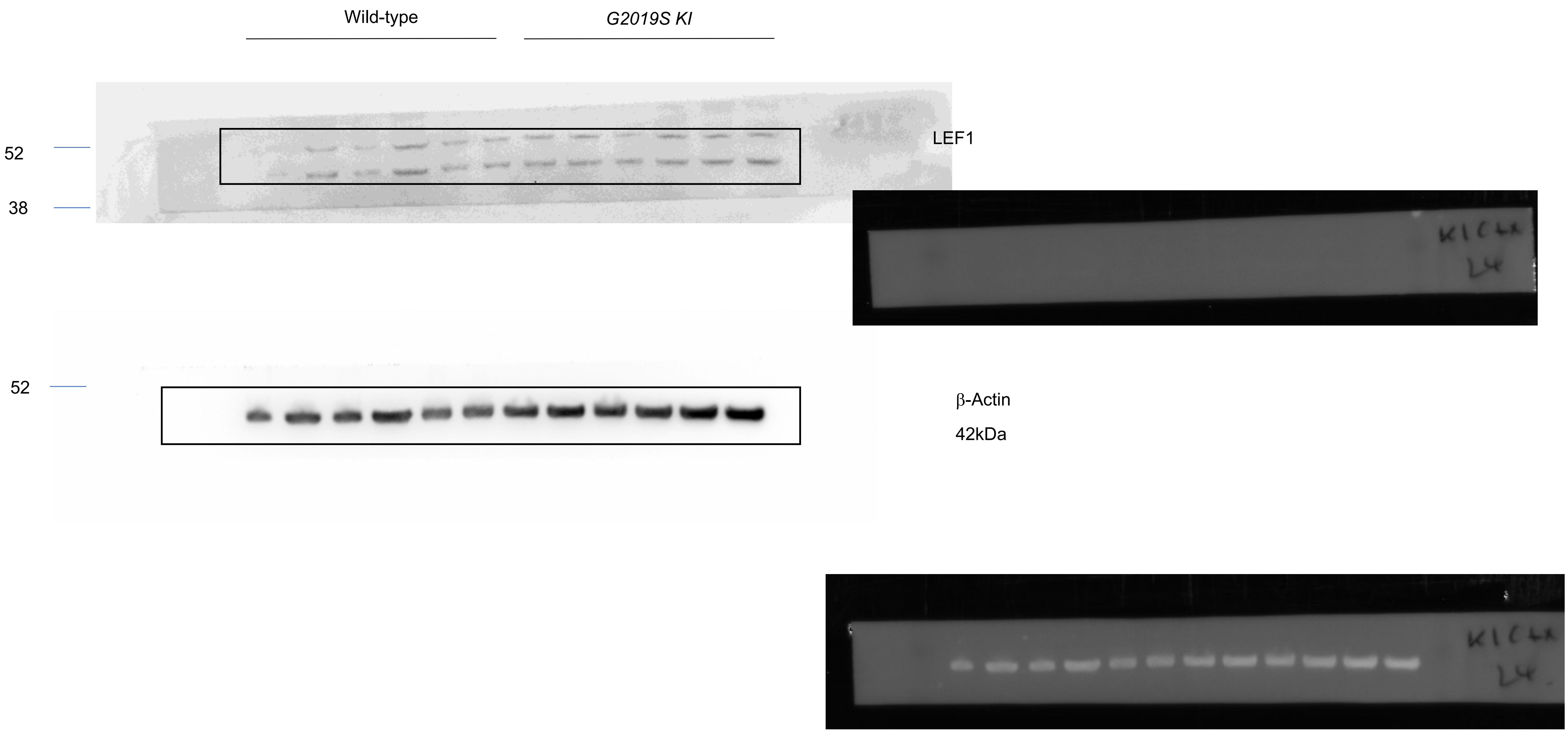

L

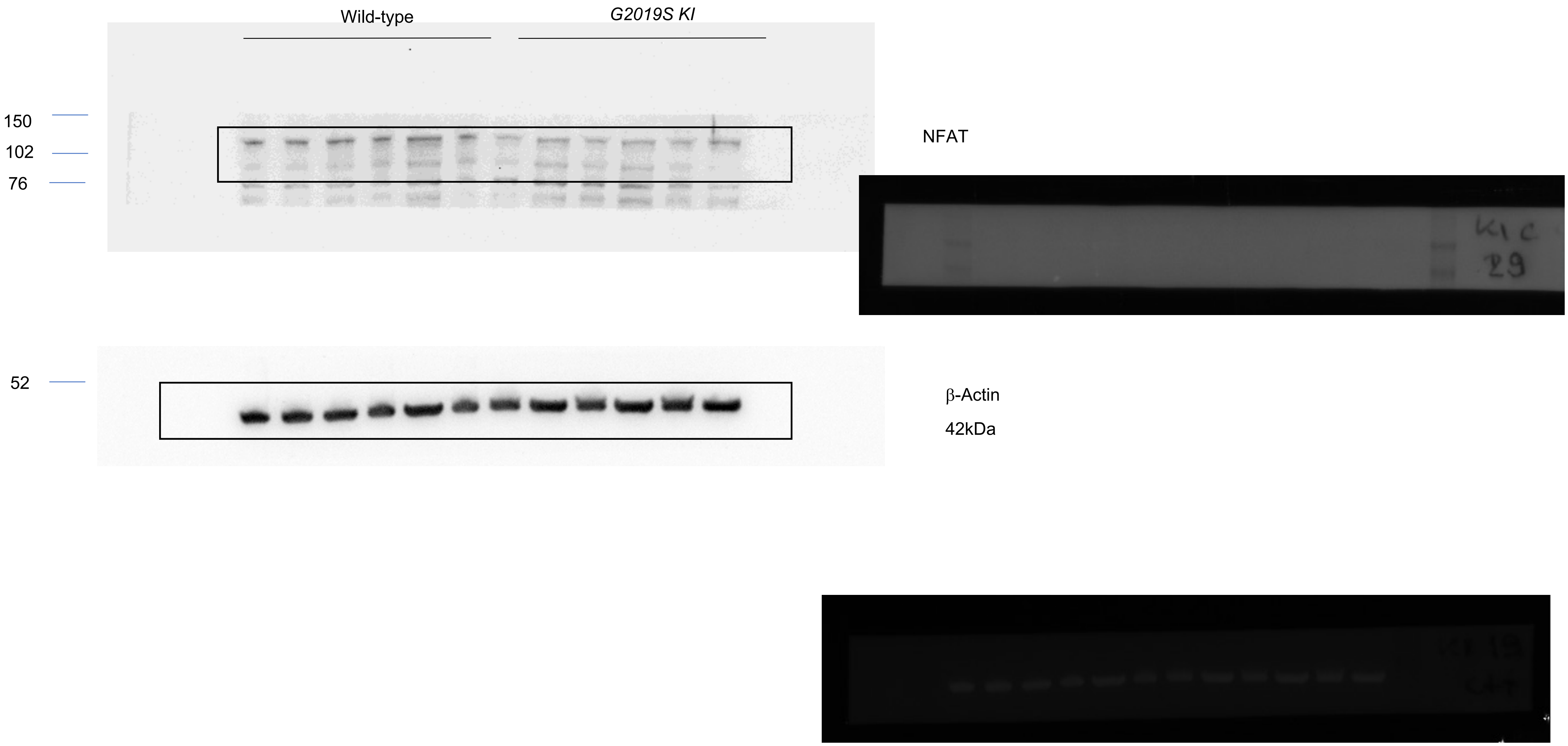

M

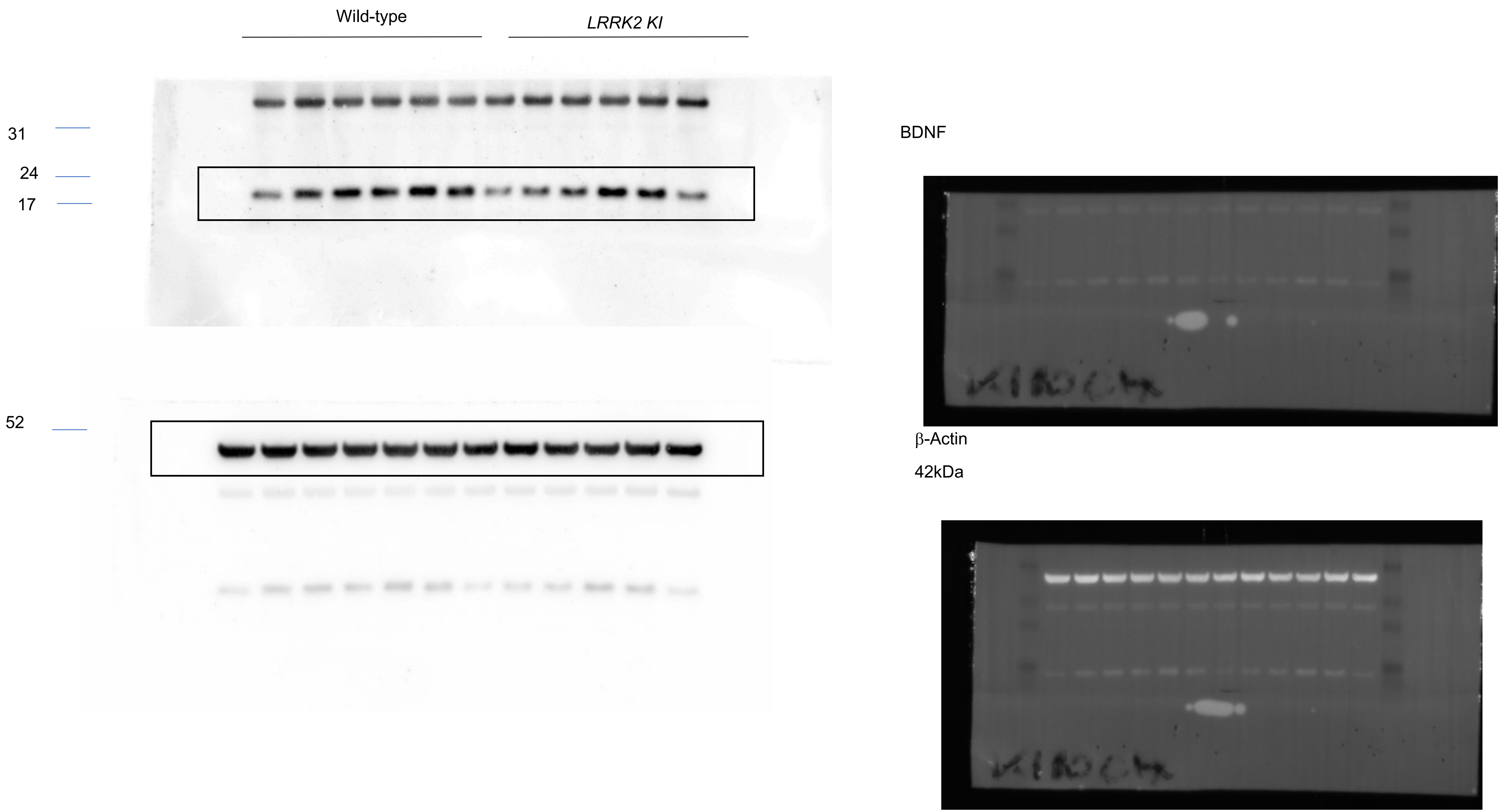

Supplement: Supplementary file 3 — Supplementary Figure 2. [file 41598_2024_63130_MOESM3_ESM.pdf]
