# Supplementary material for: Dysregulated Wnt and NFAT signaling in a Parkinson’s disease LRRK2 G2019S knock-in model
Source: Sci Rep. 2024 May 29;14:12393. doi: 10.1038/s41598-024-63130-8 (PMC11137013; doi:10.1038/s41598-024-63130-8)

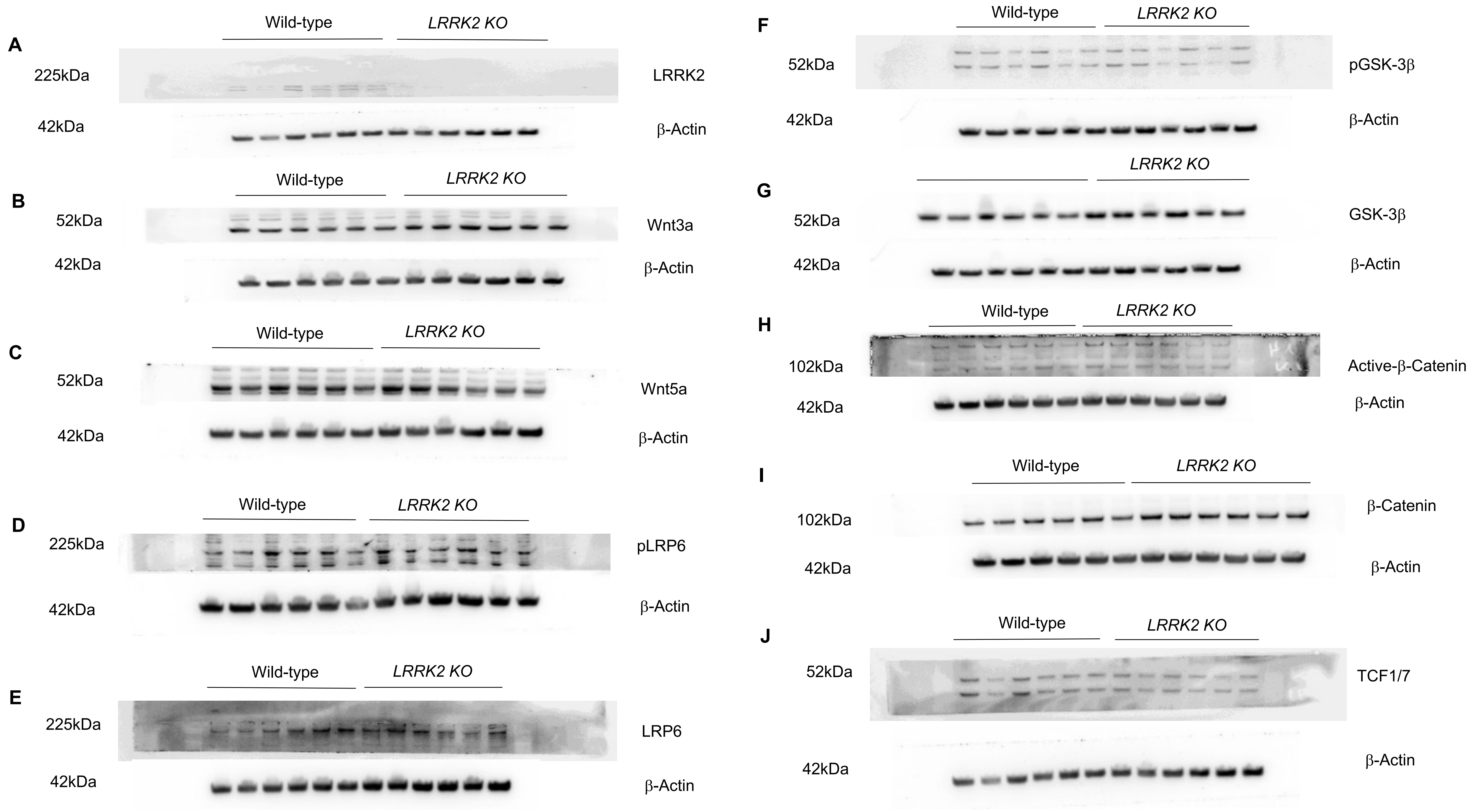
A
Wild-type
LRRK2 KO
225kDa
LRRK2
42kDa
β-Actin
B
Wild-type
LRRK2 KO
52kDa
Wnt3a
42kDa
β-Actin
C
Wild-type
LRRK2 KO
52kDa
Wnt5a
42kDa
β-Actin
D
Wild-type
LRRK2 KO
225kDa
pLRP6
42kDa
β-Actin
E
Wild-type
LRRK2 KO
225kDa
LRP6
42kDa
β-Actin
F
Wild-type
LRRK2 KO
52kDa
pGSK-3β
42kDa
β-Actin
G
LRRK2 KO
52kDa
GSK-3β
42kDa
β-Actin
H
Wild-type
LRRK2 KO
102kDa
Active-β-Catenin
42kDa
β-Actin
I
Wild-type
LRRK2 KO
102kDa
β-Catenin
42kDa
β-Actin
J
Wild-type
LRRK2 KO
52kDa
TCF1/7
42kDa
β-Actin

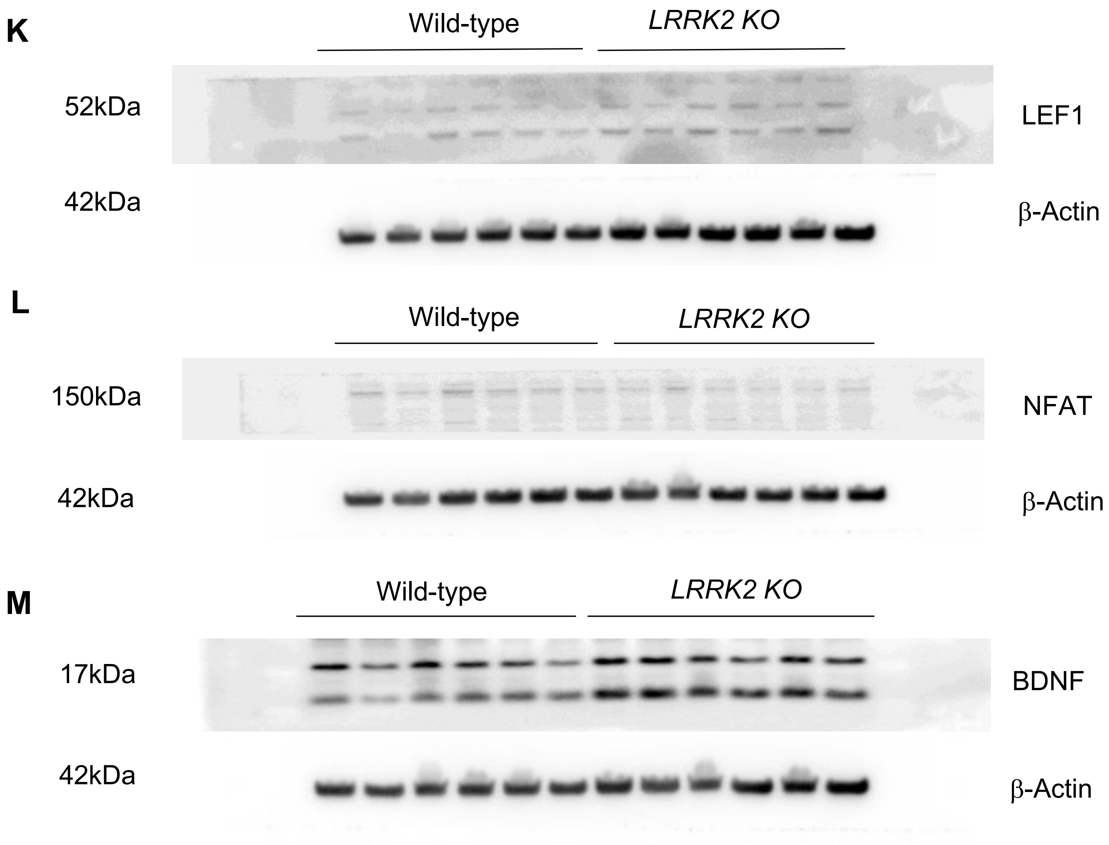
K
Wild-type
LRRK2 KO
52kDa
LEF1
42kDa
β-Actin
L
Wild-type
LRRK2 KO
150kDa
NFAT
42kDa
β-Actin
M
Wild-type
LRRK2 KO
17kDa
BDNF
42kDa
β-Actin

A

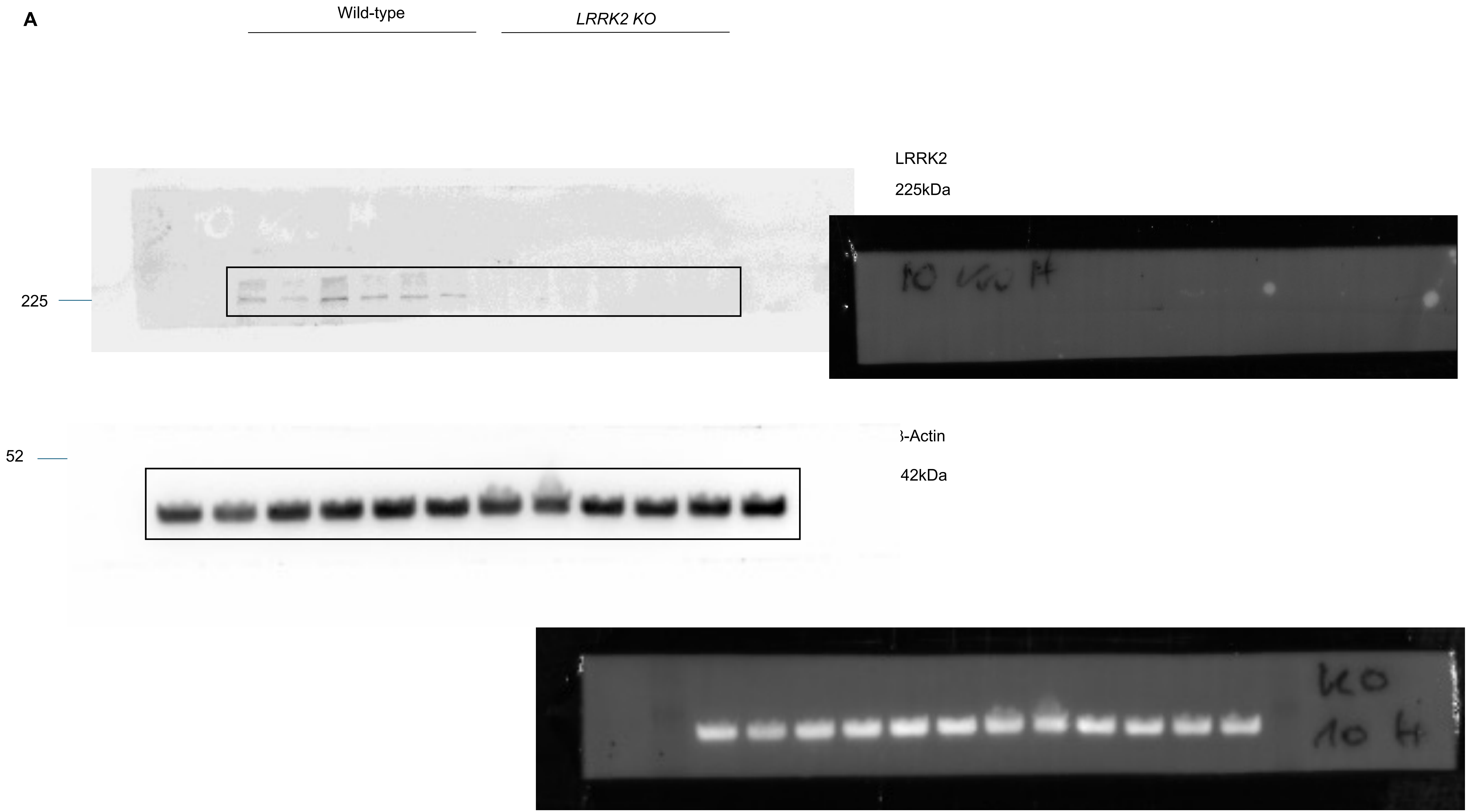

B

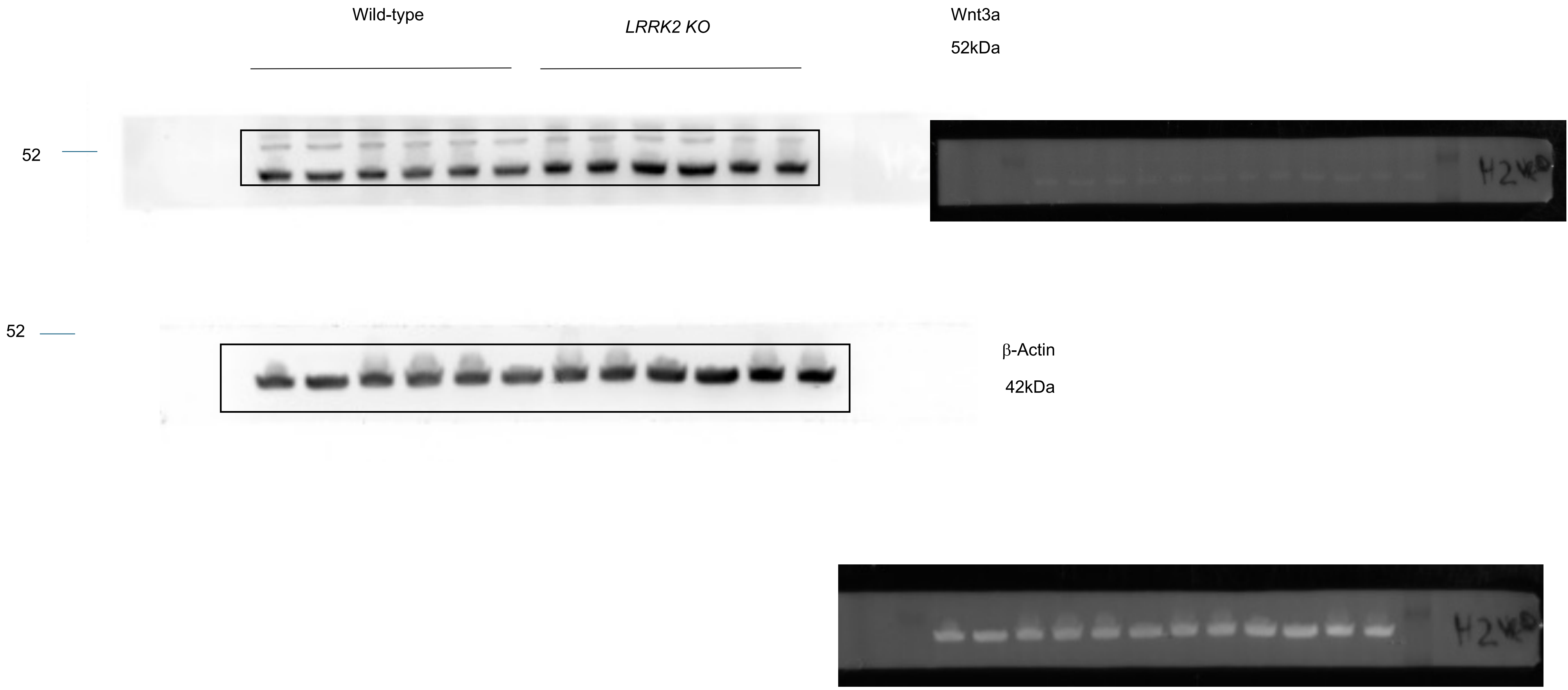

C

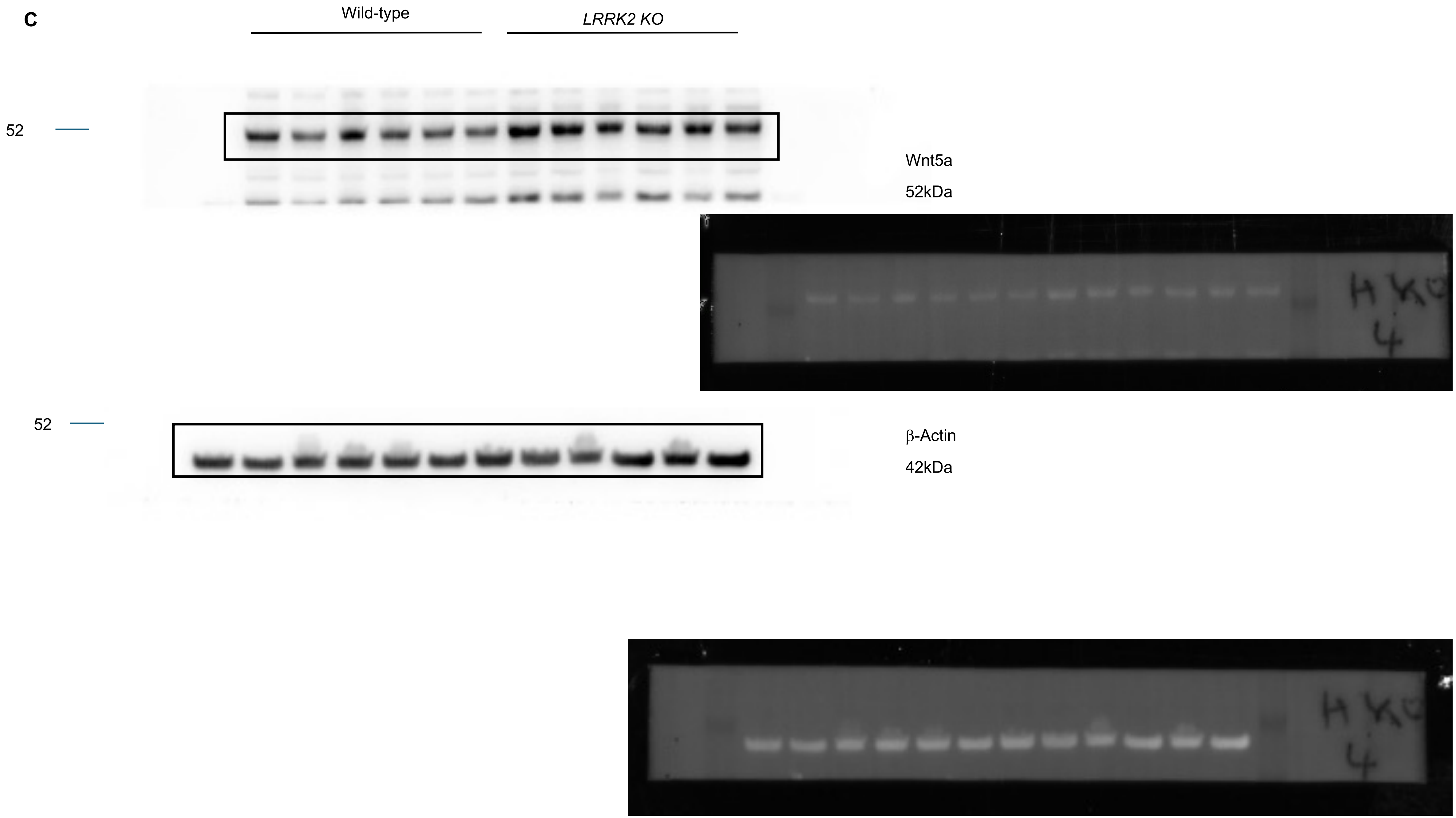

D

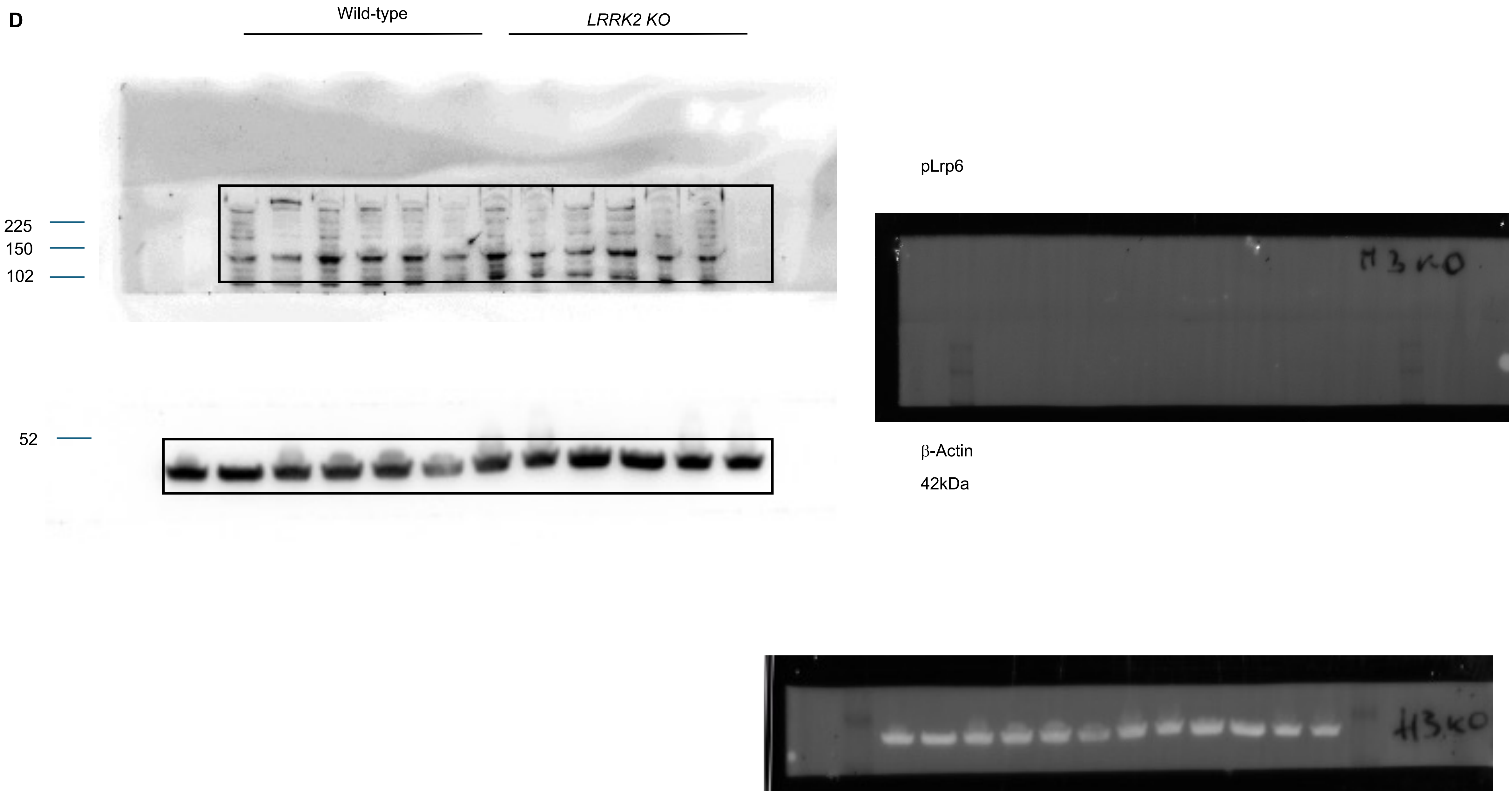

E

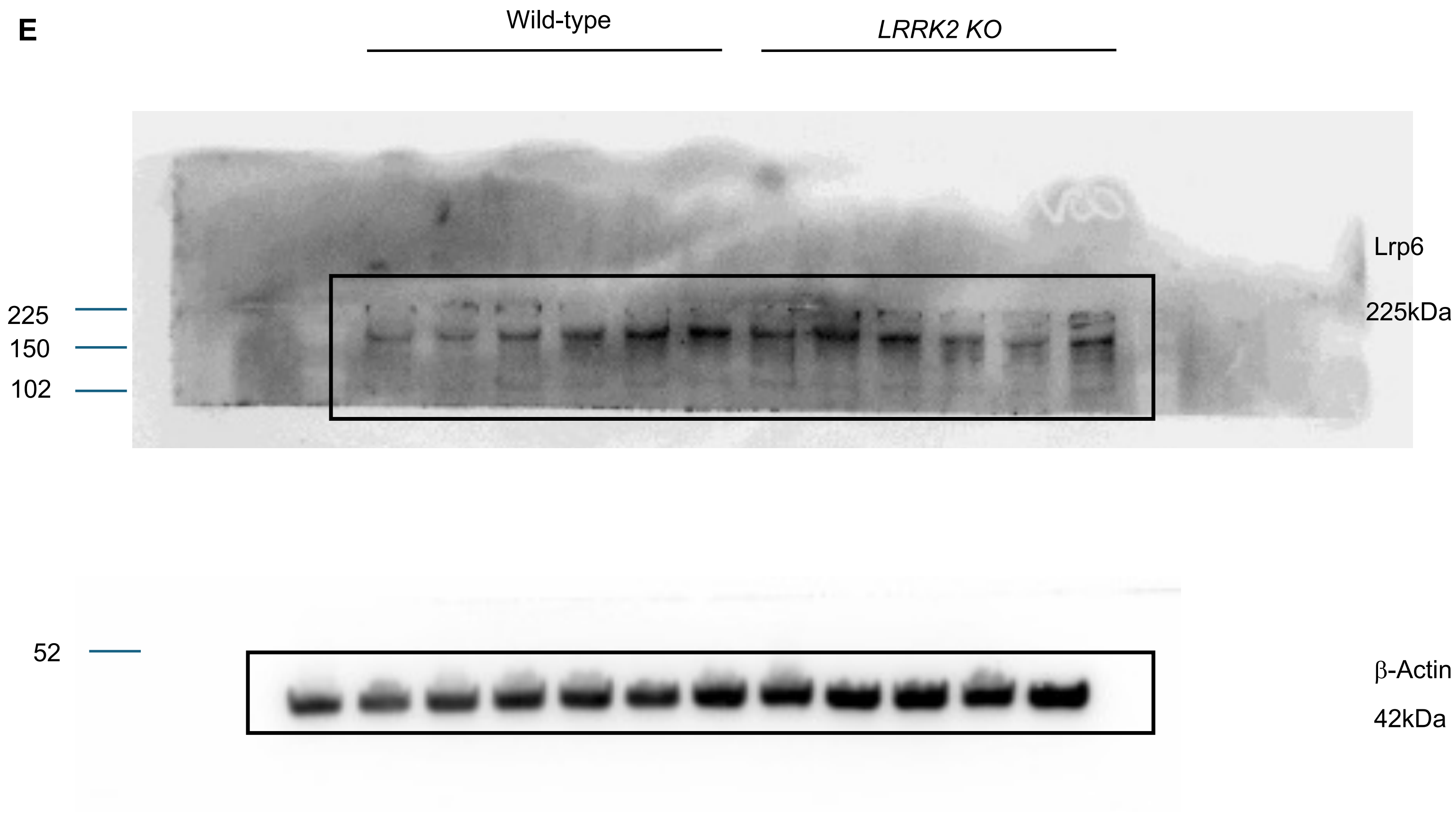

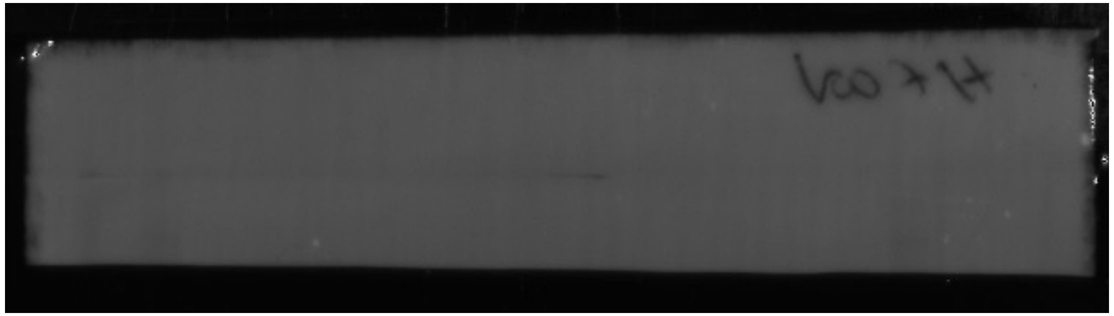

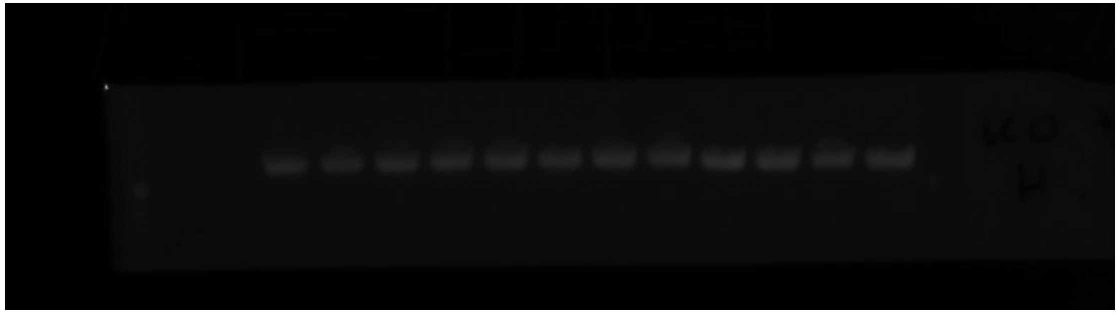

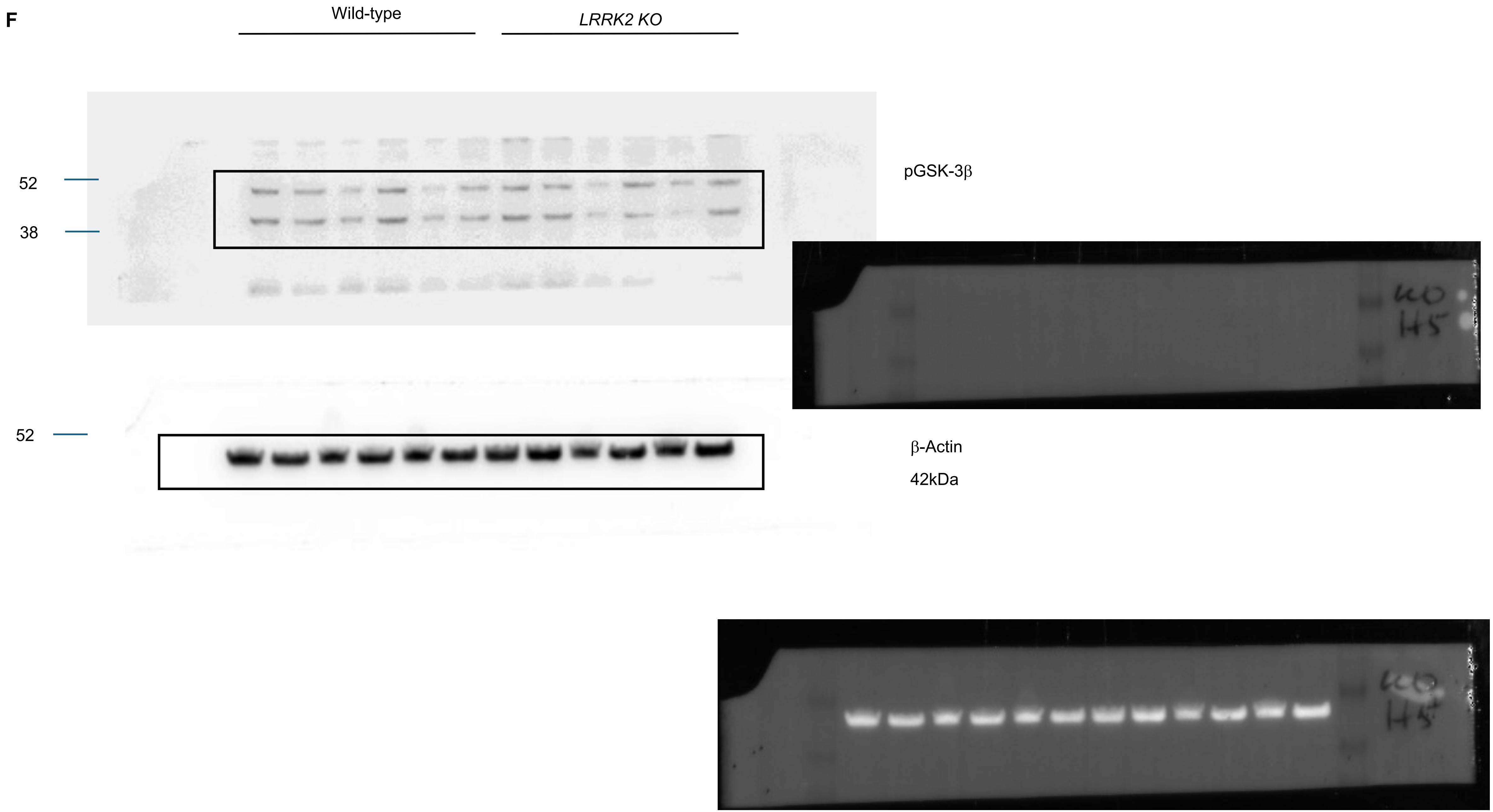
F
Wild-type
LRRK2 KO
52
38
pGSK-3β
52
β-Actin
42kDa
KO
H5
KO
H5

G

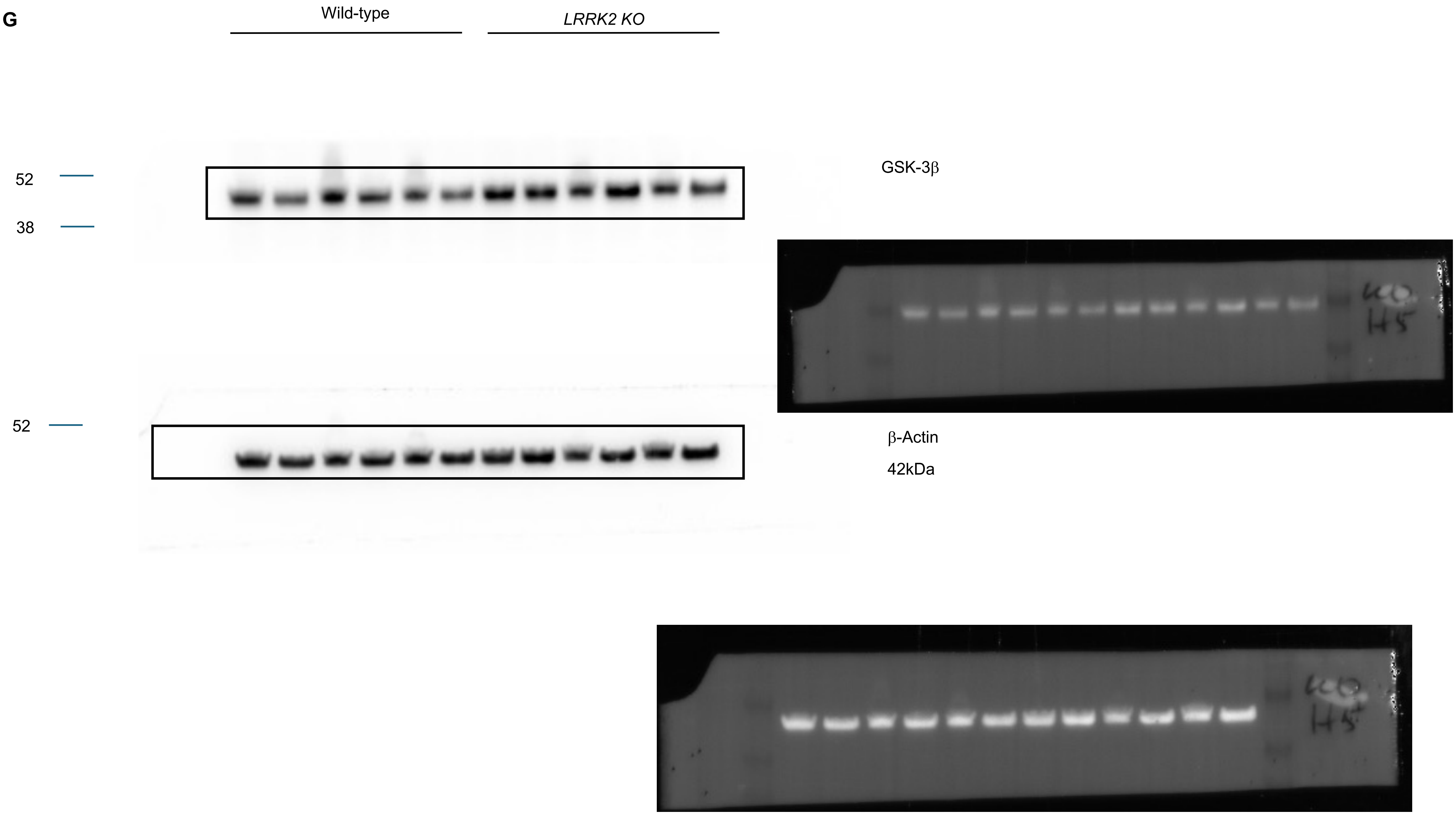

H

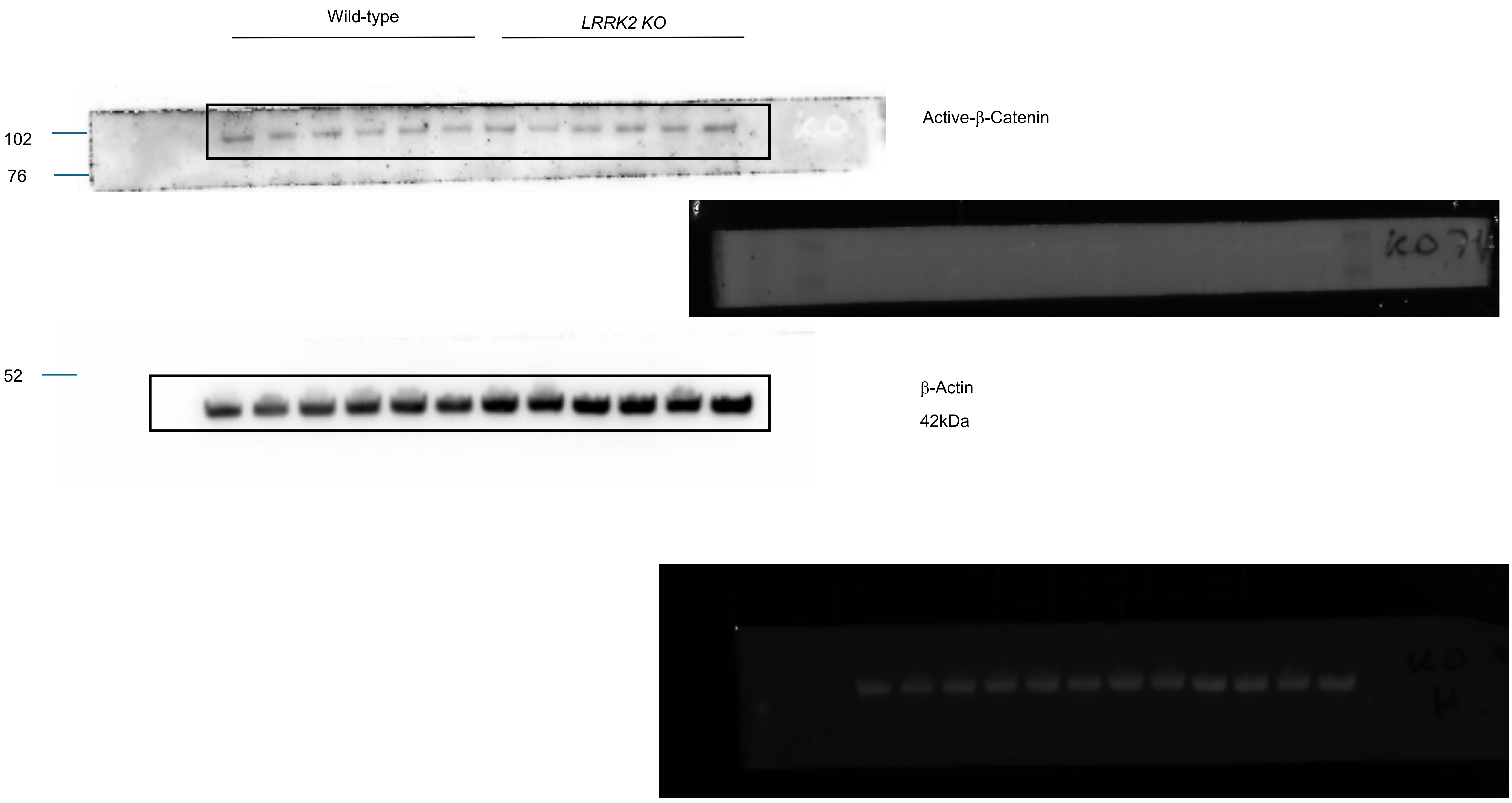

Wild-type
LRRK2 KO
102
76
Active-β-Catenin
KO
KO7
52
β-Actin
42kDa

I

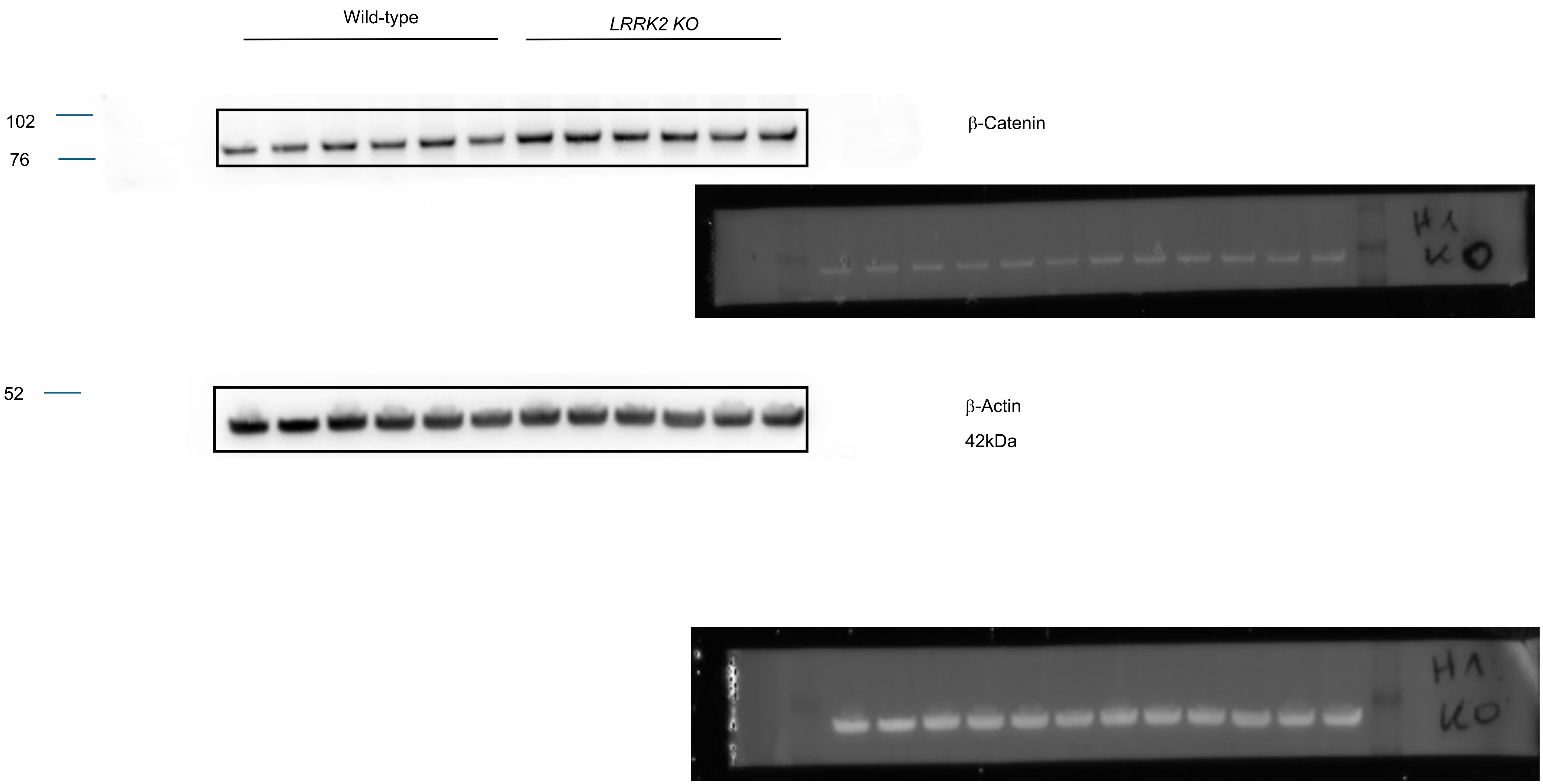

J

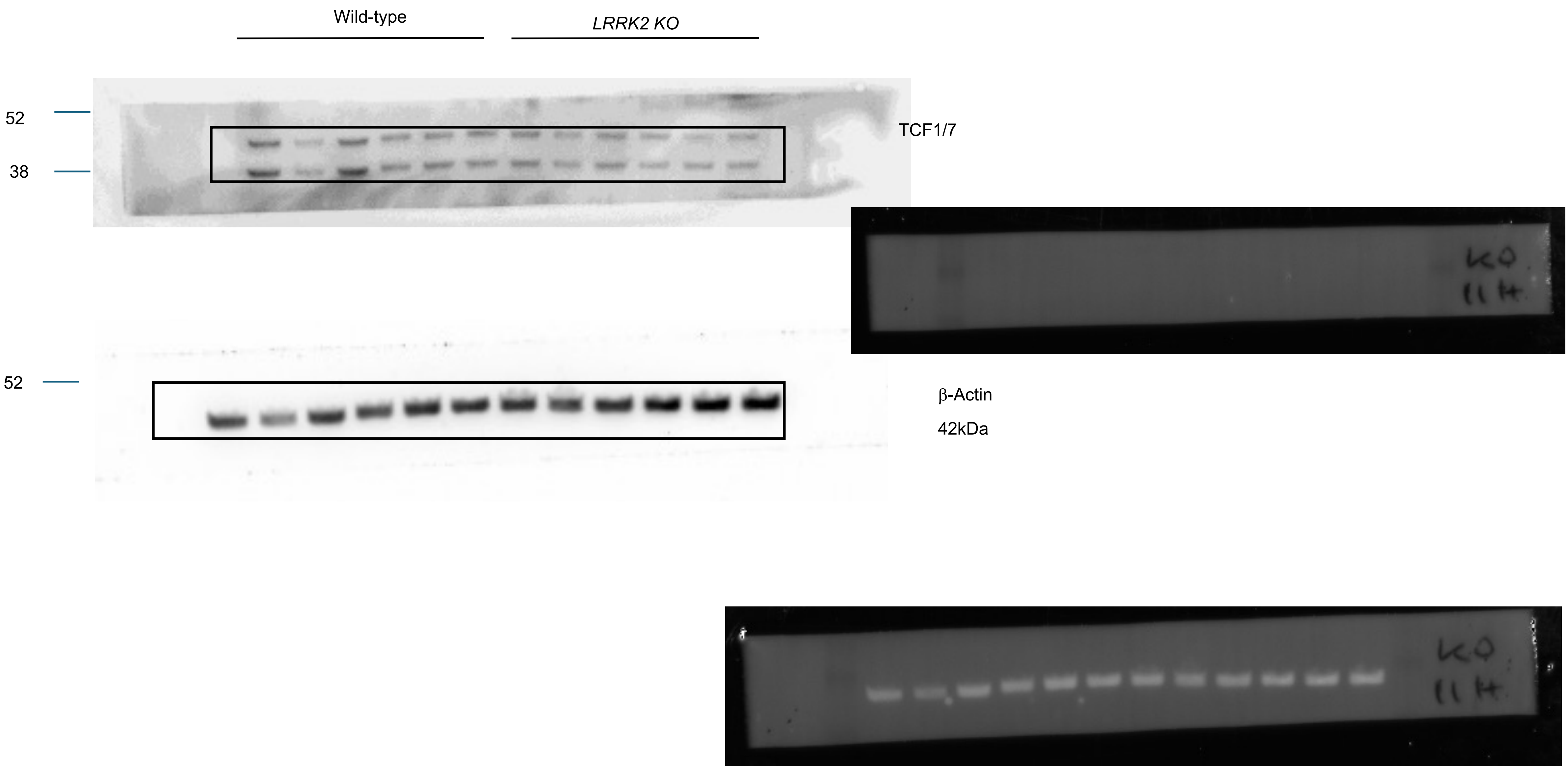

K

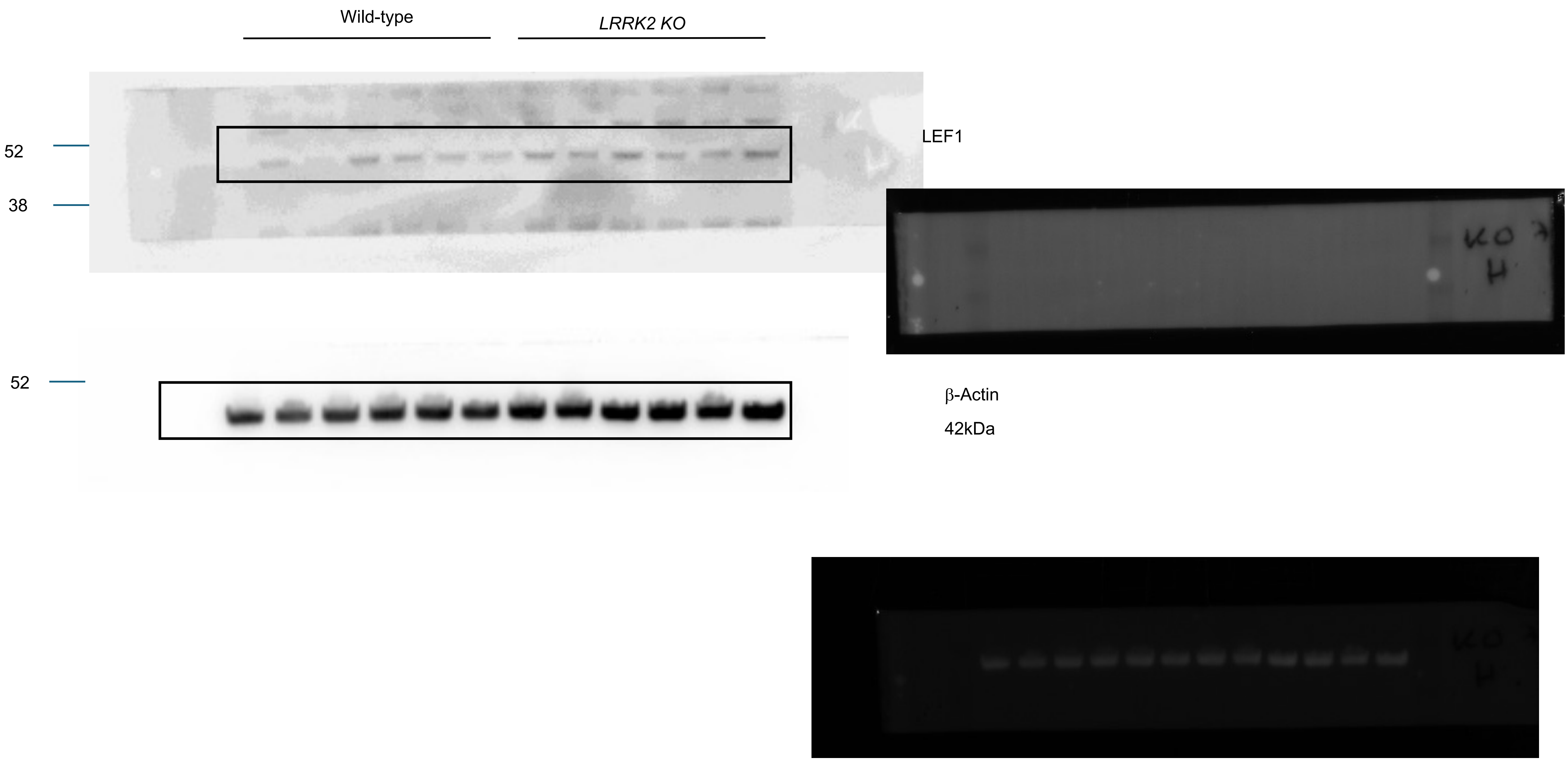

L

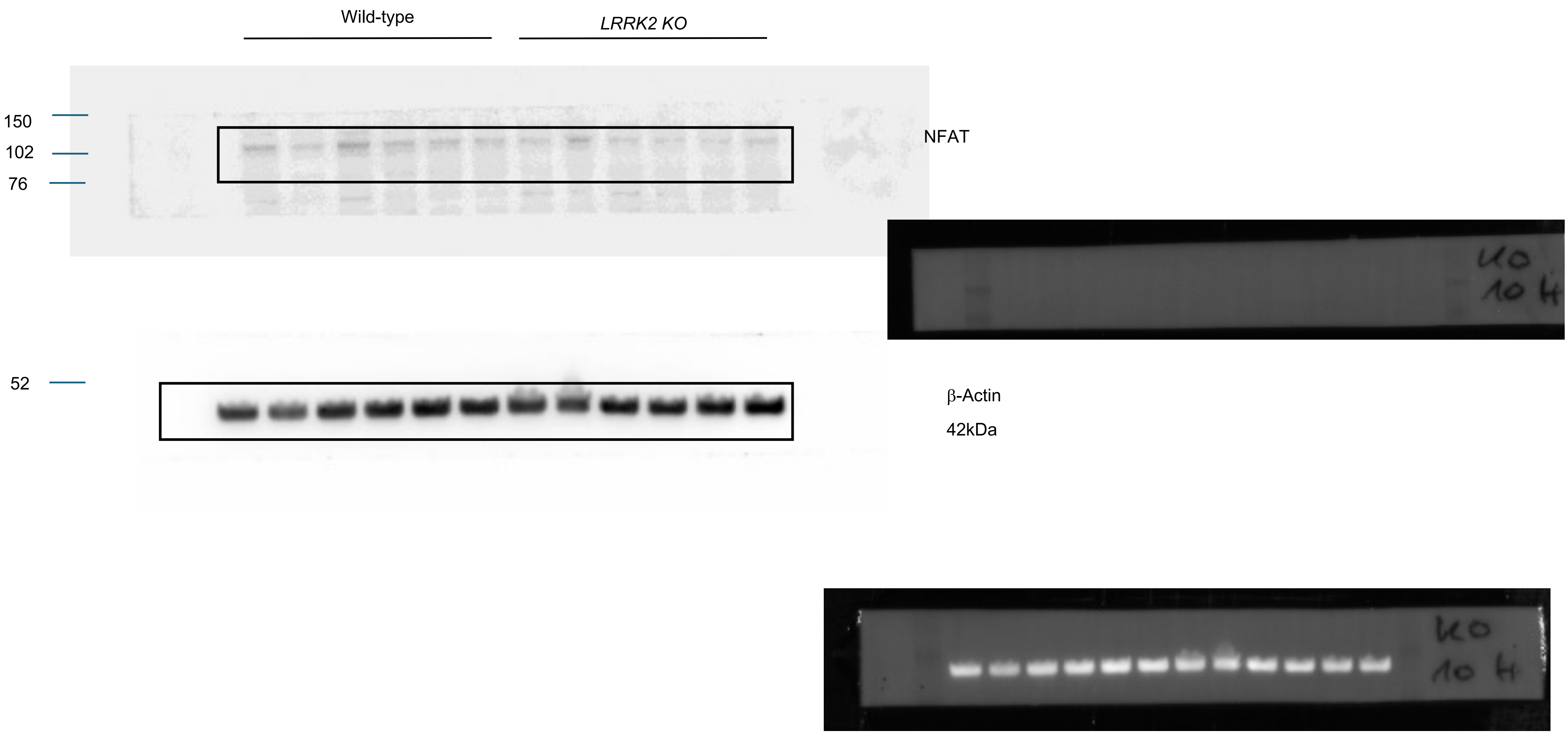

M

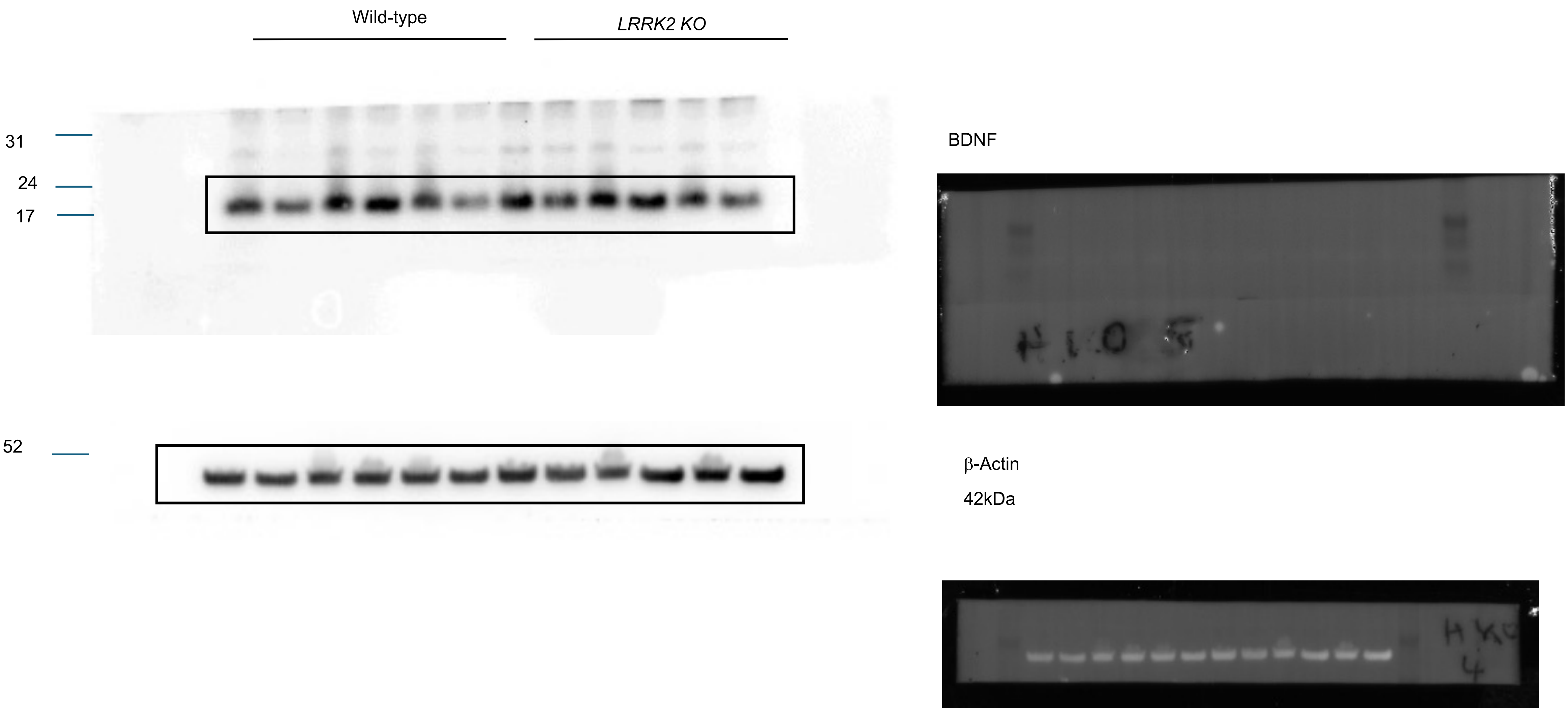

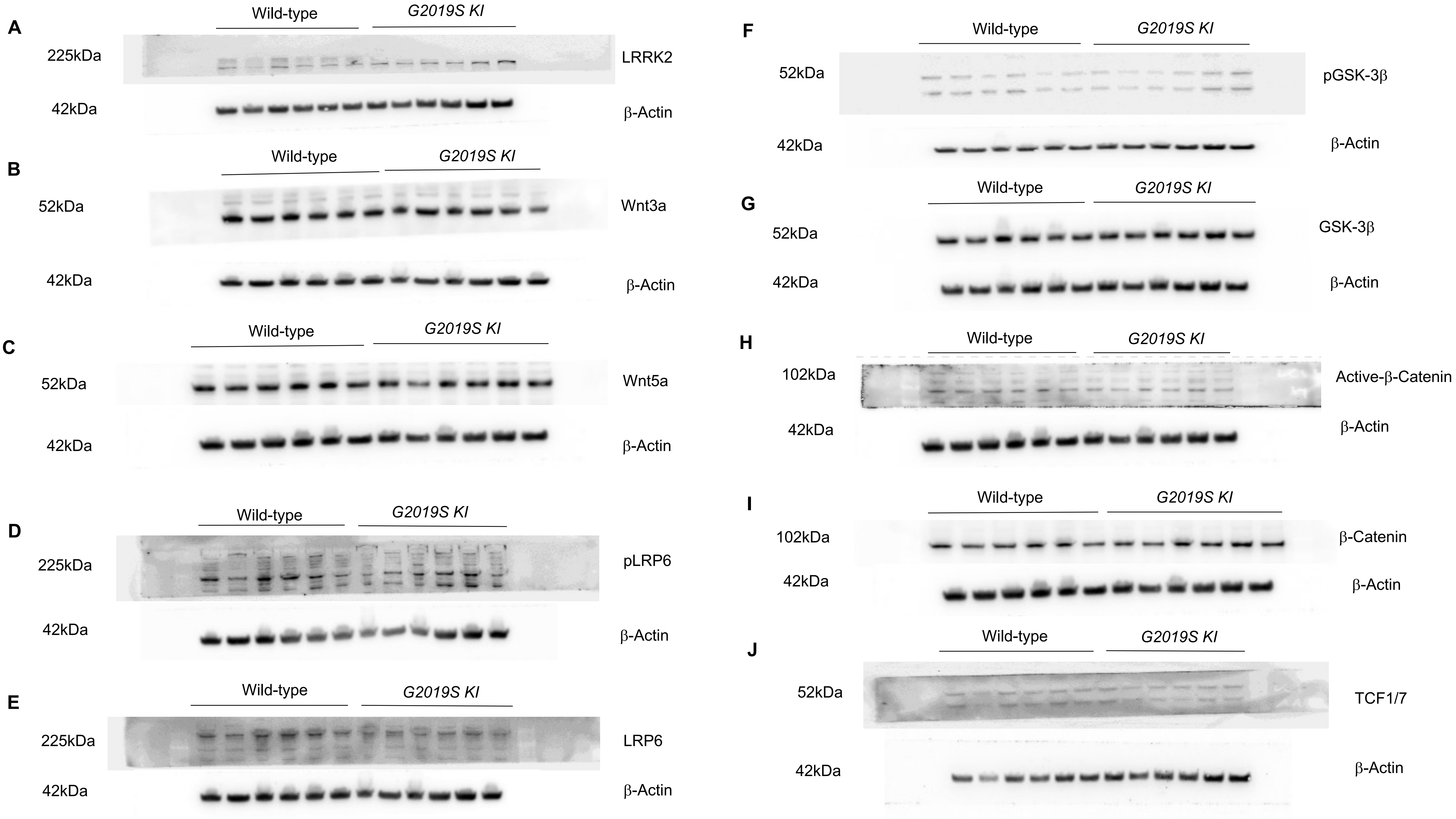

A
Wild-type
G2019S KI
225kDa
LRRK2
42kDa
β-Actin
B
Wild-type
G2019S KI
52kDa
Wnt3a
42kDa
β-Actin
C
Wild-type
G2019S KI
52kDa
Wnt5a
42kDa
β-Actin
D
Wild-type
G2019S KI
225kDa
pLRP6
42kDa
β-Actin
E
Wild-type
G2019S KI
225kDa
LRP6
42kDa
β-Actin
F
Wild-type
G2019S KI
52kDa
pGSK-3β
42kDa
β-Actin
G
Wild-type
G2019S KI
52kDa
GSK-3β
42kDa
β-Actin
H
Wild-type
G2019S KI
102kDa
Active-β-Catenin
42kDa
β-Actin
I
Wild-type
G2019S KI
102kDa
β-Catenin
42kDa
β-Actin
J
Wild-type
G2019S KI
52kDa
TCF1/7
42kDa
β-Actin

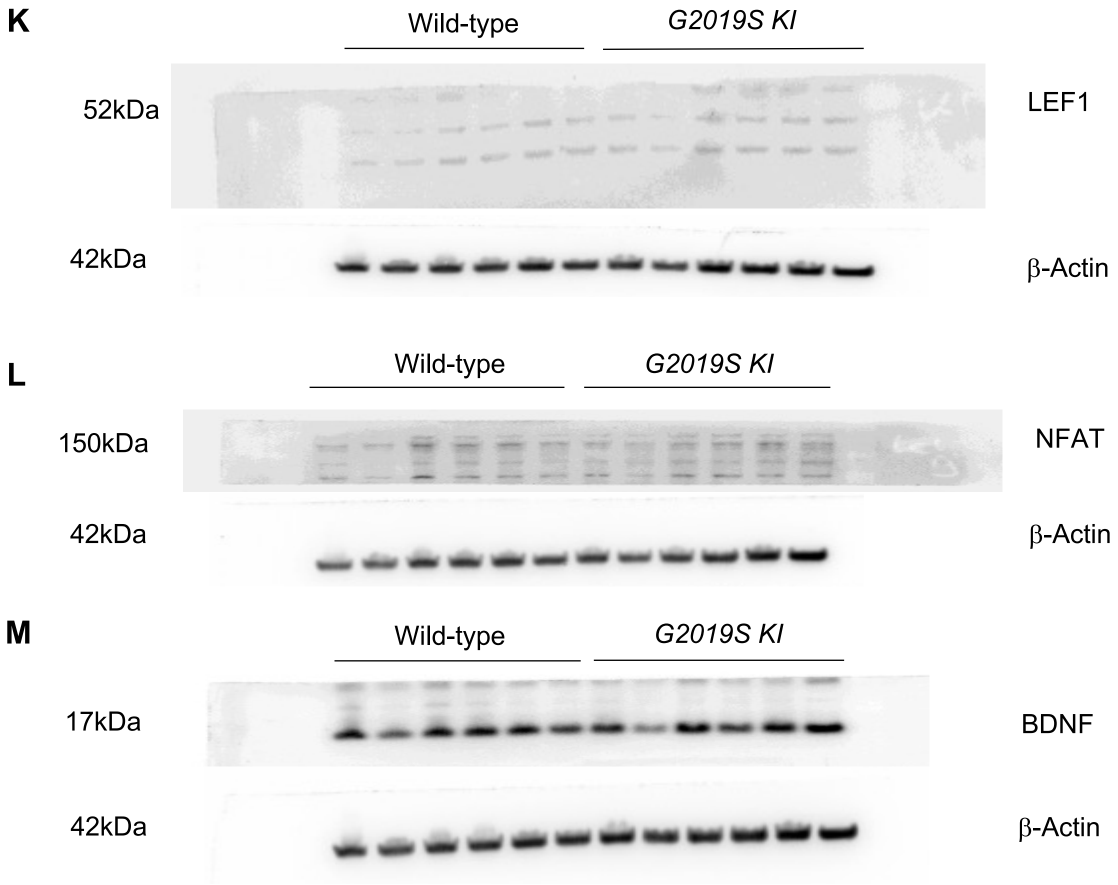
K
Wild-type
G2019S KI
52kDa
LEF1
42kDa
β-Actin
L
Wild-type
G2019S KI
150kDa
NFAT
42kDa
β-Actin
M
Wild-type
G2019S KI
17kDa
BDNF
42kDa
β-Actin

A

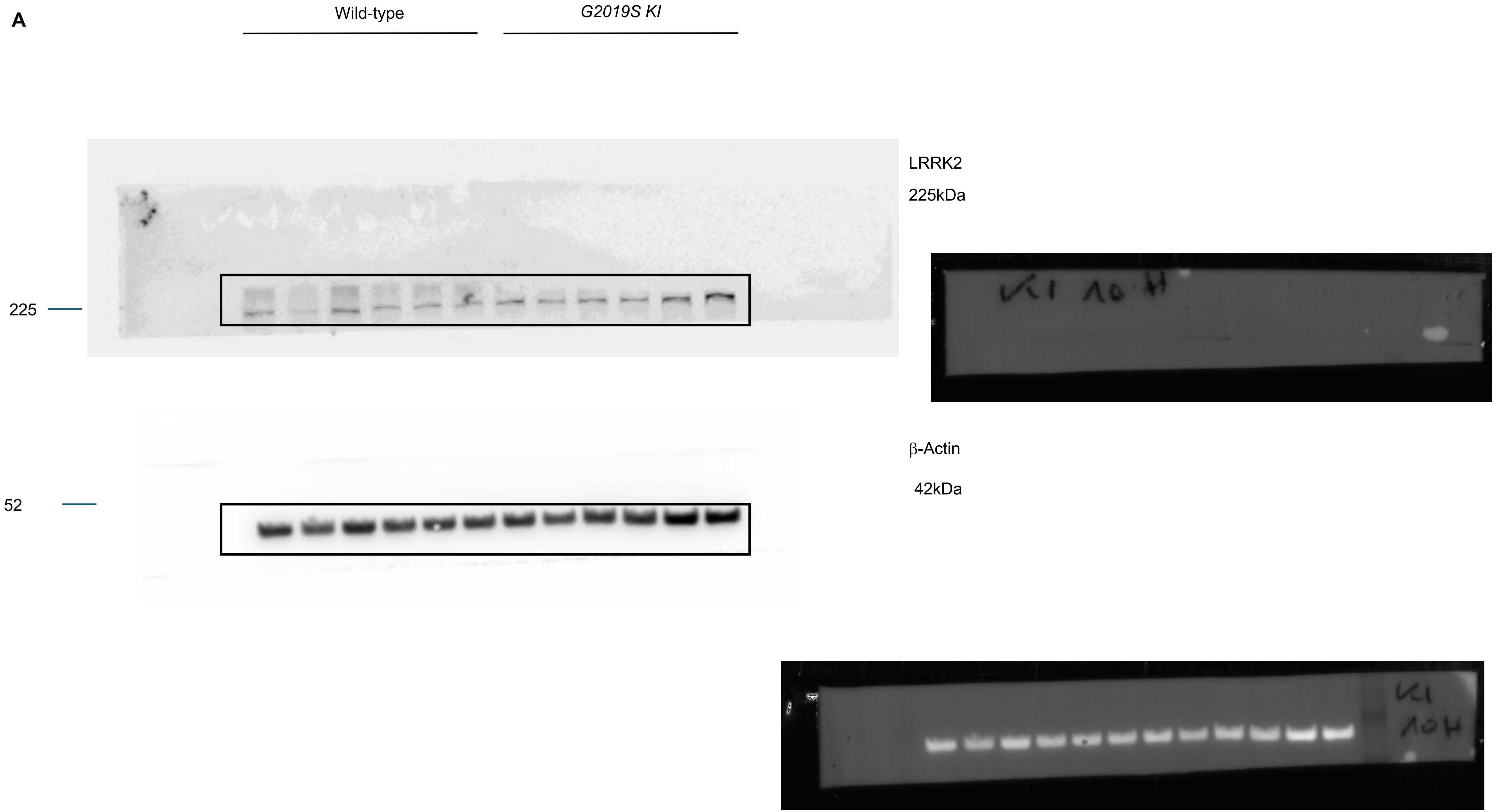

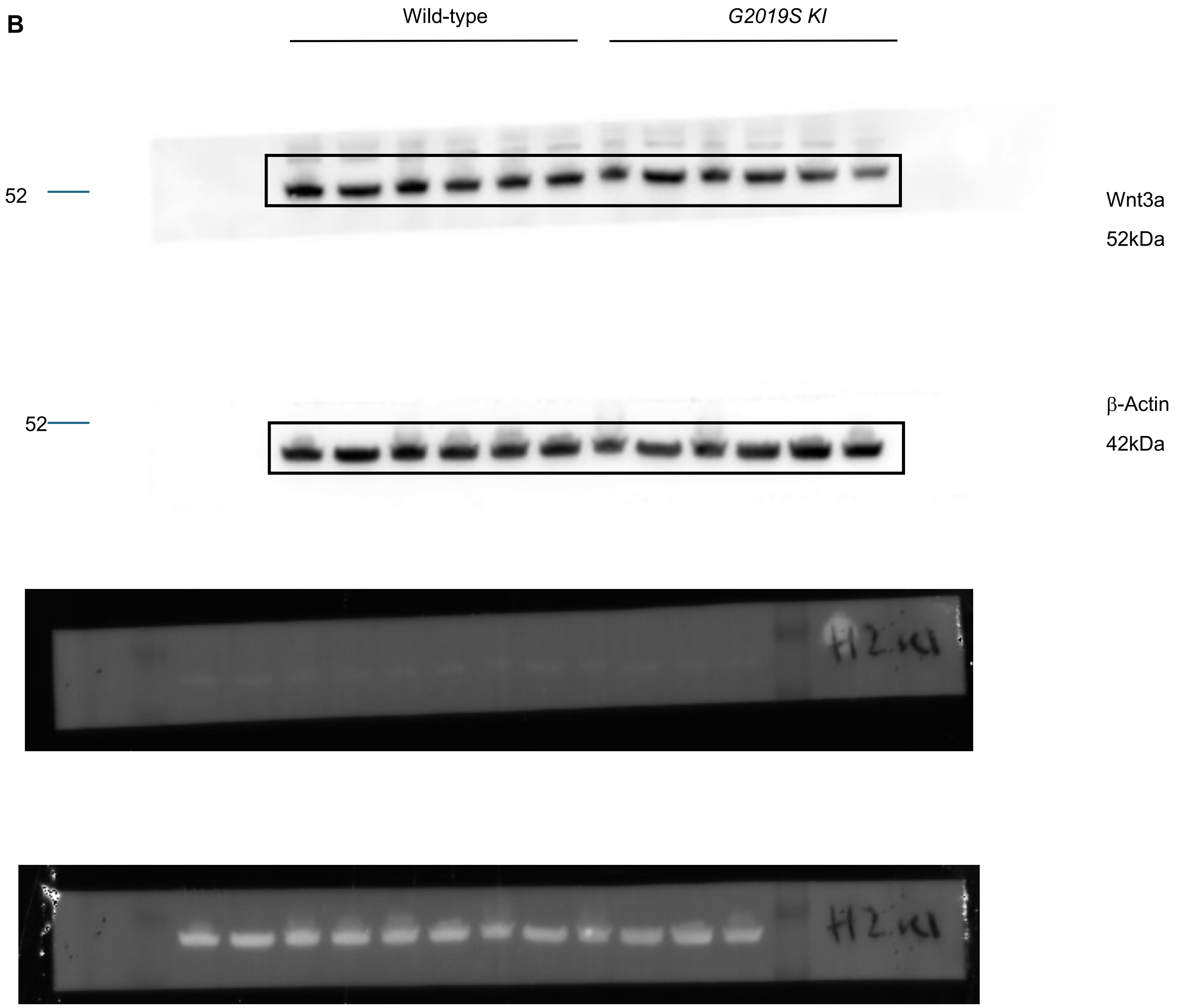
B
Wild-type
G2019S KI
52
Wnt3a
52kDa
52
β-Actin
42kDa

C

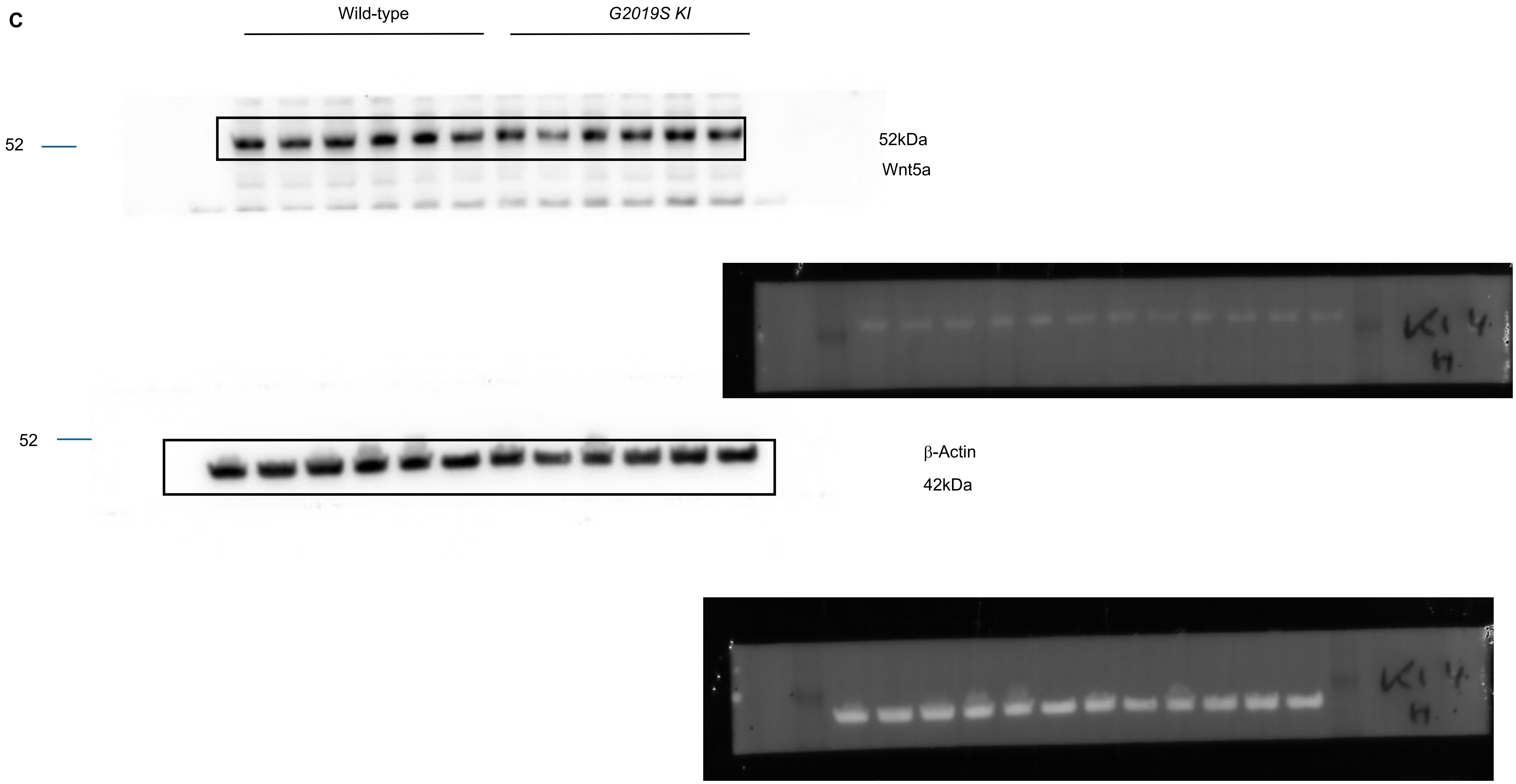

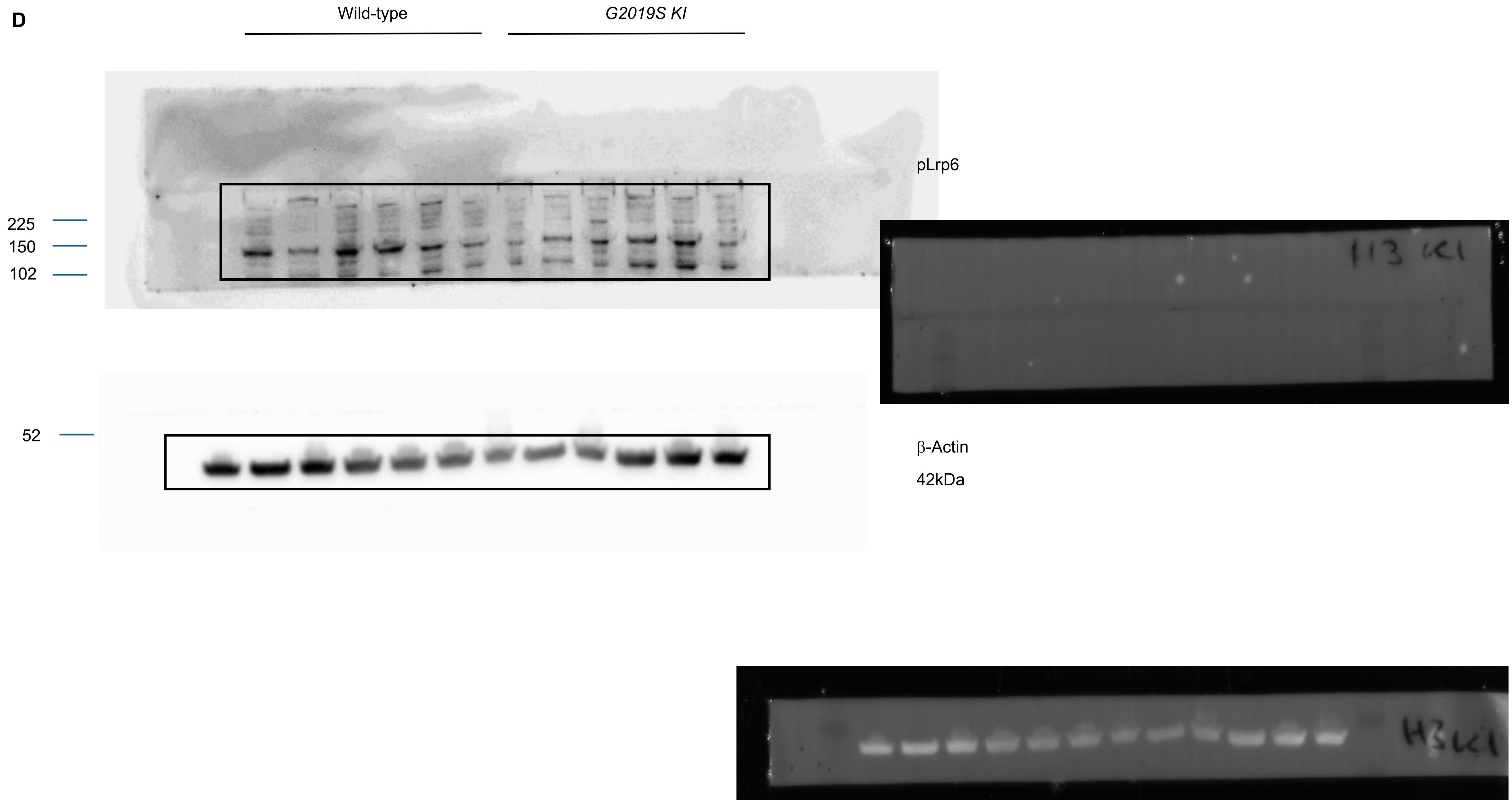
D
Wild-type
G2019S KI
225
150
102
pLrp6
52
β-Actin
42kDa

E

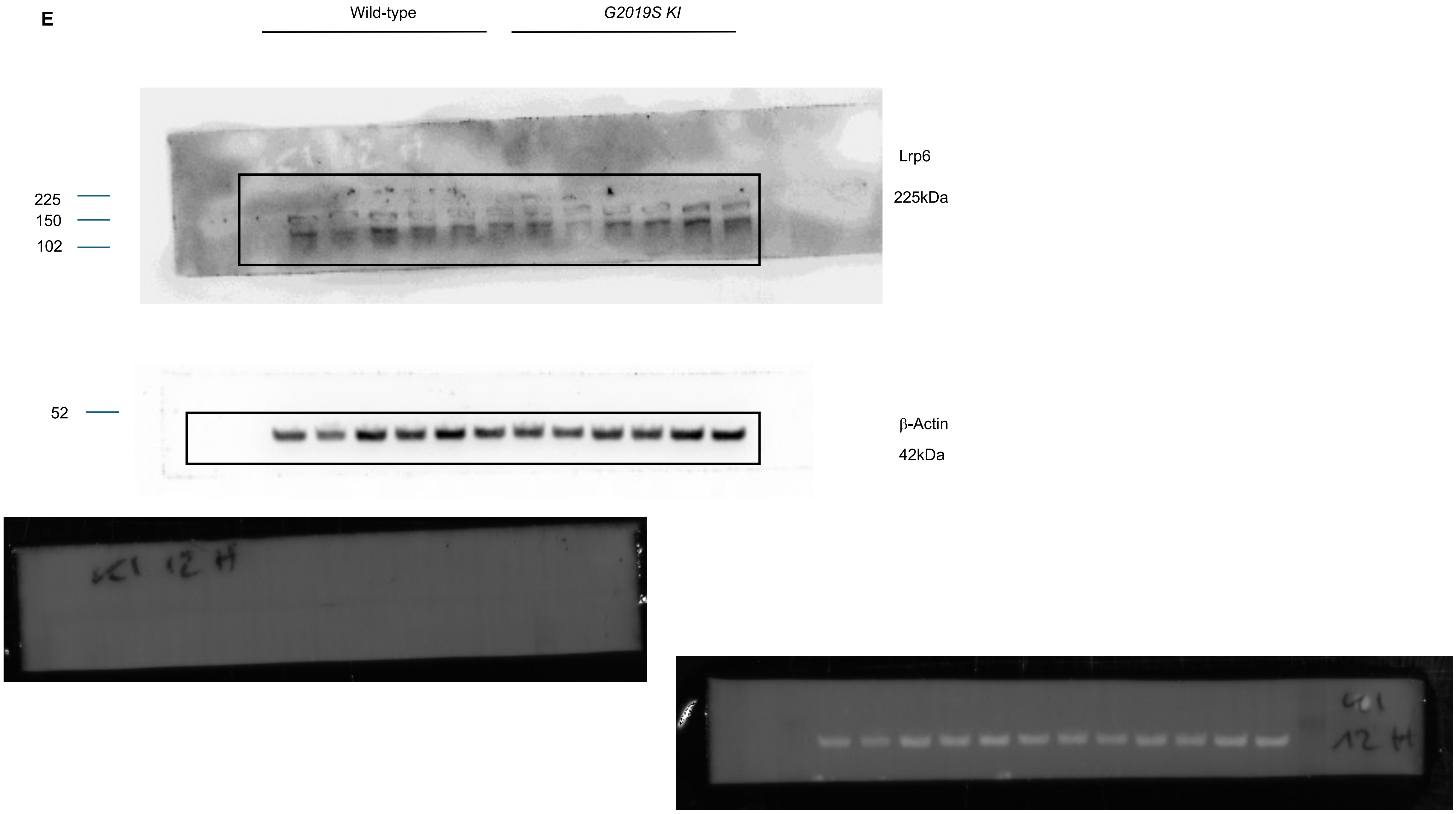

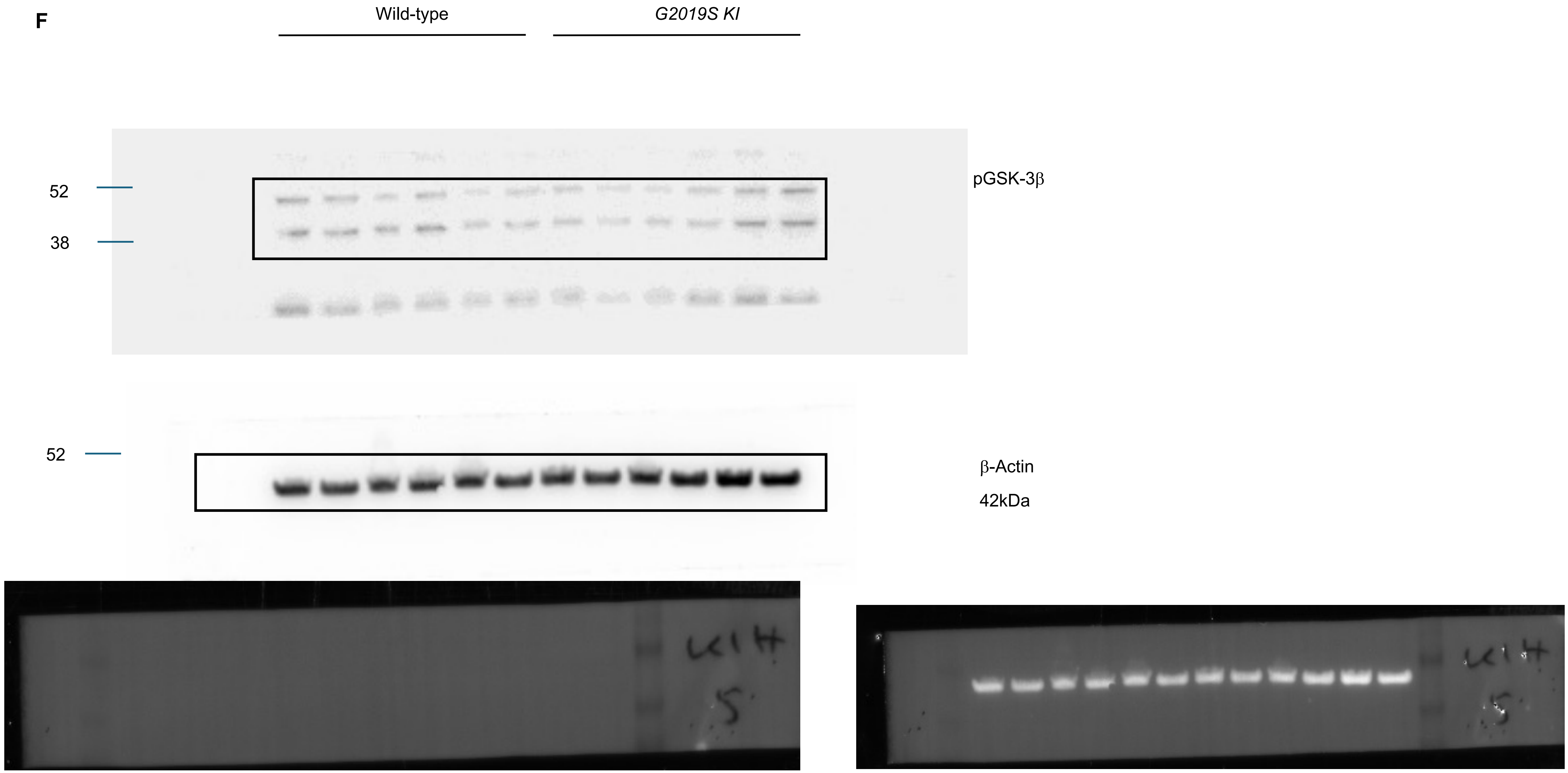
F
Wild-type
G2019S KI
52
38
pGSK-3β
52
β-Actin
42kDa

G

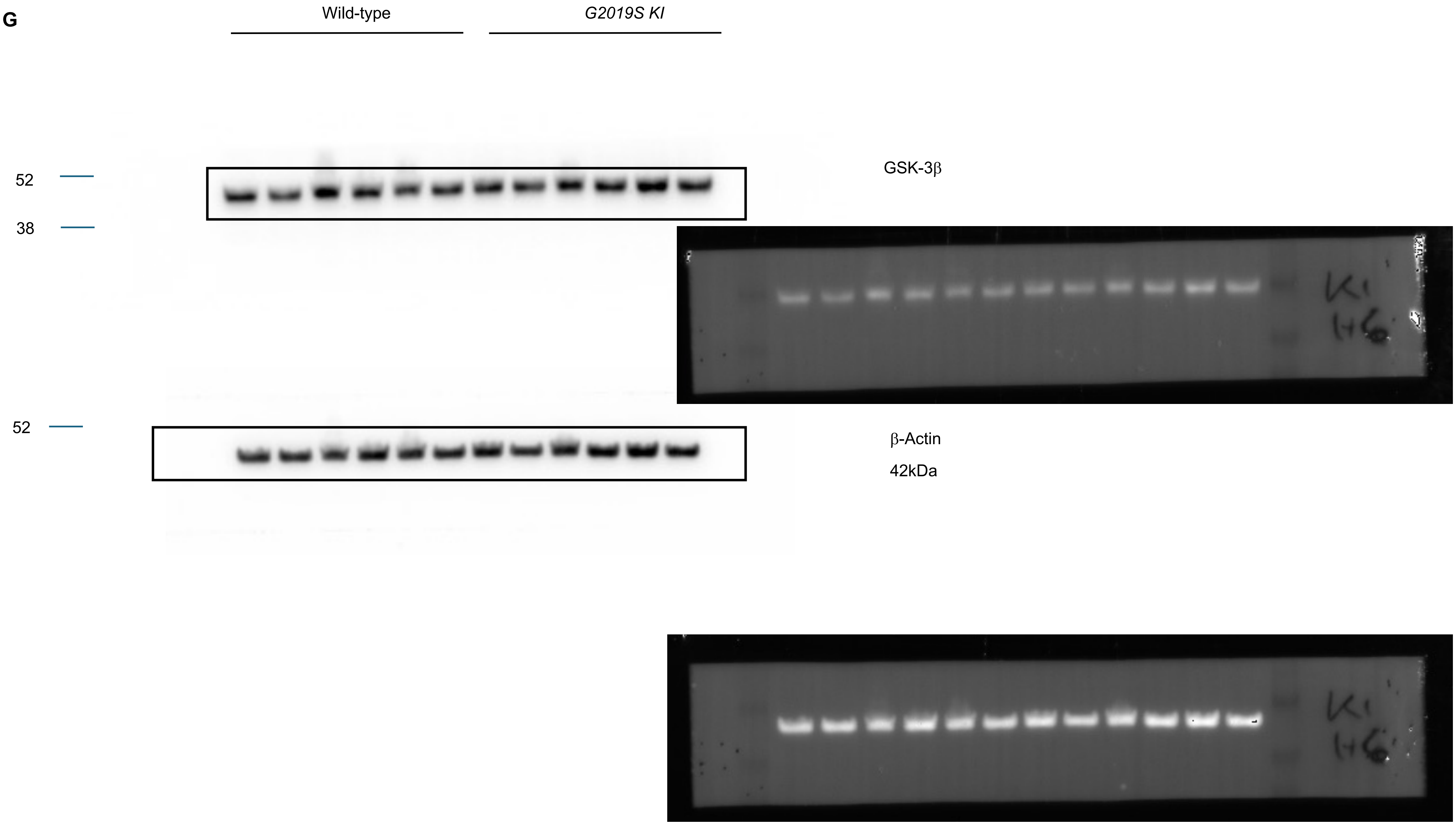

H

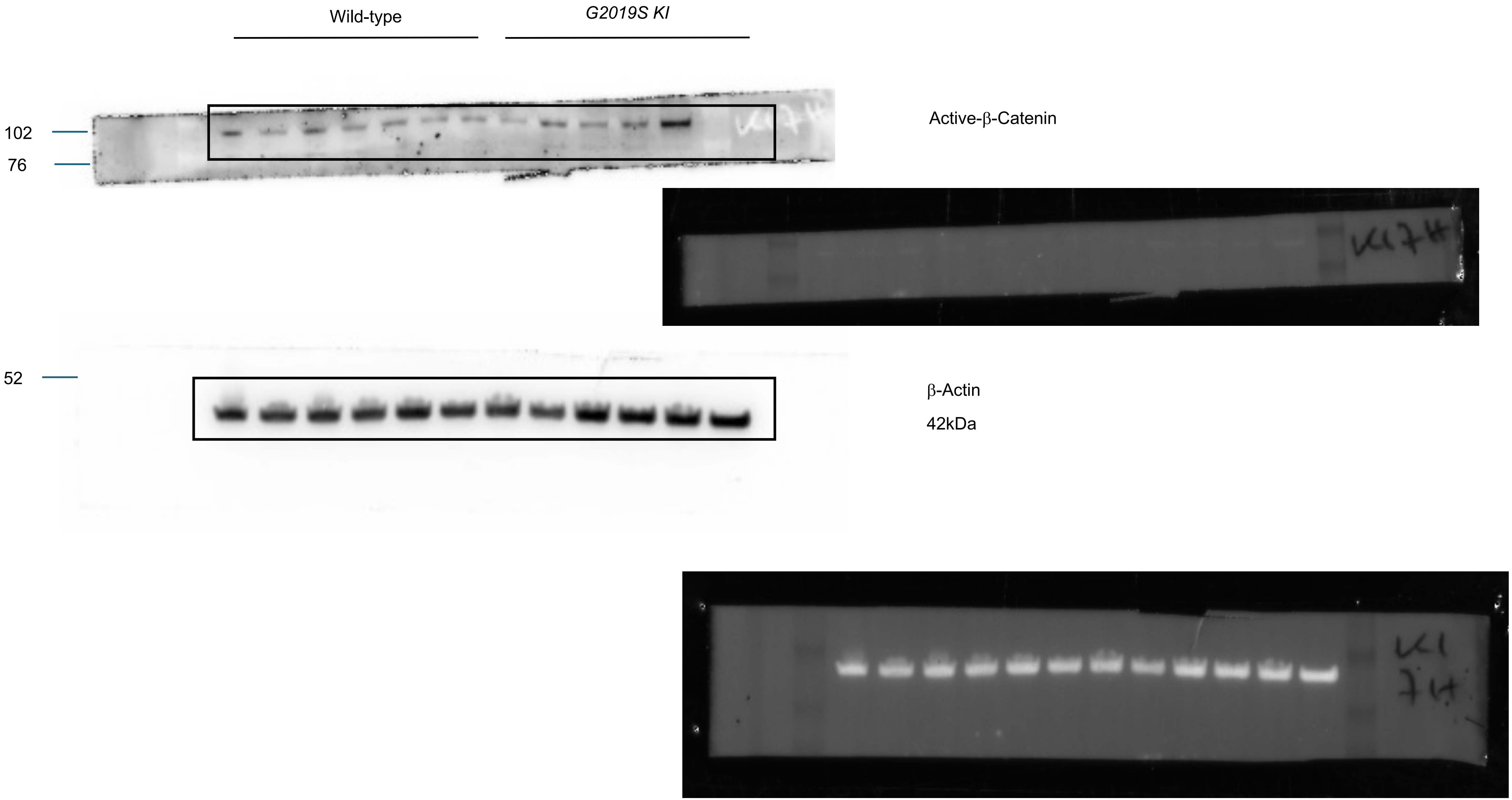

I

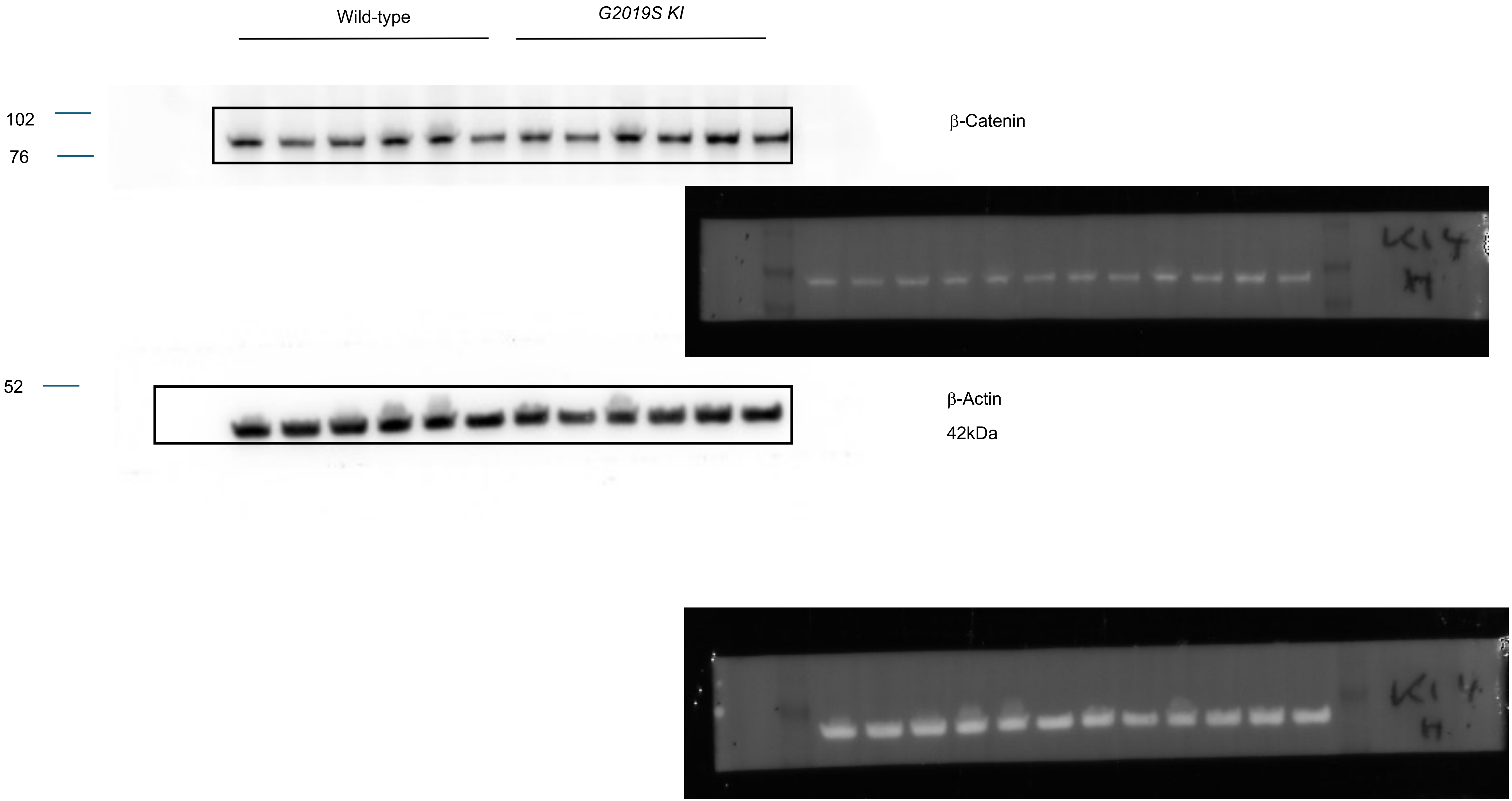

J

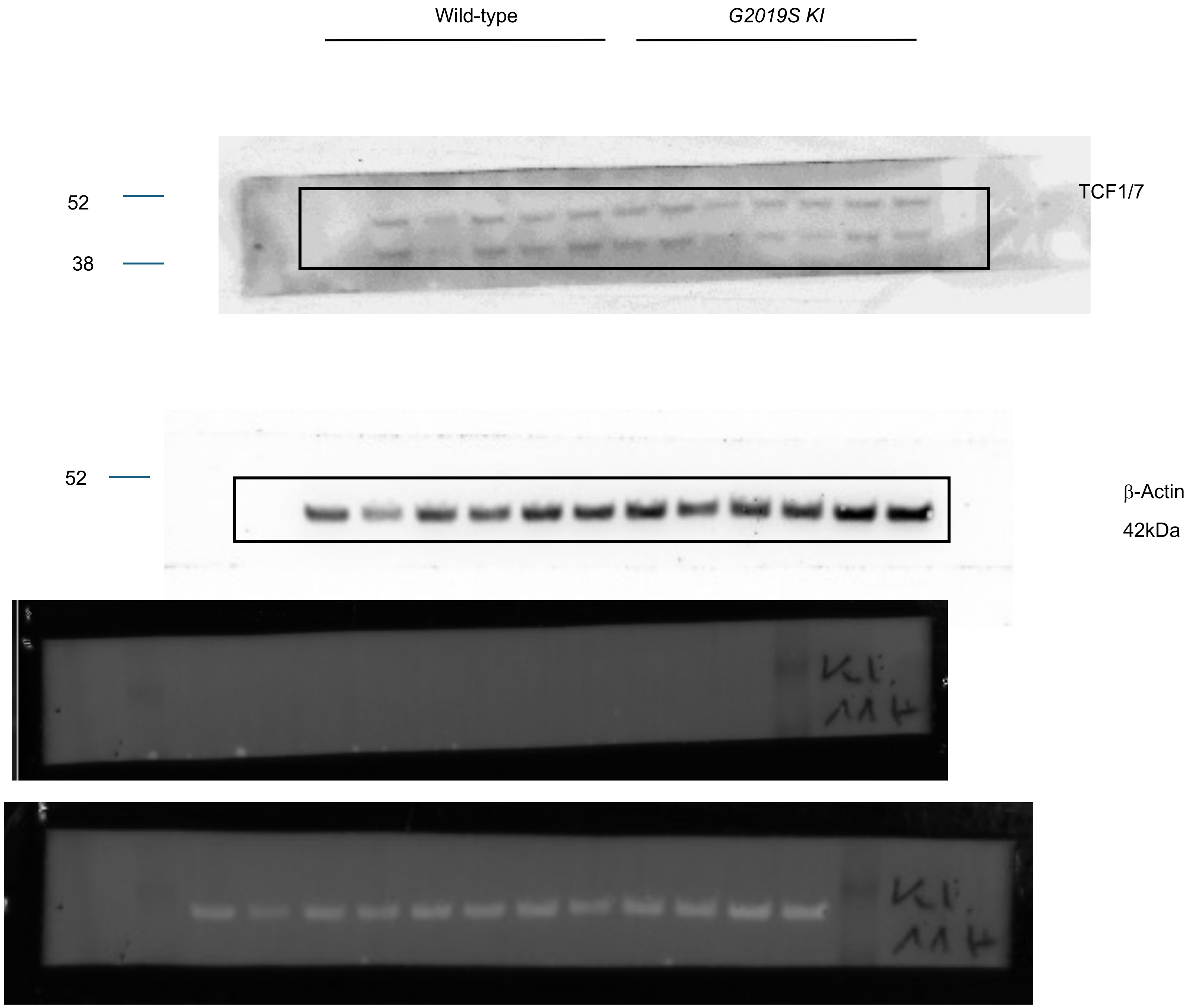

K

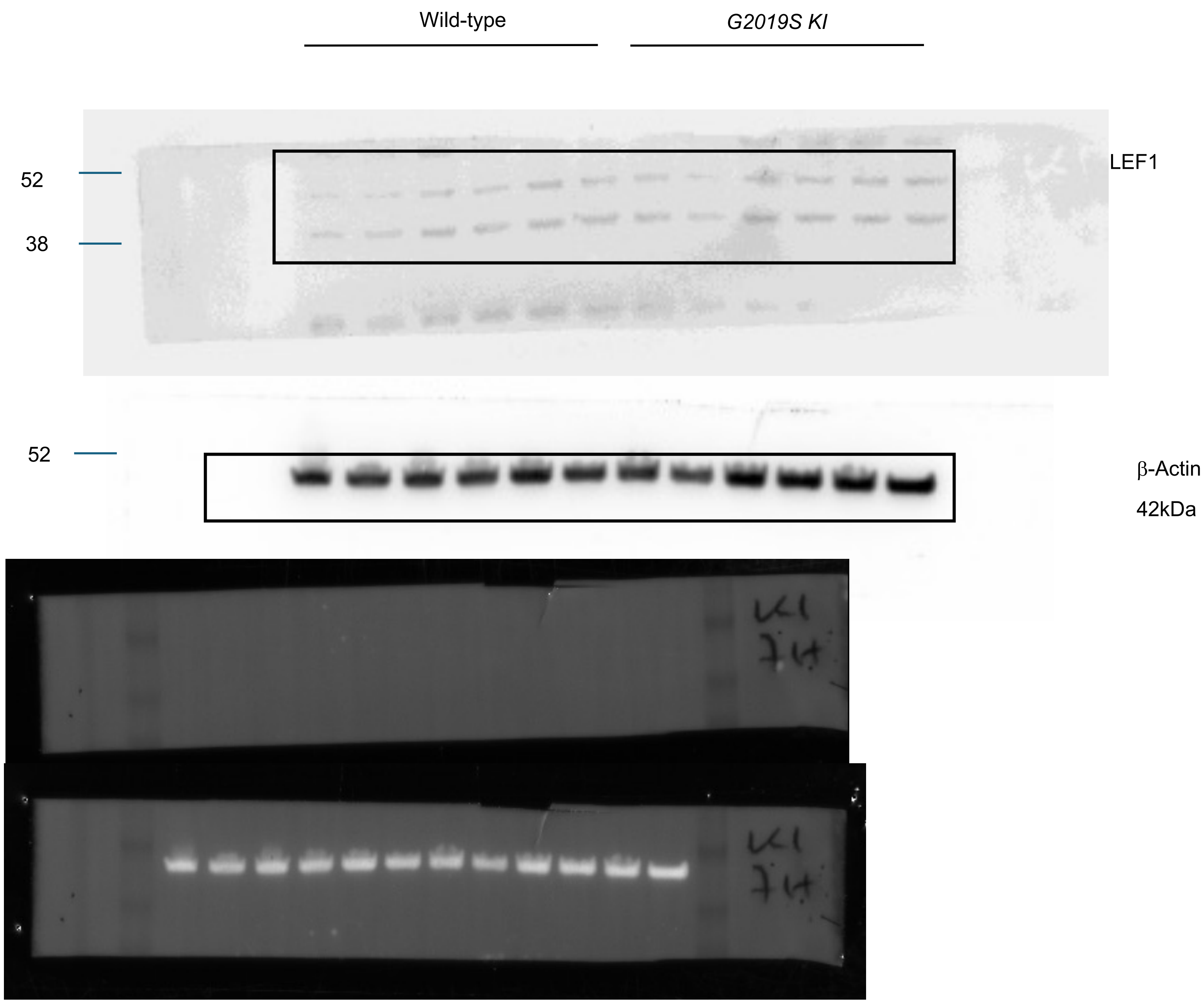

L

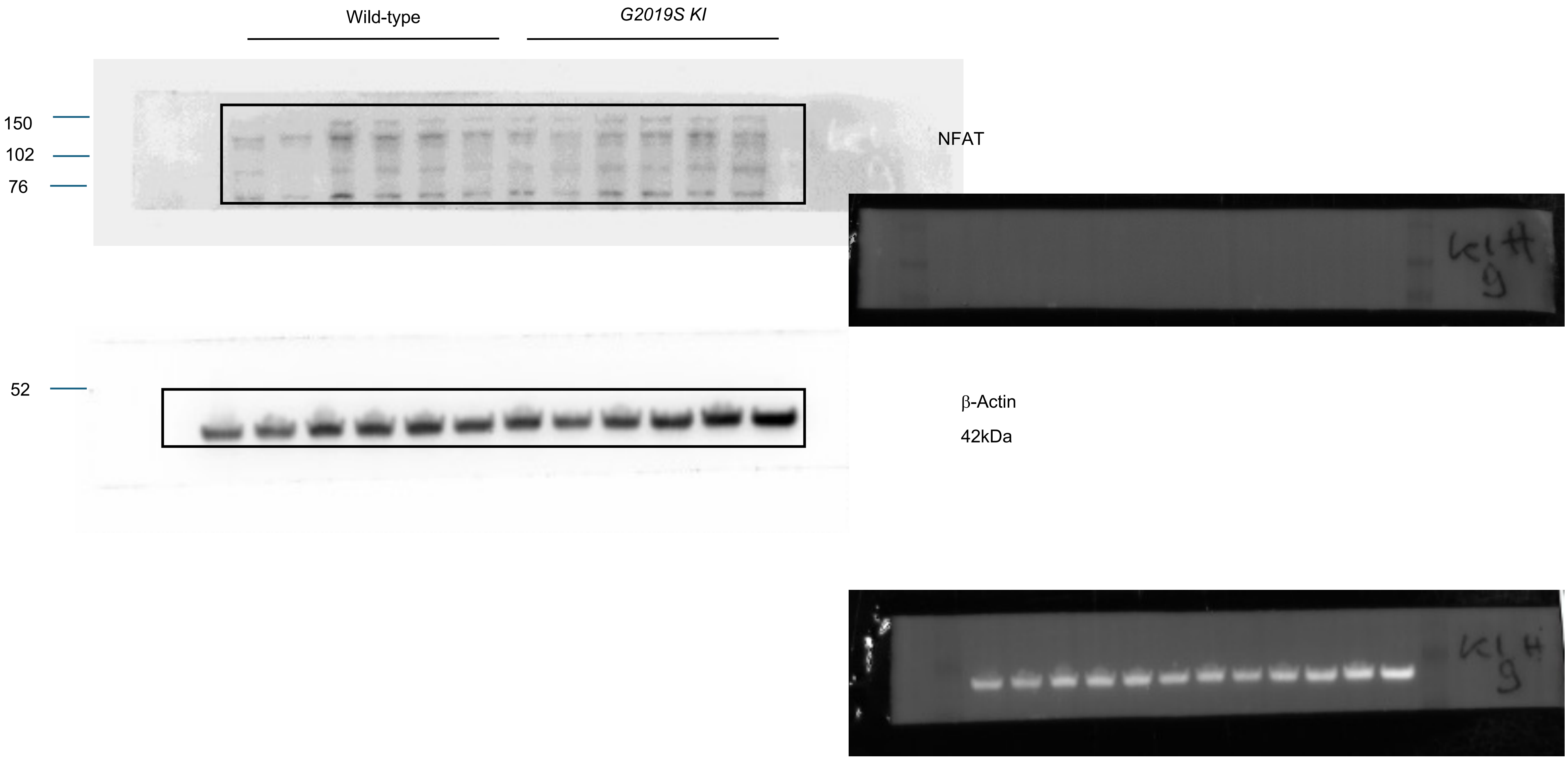

M

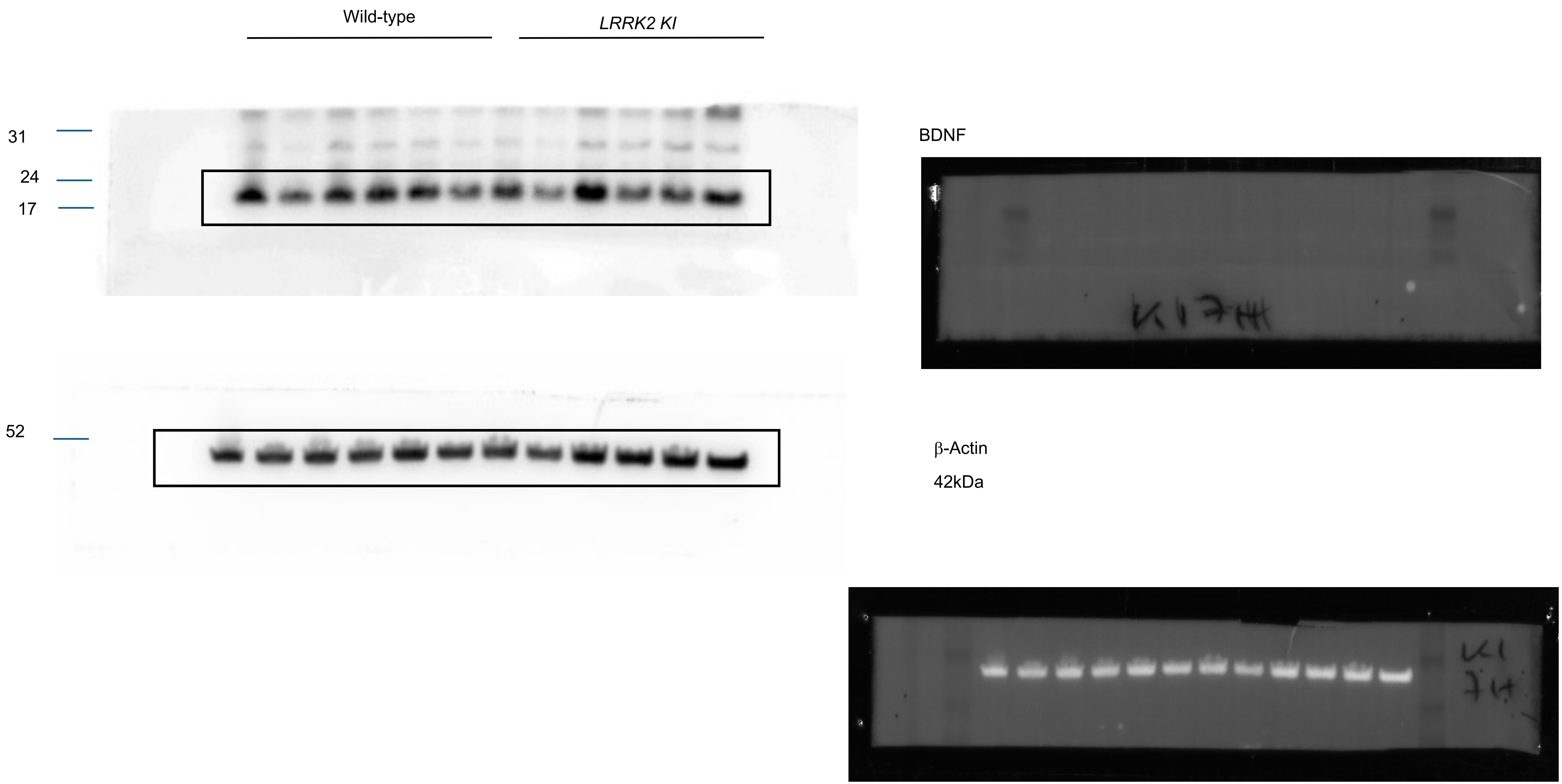

Supplement: Supplementary file 4 — Supplementary Figure 3. [file 41598_2024_63130_MOESM4_ESM.pdf]
