# Supplementary material for: Dysregulated Wnt and NFAT signaling in a Parkinson’s disease LRRK2 G2019S knock-in model
Source: Sci Rep. 2024 May 29;14:12393. doi: 10.1038/s41598-024-63130-8 (PMC11137013; doi:10.1038/s41598-024-63130-8)
Supplement: Supplementary file 5 — Supplementary Figure 4. [file 41598_2024_63130_MOESM5_ESM.pdf]

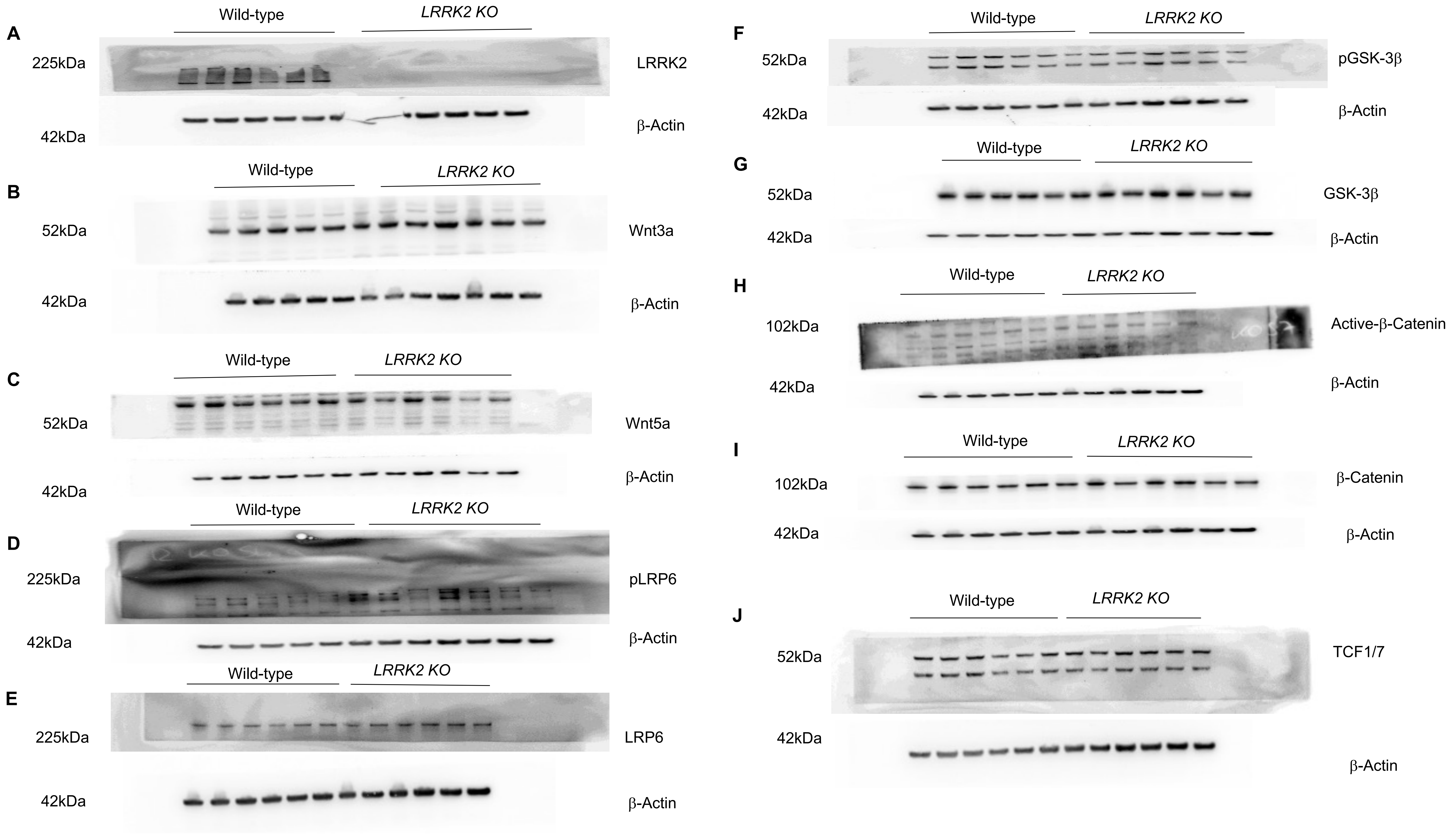
A
Wild-type
LRRK2 KO
225kDa
LRRK2
42kDa
β-Actin
B
Wild-type
LRRK2 KO
52kDa
Wnt3a
42kDa
β-Actin
C
Wild-type
LRRK2 KO
52kDa
Wnt5a
42kDa
β-Actin
D
Wild-type
LRRK2 KO
225kDa
pLRP6
42kDa
β-Actin
E
Wild-type
LRRK2 KO
225kDa
LRP6
42kDa
β-Actin
F
Wild-type
LRRK2 KO
52kDa
pGSK-3β
42kDa
β-Actin
G
Wild-type
LRRK2 KO
52kDa
GSK-3β
42kDa
β-Actin
H
Wild-type
LRRK2 KO
102kDa
Active-β-Catenin
42kDa
β-Actin
I
Wild-type
LRRK2 KO
102kDa
β-Catenin
42kDa
β-Actin
J
Wild-type
LRRK2 KO
52kDa
TCF1/7
42kDa
β-Actin

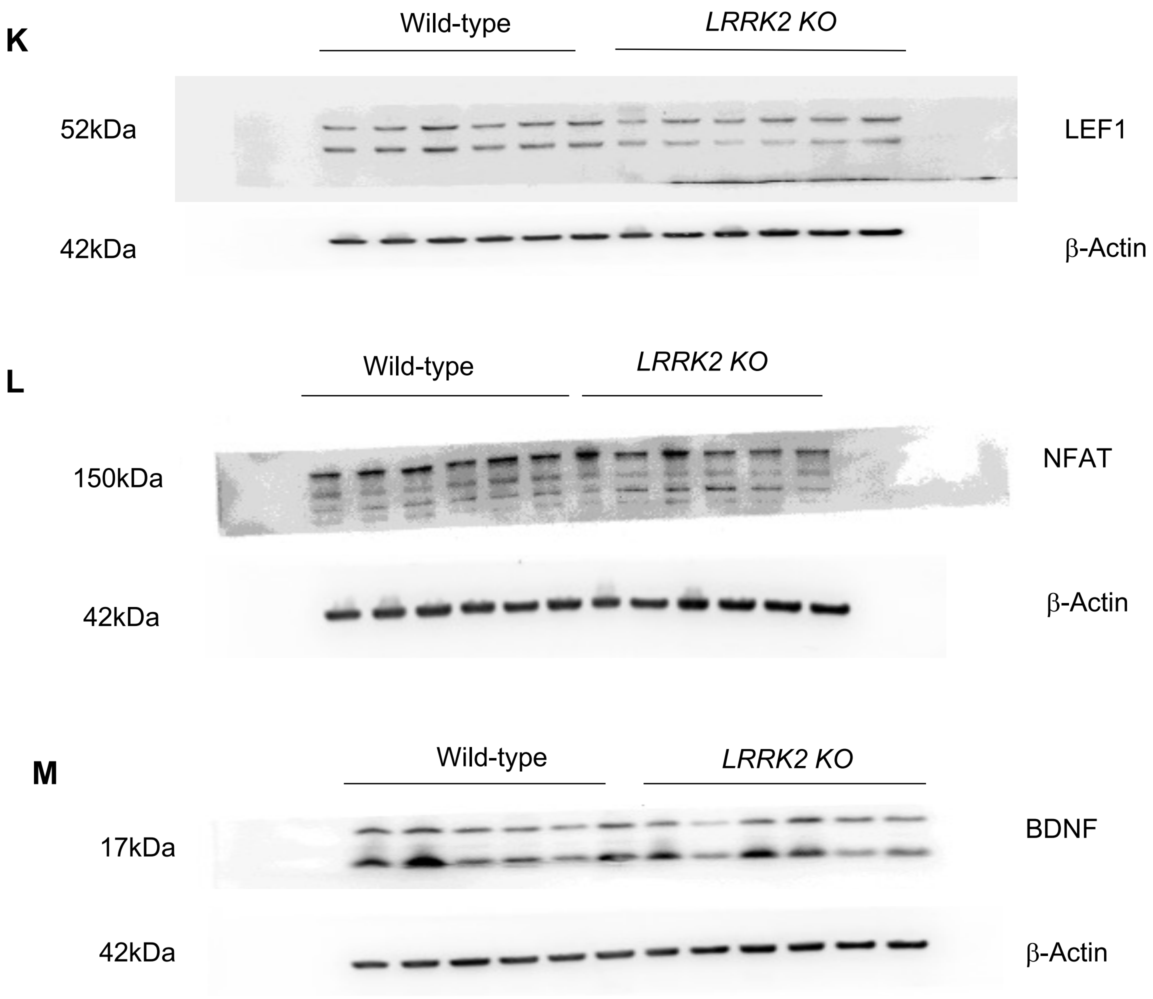
K
Wild-type
LRRK2 KO
52kDa
LEF1
42kDa
β-Actin
L
Wild-type
LRRK2 KO
150kDa
NFAT
42kDa
β-Actin
M
Wild-type
LRRK2 KO
17kDa
BDNF
42kDa
β-Actin

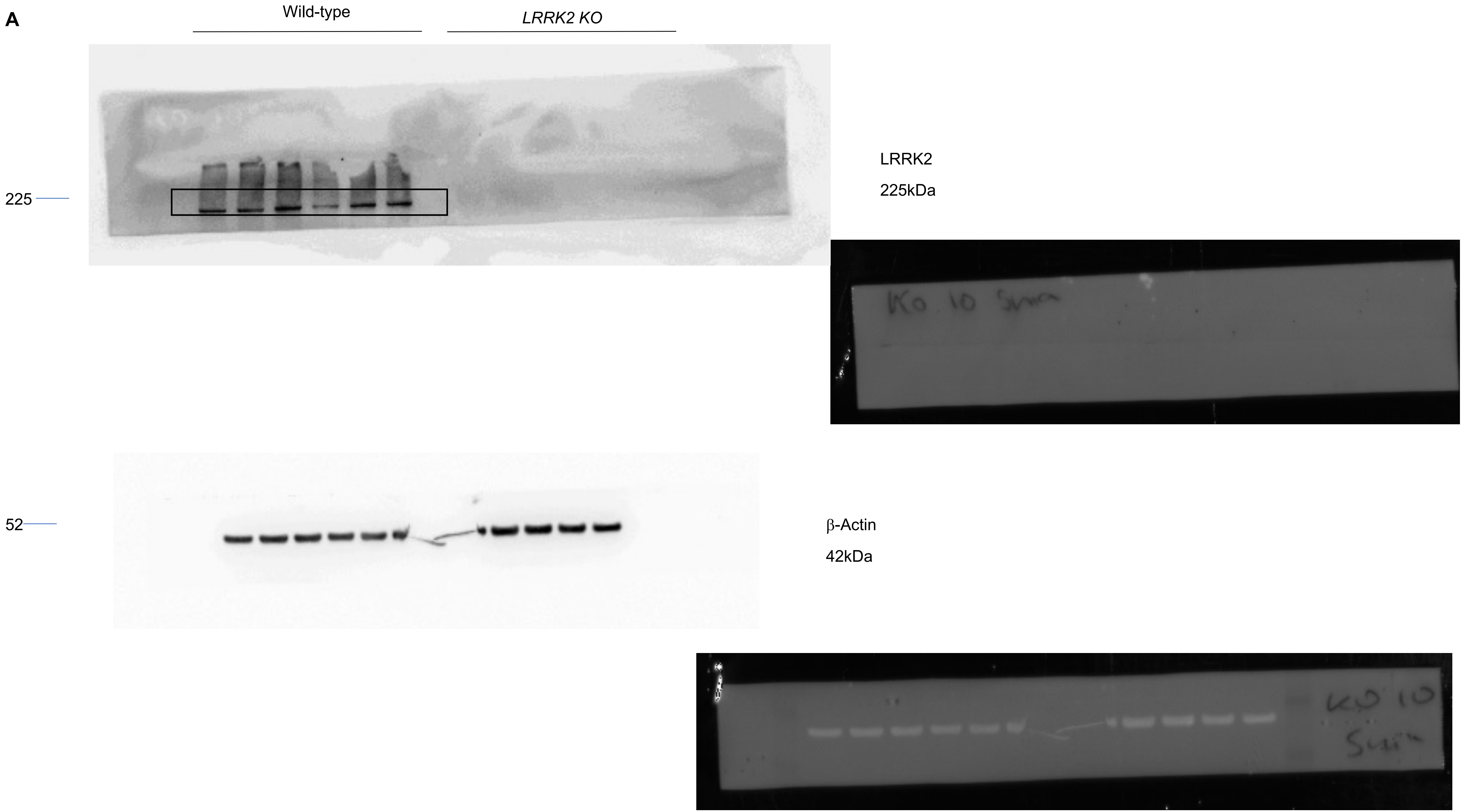
A
Wild-type
LRRK2 KO
225
LRRK2
225kDa
52
β-Actin
42kDa

B

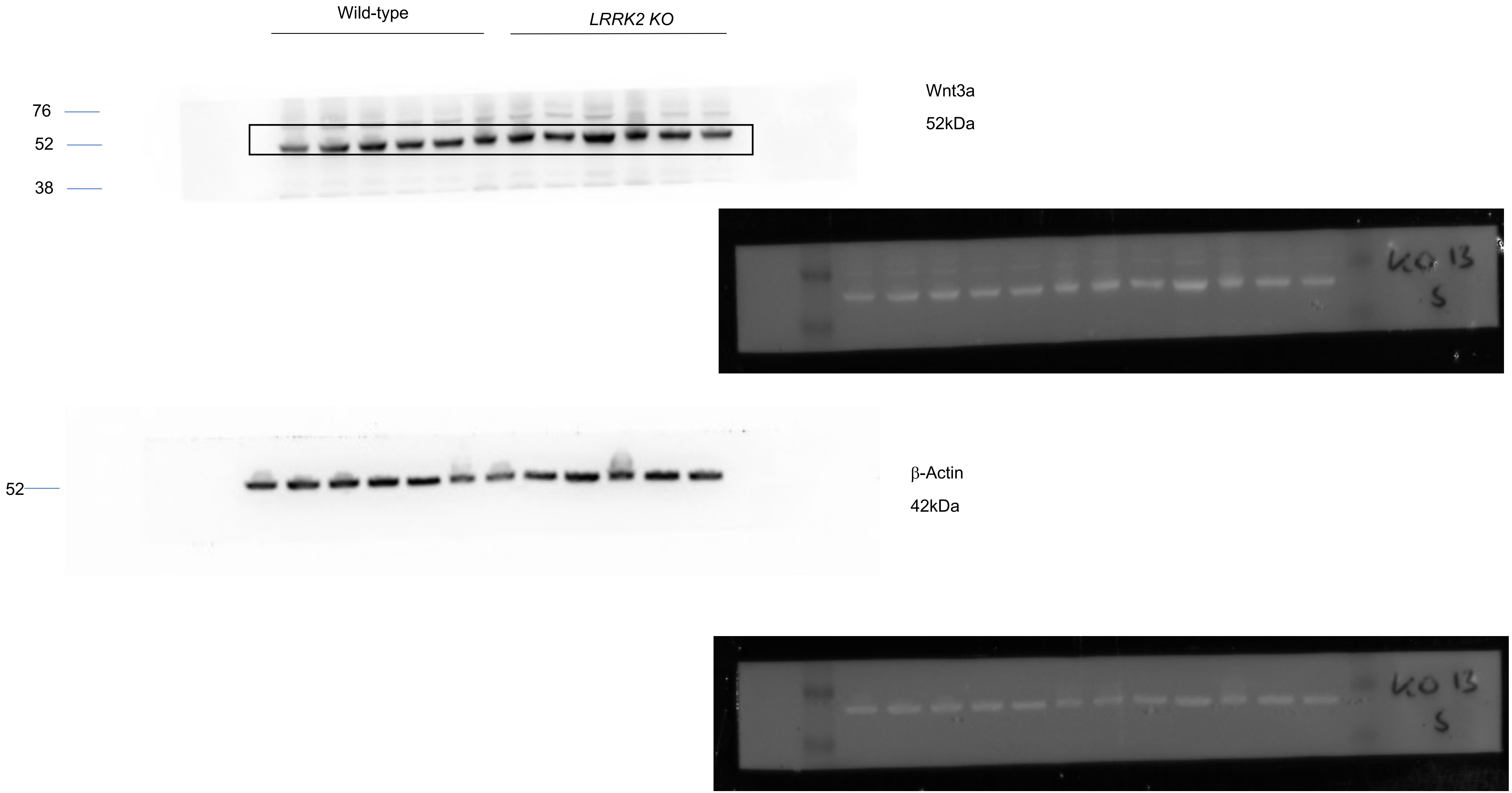
Wild-type
LRRK2 KO
76
52
38
Wnt3a
52kDa
KO 13
S
52
β-Actin
42kDa
KO 13
S

C

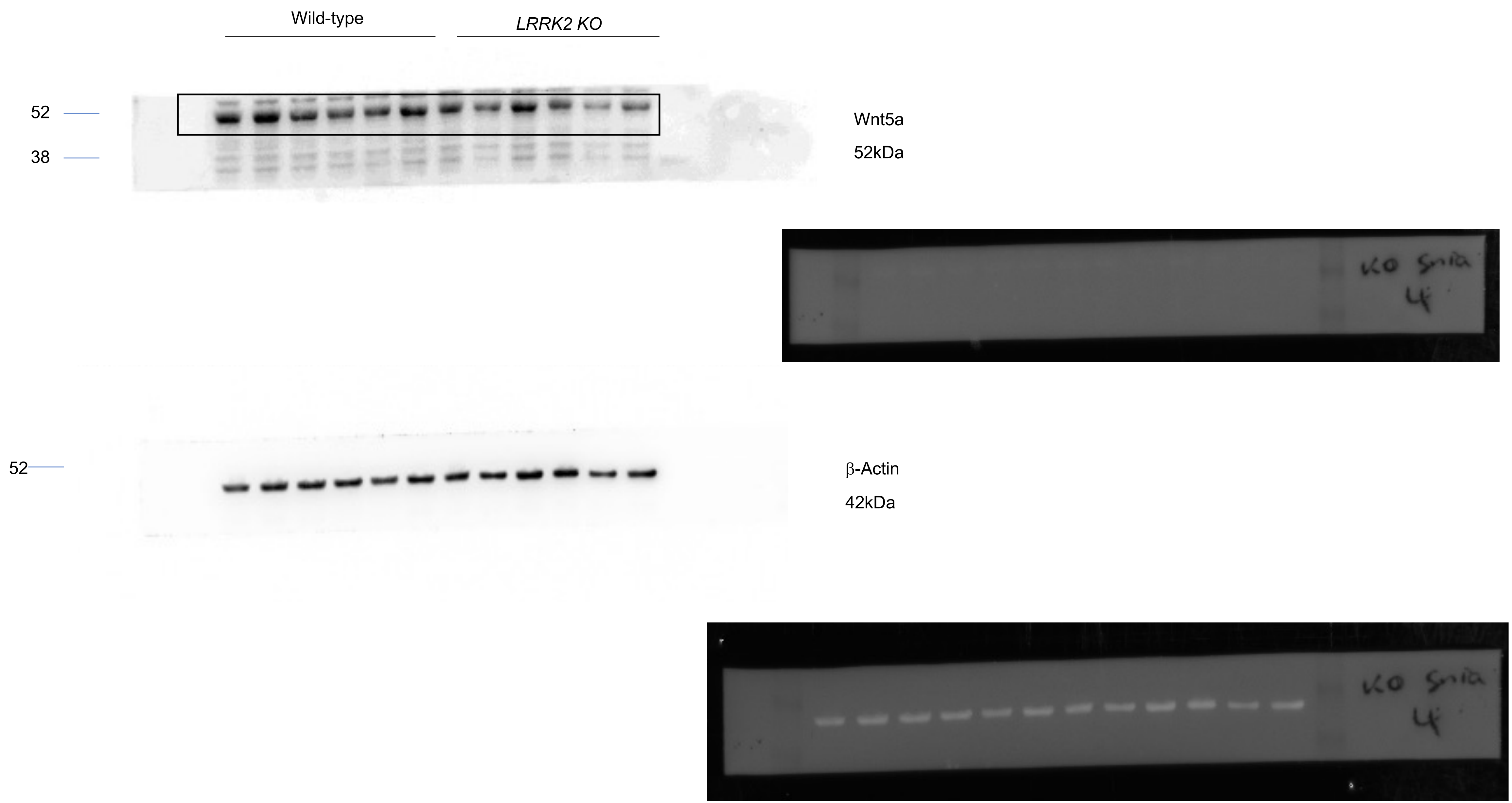

D

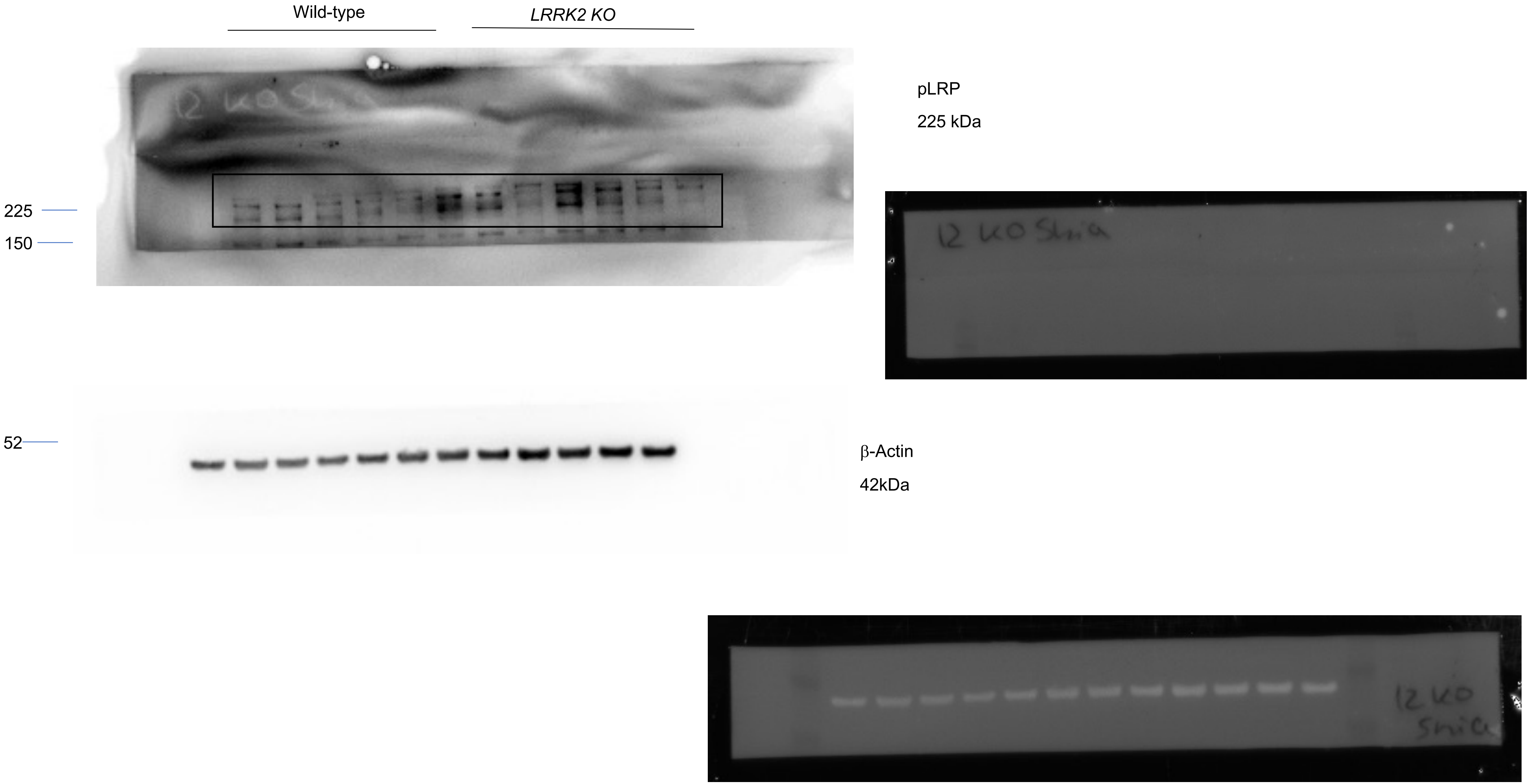

E

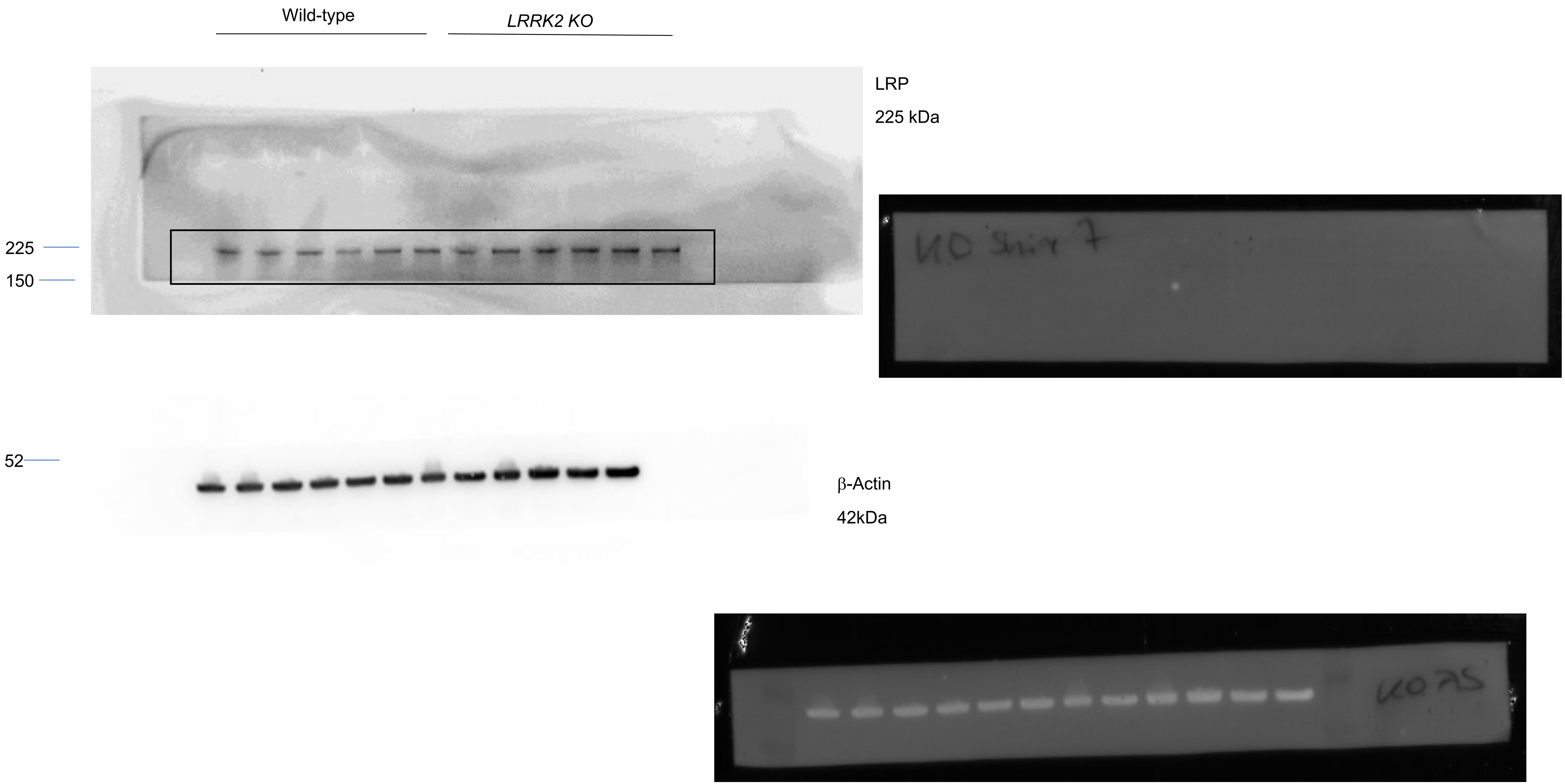

F

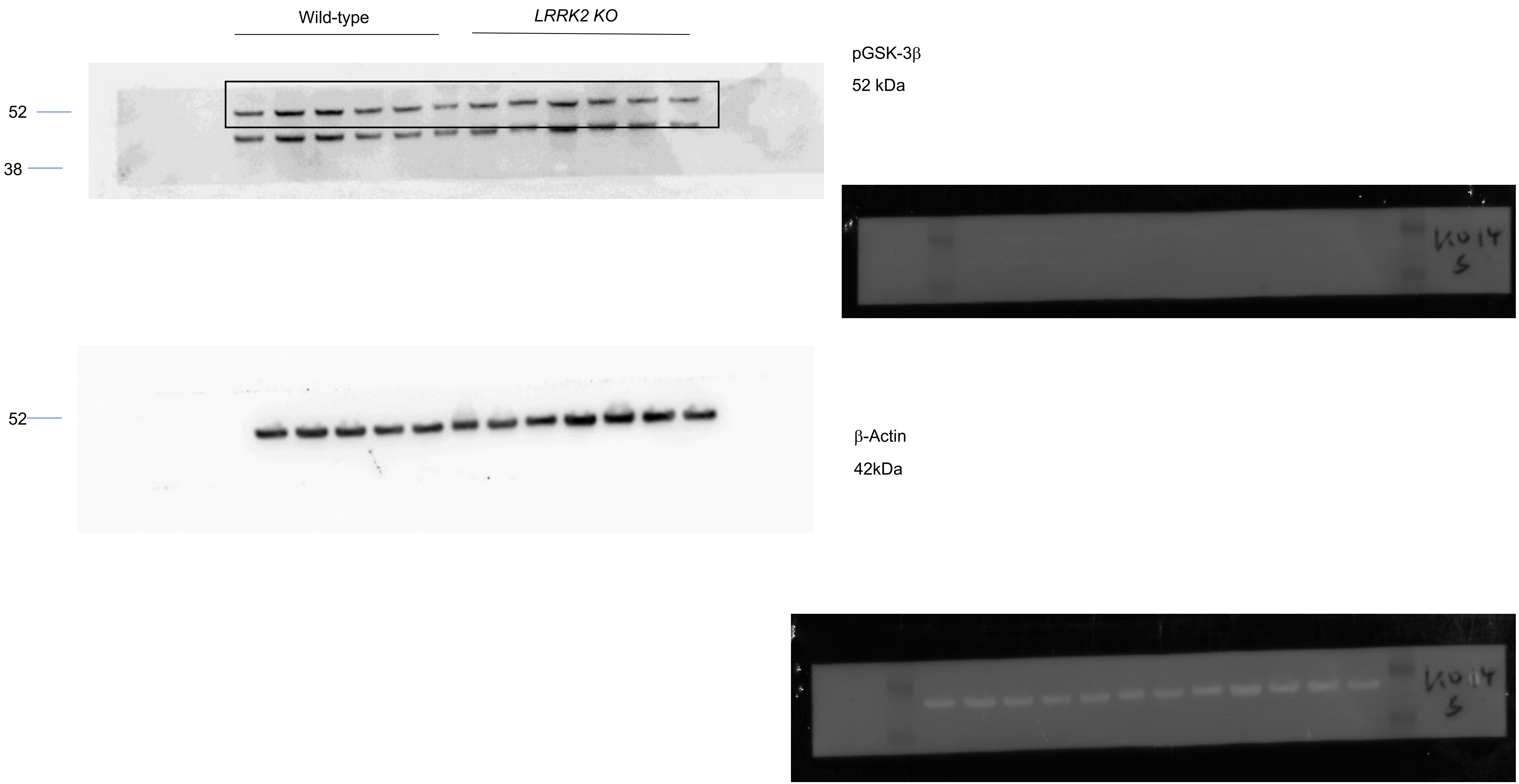

G

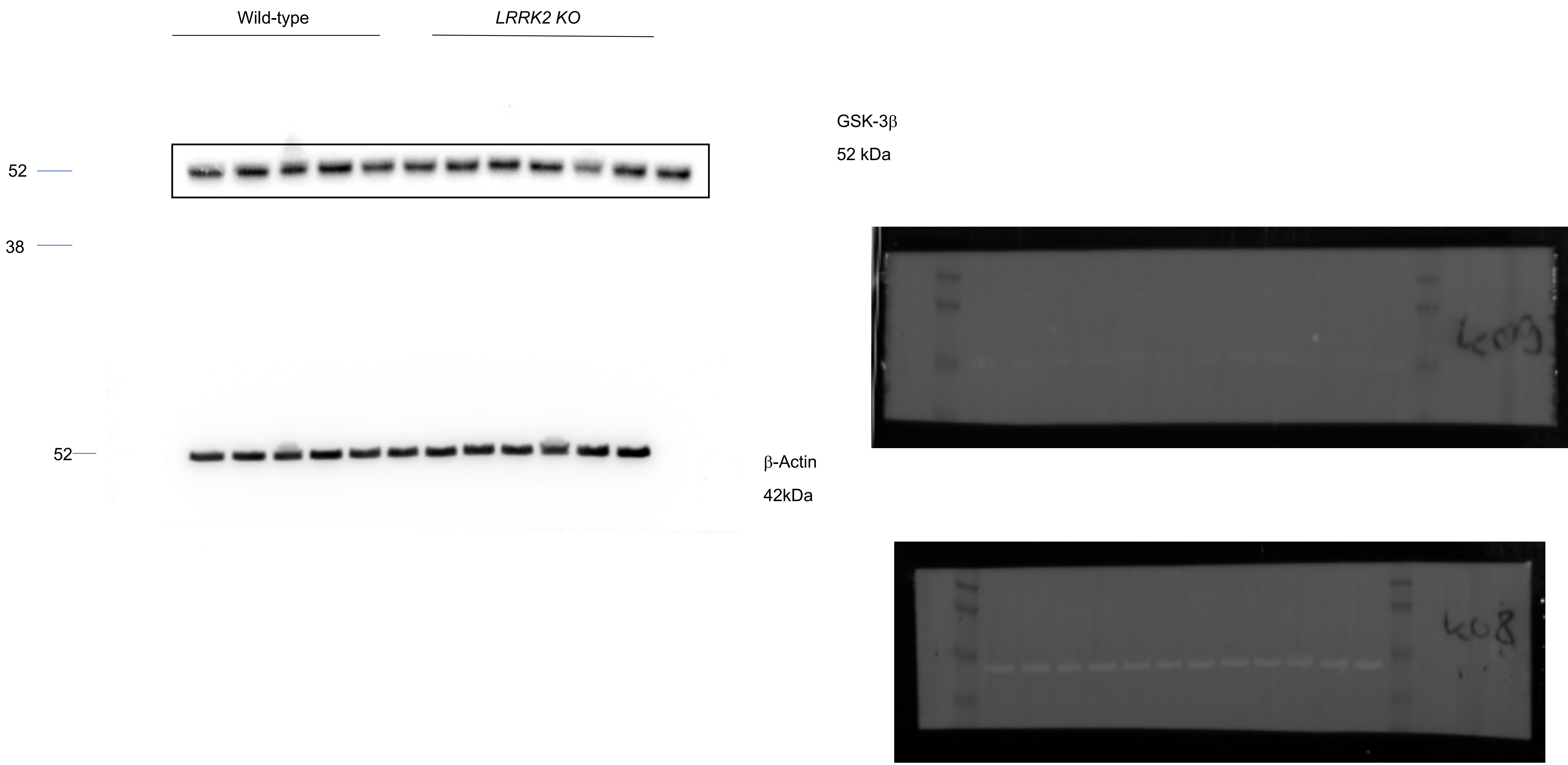

H

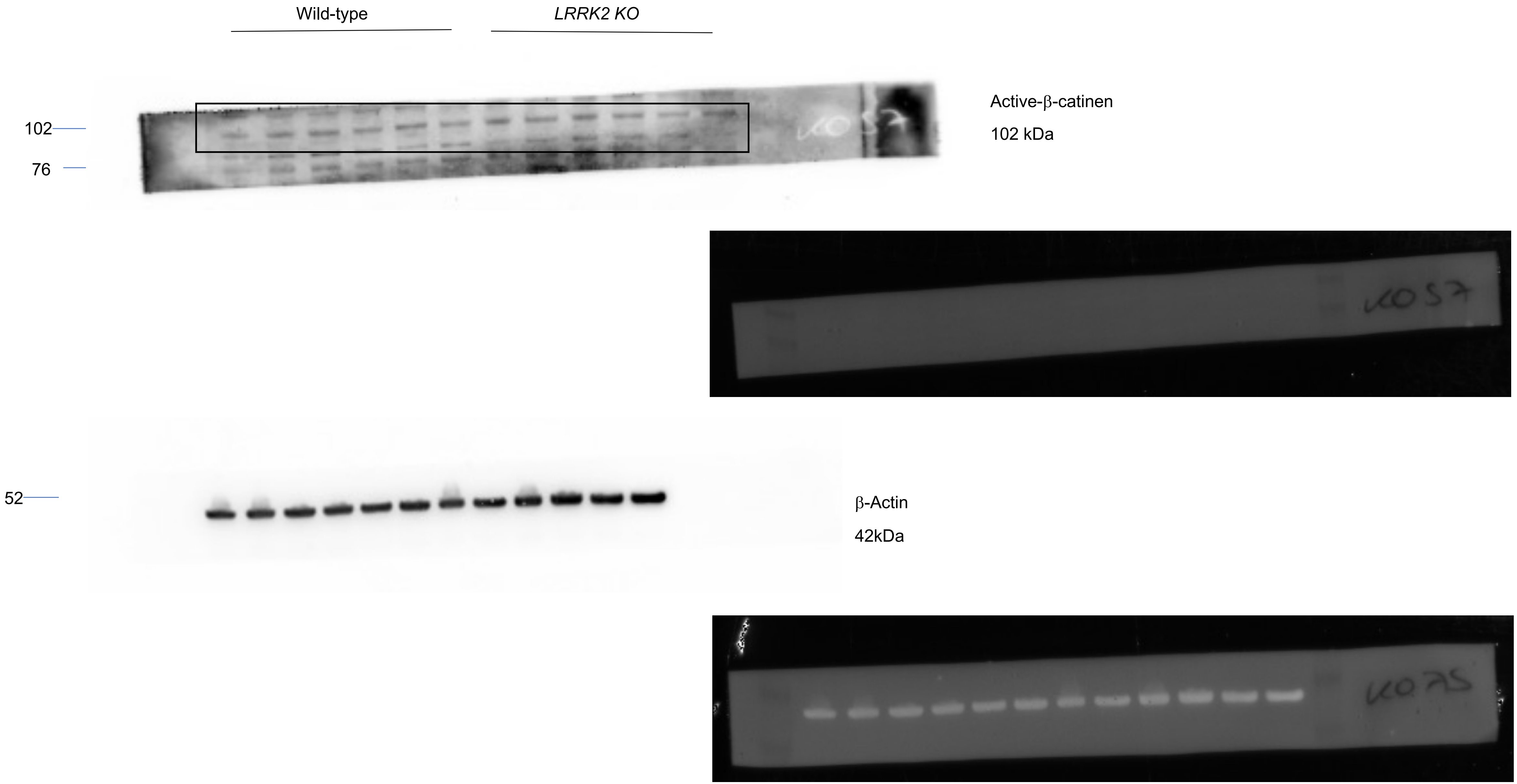

Wild-type
LRRK2 KO
102
76
Active-β-catinen
102 kDa
52
β-Actin
42kDa

I

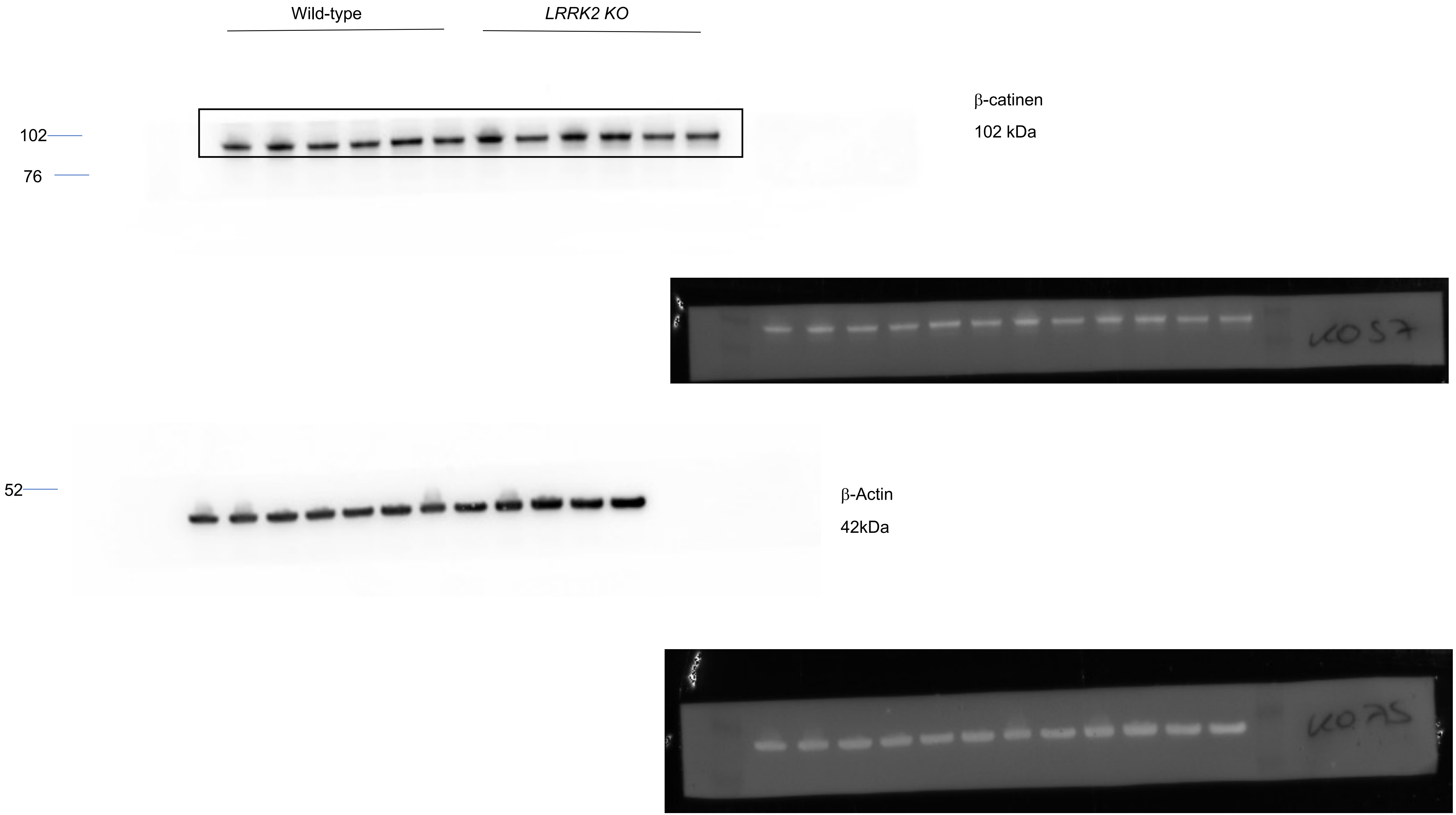

J

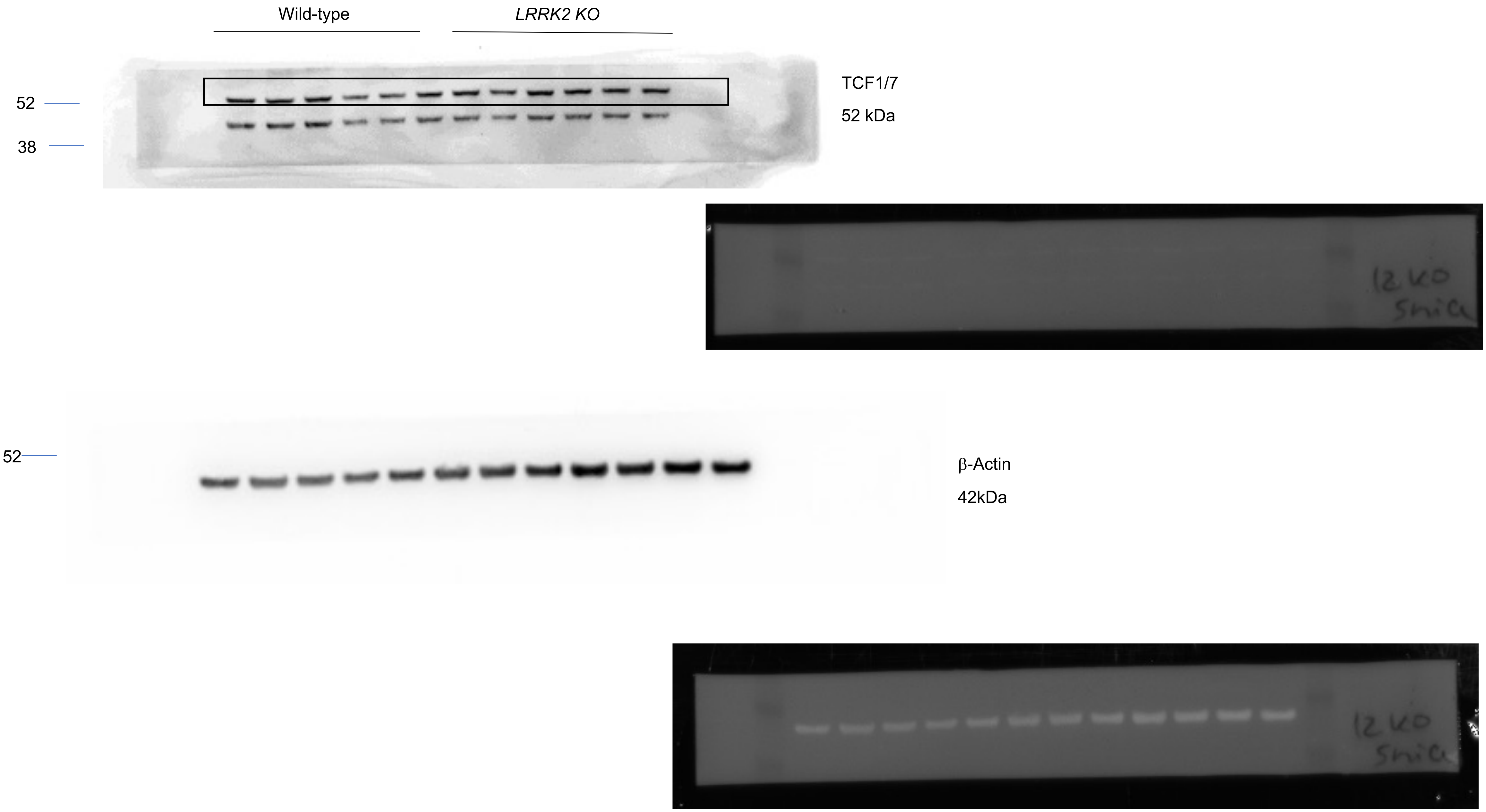

K

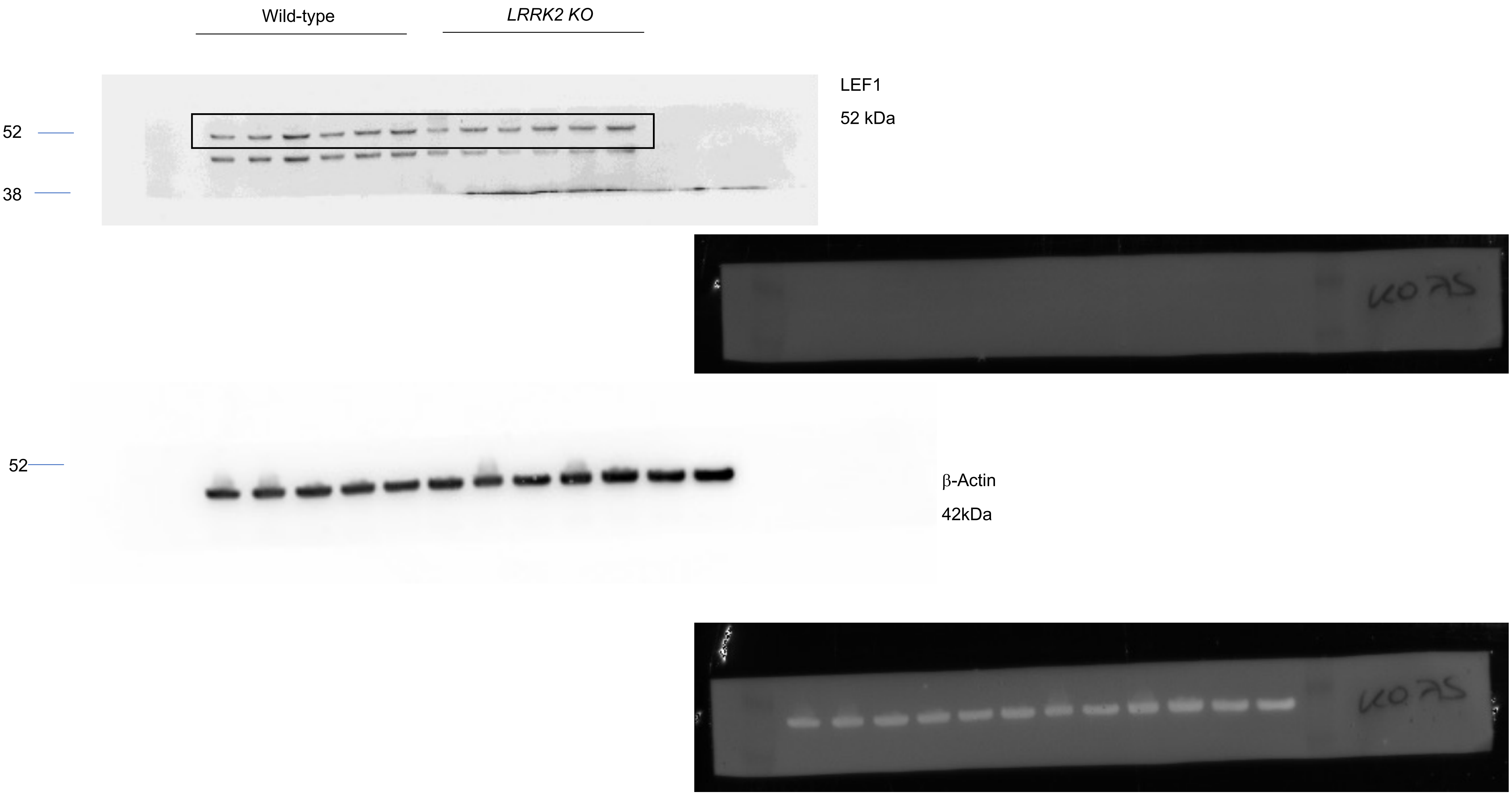

L

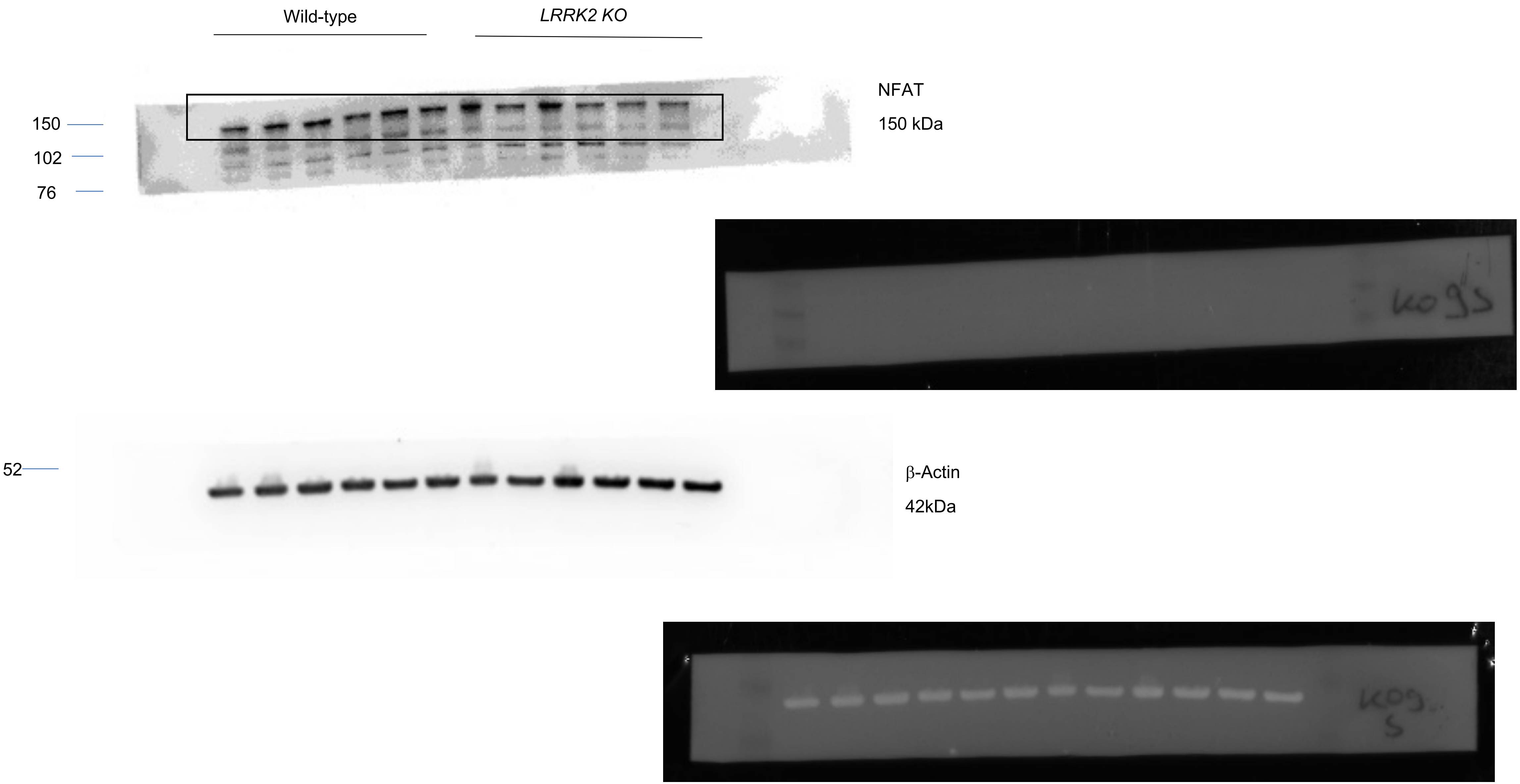

M

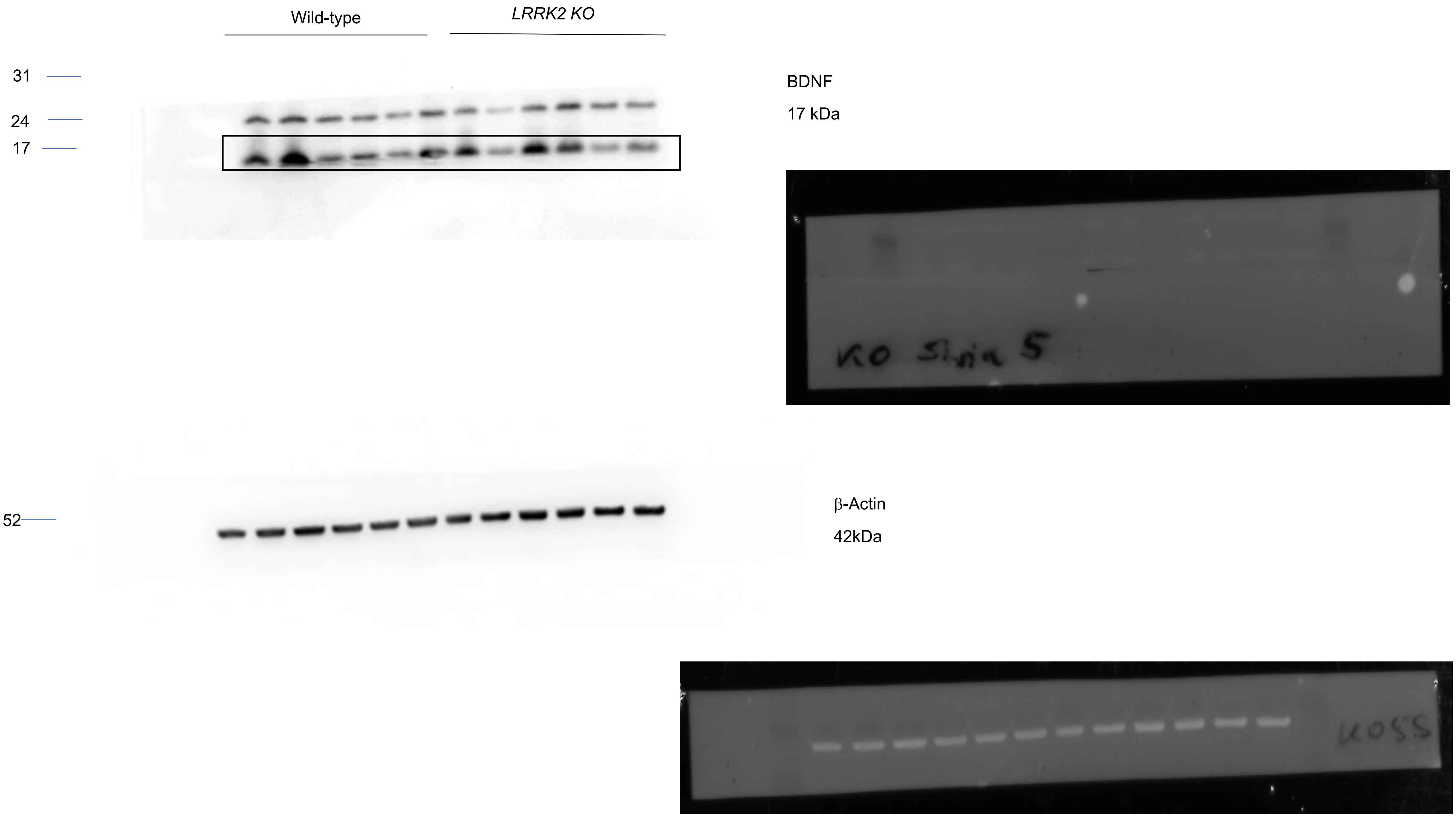

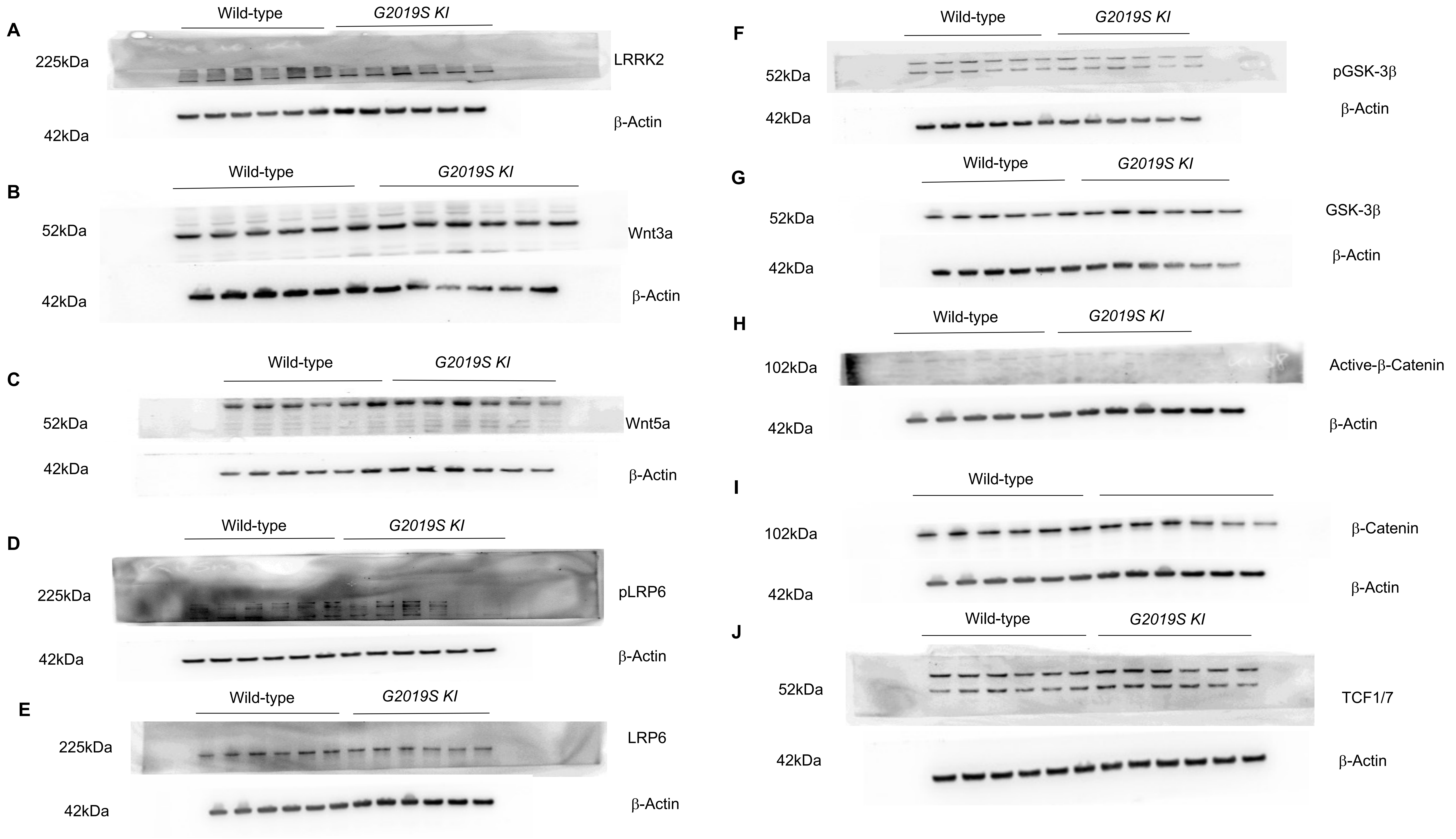
A
Wild-type
G2019S KI
225kDa
LRRK2
42kDa
β-Actin
B
Wild-type
G2019S KI
52kDa
Wnt3a
42kDa
β-Actin
C
Wild-type
G2019S KI
52kDa
Wnt5a
42kDa
β-Actin
D
Wild-type
G2019S KI
225kDa
pLRP6
42kDa
β-Actin
E
Wild-type
G2019S KI
225kDa
LRP6
42kDa
β-Actin
F
Wild-type
G2019S KI
52kDa
pGSK-3β
42kDa
β-Actin
G
Wild-type
G2019S KI
52kDa
GSK-3β
42kDa
β-Actin
H
Wild-type
G2019S KI
102kDa
Active-β-Catenin
42kDa
β-Actin
I
Wild-type
102kDa
β-Catenin
42kDa
β-Actin
J
Wild-type
G2019S KI
52kDa
TCF1/7
42kDa
β-Actin

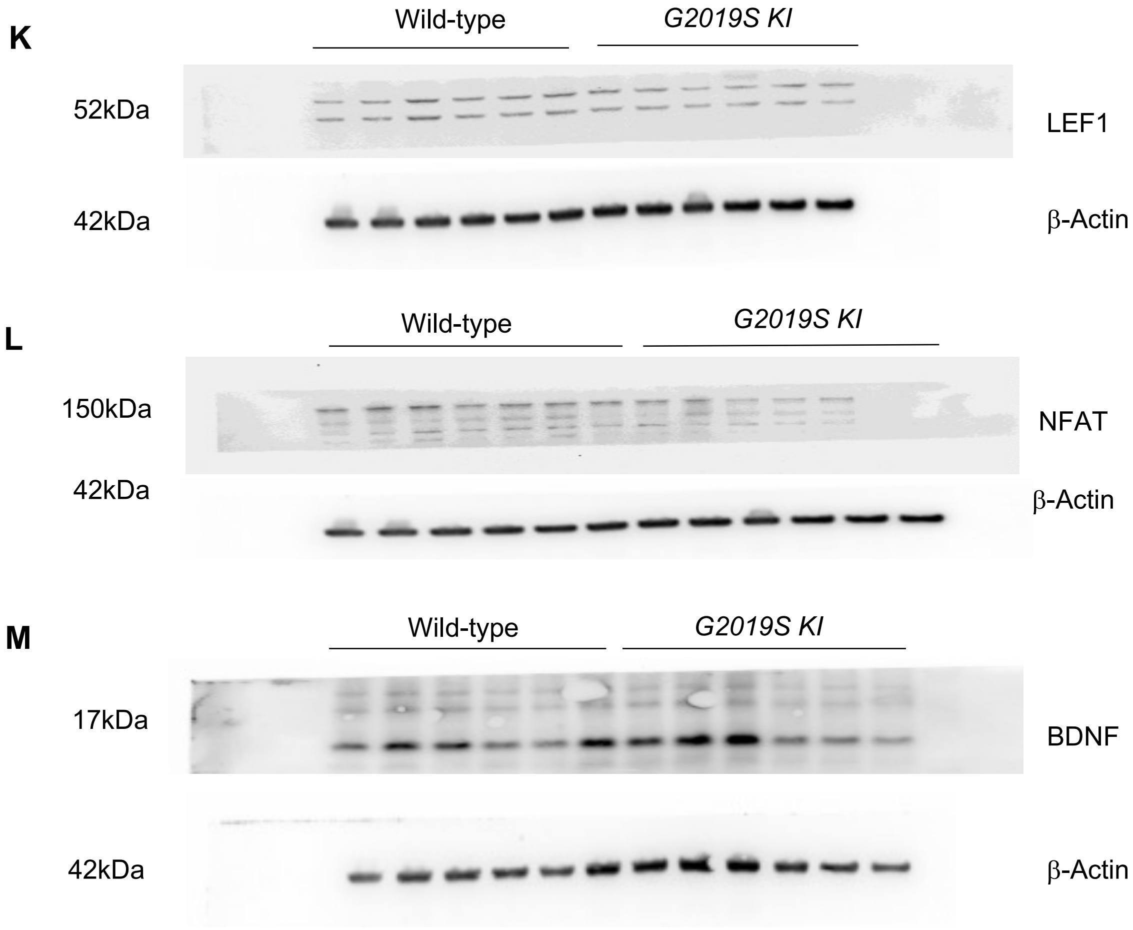
K
Wild-type
G2019S KI
52kDa
LEF1
42kDa
β-Actin
L
Wild-type
G2019S KI
150kDa
NFAT
42kDa
β-Actin
M
Wild-type
G2019S KI
17kDa
BDNF
42kDa
β-Actin

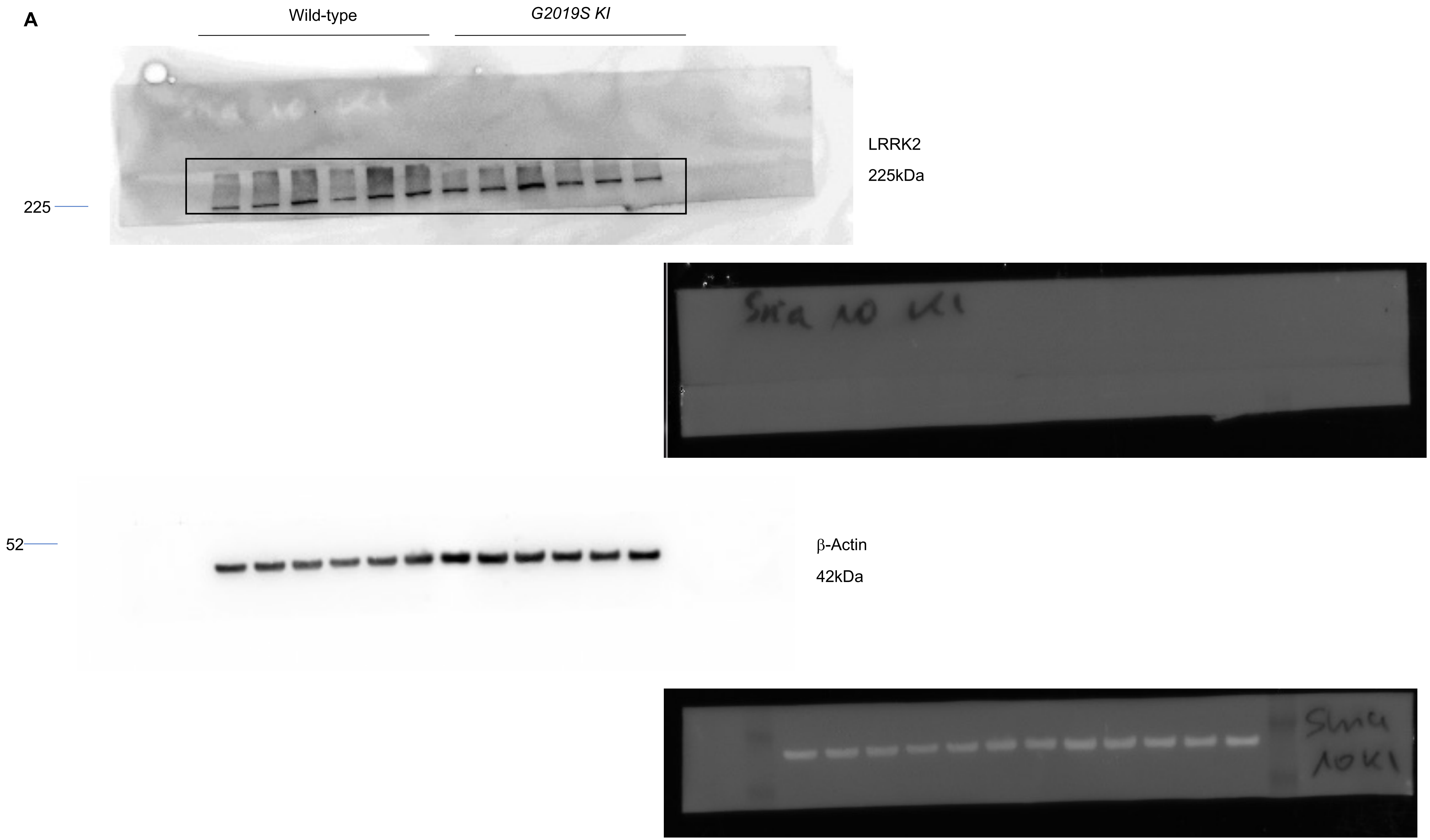
A
Wild-type
G2019S KI
LRRK2
225kDa
225
52
β-Actin
42kDa

B

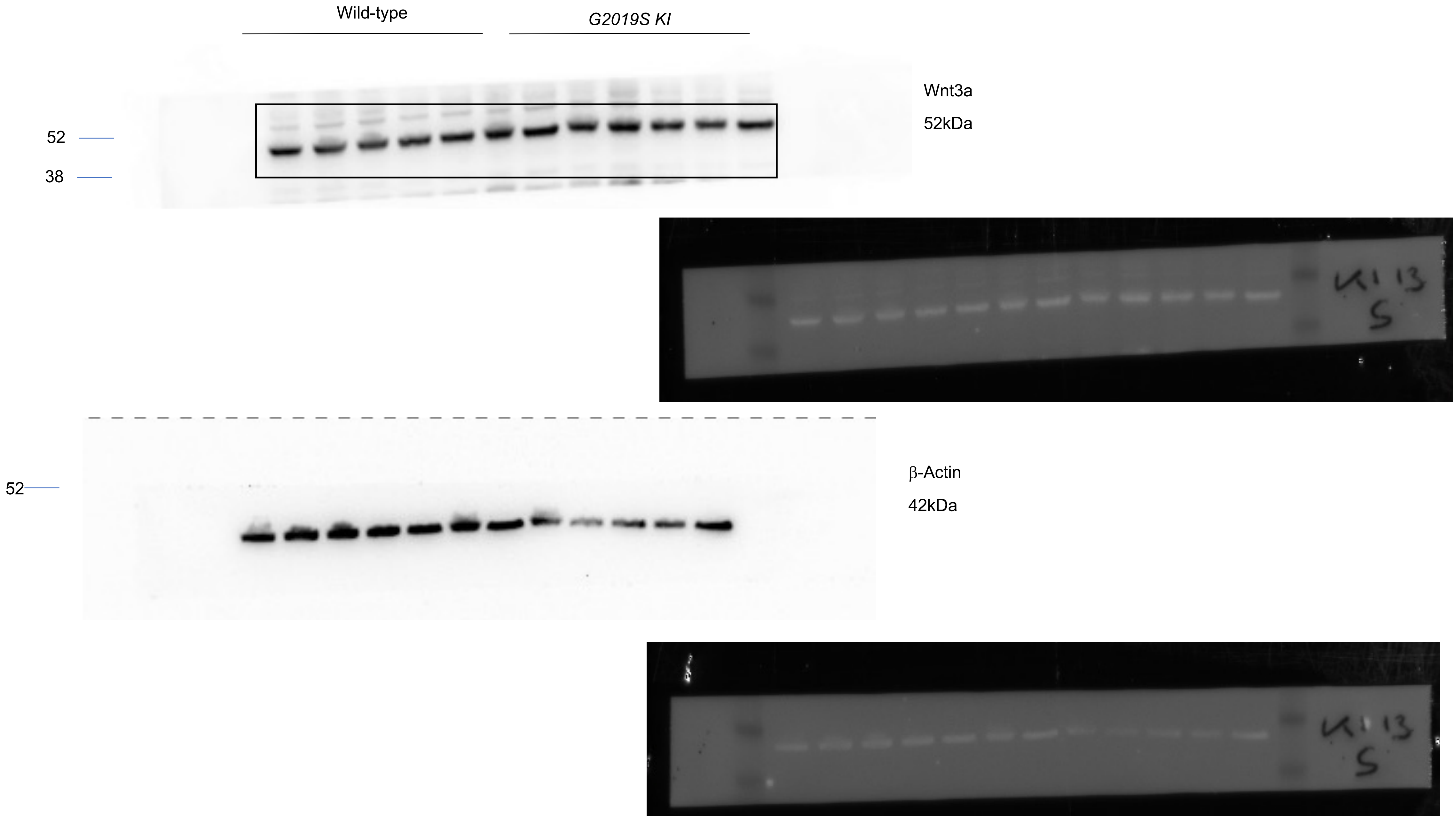
Wild-type
G2019S KI
Wnt3a
52kDa
52
38
KI 13
S
β-Actin
42kDa
52
KI 13
S

C

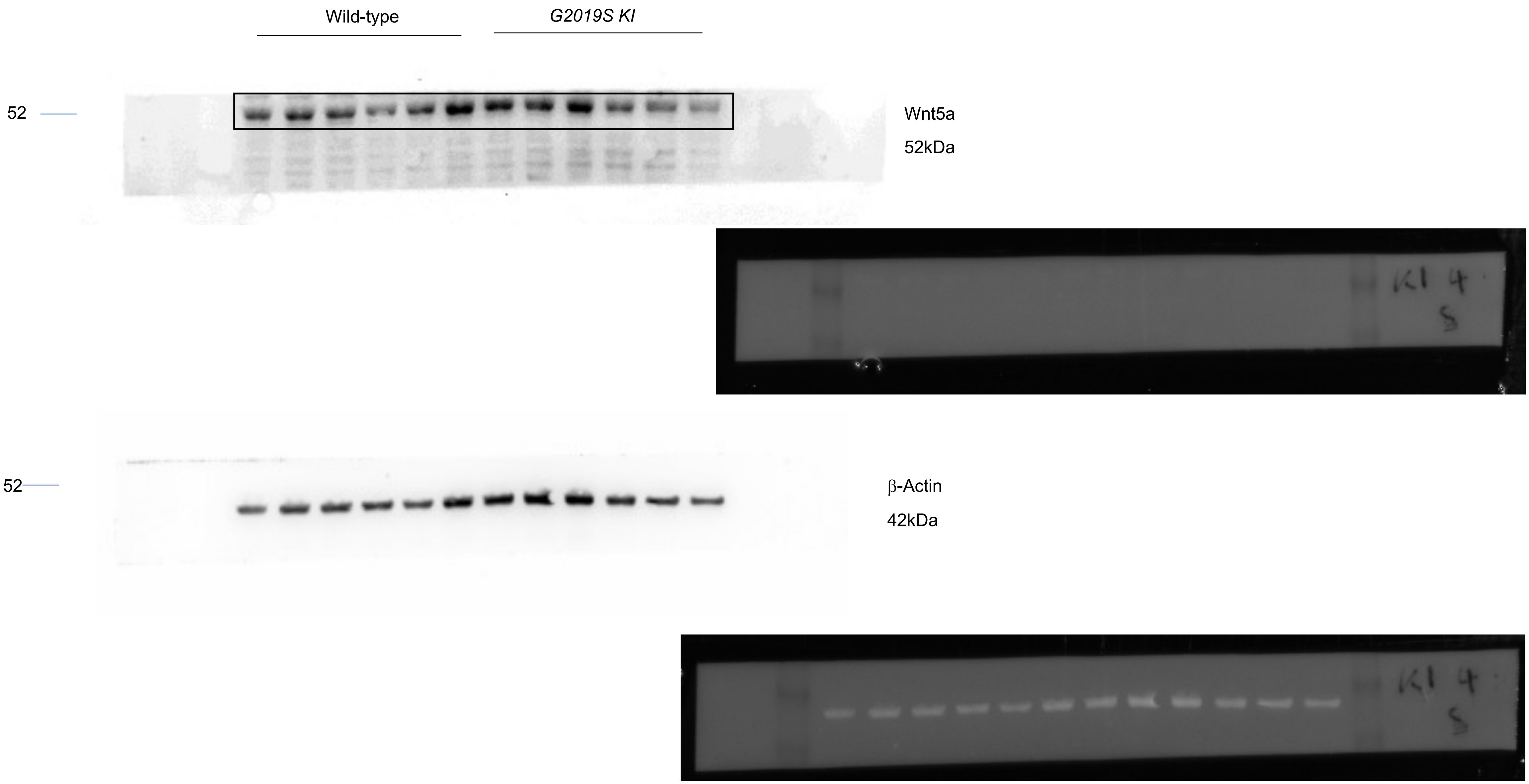

D

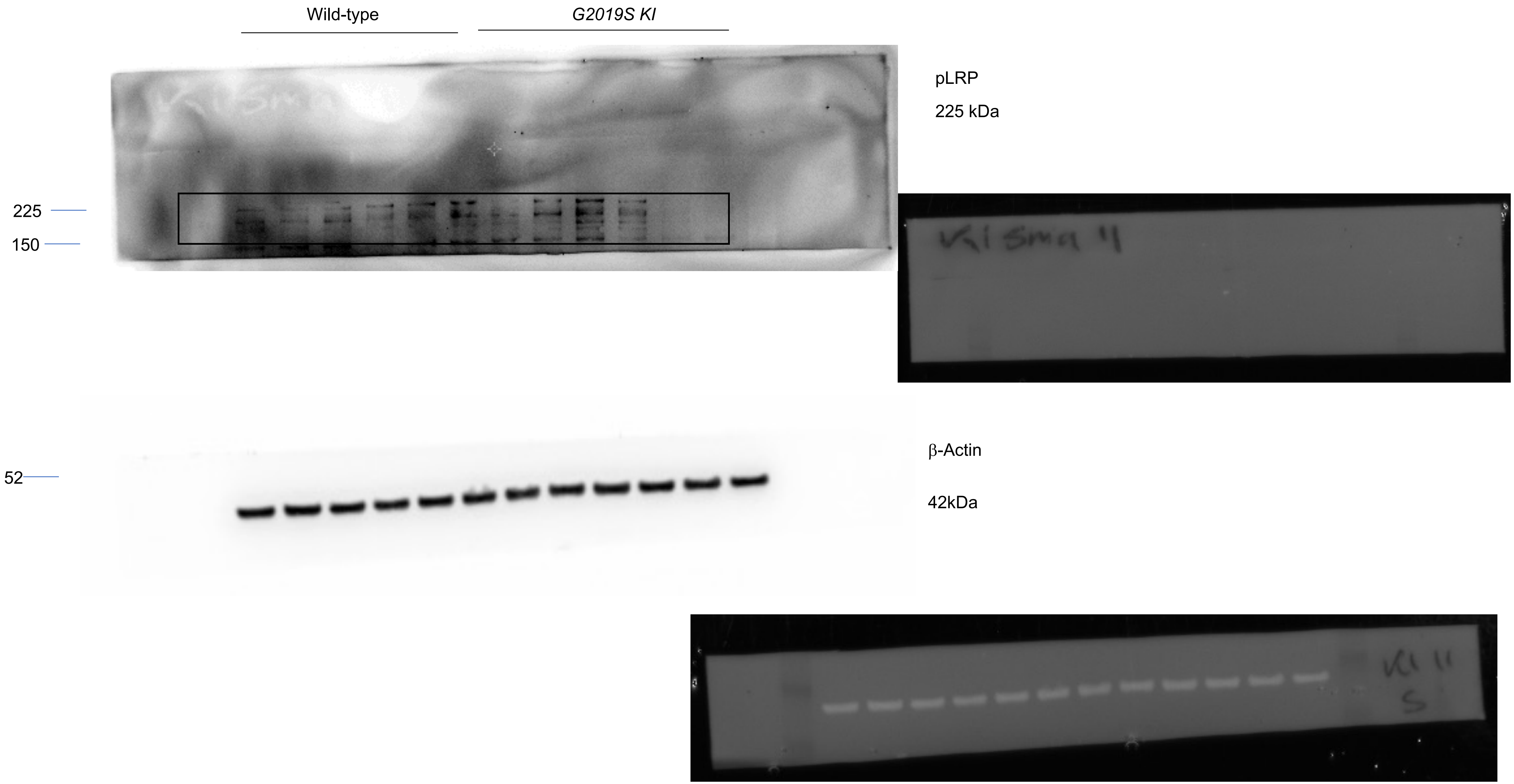

E

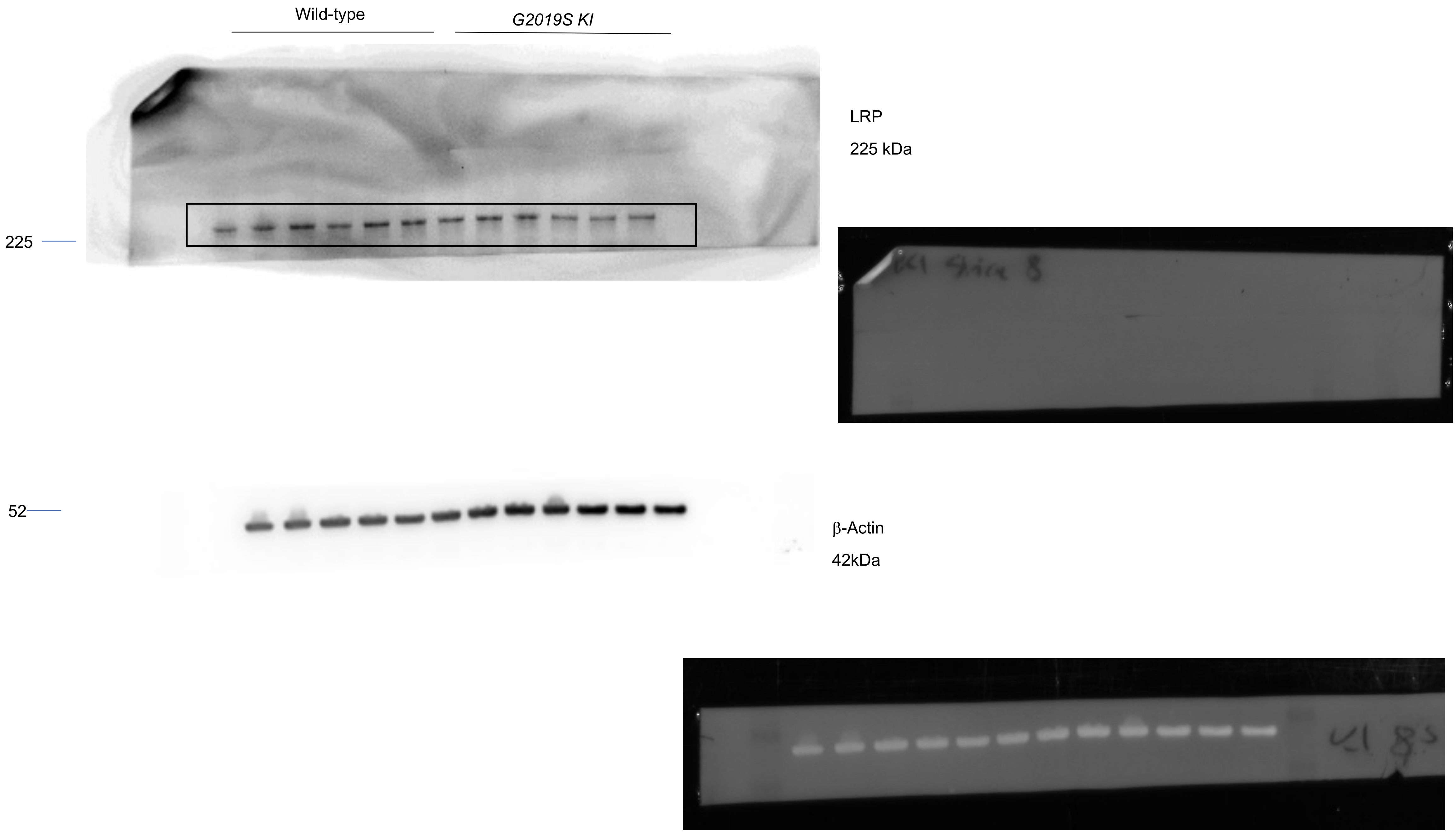

F

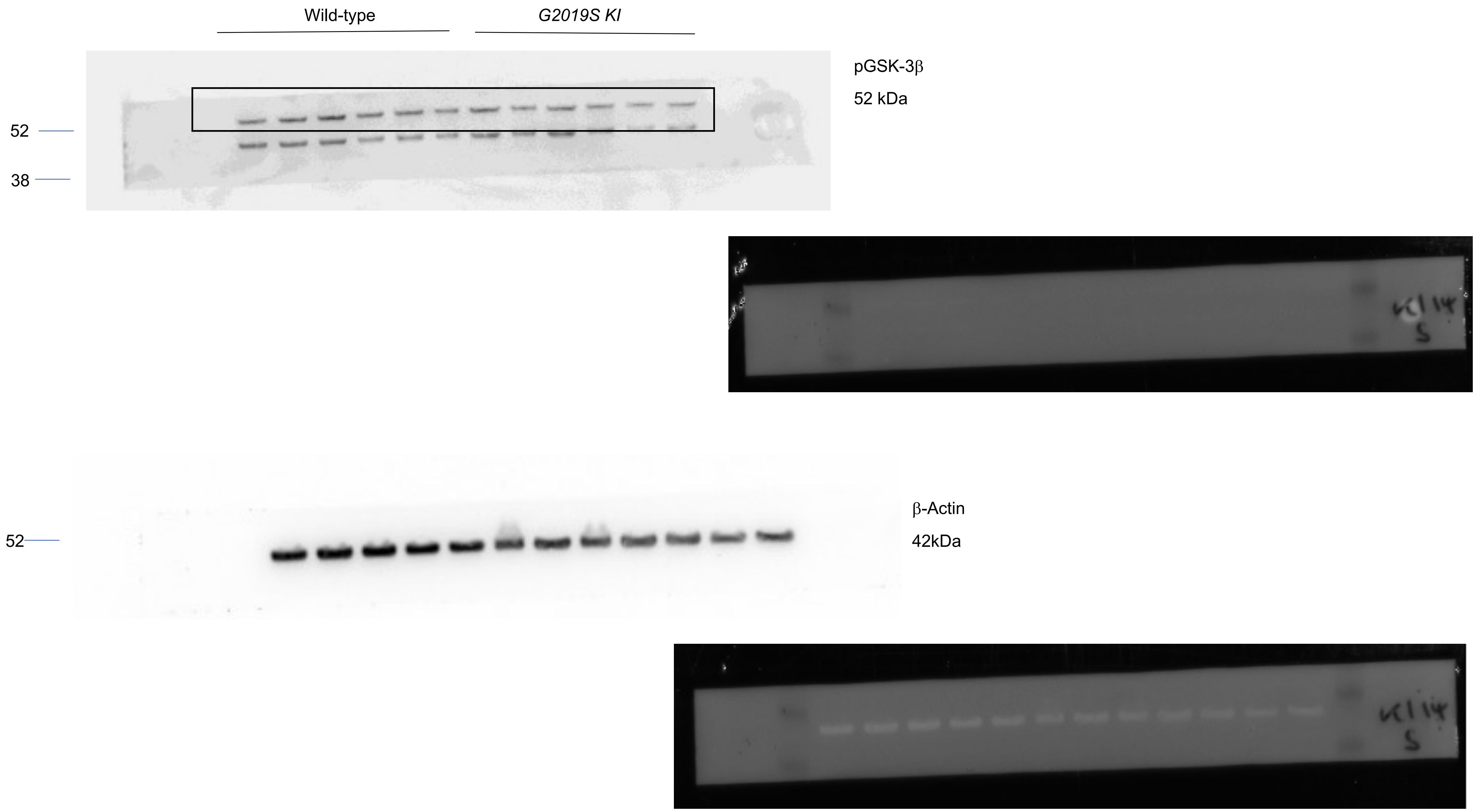

G

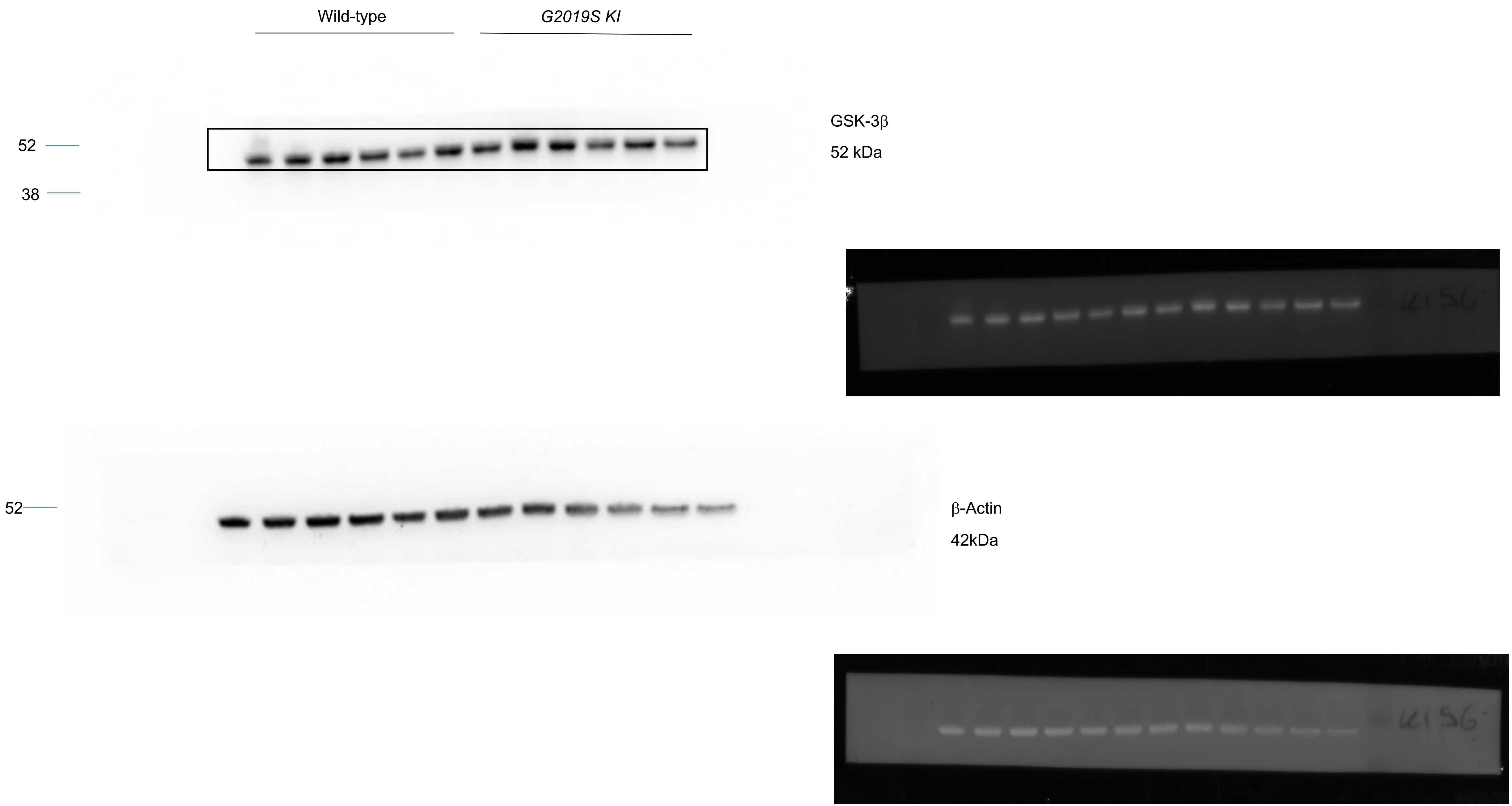

H

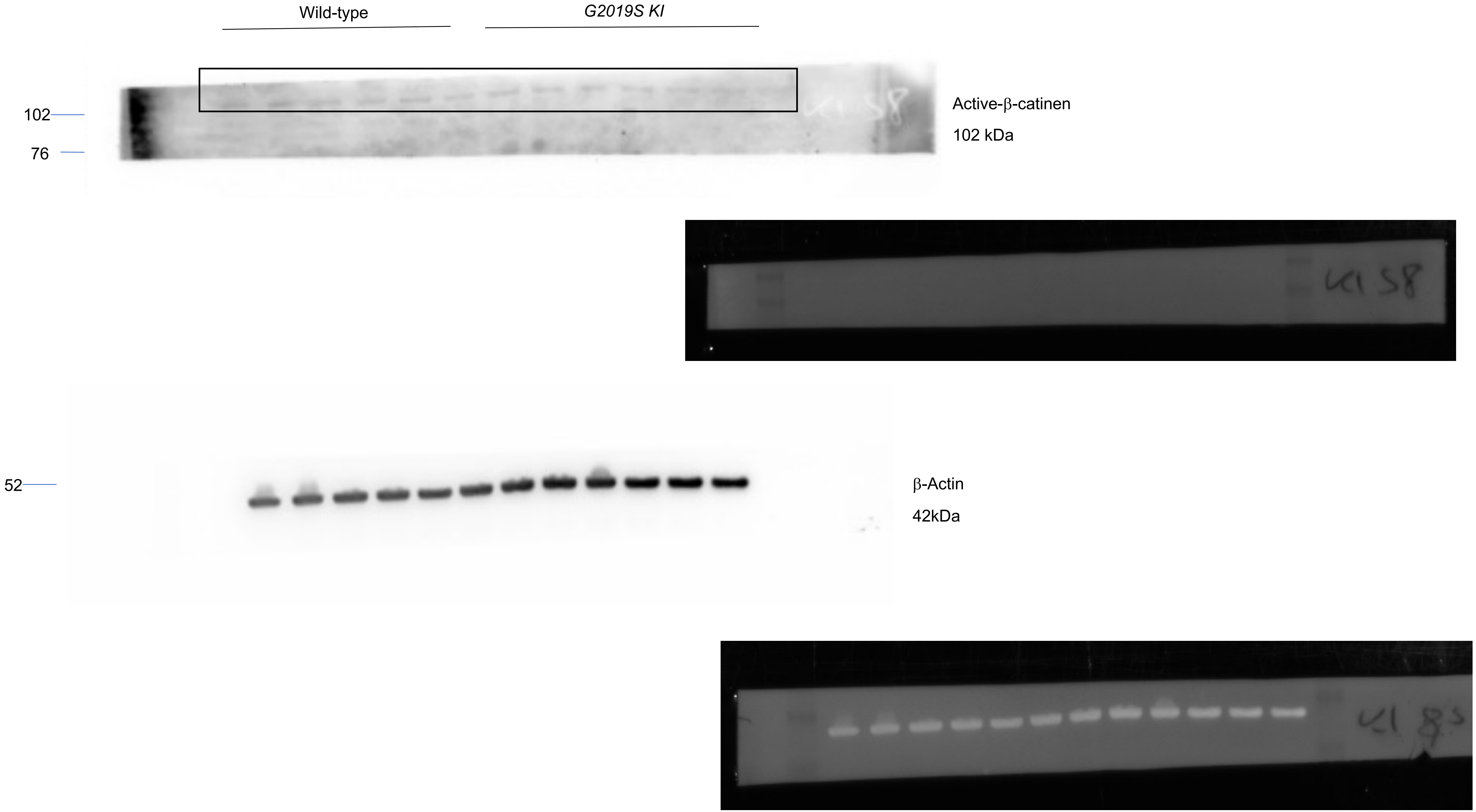
Wild-type
G2019S KI
102
76
Active-β-catinen
102 kDa
52
β-Actin
42kDa

I

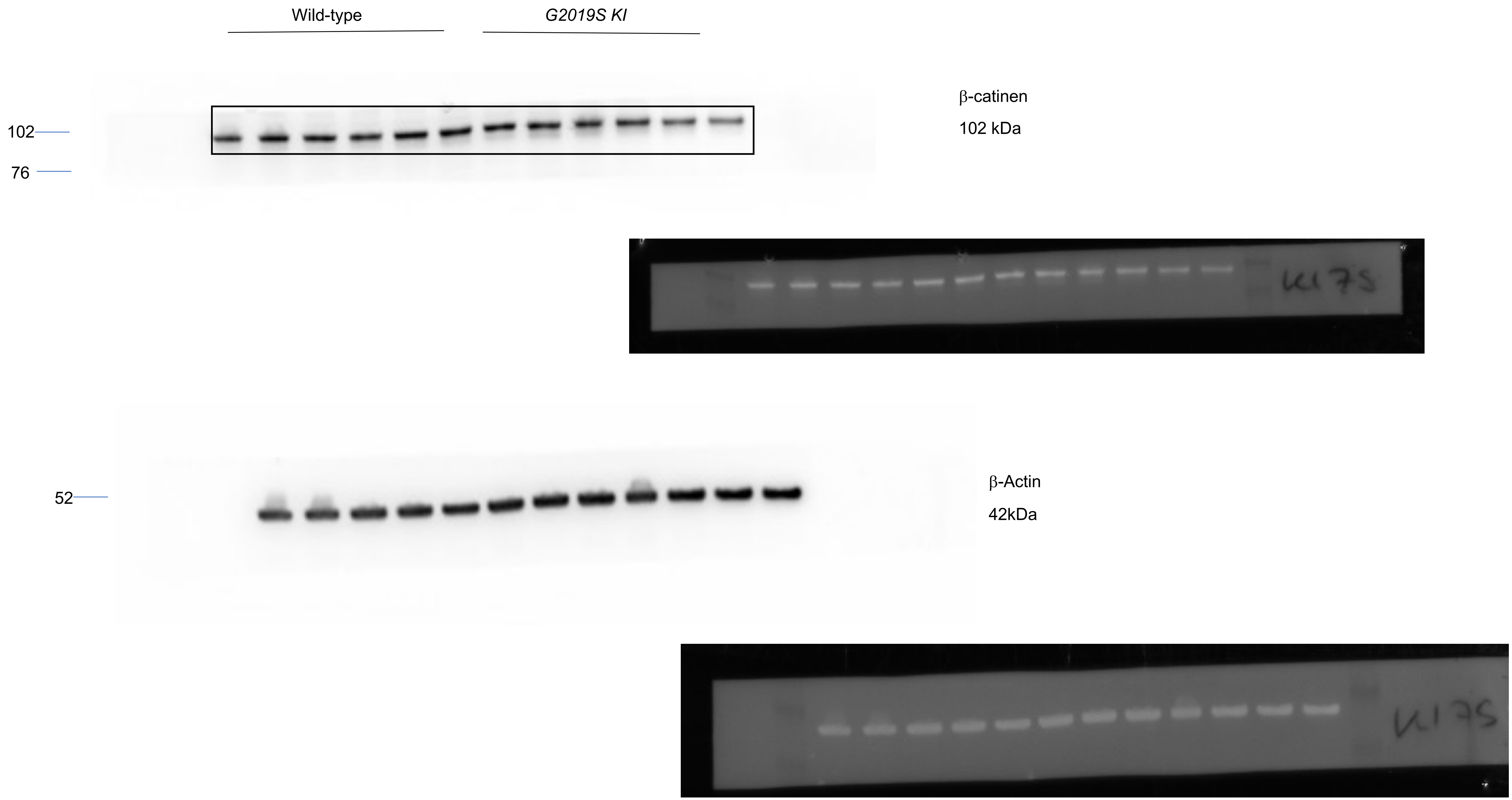

J

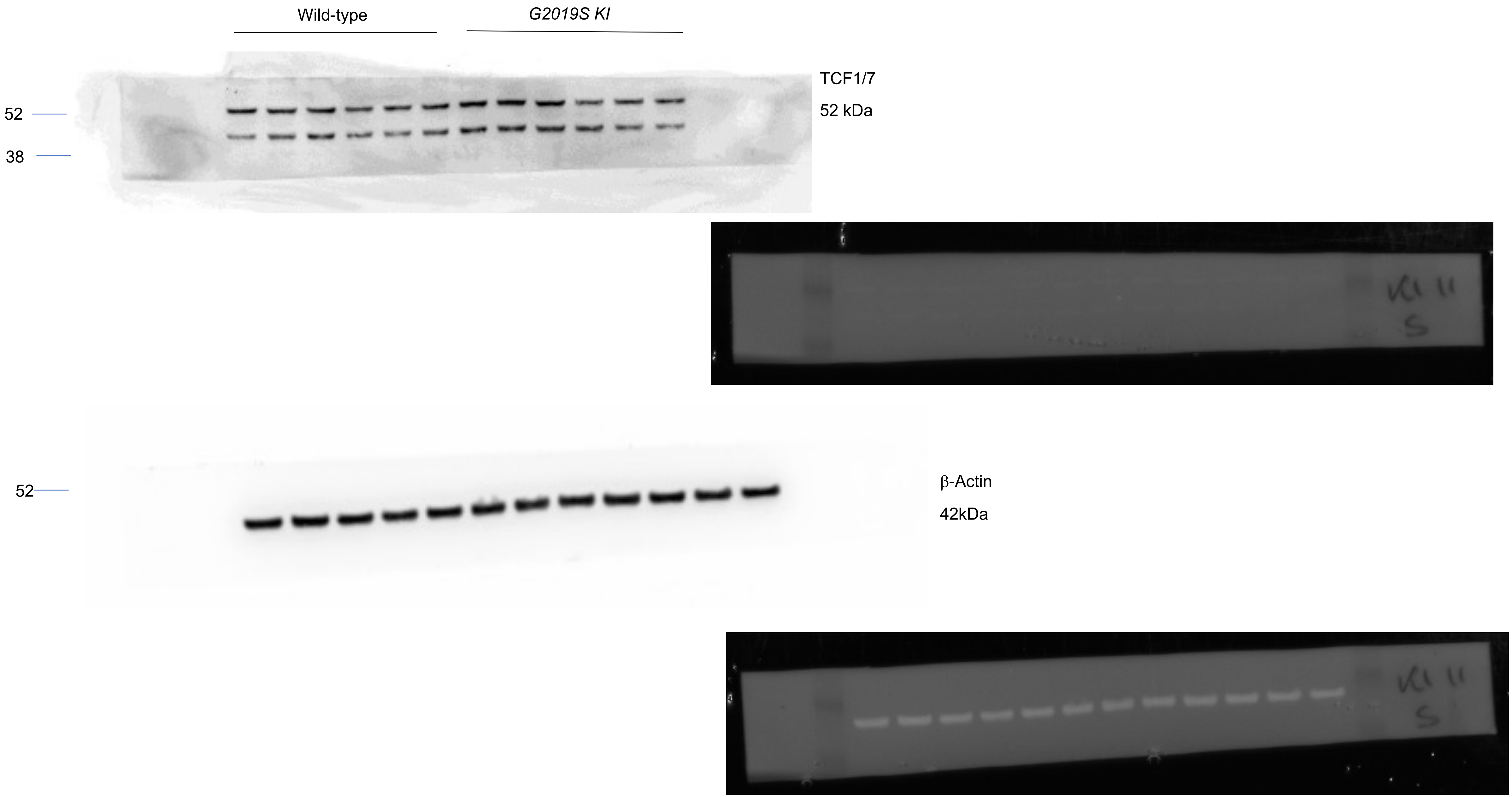

K

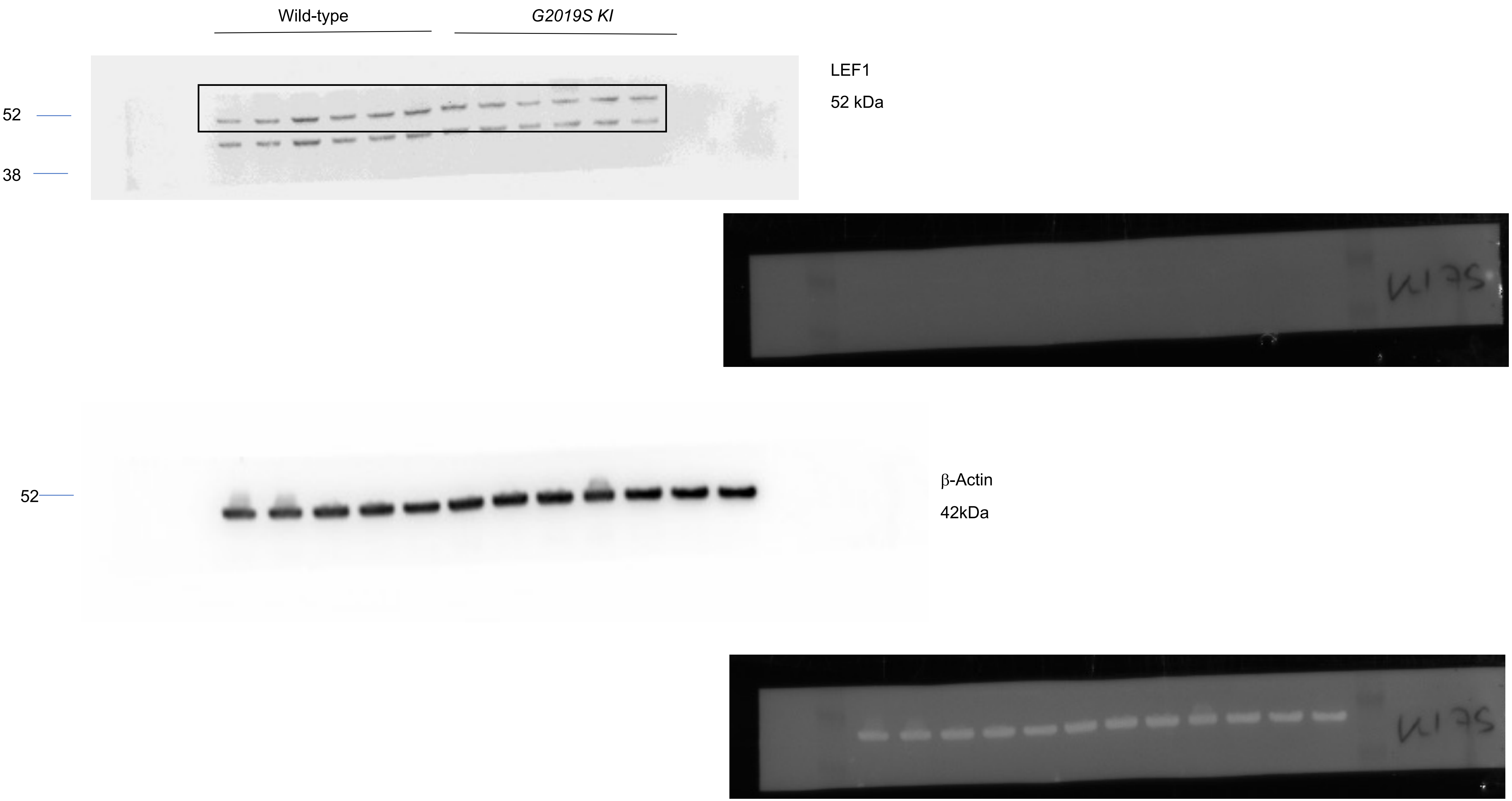

L

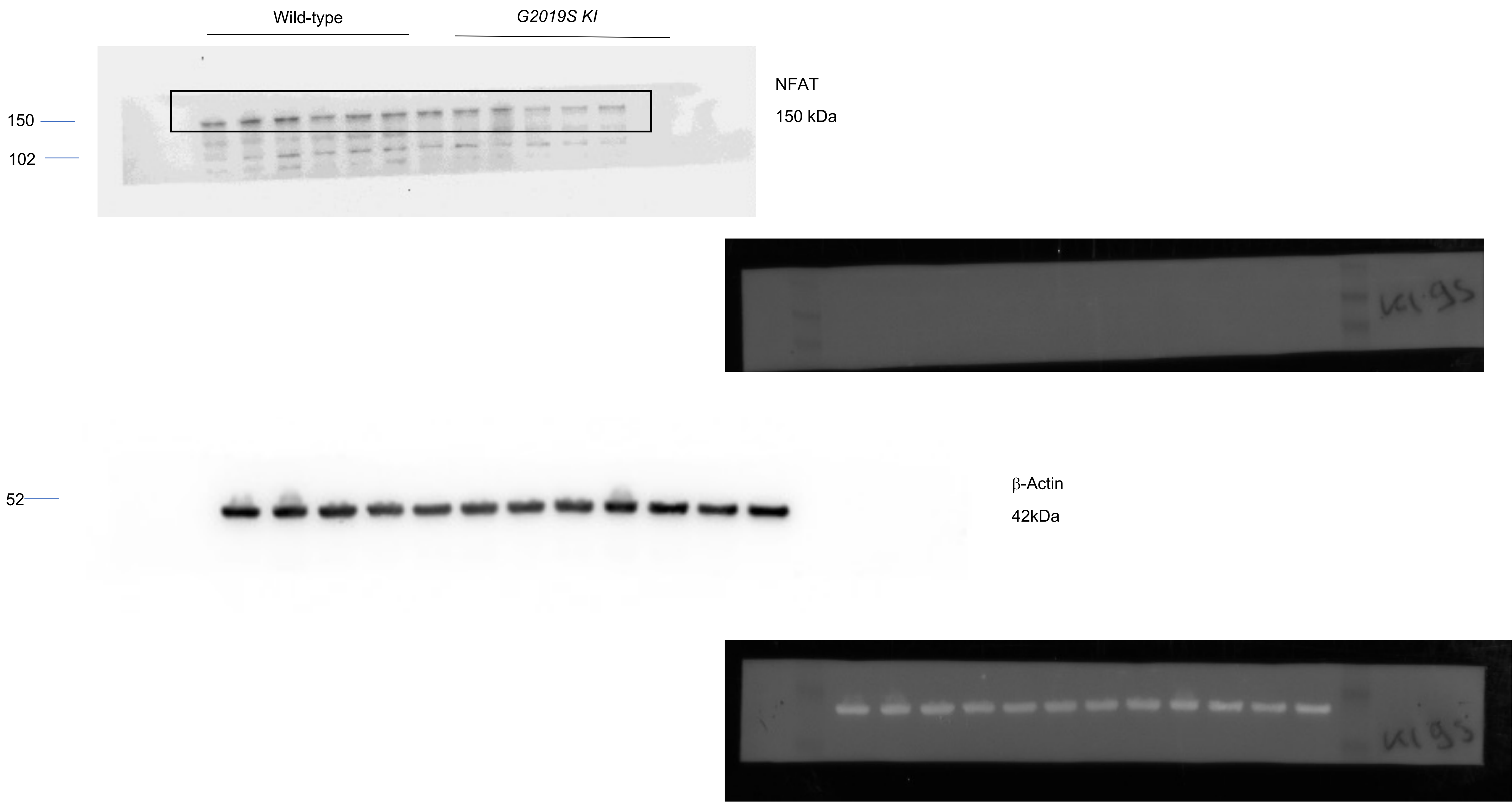

M

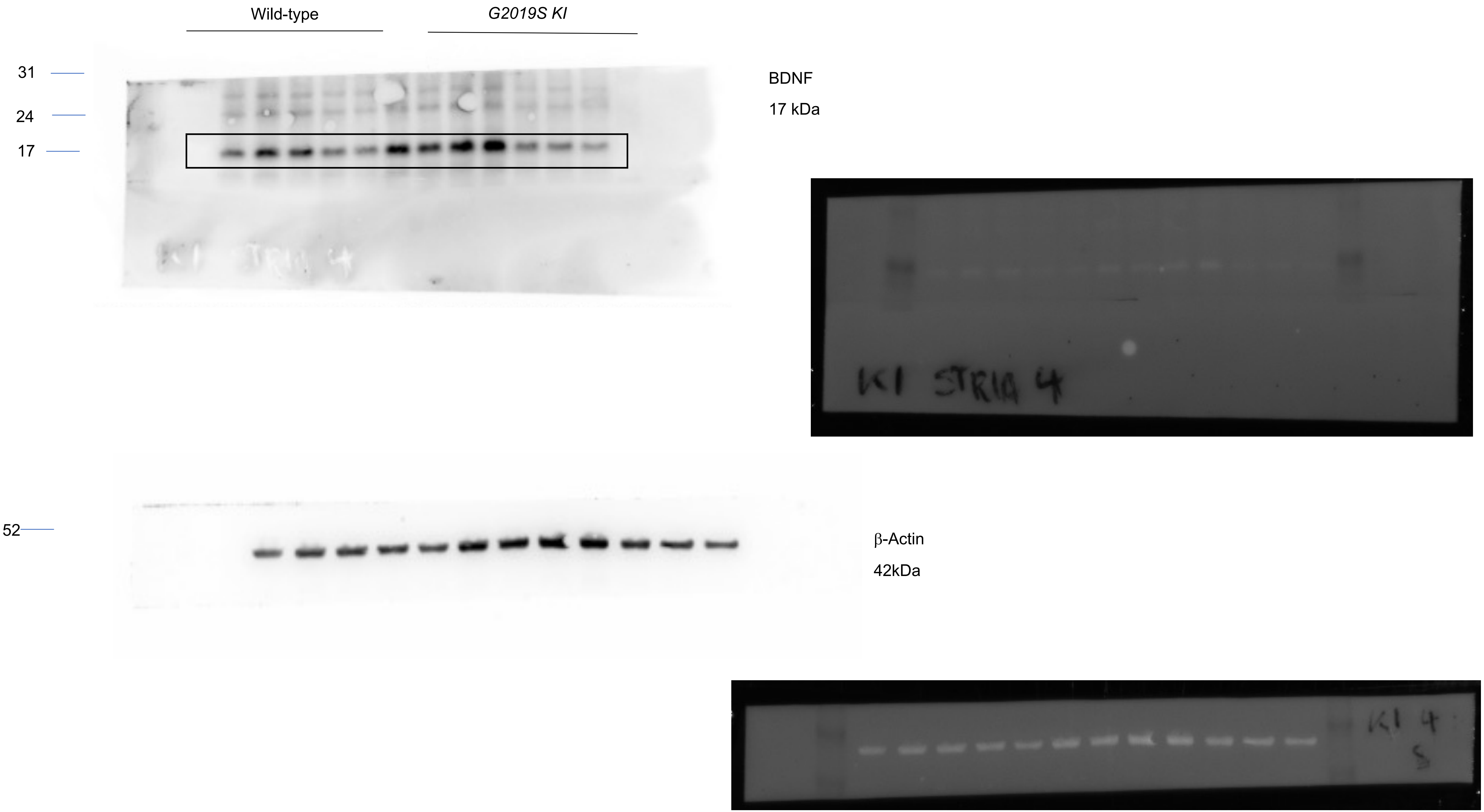
Wild-type
G2019S KI
31
24
17
BDNF
17 kDa
KI STRIA 4
52
β-Actin
42kDa
KI 4
